# CpG-binding protein CFP1 promotes ovarian cancer cell proliferation by regulating *BST2* transcription

**DOI:** 10.1038/s41417-022-00503-z

**Published:** 2022-07-21

**Authors:** Liu-Qing Yang, Han-Yin Hu, Yao Han, Ze-Yi Tang, Jie Gao, Qi-Yin Zhou, Yi-Xuan Liu, Hao-Sa Chen, Tu-Nan Xu, Lei Ao, Ying Xu, Xuan Che, Ya-Bo Jiang, Chun-Wei Xu, Xian-Chao Zhang, Yu-Xin Jiang, Michal Heger, Xiao-Min Wang, Shu-Qun Cheng, Wei-Wei Pan

**Affiliations:** 1 Department of Cell Biology, College of Medicine, Jiaxing University, 118 Jiahang Road, Jiaxing, 314001 China; 2 Department of Anesthesiology, Jiaxing Maternity and Child Health Care Hospital, Affiliated Women and Children Hospital, Jiaxing University, Jiaxing, 314001 Zhejiang Province China; 3 Department of Hepatic Surgery VI, Eastern Hepatobiliary Surgery Hospital, Second Military Medical University, 225 Changhai Road, Shanghai, 200438 China; 4 Department of Pathology, Fujian Cancer Hospital, Fujian Medical University Cancer Hospital, 350014 Fuzhou, Fujian China; 5 Institute of Information Network and Artificial Intelligence, Jiaxing University, 118 Jiahang Road, Jiaxing, 314001 China; 6 Department of Pharmaceutics, Utrecht Institute for Pharmaceutical Sciences, Utrecht University, Utrecht, The Netherlands; 7 Laboratory of Experimental Oncology, Department of Pathology, Erasmus MC, Rotterdam, the Netherlands; 8 G60 STI Valley Industry & Innovation Institute, Jiaxing University, 118 Jiahang Road, Jiaxing, 314001 China

**Keywords:** Oncogenes, Cell biology

## Abstract

Epigenetic alterations have been functionally linked to ovarian cancer development and occurrence. The CXXC zinc finger protein 1 (CFP1) is an epigenetic regulator involved in DNA methylation and histone modification in mammalian cells. However, its role in ovarian cancer cells is unknown. Here, we show that CFP1 protein is highly expressed in human ovarian cancer tissues. Loss of CFP1 inhibited the growth of human ovarian cancer cells, promoted apoptosis, and increased senescence. CFP1 knockdown resulted in reduced levels of SETD1 (a CFP1 partner) and histone H3 trimethylation at the fourth lysine residue (H3K4me3). RNA-sequencing revealed that deletion of CFP1 resulted in mRNA reduction of bone marrow stromal cell antigen 2 (BST2). Bioinformatics analysis and chromatin immunoprecipitation showed that CFP1 binds to the promoter of BST2 and regulates its transcription directly. Overexpression of BST2 rescued the growth inhibitory effect of CFP1 loss. Furthermore, depletion of cullin-RING ubiquitin ligases 4 (CRL4) components *ROC1* or *CUL4A* had significantly inhibited the expression of CFP1 and BST2 similar to MLN4924 treatment that blocked cullin neddylation and inactivated CRL4s. In conclusion, CFP1 promotes ovarian cancer cell proliferation and apoptosis by regulating the transcription of BST2, and the expression of CFP1 was affected by CRL4 ubiquitin ligase complex.

## Introduction

Ovarian cancer is one of the most common malignant tumors and it has the highest mortality rate among all gynecological cancers [1]. Ovarian cancer is insidious, highly invasive, and metastatic [2]. In fact, most patients present with an advanced stage malignancy at the time of diagnosis and the 5-year survival rate of these patients is around 44% [3]. In Western European countries, such as the UK, the incidence rate of ovarian cancer has remained stable for the last three decades, at around 23/100k [4] in women. Women of all ages are at risk for ovarian cancer, although it is most common in individuals aged 65–80 [5]. Common treatments for ovarian cancer include surgery, chemotherapy, and endocrine therapy. However, these treatments improve survival rate only to a limited extent [6]. This is ascribed to ovarian cancer cells developing resistance to chemotherapy and exhibiting a strong propensity to proliferate and become metastatic in the abdominal cavity [7]. Consequently, there is a strong clinical need to develop effective therapeutics for ovarian cancer that not only trigger tumor cell death but also deter its proliferation and metastasis.

Cullin (CUL)-RING E3 ligase (CRL) is the largest ubiquitin ligase family of the ubiquitin-proteasome system [8]. It comprises seven CUL proteins, of which CUL4 is overexpressed in many tumors, including breast cancer, hepatocellular carcinoma, and mesenchymoma [9]. CUL4A/B is a skeletal protein, and its C-terminus is connected to E2 ubiquitin ligase by a small-molecule CUL ring, containing a zinc finger with ubiquitination function. The N-terminus of CUL4A/B binds to the adaptor protein DNA-binding protein 1 (DDB1), which further binds to cognate proteins (e.g., ubiquitin ligase substrates), leading to their ubiquitination [10]. A prerequisite for the activity of CUL4-DDB1 E3 ligase is CUL neddylation [11]. In neddylation, the small-molecule ubiquitin-like molecule neuronal precursor cell-expressed developmentally downregulated protein 8 (NEDD8) is added to the target protein. This is therefore a form of post-transcriptional modification [12]. Soucy et al. found that MLN4924 (pevonedistat), a selective inhibitor of NEDD8-activating enzyme and CRL E3 ligase, induced chromatin licensing and DNA replication factor 1 accumulation [13] and apoptosis [14–16] in human cancer cell lines HCT116 (colon carcinoma), H1299 (non-small cell lung carcinoma), and U87 (glioblastoma) (Supplementary Fig. S1).

The CXXC zinc finger protein 1 (CFP1), encoded by the *CXXC1* gene, contains a plant homeodomain (PHD) at its N-terminus that directly binds to tri-methylated histone 3 at the fourth lysine residue (H3K4me3) [17]. The SET1 interaction domain (SID) is required for CFP1 binding to the histone H3K4 methyltransferases SETD1A and SETD1B [18]. The CXXC domain on CFP1 binds to the phosphate main chain of negatively charged DNA through its charged surface forming multiple main chain hydrogen bonds with CpG islands [19]. After CFP1 binds to the DNA sequence of unmethylated CpG dinucleotides through CXXC, it inhibits CpG methylation in this region, and recruits the SETD1A complex to methylate H3K4 to activate gene transcription [20]. In gastric cancer patients, CFP1 protein expression negatively correlates with survival [21].

In the present study, we first found that MLN4924 (pevonedistat), as an inhibitor of the CRL4 ubiquitin ligase complex, reduced the expression of CFP1 in vivo and in vitro. Furthermore, CFP1 knock-out suppressed ovarian cancer cell proliferation in vitro and in vivo. We then explored the molecular mechanism of CFP1-mediated bone marrow stromal antigen 2 (BST2) transcriptional activity and identified how CFP1 affects ovarian cancer cell transcriptome. Our study not only identified potential CFP1 target genes for the regulation of cell proliferation but also novel biomarkers and therapeutic strategies for ovarian cancer.

## Results

### CFP1 protein is highly expressed in human ovarian cancer tissues and cells

**Fig. 1:**
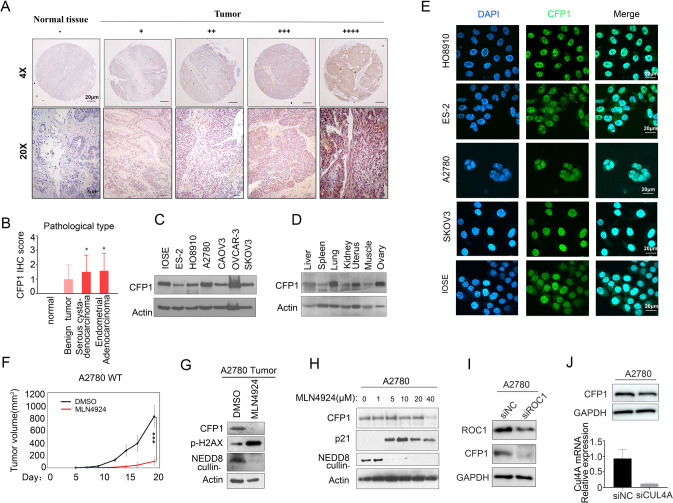
Expression pattern of CFP1 in ovarian cancer tissues and ovarian cancer cells. **A** Immunohistochemistry for CFP1 protein expression in human ovarian cancer tissues. Scale bars, 5 and 20 μm. **B** Statistical analysis of CFP1 protein expression in different pathological types of 158 human ovary and ovarian cancer tissues. **C** Immunoblotting for CFP1 expression in ovarian cancer cells (OVCAR-3, HO8910, ES-2, SKOV3, CAVO3, and A2780), and immortalized mouse ovarian surface epithelium (IOSE). Actin was used as the loading control. **D** Immunoblotting for CFP1 expression levels in mouse tissues (liver, spleen, lung, kidney, uterus, muscle, and ovary). **E** Immunofluorescence assay for CFP1 (green) localization in ovarian cells (HO8910, ES-2, SKOV3, and A2780) and IOSE cells. Nuclei were stained with DAPI (blue). Scale bar, 20 μm. **F** MLN4924 inhibited tumor growth in mice. 1 × 10^6^ A2780 cells were injected into the flank of nude mice. After tumor growth had reached roughly 200 mm^3^, the mice were randomly assigned to the MLN4924-treated group or control group. The single injection dose was 2 mg/kg. **P < 0.01, ***P < 0.001. The *P* value of tumor volume was determined using two-way ANOVA. **G** Immunoblotting revealing that MLN4924 inhibited CFP1 expression in vivo. A2780 tumor samples were harvested and then subjected to immunoblot analysis with the indicated antibodies. **H** Immunoblotting revealing that MLN4924 inhibited CFP1 expression in ovarian cancer (A2780) cells. Cells were subject to control or MLN4924 treatment and protein was subjected to immunoblot analysis with the indicated antibodies. **I** Immunoblotting results for ROC1 siRNA depletion efficiency and CFP1 expression in A2780 cells. **J** Western blot and qRT-PCR results for CUL4A siRNA depletion efficiency and CFP1 expression.

To ascertain the potential role of CFP1 protein in ovarian cancer, immunohistochemistry was performed on ovarian cancer tissue microarrays (TMAs) to examine the expression of CFP1 protein in normal human ovarian tissues (*n* = 4), benign tumor (*n* = 5), and ovarian cancer tissues (*n* = 158) (Table 1). Based on staining intensity, we classified the samples into five groups, from the weakest (–) to the strongest (++++) staining intensity (Fig. 1A). As summarized in Table 1, strong CFP1 signal was observed in 125 ovarian cancer samples: (+) in 50 samples (31.6%); (++) in 40 samples (25.3%); (+++) in 26 samples (16.4%); and (++++) in 9 samples (5.7%). Partial or complete loss of CFP1 staining was observed in 33 samples. As shown in Fig. 1B and Supplementary Fig. S2A, the expression of CFP1 was high in ovarian cancer tissue, especially in endometrioid carcinoma and serous cystadenoma (*P* < 0.05). Immunoblotting and quantitative real-time PCR (qRT-PCR) revealed that CFP1 was highly expressed in immortalized mouse ovarian surface epithelia (IOSE), A2780, and ES-2 cells, as well as in mouse ovary tissues (Fig. 1C, D and Supplementary Fig. S2B). Furthermore, CFP1 was highly expressed in a variety of ovarian cancer cells and localized predominantly to cell nuclei (Fig. 1E). These results suggested that CFP1 may play an important role in ovarian tumor biology.

**Table 1:** Demographics and medical data of the ovarian cancer patient cohort (*n* = 158).

Parameter	Mean (standard deviation) (range)
Age	48.38 (13.53) (17–82)
	Number (%) of patients
Pathological type	
Normal	4 (2.5)
Benign tumor	5 (3.1)
Serous papillary	74 (46.8)
Endometrial	53 (33.5)
Mucinous	31 (19.6)
Pathological grading	
I	48 (30.4)
II	40 (25.3)
III	66 (41.7)
T stage	
T1	76 (48.1)
T2	56 (35.4)
T3	26 (16.4)
N stage	
N0	150 (94.9)
N1	7 (4.5)
M stage	
M0	135 (85.4)
M1	22 (14.0)
CFP1 immunohistochemistry score	
Negative (0)	38 (24.1)
+ (1)	50 (31.6)
++ (2)	40 (25.3)
+++ (3)	26 (16.4)
++++ (4)	9 (5.7)

### MLN4924 decreased CFP1 protein expression in vivo and in vitro

We have previously observed that CRL4 E3 ubiquitin ligase was a potential drug target in ovarian cancers and that MLN4924 treatment suppressed tumor cell growth in vitro [22]. In an effort to expand on these findings, we treated BALB/c-nude mice bearing A2780 and ES-2 xenografts with the CRL4 E3 ubiquitin ligase inhibitor MLN4924 twice a week, for 4 weeks. Intraperitoneal injection of MLN4924 significantly inhibited tumor proliferation (*P* < 0.01) (Fig. 1F and Supplementary Fig. S2C). Inhibition of CRL4 E3 ubiquitin ligase activity nullified CFP1 and NEDD8 protein levels and induced DNA double-strand breaks (Fig. 1G and Supplementary Fig. S2D). Next, ovarian cancer (A2780 and ES-2) cells were subjected to increasing doses of MLN4924 treatment and it was found that MLN4924 >20 µM inhibited the expression of CFP1. At MLN4924 >5 µM, NEDD8 expression was inhibited while P21 accumulation was promoted (Fig. 1H). To confirm that the pharmacological effects of MLN4924 were due to disrupting CRL4s, we silenced RBX1 (ROC1) and CUL4A, the core component of CRL complexes, in ovarian cancer A2780 and ES-2 cells. *Roc1* and *Cul4a* were efficiently knocked down by using small interfering RNA (siRNA) oligos, as shown by western blot and qRT-PCR results. *Roc1* and *Cul4a* depletion inhibited the expression of CFP1 protein (Fig. 1I, J and Supplementary Fig. S2F). These results suggested that inhibiting CRL4 E3 ubiquitin ligase activity may affect ovarian cancer cell proliferation through the regulation of CFP1 protein expression in vivo and in vitro.

### CFP1 knock-out inhibits ovarian cancer cell proliferation and clone formation

**Fig. 2:**
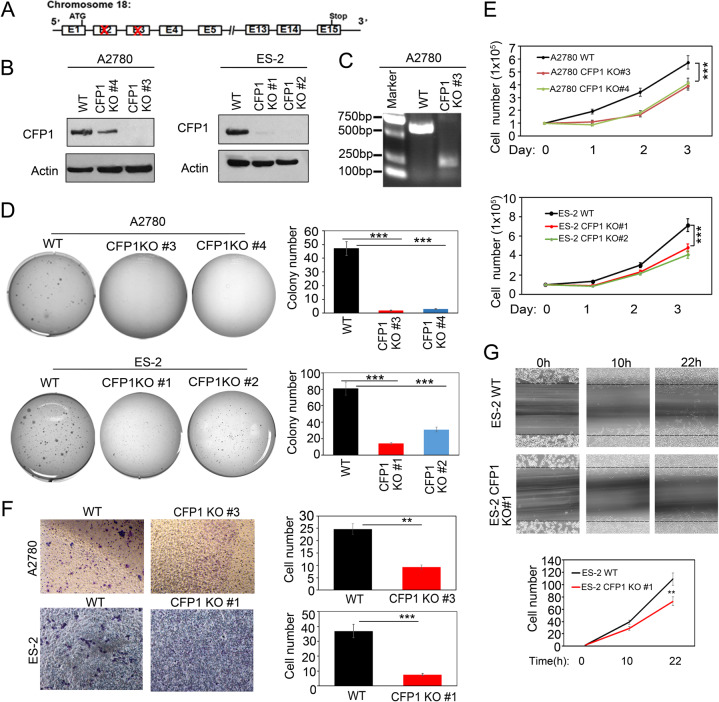
CFP1 deficiency inhibits ovarian cancer cell proliferation, clone formation, and metastatic ability. **A** Diagram showing the strategy of *CFP1 knock-out* in ES-2 and A2780 cells. **B** CFP1-deleted cells were subjected to western blot analysis with anti-CFP1 and anti-Actin antibodies in ES-2 and A2780 cells. Two independent clones (#4 and #3 in A2780; #1 and #2 in ES-2) are shown. **C** CFP1-deleted cells were subjected to PCR in A2780 CFP1 KO #3. **D** CFP1 knock-out inhibited anchorage-independent growth of A2780 and ES-2 cells in vitro. CFP1 knock-out in two independent clones (#4 and #3 in A2780; #1 and #2 in ES-2) is shown. Soft-agar colony-formation assay was performed, and the colonies were stained with crystal violet for quantification. ***P < 0 .001. **E** CFP1 knock-out suppressed ovarian cancer cell growth. A2780 and ES-2 WT and CFP1-deleted cells (1 × 10^5^) were plated in six-well culture dishes and viable cells were counted after trypan blue staining. CFP1 knock-out in two independent clones (#4 and #3 in A2780; #1 and #2 in ES-2) is shown. This experiment was replicated three times. The error bars represent SD. ***P < 0.01, two-way ANOVA test. **F** Transwell experiment for the migration capability of A2780 and ES-2 cells. A2780 and ES-2 WT and CFP1-deleted cells (1 × 10^5^) were plated in the upper surface of the transwell and then stained with hematoxylin and eosin. CFP1 knock-out clones (#3 in A2780; #1 in ES-2) are shown. This experiment was replicated three times. The error bars represent SD. **P < 0.01, ***P < 0.001, two-way ANOVA test. **G** Wound-healing assay for the migration capability of ES-2 WT and CFP1-deleted 1# cells cultured in six-well plates. Data are mean ± SD from three independent experiments. Student’s *t*-test was applied. **P < 0.01.

To investigate how inhibition of CRL4 E3 ubiquitin ligase activity inhibits ovarian cancer cell proliferation through CFP1, we used the clustered regularly interspaced short palindromic repeats (CRISPR)/Cas9 system to knockout *CFP1* in ES-2 and A2780 cells (Fig. 2A). We obtained several *CFP1* partial knock-out cell lines and confirmed the knockout efficiency by PCR and immunoblotting of the CFP1 protein (Fig. 2B, C). Two different clones generated by two independent CRISPR-guide sequences were used for this study. We demonstrated that CFP1 partial knock-out significantly inhibited (A2780) or delayed (ES-2) the clone-forming ability of ovarian cancer cells (Fig. 2D) and reduced cell proliferation (Fig. 2E). To verify the role of CFP1 in ovarian cancer cell invasion, we performed the transwell assay and found that CFP1 knock-out significantly inhibited the invasive ability of ovarian cancer cells (Fig. 2F). Finally, CFP1 knock-out inhibited cell migration in a scratch-wound assay (Fig. 2G). In summary, these results evidenced that CFP1 knock-out inhibits tumor cell proliferation and migration of ovarian cancer cells.

### Loss of CFP1 induces cell cycle arrest and promotes apoptosis in vitro

**Fig. 3:**
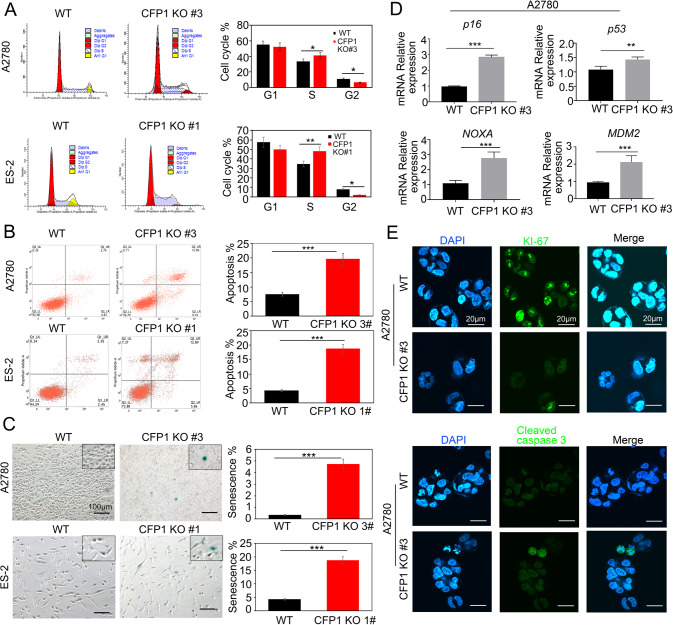
CFP1 knock-out affects the cell cycle and promotes cell apoptosis and senescence. **A** CFP1 knock-out affected the cell cycle. WT and CFP1-deleted cells of ES-2 and A2780 were cultured overnight. CFP1-deleted clones (#3 in A2780; #1 in ES-2) are shown. Propidium iodine staining detected the cell cycle stage by FACS. *P < 0.05, **P < 0.01, two-way ANOVA test. **B** CFP1 knock-out promoted apoptosis. WT and CFP1-deleted cells of ES-2 and A2780 were cultured overnight. CFP1-deleted clones (#3 in A2780; #1 in ES-2) are shown. Cells were cultured and stained with PE Annexin V. ***P < 0.001, two-way ANOVA. **C** CFP1 knock-out promoted senescence. WT and CFP1-deleted cells of ES-2 and A2780 were cultured and stained for the senescence marker SA-β-gal. CFP1-deleted clones (#3 in A2780; #1 in ES-2) are shown. ***P < 0.001, two-way ANOVA. Scale bar, 100 μm. **D** qRT-PCR detection of cell cycle-related factors in A2780 WT and CFP1-deleted clone #3 cells. The error bars represent SD. Student’s *t*-test was applied. ns (*P* > 0.05); **P < 0.01; ***P < 0.001. **E** Immunofluorescence detection of cell proliferation and apoptosis in A2780 cells. WT and CFP1-deleted clone #3 cells were subjected to immunostaining with KI-67 and Cleaved caspase 3 antibody (green) along with DAPI for DNA (blue). Scale bar, 20 μm.

The CFP1 knock-out resulted in decreased G2 phase and increased S phase in A2780 and ES-2 cells (Fig. 3A). Cell cycle arrest, induced by thymidine, was more pronounced in CFP1-deficient ovarian cancer cells, whereas wild-type (WT) cells recovered from cell cycle arrest more quickly (Supplementary Fig. S2G). Immunoblotting revealed that the expression of CFP1 protein decreased with the start of metaphase (Supplementary Fig. S2H). CFP1 knock-out ovarian cancer cells were associated with an increased rate of apoptosis (Fig. 3B) and senescence (Fig. 3C). The p53 and p16/pRB signaling pathways are important in senescence and cell cycle regulation [23, 24]. qRT-PCR revealed that CFP1 knock-out increased the expression of *p16*, *p53*, and p53 downstream genes *NOXA* and *MDM2* in A2780 and ES-2 cells (Fig. 3D and Supplementary Fig. S2I). We further observed a significant decrease in proliferation marker (Ki-67) and an increase in pro-apoptotic signaling (cleaved caspase 3) in CFP1-deleted A2780 and ES-2 cells (Fig. 3E and Supplementary Fig. S3B). These results suggested that in human ovarian cancer cells, CFP1 knock-out triggers cell cycle arrest and promotes apoptosis and senescence.

### In vivo validation of inhibition of ovarian cancer cell proliferation by CFP1 knock-out

**Fig. 4:**
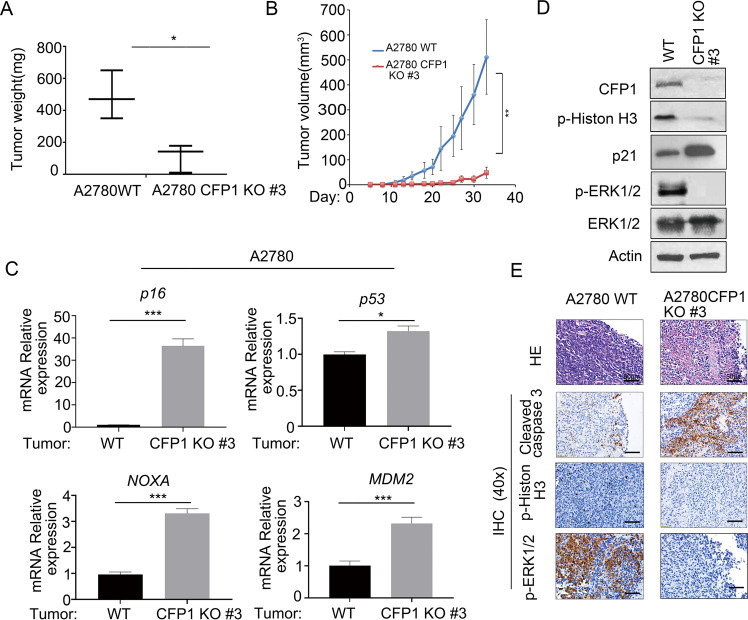
CFP1 knock-out inhibits tumor growth in vivo. **A**, **B** Deletion of CFP1 in A2780 cells inhibits tumor growth in vivo. A2780 WT and CFP1-deleted clone #3 were implanted subcutaneously into female BALB/c-nude mice (*n* = 6). Tumor weight was determined at the end of the experiments. ***P < 0.001, according to Student’s *t*-test. Tumor volume was monitored every other day. **P < 0.01, according to two-way ANOVA. **C** qRT-PCR detection of cell cycle-related factors in tumors. A2780 WT and CFP1-deleted clone #3 tumor samples were treated with TRIZOL reagent. RNA was extracted from tumor samples and subjected to qRT-PCR for cell cycle-related factors detection. The error bars represent SD. Student’s *t*-test was applied. ***P < 0.001. **D** Immunoblotting for protein expression in mouse tumor tissue. A2780 WT and CFP1-deleted clone #3 tumor samples were subjected to immunoblot analysis with CFP1, p-Histone H3, p21, p-ERK1/2, ERK1/2, and β-actin antibodies. **E** Loss of CFP1 increases apoptosis and decreases cell proliferation in tumors. A2780 WT and CFP1-deleted clone #3 tumor samples were subjected to immunohistochemical staining for cleaved caspase 3, p-ERK1/2, and p-Histone H3 in tumor tissues. Scale bar, 50 μm.

To further verify the effect of CFP1 knock-out on ovarian cancer cell proliferation in vivo, we injected A2780 WT cells (control cells) and A2780 CFP1-deleted cells #3 into nude mice and measured the tumor size regularly. CFP1 knock-out significantly inhibited tumor cell proliferation (Fig. 4A, B). qRT-PCR revealed that CFP1 knock-out increased the expression of *p16*, *p53*, and p53 downstream genes *NOXA* and *MDM2* in vivo (Fig. 4C). Immunoblotting revealed that proliferative protein p-Histone H3 was significantly reduced whereas cyclin-dependent kinase inhibitor 1A (CDKN1A) (p21) increased in CFP1-deleted tumor tissue cells (Fig. 4D). Immunohistochemical results indicated that the expression of proliferative proteins p-Histone H3 and p-ERK1/2 was significantly decreased, while the expression of apoptosis-related protein cleaved caspase 3 was increased in CFP1-deleted tumor tissue cells (Fig. 4E). These results indicated that CFP1 knock-out inhibited tumor cell proliferation in vivo.

### CFP1 affects the transcriptome of ovarian cancer cells by decreasing histone methylation

**Fig. 5:**
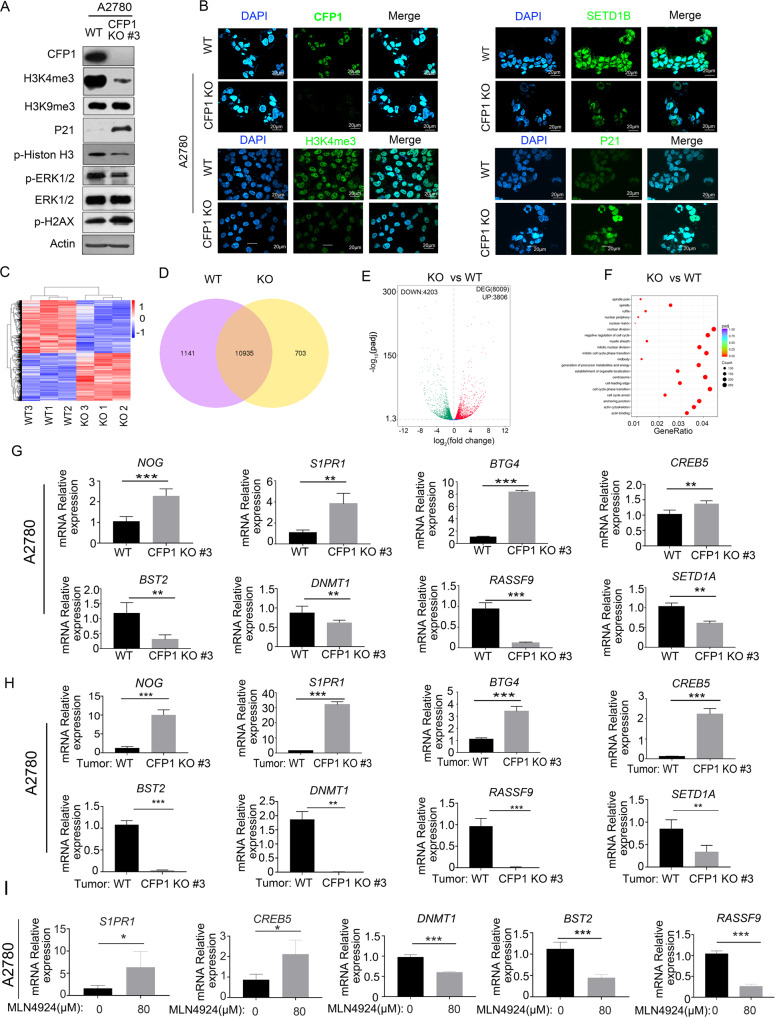
RNA-seq analysis of *CFP1 knock-out* transcriptional changes. **A** Immunoblotting for protein changes in CFP1-deleted cells. Cell lysates (A2780 WT and CFP1-deleted clone #3) were subjected to immunoblot analysis to show the reduction of CFP1, H3K4me3, p-Histone H3, and p-ERK1/2 proteins, and increase of p21 and p-H2AX protein. **B** Immunofluorescence assay for protein changes in CFP1-deleted cells. A2780 WT and CFP1-deleted clone #3 cells were subjected to immunostaining with the CFP1, P21, SETD1B, and H3K4me3 antibody (green) along with DAPI for DNA (blue). Scale bar, 20 μm. **C** Genome-wide RNA-seq analysis of A2780 WT and CFP1-deleted clone #3 cells. Differential gene expression hierarchical clustering heatmap. **D** Differential gene expression Venn diagram. A2780 CFP1 KO clone #3 vs. A2780 WT. **E** The top-up and downregulated genes between WT and CFP1-deleted cells are shown as a volcano plot. **F** GO enrichment scatter plot. A2780 CFP1 KO #3 vs. A2780 WT. **G** qRT-PCR for cellular gene expression in A2780 cells. Total RNA extracted from A2780 WT and CFP1-deleted clone #3 cells was subjected to qRT-PCR analysis for the indicated genes. Data are the mean ± SD from triplicated experiments. Student’s *t*-test was applied. **P < 0.01; ***P < 0.001. **H** qRT-PCR for tumor tissue gene expression in A2780 cells. Total RNA extracted from A2780 WT tumor and CFP1-deleted clone #3 tumor cells was subjected to qRT-PCR analysis for the indicated genes. Data are the mean ± SD from triplicated experiments. Student’s *t*-test was applied. **P < 0.01; ***P < 0.001. **I** qRT-PCR for gene expression after MLN4924 treatment in A2780 cells. Total RNA was extracted from MLN4924-treated A2780 cells and subjected to qRT-PCR analysis for the indicated genes.

It has been reported in the literature that CFP1 protein interacts with other components of the SET1 complex, binding SETD1 to CpG islands and thus causing H3K4me3 in these DNA regions [25]. As shown in Fig. 5A and Supplementary Fig. S3A, the expression of H3K4me3 was significantly reduced in CFP1-deleted ovarian cancer A2780 and ES-2 cells but H3K9me3 was not affected. In addition, CFP1 knock-out resulted in the accumulation of p21 and p-H2AX proteins in A2780 cells, and in a significant reduction of p-ERK1/2 and p-Histone H3 in A2780 and ES-2 cells (Fig. 5A and Supplementary Fig. S3A). The immunofluorescence assay revealed that H3K4me3 expression was significantly reduced, but p21 expression was increased in CFP1-deleted A2780 and ES-2 ovarian cancer cells (Fig. 5B and Supplementary Fig. S3C, D).

H3K4me3 is a near-universal chromatin modification at the transcription start site of active genes and has an instructive role in the transcription of genes. The histone methyltransferases SETD1 complex is driven to trimethylate H3K4 via its essential subunit CFP1, which engages in multivalent chromatin binding to recognize both nonmethylated DNA and existing H3K4me3. Our study revealed that CFP1 knockout resulted in the reduction of H3K4me3, which implies that CFP1 affects the transcriptome of ovarian cancer cells. We used RNA-sequencing (RNA-seq) for transcriptome analysis to further search for downstream target molecules and revealed that CFP1 knock-out affected signaling pathways, such as mitosis cell cycle phase transition and cell proliferation MAPK pathways (Fig. 5C–F and Supplementary Fig. S3F). Gene set enrichment analysis (GSEA) showed that histone-related genes were low expressed in CFP1 knockout A2780 cells (Supplementary Fig. S3G), while related genes specifically binding to promoters were high expressed (Supplementary Fig. S3H). While the expression of *NOG*, *S1PR1, BTG4*, and *CREB5* genes was significantly increased, that of *RASSF9*, *DNMT1*, *BST2*, and *SETD1A* genes was significantly decreased in CFP1-deleted A2780 and ES-2 ovarian cancer cells, based on qRT-PCR results (Fig. 5G and Supplementary Fig. S4A). Next, we extracted RNA from mouse tumor tissues and detected CFP1 downstream target genes in the tumor tissues of nude mice by qRT-PCR. As shown in Fig. 5H, the expression of *NOG* and *S1PR1* genes was also increased in CFP1-deleted tumor tissue cells, while the expression of *RASSF9* and *BST2* was decreased significantly. In addition, qRT-PCR results revealed the expression of *CDK4*, *FGF5*, *MEIS1*, and *TES* genes was significantly increased in CFP1-deleted ES-2 ovarian cancer cells (Supplementary Fig. S4B). The transcriptome change in CFP1 knock-out cells could be due to loss of CFP1 binding or the combinational effect of decreased H3K4me3 modification and loss of CFP1 binding. Next, we tested if the MLN4924 treatment could affect the genes downstream of CFP1. qRT- PCR results showed that *S1PR1* and *CREB5* expressions were increased, while those of *BST2*, *RASSF9*, and *DNMT1* were decreased after MLN4924 treatment (Fig. 5I). We also verified that the transcription levels of the histone methylation-related genes *EZH2*, *KDM8*, *STED5*, and *SMYD3* decreased significantly after MLN4924 treatment of ovarian cancer cells (Supplementary Fig. S4C). These results suggest that inhibiting CRL4 E3 ubiquitin ligase activity or deleting CFP1 affects the transcriptome of ovarian cancer cells through regulating histone methylation.

### CFP1 promotes ovarian cancer cell proliferation by affecting *BST2* transcription

**Fig. 6:**
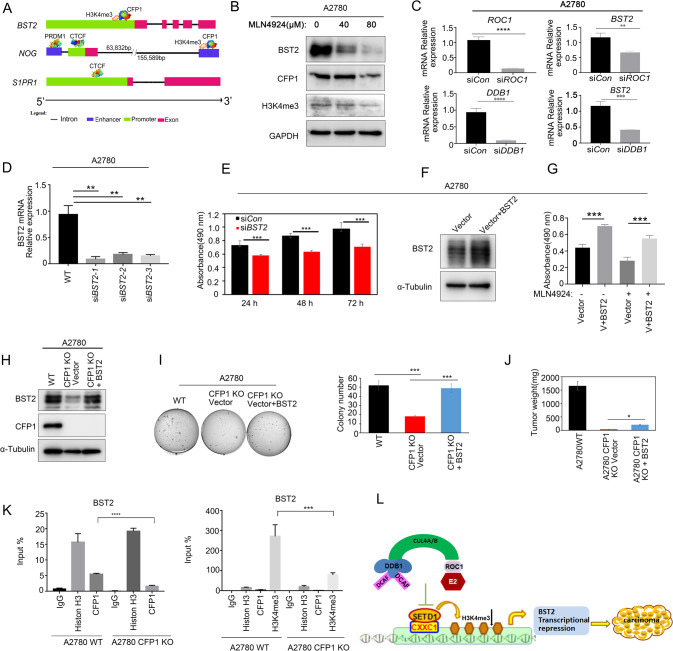
CFP1 promotes ovarian cancer cell proliferation by affecting *BST2* transcription. **A** Diagram showing the promoter regions of *BST2*, *NOG*, and *S1PR1* genes. **B** Western blot results for the changes in the levels of the indicated proteins in ovarian cancer cells (A2780) treated with increasing concentrations of MLN4924 for 24 h. **C** qRT- PCR results of BST2 mRNA levels after siRNA depletion of ROC1 in A2780 cells. **D** qRT-PCR results for BST2 siRNA depletion efficiency. Total RNA extracted from A2780 WT, CFP1 KO si*Con*, and CFP1 KO si*BST2* cells was subjected to qRT-PCR analysis for *Bst2*. Data are the mean ± SD of triplicated experiments. Student’s *t*-test was applied. **P < 0.01. **E** Effect of *BST2* depletion on A2780 cell growth as assessed by CCK8 assay. Data are the mean ± SD of triplicated experiments. Student’s *t*-test was applied. *P < 0.05; **P < 0.01; ***P < 0.001. **F** Immunoblotting for *BST2* overexpression (vector+BST2) efficiency on A2780 WT cells. **G** Effect of *BST2* overexpression (V+BST2) on the growth of A2780 cells treated with MLN4924 as assessed by CCK8 assay. Data are the mean ± SD of triplicated experiments. Student’s *t*-test was applied. ***P < 0.001. **H** Immunoblotting for *BST2* overexpression efficiency. Cell lysates (A2780 WT, CFP1 KO-vector, and A2780 CFP1 KO-BST2) were subjected to immunoblot analysis to verify the expression of BST2 protein. **I** Soft-agar assay for cell clone formation. The soft-agar assay was performed, and the colonies were stained with crystal violet for quantification, which is shown on the right panel. Data are the mean ± SD of three independent experiments. ***P < 0.001. **J** Mouse tumor-bearing model assay. A2780 WT, CFP1 KO-vector, and CFP1 KO-BST2 cells were implanted subcutaneously into nude mice. Two-way ANOVA test. *P < 0.05; ***P < 0.001. **K** CFP1 and H3K4me3 bind to the promoters of *BST2*. ChIP was performed with an antibody against CFP1 or H3K4me3 or with control IgG. The precipitated DNA was quantitated by qRT-PCR. *Gapdh* was included as the negative control. Data are the mean ± SD of quadruplicate experiments. **L** CRL4 E3 ubiquitin ligase affects histone methylation level in ovarian cancer by affecting CFP1 protein expression, thus regulating cell proliferation patterns.

The RNA-seq analysis results revealed that the transcription of several genes was increased in CFP1-deleted ovarian cancer cells. It was found that the CFP1 CXXC1 domain binds unmethylated DNA while the PHD domain located on its N-terminus binds H3K4me3 [26]. Previous studies showed S1PR1 and NOG can trigger various signaling pathways involved in carcinogenesis [27–29]. By predicting the relationship between *NOG* and *S1PR1* genes and *CFP1*, we found that CFP1 interacted with the enhancer position of the distal 3’ untranslated regions (UTRs) of NOG, but not with S1PR1. *NOG* and *S1PR1* have the same promoter, CTCF, while NOG has enhancer PRDM1 in the distal 5’ UTRs (Fig. 6A). To verify whether CFP1 protein affects ovarian cancer cell proliferation through *NOG* and *S1PR1* genes, we silenced both genes in CFP1-deleted cells by siRNA, and the expression of *NOG* and *S1PR1* genes was significantly decreased as revealed by qRT-PCR (Supplementary Fig. S4D). However, cell proliferation could not be rescued by silencing the *NOG* and *S1PR1* genes in CFP1-deleted cells (Supplementary Fig. S4E). To further verify whether CFP1 binds to the promoter regions of *NOG* and *S1PR1* genes, we conducted chromatin immunoprecipitation (ChIP) and found that CFP1 did not bind to the promoter regions of these genes (Supplementary Fig. S4F). These results indicated that CFP1 does not affect the proliferation of ovarian cancer cells through *NOG* and *S1PR1*, which is consistent with bioinformatics prediction.

We found that CFP1 knock-out significantly inhibited BST2 expression (Fig. 5G). The expression of BST2 also decreased significantly after MLN4924 treatment, as confirmed by western blot (Fig. 6B). Silencing of *ROC1* and *DDB1* by siRNA inhibited BST2 transcription, similar to CFP1 knock-out inhibiting BST2 expression (Fig. 6C). It has been indicated that decreased BST2 expression can inhibit tumor cell proliferation [30]. Therefore, we inhibited BST2 transcription by siRNA in ovarian cancer cells. The qRT-PCR results showed a significant decrease in BST2 expression (Fig. 6D). Reducing BST2 expression could significantly inhibit the proliferation of ovarian cancer cells (Fig. 6E). In addition, we also overexpressed BST2 in ES-2 and A2780 cells (Fig. 6F and Supplementary Fig. S4G). Cell counting kit 8 experimental results showed that BST2 overexpression could significantly promote the proliferation and cell death rescue of MLN4924-treated ovarian cancer cells (Fig. 6G and Supplementary Fig. S4H). These results suggested that CFP1 knock-out may affect ovarian cancer cell proliferation by inhibiting BST2 expression.

To test this hypothesis, we overexpressed BST2 in CFP-deleted cells, and the immunoblotting results showed that BST2 expression was significantly increased (Fig. 6H). BST2 elevation rescued the ability to form clones inhibited by CFP1 knock-out (Fig. 6I). In addition, we verified in vivo that BST2 overexpression could promote the tumor-forming ability of CFP1-deleted ovarian cancer cells using nude mouse tumor-forming experiments (Fig. 6J). By predicting the relationship between *BST2* and *CFP1* genes, we found that CFP1 interacted with the promoter position of BST2 (Fig. 6A). To further verify if CFP1 could affect *BST2* transcription levels, we performed ChIP and verified that both CFP1 and H3K4me3 could bind to the *BST2* promoter region, and thus regulate gene transcription (Fig. 6K). In summary, the CRL4 E3 ubiquitin ligase complex could inhibit ovarian cancer cell proliferation by affecting CFP1 protein expression, while both CFP1 and SETD1 affected *BST2* transcription to regulate ovarian cancer cell proliferation, apoptosis, and clone formation (Fig. 6L).

## Discussion

CFP1 is an important subunit in the SET1 histone methylation complex that recognizes and binds to CpG islands present in a nonmethylated state in the genome [31, 32]. The SET1 complex mediates H3K4me3, and such methylation leads to looser chromatin structure and easier gene transcription [33]. As a result, the CFP1 protein interacts with SETD1A/B and other components of the SET1 complex, and brings SETD1 to CpG islands, resulting in more H3K4me3 in these DNA regions [34–36]. Mouse embryos lacking *CFP1* died before gastrulation, whereas embryonic stem (ES) cells lacking *CFP1* were viable, but failed to differentiate [37, 38]. Mouse ES cells lacking *CFP1* exhibited a loss of H3K4me3 in many CpG islands, and mislocalized this epigenetic mark to the heterochromatic subnuclear domain [39]. Furthermore, these cells failed to differentiate in vitro. These defects were repaired after the introduction of a plasmid expressing *CFP1* [40].

Fan et al. found that *CFP1*-deleted female mice were completely sterile, CFP1 regulates downstream gene transcription in oocytes, and that knockout of *CFP1* caused failure to accumulate maternal mRNA, resulting in failure to maintain oocytes as well as the failure of oocyte development [41]. These results indicated that both DNA hypermethylation due to lack of CRL4 E3 activity and decreased mRNA transcription due to CFP1 knock-out severely impeded the ability of oocytes to maintain and reprogram, resulting in premature ovarian cancer and female infertility [42]. In addition, CFP1 knock-out led to delayed meiotic response and arrest in the metaphase of the first meiosis via H3K4 methylation effects [43]. In the present study, in vitro and in vivo experiments revealed that inhibition of CRL4 E3 ubiquitin ligase complex activity affected CFP1 protein expression. MLN4924 treatment decreased the expression of CFP1 and BST2 proteins by affecting the CRL4 E3 ubiquitin ligase complex. Silencing of the CRL4 E3 ubiquitin ligase complex *ROC1* and *CUL4A* genes by siRNA significantly decreased the expression of CFP1 and BST2, consistent with that observed after MLN4924 treatment of ovarian cancer cells. However, whether CRL4 E3 ubiquitin ligase complex degraded CFP1 protein through ubiquitination or interacted with CFP1 protein through DDB1–CUL4-associated factors was not explained in the present study. In addition, the RNA-seq results showed that after the knockout of *CFP1*, cell cycle-related signaling pathways, negative cell cycle regulatory pathways, and mitotic nucleus division signaling pathways changed significantly. We found that CFP1 knock-out affected H3K4me3 protein and H3/ERRK1/3 phosphorylation. Although the direct causal relationship between histone methylation and CFP1 downstream genes’ transcription is missing, as suggested in Fig. 6K, CFP1 could directly bind to gene promotor, so there is a possibility that the transcriptome change in CFP1 knock-out cells could be due to loss of CFP1 binding or the combinational effect of CFP1 and H3K4me3 and CFP1 binding.

In the present study, we knocked out CFP1 in ovarian cancer cells by the CRISPR/Cas9 method and detected that CFP1 partial deletion inhibited ovarian cancer cell proliferation both in vitro and in vivo, and promoted apoptosis and senescence. RNA-seq analysis of the CFP1-deleted ovarian cancer cells transcriptome showed that 703 genes were altered (Fig. 5D), and gene ontology analysis showed that many of these genes were associated with cell mitosis and cell cycle regulation, such as *CREB5* and *BST2* (Fig. 5F) [44]. This suggests that CFP1 knock-out affects the transcriptome of ovarian cancer cells. In addition, SETD1A and H3K4me3 were significantly reduced as revealed by qRT-PCR and immunoblotting, and these results suggest that CFP1 and the SET1 complex can affect H3K4me3 of engineered ovarian cell genes. Moreover, CFP1 affects gene transcriptional activity by binding to the *BST2* promoter region to regulate the proliferation, apoptosis, and other biological activities of ovarian cancer cells (Fig. 6). Overall, the present study is the first to verify that CRL4 E3 ubiquitin ligase complex can regulate CFP1 protein expression in ovarian cancer cells, and to reveal the molecular mechanism underlying the CFP1 regulation of ovarian cancer cell proliferation by binding to the *BST2* promoter region, thereby influencing the transcription level of *BST2*. This mechanism lays the theoretical foundation for identifying molecular targets of ovarian cancer clinical treatment and further demonstrates the importance of CRL4 E3 ubiquitin ligase complex as a therapeutic target for ovarian cancer.

## Materials and methods

### Cell lines and culturing

Human ovarian cancer cell lines A2780, ES-2, OVCAR-3, HO8910, CAOV3, and SKOV3 were purchased from ATCC (Manassas, VA, USA) and IOSE cells were supplied by Heng-Yu Fan, Zhejiang University. IOSE, A2780, HO8910, and CAOV3 cells were cultured in fully supplemented high-glucose Dulbecco’s modified Eagle medium (DMEM; Gibco; Thermo Fisher Scientific, Waltham, MA, USA) containing 10% fetal bovine serum (FBS; Gibco; Thermo Fisher Scientific), 100 U/mL penicillin, 100 μg/mL streptomycin (Pen-Strep, Gibco; Thermo Fisher Scientific), and phenol red. OVCAR-3 cells were cultured in Roswell Park Memorial Institute 1640 (RPMI-1640, Gibco; Thermo Fisher), whereas ES-2 and SKOV3 cells were cultured in McCOY’s 5A medium (Modified), no serum (Gibco; Thermo Fisher Scientific). Cells were cultured under standard conditions (37 °C, humidified atmosphere composed of 95% air and 5% CO_2_). The passage ratio for all cell lines was 1:5. Cells were tested for mycoplasma infection by PCR (TakaRa Bio, Beijing, China) before use in experiments.

### *CFP1* gene editing

CFP1-deleted cells were created using CRISPR/Cas9 [45]. Transient CRISPR plasmids pX459-Cfp1-1 and pX459-Cfp1-2 (pX459; plasmid #48139, Addgene, Watertown, MA, USA) were prepared for CFP1 knock-out to avoid unspecific effects mediated by Cas9/single-guide RNA (sgRNA). Human *Cfp1* sgRNA sequences (5’-AGCGGGACAGCAGTGAGCCC-3’ and 5’-GAGGACAGCAAGTCCGAGAA-3’) were designed using http://crispr.mit.edu. To construct the plasmid, pX459 was digested with *Bbsl* for 30 min at 37 °C. Each sgRNA primer was phosphorylated and annealed using an annealing agent for DNA oligos (Cat: D0251, Beyotime, Shanghai, China). The *Bbsl*-digested pX459 plasmid was then ligated with the phosphorylated and annealed oligo duplex, and the recombinant plasmid was transformed and verified.

A2780 and ES-2 cells were transfected with Cas9 and sgRNA using lipofectamine3000 reagent (Cat: L3000150, Thermo Fisher Scientific, Waltham, MA, USA) according to the manufacturer’s instructions. Forty-eight hours after transfection, cells’ medium was supplemented with 1–2 μg/mL puromycin (Gibco; Thermo Fisher Scientific) to select cells, and then changed to a fresh puromycin-containing medium every 2 days. After 7 days, cell samples were collected for western blot to select the best primer. Cells were then subject to fluorescence-activated cell sorting (FACSAria III, BD Biosciences, Franklin Lakes, NJ, USA) into 96-well plates with a single cell per well. The clones were screened by western blot as described below using antibodies against CFP1 (Supplementary Table S1). Two independent clones were analyzed, and the parental CFP1 WT cells not transfected with pX459 were used as controls.

### Retroviral transfection of *BST2*

A BST2 overexpression vector was constructed using a pQCXIH retroviral vector (Addgene, plasmid #17394) containing the full-length cDNA sequence of BST2. A total of 293 Phoenix retrovirus packaging cells were transfected with empty pQCXIH or pQCXIH-BST2, Retro VSVG, and Retro GPE constructs using PolyJet^TM^ DNA in vitro Transfection Reagent (Signagen Laboratories, Frederick, MD, USA) according to the manufacturer’s instructions. Forty-eight hours after transfection, the retroviral supernatant was supplemented with 5 μg/mL polybrene and filtered through a sterile 0.45-μm filter (Gibco; Thermo Fisher Scientific) and used to infect the target A2780 CFP1-deleted cells. Forty-eight hours after infection, cells were selected with 200 μg/mL hygromycin in a fully supplemented culture medium.

### Colony-formation assay

Six-well plates were coated with 1.5 mL of 0.5% bottom agar (Sigma-Aldrich, St. Louis, MO, USA) in a 35-mm diameter culture dish (Corning, Corning, NY, USA). Cells were suspended in 1 mL of 0.35% agar containing 1× cell culture medium and 10% FBS and poured over the culture plates. The final cell concentration in each culture was 0.25 × 10^3^ cells/mL. Triplicate cultures were used for each experiment. Colonies were counted 3 weeks after plating using an Omnicon FAS II (BD Biosciences, Franklin Lakes, NJ, USA) image analysis system.

### Wound-healing assay

ES-2 WT and CFP1-deleted cells were grown to confluence in DMEM supplemented with 10% FBS in six-well plates. Then, the medium was changed to FBS-free DMEM, and the cell monolayers were scraped in a straight line using a P-10 pipette tip to create a “scratch wound”. The plates were photographed at 0 and 24 h using a phase-contrast inverted microscope (Nikon Ti, Nikon Corp., Tokyo, Japan).

### Transwell migration assay

Twenty-four-well tissue culture plate inserts with 8-μm pore filters and BioCoat Matrigel (BD Biosciences, San Diego, CA, USA) were used to assess the migration and invasive potential of ES-2 and A2780 WT and *CFP1*-deleted cells. Cells were suspended in a serum-free medium, and then added to a transwell (100 μL cell suspension/well at a concentration of 0.5–1 × 10^5^ cells/mL). After incubation for 24 h at 37 °C, cells at the upper surface of the transwell were removed with cotton swabs. Cells that migrated were attached to the lower surface and stained with hematoxylin and eosin. Transwells were rinsed with water and air-dried. Positive cells were quantified using Image-Pro Plus 6.0 software (Media Cybernetics Inc., Rockwell, MD, USA).

### Mode of cell death

Apoptosis was detected using the FITC-annexin V apoptosis detection kit (BD Biosciences), according to the manufacturer’s instructions. A2780 and ES-2 WT and CFP1-deleted cells (2.5 × 10^6^) were cultured in six-well plates using DMEM. After 18 h, the culture medium was replaced with a new one. Cells were collected into 15-mL tubes after 24 h, washed twice with ice-cold phosphate-buffered saline (PBS), and then resuspended in 1× binding buffer at a concentration of 1 × 10^6^ cells/mL. Subsequently, 1 × 10^5^ cells were transferred to a 5-mL culture tube, and 5 µL of PE-conjugated annexin V and 7-AAD were added to 15 mL detecting tubes. Cells were gently mixed and incubated for 15 min at room temperature in the dark before 400 µL of 1× binding buffer was added to each tube for flow cytometry analysis within 1 h. Results were analyzed using FlowJo software (BD Biosciences).

### Western blotting

Total protein was isolated from the cell extracts, mouse tumor cells, and ovarian tissue, and 30 μg of protein was separated by 8% or 12% sodium dodecyl sulfate-polyacrylamide gel electrophoresis at 120 V for 1 h. Proteins were transferred onto polyvinylidene difluoride membranes (Millipore, Bedford, MA, USA) that were blocked with 5% (w/v) milk for 1 h at 18 °C. After probing with primary antibodies, membranes were washed in tris-buffered saline that contained 0.05% (w/v) Tween-20 (TBST) and incubated with an anti-rabbit IgG, horseradish peroxidase-linked secondary antibody (cat# 7074, Cell Signaling Technology, Danvers, MA, USA). Finally, the obtained bands were detected using an Enhanced Chemiluminescence Detection Kit (Millipore). Results were collected using Imager 680 (Amersham; GE Healthcare, Chicago, IL, USA). The list of primary antibodies is provided in Supplementary Table S1 (see Supplementary experimental procedures).

### RNA extraction and qRT-PCR

Total RNA was extracted using the RNeasy Plus Mini Kit (Qiagen, Hilden, Germany) according to the manufacturer’s instructions. The qRT-PCR was performed using an SYBR FAST qPCR kit (Kapa Biosystems, Wilmington, MA, USA) on an Applied 7300 real-time PCR system (ABI Biosystems; Thermo Fisher Scientific, Foster City, CA, USA). Relative mRNA levels were determined by normalizing the obtained expression levels to the endogenous GAPDH mRNA levels. The relative transcript levels of the control sample were set to 1, and the transcript levels of other samples were normalized to that of the control. qRT-PCR reactions were performed in triplicate using the primers presented in Supplementary Table S2 (see Supplementary experimental procedures).

### Animals and xenograft models

Animal experiments were approved by the animal ethics committee of Jiaxing University. Complying with animal ethical guidelines, strain, age- and sex-matched mice were chosen for the study based on preliminary data. Usually, *n* = 5–12 mice were used for each group. BALB/c-nude mice (female, 5–6 weeks of age) were purchased from Zhejiang Province Experimental Animal Center (Hangzhou, China). BALB/c-nude mice were housed in individually ventilated cages under standard conditions, under a 14/10 h light/dark schedule, and were provided sterilized food and water ad libitum. All animal experiments were performed in accordance with the NIH Guide for the Care and Use of Laboratory Animals, and animals were handled as directed by the animal ethics and welfare committee of Jiaxing University.

A2780 WT and ES-2 WT cells (1 × 10^6^) were trypsinized, and resuspended in 100 µL of PBS, followed by subcutaneous injection in the right dorsal flank of nude mice (*n* = 12/group). Animals were randomly divided into a control group and an MLN4924 treatment group (*n* = 6/group). Pevonedistat (MLN4924) was dissolved in sterile 10% DMSO containing PBS and stored at −20 °C until use. After the tumor had reached approximately 150–200 mm^3^ (cubic mm) in volume [43, 46], mice were injected intraperitoneally with 0.1 mL of MLN4924 (2 mg/kg) or PBS once per day, 5 consecutive days per week, for 4 weeks. The investigators who are blind to allocation will then measure the weight of the mice and the size of the tumor volume. The animals were weighed once per week, and the tumor volume was measured with a caliper every other day. The tumor volume was determined by (width)^2^ × height × 0.523. The mice were sacrificed after 28 days. Tumors were excised and loafed, fixed in 4% paraformaldehyde, dehydrated in a graded ethanol and xylene series, and embedded in paraffin.

To assess cancer cell proliferation in vivo, A2780 WT, A2780 CFP1-deleted #3, A2780 CFP1 KO #3-Vector, and A2780 CFP1 KO #3-BST2 overexpression cells (5 × 10^5^) were subcutaneously transplanted into both dorsal flanks of 6-week-old female BALB/c-nude mice (*n* = 6/group). Tumor volume was measured every 2–3 days. The animals were sacrificed using CO_2_ when a human endpoint was reached, which included the tumor growing to 15 mm in diameter or when signs of ulceration were evident. Following sacrifice, tumors were loafed and collected and processed as described above.

### Histochemistry and immunohistochemistry of healthy human ovary tissue and tumor tissue

Paraffin-embedded tissue from ovarian tumors and normal ovaries were provided by the Affiliated Hospital of Jiaxing University. Human ovarian cancer TMAs were purchased from Fanpu Biochec, Inc (GuiLin, China). Each cancer tissue specimen from each patient was represented by two cores on each TMA slide, which was 1.5 mm in diameter. Clinical information was provided by Fanpu Biochec, Inc (Supplementary Table S5). The use of archived cancer samples in this study was approved by the Jiaxing University Institutional Review Board (LS2021-KY-337).

Mouse primary tumor tissues were embedded and cut into 5-μm sections (RM2235 microtome, Leica, Wetzlar, Germany), deparaffinized, and stained with hematoxylin and eosin. For immunochemistry, deparaffinized sections were incubated in 0.3% H_2_O_2_ for 10 min_._ After antigen retrieval with 10 mM sodium citrate (pH 6.0) for 15 min, sections were incubated with CFP1, p-ERK1/2, p-Histone H3, and cleaved caspase 3 primary antibodies (Supplementary Table S1, 1:200) at room temperature 18 °C for 1 h, washed three times with 1× PBS, and incubated with biotin-labeled secondary antibodies (1:400; Cell Signaling Technology, Danvers, MA, USA) for 30 min. Sections were counterstained using a Vectastain ABC kit and a 3,3′-diaminobenzidine peroxidase substrate kit (Vector Laboratories, Burlingame, CA, USA).

### Immunofluorescence staining and imaging of cultured cells

Cells (1 × 10^5^) were seeded in six-well plates overnight, washed thrice with PBS, and then fixed for 10 min at room temperature 18 °C with 4% paraformaldehyde in PBS. Cells were permeabilized with 0.3% (w/v) Triton X-100 in PBS, incubated with blocking buffer (PBST containing 5% bovine serum albumin), and sequentially probed with anti-CFP1, anti-SETD1B, anti-H3K4me3, anti-Ki-67, anti-p21, and anti-cleaved caspase 3 primary antibodies (Supplementary Table S1, 1:200) and Alexa488-conjugated secondary antibodies (Cell Signaling Technology). Slides were mounted using VectaShield containing 4′,6-diamidino-2-phenylindole (Vector Laboratories). Digital images were acquired with a confocal laser scanning microscope FV3000 (Omnicon,) at ×6–100 magnification.

### Bioinformatics analysis

The promoter binding regions of *BST2*, *NOG*, and *S1PR1* genes and their interacting proteins were predicted by using a human gene database (www.genecards.org/genecarna). The relationship between these genes and *Cfp1* was analyzed using a gene structure display system (gsds.gao-lab.org) to present the results. The gene enrichment of histone methylation-related pathways was analyzed by GSEA (http://www.gsea-msigdb.org/gsea).

### ChIP analysis

A2780 WT and CFP1-deleted cells were fixed in 1% formaldehyde for 10 min and quenched with 0.125 M glycine for 5 min at room temperature 18 °C. Harvested cells were washed with ice-cold PBS and then lysed in ice-cold PBS containing protease inhibitor. The mixture was centrifuged at 2000 × *g* for 5 min at 4 °C to pellet the nuclei, and the chromatin DNAs were then digested with micrococcal nuclease (MNase) in ChIP MNase buffer. Immunoprecipitation reactions were carried out with chromatin extracts overnight at 4 °C using 5 μg of antibodies against CFP1 and H3K4me3. Histone H3 was used as the positive control and rabbit IgG (Cell Signaling Technology) was used as the negative control. Two percent of the chromatin extract was set aside for input. Antibody-protein-DNA complexes were then bound to 30 μL of ChIP-grade protein G magnetic beads (Cell Signaling Technology) for 3 h at 4 °C. The beads were washed twice with low-salt wash buffer, twice with high-salt wash buffer, and once with TE buffer. Beads were resuspended in elution buffer and incubated for 30 min at 65 °C with frequent mixing. The resulting eluate and input samples were transferred into new tubes, and reverse cross-linking reactions were carried out overnight at 65 °C. Samples were then treated with RNase A (0.2 mg/mL) for 30 min at 37 °C, followed by proteinase K (0.2 mg/mL) digestion for 4 h at 65 °C. The obtained DNA was ethanol-precipitated and purified using the QiaPrep DNA purification columns (Qiagen). Precipitated DNA was quantitated by qRT-PCR. For detailed steps, please refer to SimpleChIP Enzymatic Chromatin IP Kit (cat # 9003, Cell Signaling Technology). The primers are detailed in Supplementary Table S3 (see Supplementary experimental procedures).

### RNA sequencing

A2780 WT and CFP1-deleted cells were collected, and total RNA was extracted with TRIZOL reagent (Invitrogen) followed by purification using the RNeasy kit (Qiagen). RNA degradation and contamination were assayed by 1% agarose gel electrophoresis. A total amount of 3 µg of RNA per sample was used as input material for constructing RNA libraries for transcriptome sequencing and clustering, which were outsourced to Novogene (Beijing, China). The resulting *p* values were adjusted using the Benjamini and Hochberg method. A corrected *p* value of 0.05 and absolute fold change of 2 were set as the threshold for significantly different expressions.

### RNA interference

A2780 WT cells were transfected with siRNAs using siRNA-Mate Reagent (GenePharma, Shanghai, China). Briefly, cells were seeded in six-well plates and transfected with siRNA and siRNA-Mate Reagent, each incubated separately in Opti-minim essential medium for 5 min, mixed together for 10 min at room temperature 18 °C, and then the mixture was applied to the cells (1 mL/well, final siRNA concentration of 80 nM). siRNA sequences are presented in Supplementary Table S4 (see Supplementary experimental procedures).

### Statistical analyses

Usually, no data were excluded, except in the in vivo mouse experiments when 1–2 mice were dropped out for further analysis due to accidental death. Data are presented as mean ± standard deviation. All in vitro assays were performed in triplicate. Groups were compared by Mann–Whitney *U* test, Kruskal–Wallis test, or analysis of variance (ANOVA) using GraphPad Prism software (GraphPad Prism, San Diego, CA, USA). In any case, samples with *n* < 8 were analyzed with nonparametric tests. The normal distribution of the data was tested with ANOVA. The variance is similar between the groups that are being statistically compared. *P* value of ≤0.05 was considered significant.

## Supplementary information

Supplementary materials

Supplementary figure 1

Supplementary figure 2

Supplementary figure 3

Supplementary figure 4

Table S5

Dataset 1

Dataset 2

## Data Availability

All data needed to evaluate the conclusions in the paper are present in the paper and/or the Supplementary materials.
