# Supplementary material for: CpG-binding protein CFP1 promotes ovarian cancer cell proliferation by regulating BST2 transcription
Source: Cancer Gene Ther. 2022 Jul 21;29(12):1895–907. doi: 10.1038/s41417-022-00503-z (PMC9750859; doi:10.1038/s41417-022-00503-z)
Supplement: Supplementary file 1 — Supplementary materials [file 41417_2022_503_MOESM1_ESM.docx]

**SUPPLEMENTAL MATERIAL**

**CpG-binding protein CFP1 promotes ovarian cancer cell proliferation by regulating *BST2* gene transcription**

Liu-Qing Yang^1#^, Han-Yin Hu^1#^, Yao Han^1^, Ze-Yi Tang^1^, Jie Gao^1^, Qi-Yin Zhou^1^, Yi-Xuan Liu^1^, Hao-Sa Chen^1^, Tu-Nan Xu^1^, Lei Ao^1^, Ying Xu^1^, Xuan Che^2^, Ya-Bo Jiang^3^, Chun-Wei Xu^4^, Xian-Chao Zhang^7^, Yu-Xin Jiang^1^, Michal Heger^1,5,6^, Xiao-Min Wang^1^*, Shu-Qun Cheng^3^*, Wei-Wei Pan^1,8^*

*^1^ Department of Cell biology, College of Medicine, Jiaxing University, 118 Jiahang Road, Jiaxing 314001, China*

*^2^ Department of Anethesiology, Jiaxing Maternity and Child Health Care Hospital, Affiliated Women and Children Hospital, Jiaxing University, Zhejiang Province, China. Jiaxing 314001, China*

*^3^ Department of Hepatic Surgery VI, Eastern Hepatobiliary Surgery Hospital, Second Military Medical University, 225 Changhai Road, Shanghai 200438, China*

*^4^ Department of Pathology, Fujian Cancer Hospital, Fujian Medical University Cancer Hospital, 350014, Fuzhou, Fujian, China*

*^5^ Department of Pharmaceutics, Utrecht Institute for Pharmaceutical Sciences, Utrecht University, Utrecht, the Netherlands*

*^6^ Laboratory of Experimental Oncology, Department of Pathology, Erasmus MC, Rotterdam, the Netherlands*

*^7^* *Institute of Information Network and Artificial Intelligence, Jiaxing University, 118 Jiahang Road, Jiaxing 314001, China*

*^8^G60 STI Valley Industry & Innovation Institute, Jiaxing University, 118 Jiahang Road, Jiaxing 314001, China*

**Figures S1 to S3**

**Supplemental figure legends**

**Supplemental experimental procedures**

**SUPPLEMENTAL FIGURE LEGENDS**

**Supplemental figure 1.** Targeting the CRL4 ubiquitin ligase to trigger multiple cells killing pathways

CRL4 ubiquitin ligase complex can be indicated by targeting its oncogenic components, including RBX1, Cul4A, Cul4B, or DDB1 via siRNA silencing approach or by pharmaceutical inhibition of cullin neddylation with a NAE inhibitor, MLN4924. Inactivation of CRL4 ubiquitin ligase causes the accumulation of its substrates which suppress cancer cell growth by triggering multiple cancer killing pathways, including apoptosis, senescence, autophagy and DNA damage with the mechanisms subjected to future investigation.

**Supplemental figure2. CFP1 deletion inhibited cell cycle.**

**A** The statistical result of CFP1 protein expression in human ovarian cancer and normal ovary tissues. **B** Quantitative RT-PCR analysis of *CFP1* expression in the mouse tissue. **C** MLN4924 inhibited tumor growth in mice. 1 × 10^6^ ES-2 cells was injected into the flank of nude mice. After tumor growth had reached roughly 200 mm^3^, the mice were randomly assigned to the MLN4924-treated group or control group. The single injection dose was 2 mg/kg. ** *P* < 0.01, *** *P* < 0.001. The P-value of tumor volume was determined using two-way ANOVA. **D** Immunoblotting revealing that MLN4924 inhibited CFP1 expression *in vivo*. ES-2 tumor samples were harvested and then subjected to immunoblot analysis with the indicated antibodies. **E** Immunoblotting revealing that MLN4924 inhibited CFP1 expression in ovarian cancer (ES-2) cells. Cells were subject to control or MLN4924 treatment and the CFP1 protein was subjected to immunoblot analysis with the indicated antibodies. **F** Immunoblotting results for ROC1 siRNA depletion efficiency and CFP1 expression in ES-2 cells. **G** Cell cycle arrest after thymidine-nocodazole block. A2780 WT and CFP1 deletion #3 cells were treated with thymidine-nocodazole. Propidium iodine staining detected the cell cycle stage by FACS. **P* < 0.05. ** *P* < 0.01.**H** CFP1 levels in cell cycle-synchronized A2780 cells. A2780 WT cells were arrested at metaphase by nocodazole(1μM). Samples were taken at the indicated time points after being released from nocodazole arrest. **I** qRT- PCR detection of cell cycle-related factors in ES-2 cells. ES-2 WT and CFP1 deletion #1cells were used. The error bars represent s.d. Student’s t-test was applied.

**Supplemental figure 3. CFP1 deletion inhibition ovarian cancer cell proliferation**

**A** Immunoblotting for protein changes in CFP1 deletion cells. Cell lysates (ES-2 WT and clone #1) were subjected to immunoblot analysis to show the reduction of CFP1, H3K4me3, p-Histon H3 and p-ERK1/2 proteins. **B-E** Immunofluorescence detection of cell proliferation and apoptosis in ES-2 cells. ES-2 WT and CFP1 deletion #1 cells were subjected to immunostaining with the KI-67, H3K4me3, P21 and SETD1B antibody (green) along with DAPI for DNA (blue). Scale bar, 20 μm. **F** Top 20 enriched KEGG analysis. A2780 CFP1 knock-out clone #3 (KO #3) vs A2780 WT. Fold > 1.5, *P* < 0.05. **G** GSEA analysis of transcriptome.

**Supplemental figure 4. *CFP1 deletion*** **inducing gene transcriptional changes.**

**A-B** Quantitative PCR detection of gene transcriptional changes in ES-2 cells. ES-2 WT and CFP1 deletion #1 cells were used. The error bars represent s.d. Student’s t-test was applied. ns (*P* >0.05), ** *P* < 0.01, *** *P* < 0.001. **C** Quantitative PCR detection of histone methylation relative gene after MLN4924 treatment. **D** Quantitative PCR assay to detect the interference efficiency of *NOG* and *S1pr1.* Total RNA extracted from A2780 WT, CFP1 KO si*Con* and CFP1 KO si*Nog* was subjected to RT-PCR analysis for *Nog* genes. Data are mean±SD from triplicated experiments. Student’s t-test was applied. *** denotes *P*< 0.001. **E** Cell proliferation assay to detect cell proliferation after *CFP1* deletion cells interfered with *NOG* and *S1PR1* in A2780 cells*.* **F** Chromatin immunoprecipitation assay to detect the binding of CFP1 to the promoter regions of *NOG* and *S1pr1*. ChIP with an antibody against CFP1 or H3K4me3 or control IgG was performed. The precipitated DNA was quantitated by real-time PCR. *Gapdh* was included as a negative control. Data are means ±SD of quadruplicates. **G** Immunoblotting for *BST2* overexpression (vector+BST2) efficiency on ES-2 WT cells. **H** Effect of *BST2* overexpression (V+BST2) on the growth of ES-2 cells treated with MLN4924 as assessed by CCK8 assay. Data are the mean ± SD of triplicated experiments. Student’s *t*-test was applied. ***, *P* < 0.001.

**Table S1**. Summary of antibodies used.

| **Antigen** | **Source** | **Catalogue #** |
| --- | --- | --- |
| CFP1 | Abcam | ab198977 |
| H3K4me3 | Abcam | ab8580 |
| H3K9me3 | Abcam | ab8898 |
| BST2 | Ptoteintec | 13560-1-AP |
| P21 | Cell Signaling | 12D1 |
| ERK1/2 | Cell Signaling | 4695 |
| p-ERK1/2 | Cell Signaling | 4370 |
| Actin | Abcam | ab3280 |
| AKT | Cell Signaling | 9272 |
| p-AKT | Cell Signaling | 4058 |
| PARP | Cell Signaling | 9532 |
| cleaved caspase-3 | Cell Signaling | 9664 |
| KI-67 | Cell Signaling | 9129 |
| p-Histone H3 | Cell Signaling | 9701 |

**RNA extraction and real-time RT–PCR analysis**

Table S2. The primers sequences (Sangon Biotech, China) used in this study.

| Name | Sequence |
| --- | --- |

*Actin:* 5'-GCTCTTTTCCAGCCTTCCTT-3'(forward);

5'-GTACTTGCGCTCAGGAGGAG-3'(reverse);

*S1pr1*: 5'-CTGCCAACAGGTCTGAGAGG-3'(forward);

5'-ATATCAGCGCGGACAAGGAG-3'(reverse);

*Nog*: 5'-GATCTGAACGAGACGCTGCT-3'(forward);

5'-CCTTTGATCTCGCTCGGCAT-3'(reverse);

*Nt5e*: 5'-TCGGCTCTTCACCAAGGTTC-3'(forward);

5'-CCTCTTTGAGGAGTGGCTCG-3'(reverse);

*Creb5*: 5'-CAGGAGGGAATTCAGCCTCA-3'(forward);

5'-AGCAGGGTGGTGAGTCAATG-3'(reverse);

*Rassf9*: 5'-AGAAGCTTGTCTGTGGGCTG-3'(forward);

5'-CATCTCCCCACGCTTTCCAA-3'(reverse);

*Bst2*: 5'-ATGGAAGACGGGGATAAGCG-3'(forward);

5'-AGGAGATGGGTGACATTGCG-3'(reverse);

*Setd1a*: 5'-TCACCGTACTTACGGCACAT-3'(forward);

5'-CGTTGCCATGTCAGGTCCAA-3'(reverse);

*P16*: 5'-CTGCCCAACGCACCGAATAG-3'(forward);

5'-ACCACCAGCGTGTCCAGGAA-3'(reverse);

*Noxa*: 5'-GTTGATGGAAATGCCTGGTATT-3'(forward);

5'-CCTAGCTCCACGACTCTTCAAA-3'(reverse);

*P27*: 5′-CAAACTCTGAGGACCGGCAT′-3(forward);

5′-CTTCCTCATCCCTGGACACTG-3′(reverse).

**Chromatin immunoprecipitation (ChIP)**

Table S3. The primers sequences (Sangon Biotech, China) used in this study.

| Name | Sequence |
| --- | --- |

BST2-CL1: 5'-TAAGTCAGGCAAAGGTCGTGTCTTT-3'

BST2-CR1: 5'-GAGTGGGATCCCATCTCTTAAACCA-3'

NOG-CL1: 5' –CGCTTTCAGCGGAAACTGCCCAC- 3'

NOG-CR1: 5' –CCCATTGTGACAACCTAAATCGCT- 3'

S1PR1-CL1: 5' –TTGGAACCCAGCTCTGCCACTCA- 3'

S1PR1-CR1: 5' –GCATTCTTTAAAGCCAAAGGAATC- 3'

**RNA interference**

Tabel S4. The siRNA sequences (GenePharma, Shanghai, China) used in this experiment.

| Name | Sequence |
| --- | --- |

siCON(siNC): UUCUCCGAACGUGUCACGUTT

BST2 siRNA-1: GCUCCUGAUCAUCGUGAUUTT

BST2 siRNA-2: CCUGCAACCACACUGUGAUTT

BST2 siRNA-3: GGAGAGAUCACUACAUUAATT

ROC1 siRNA: GACUUUCCCUGCUGUUACCUAATT

CUL4A siRNA: GAAGCUGGUCAUCAAGAAC

DDB1 siRNA: GGCCAAGAACAUCAGUGUG

**Thymidine-Nocodazole block cell cycle**

A2780 WT and CFP1-deficient cells #3 (1.5 × 10^6^) were cultured in 6-wells plates using DMEM culture medium overnight. Cells were replaced with DMEM including 2mM Thymidine for 24 h. Cells were change with fresh DMEM (10% FBS, 1% Pen-Strep) for 3 h to release cells. After releasing of the cells, we add 100ng/ml Nocodazole to the DMEM media for 12 h. Then cells were washed with 1×PBS and added fresh DMEM including 10% FBS, 1% Pen-Strep to release cells. Cells were collected and washed with PBS twice, and then fixed with precooled anhydrous ethanol overnight. Cells were washed twice with ice-cold PBS and then resuspend with 500 µl PBS containing 0.25% Triton-100. The final concentration of PI and RNA enzyme was added to 50 µg/ml (PI) and 10 µg/ml (RNAase) under dark conditions. After incubation at 37 ℃ for 30 min, the machine was directly used for detection within 1 hour. FLOWJ was used for cell cycle analysis.
