# Supplementary figures and images for: CpG-binding protein CFP1 promotes ovarian cancer cell proliferation by regulating BST2 transcription
Source: Cancer Gene Ther. 2022 Jul 21;29(12):1895–907. doi: 10.1038/s41417-022-00503-z (PMC9750859; doi:10.1038/s41417-022-00503-z)

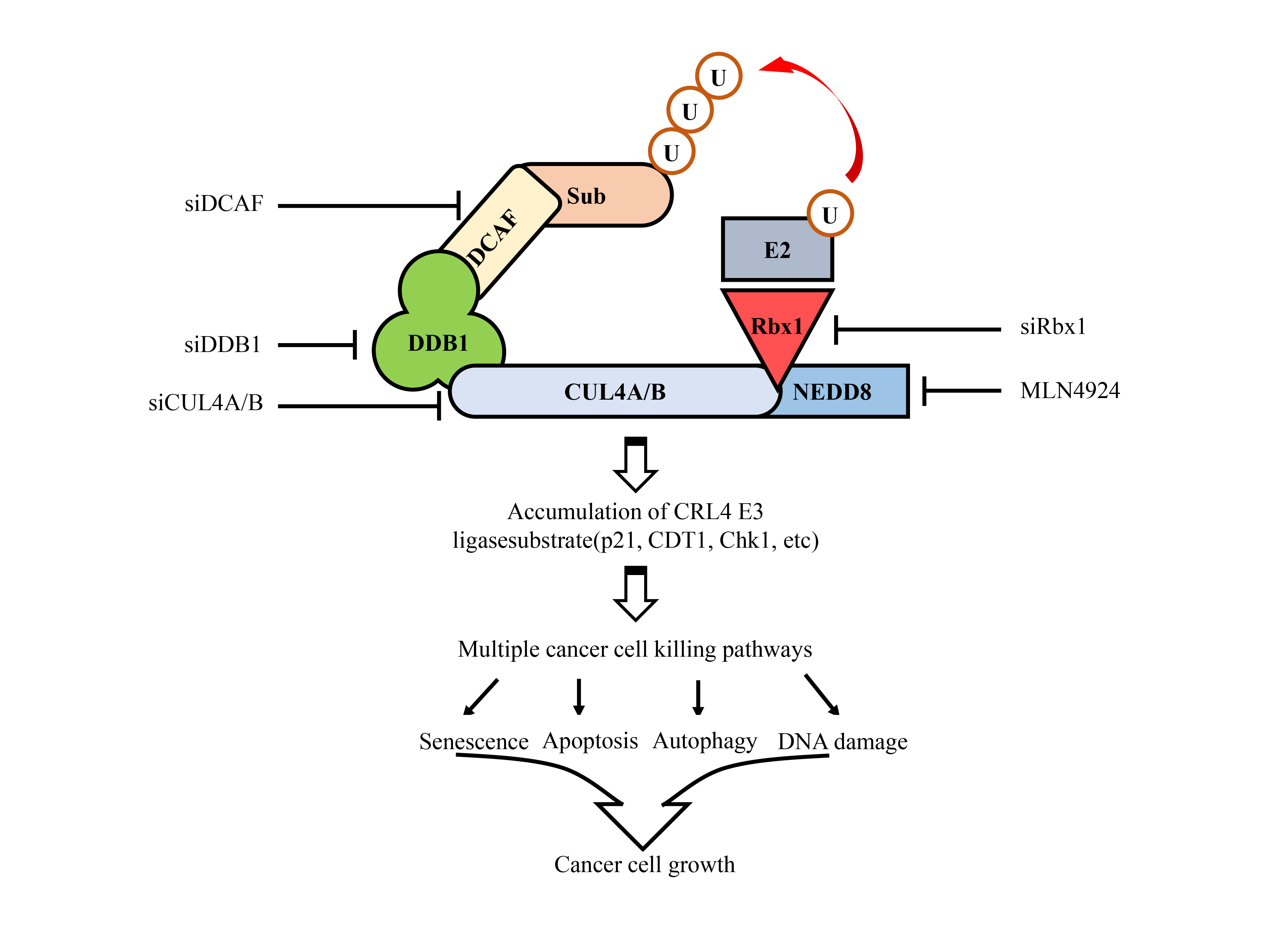

Supplement: Supplementary file 2 — Supplementary figure 1 [file 41417_2022_503_MOESM2_ESM.jpg]

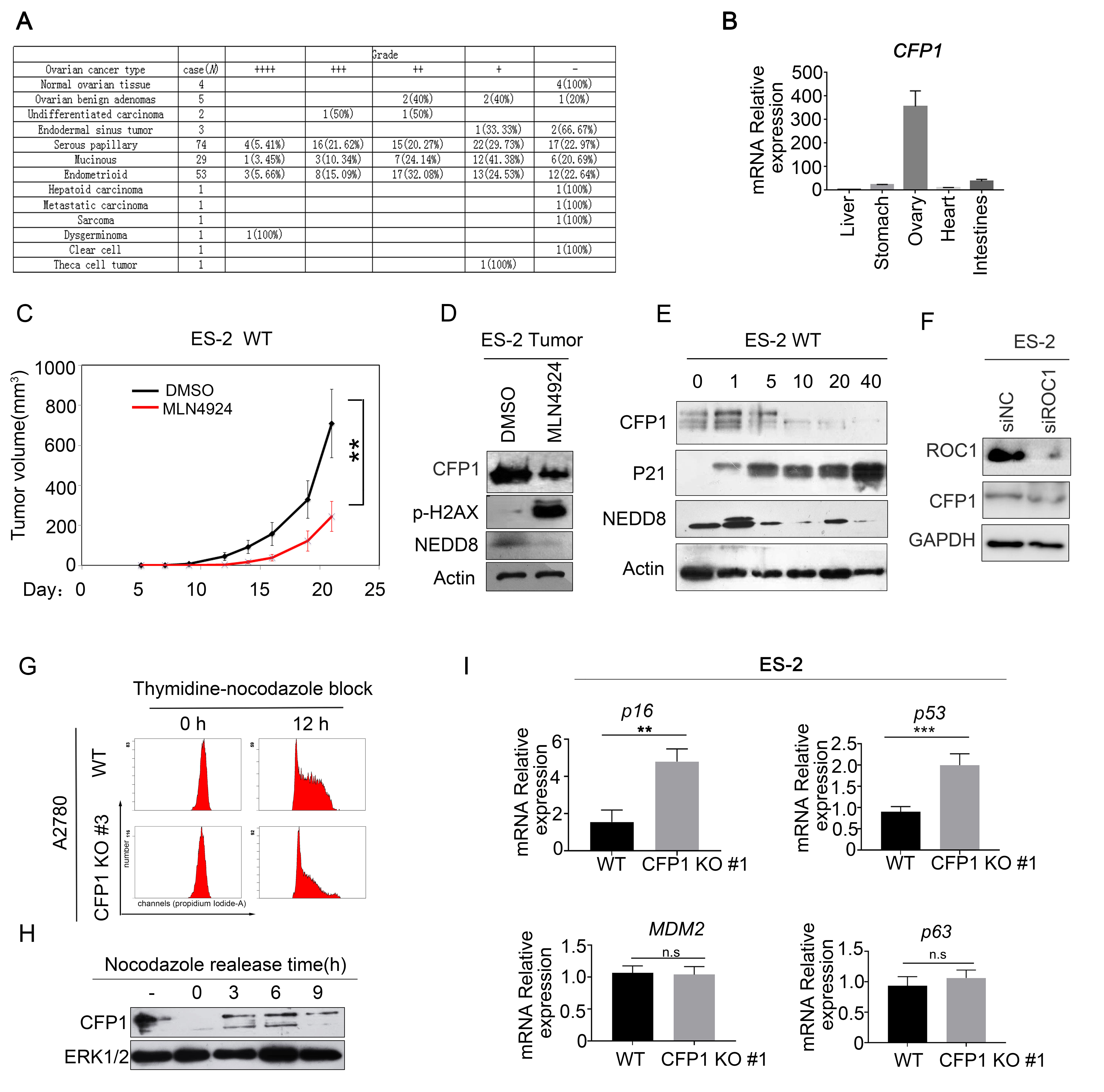

Supplement: Supplementary file 3 — Supplementary figure 2 [file 41417_2022_503_MOESM3_ESM.jpg]

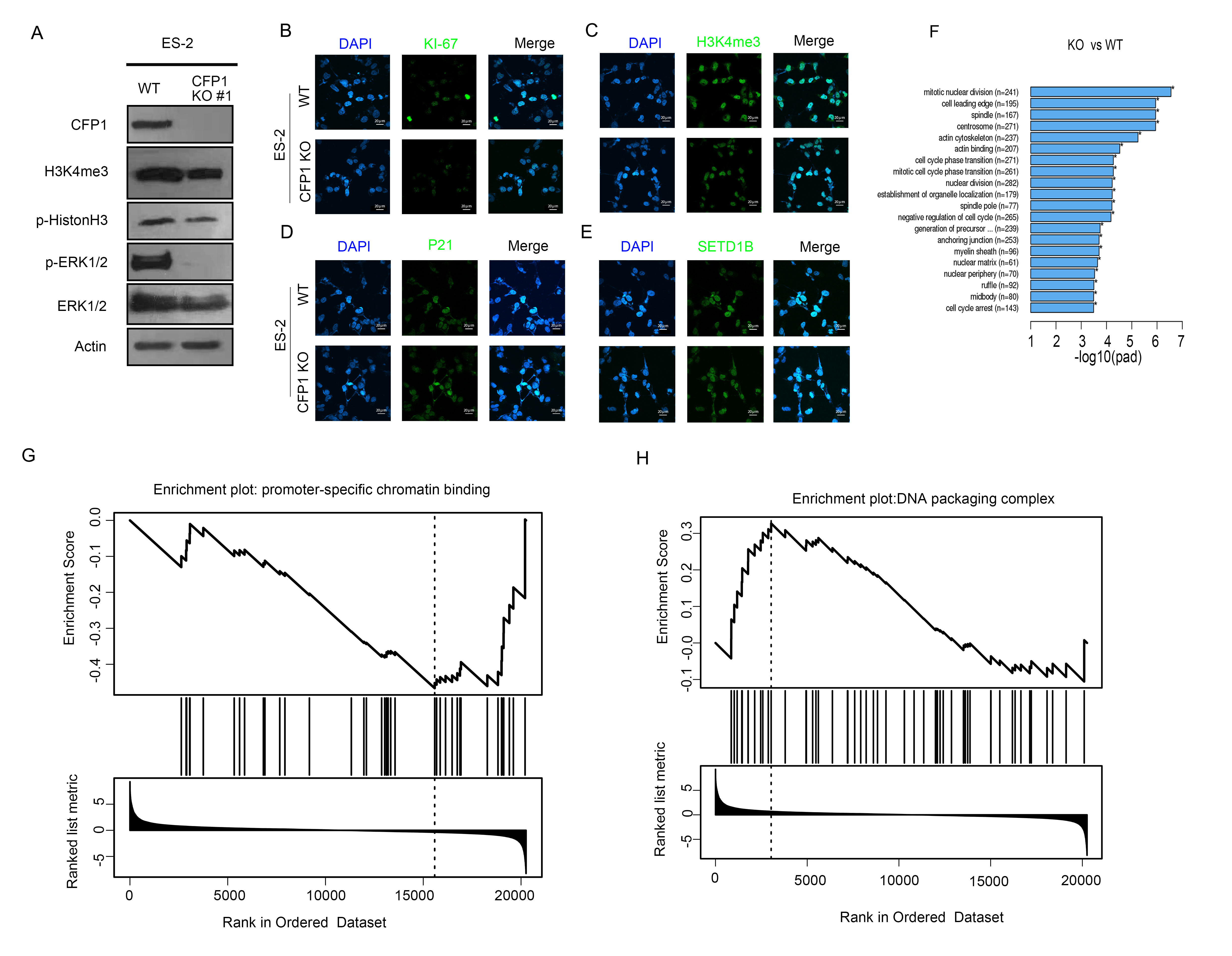

Supplement: Supplementary file 4 — Supplementary figure 3 [file 41417_2022_503_MOESM4_ESM.jpg]

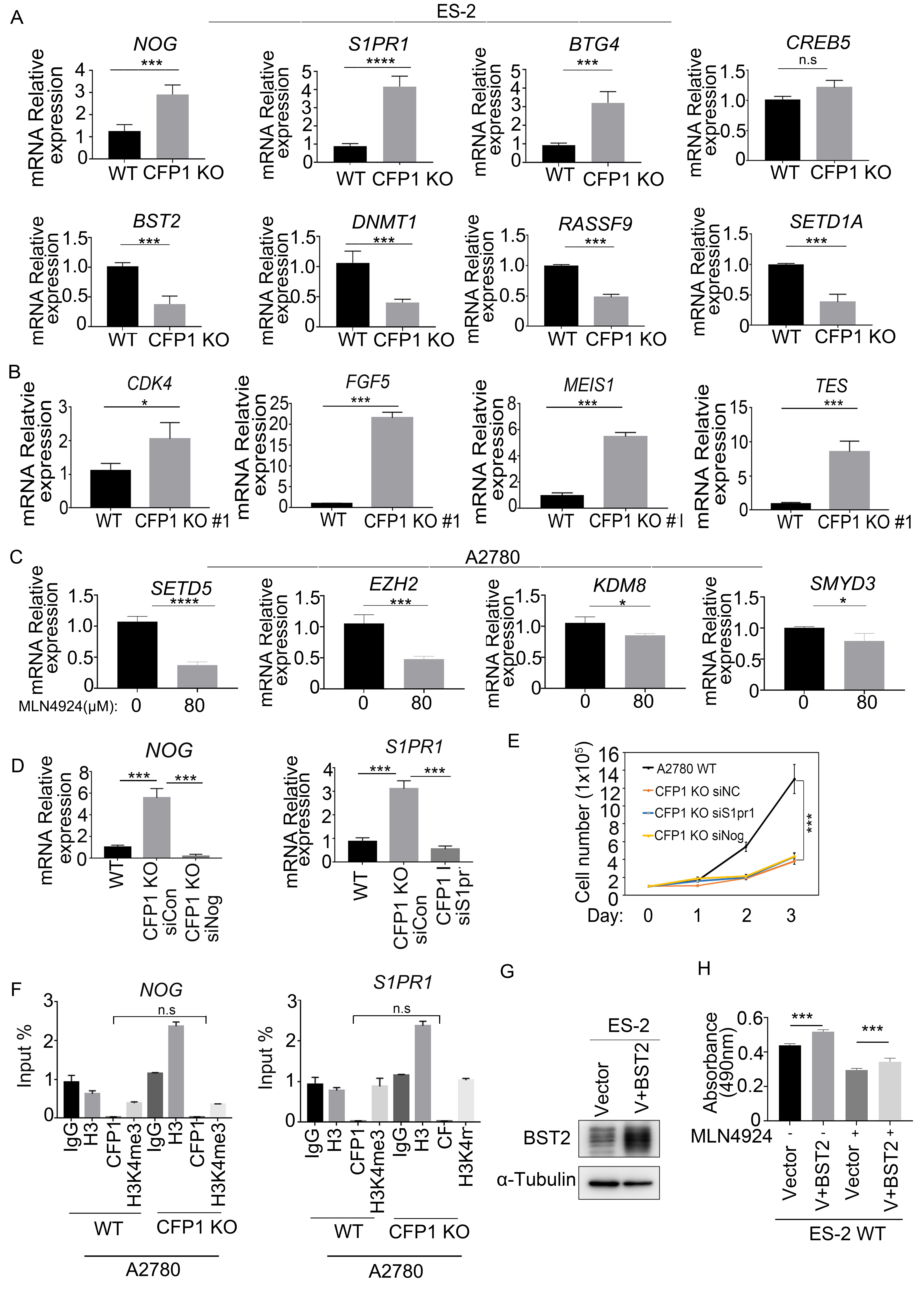

Supplement: Supplementary file 5 — Supplementary figure 4 [file 41417_2022_503_MOESM5_ESM.jpg]
