# Supplementary material for: CpG-binding protein CFP1 promotes ovarian cancer cell proliferation by regulating BST2 transcription
Source: Cancer Gene Ther. 2022 Jul 21;29(12):1895–907. doi: 10.1038/s41417-022-00503-z (PMC9750859; doi:10.1038/s41417-022-00503-z)
Supplement: Supplementary file 7 — Dataset 1 [file 41417_2022_503_MOESM7_ESM.pdf]

Pos	Name	Ct SYBR	Amount SYBR	Target SYBR
A1		14.85	-	
A2		14.79	-	
A3		14.75	-	
A4		14.84	-	
A5		11.41	-	
A6		11.46	-	
A7		10.91	-	
A8		11.1	-	
A9			-	
A10		12.17	-	
A11		10.51	-	
A12			-	
B1		35.36	-	
B2		33.06	-	
B3		32.64	-	
B4		32.89	-	
B5		33.66	-	
B6		33.26	-	
B7		33.71	-	
B8		32.03	-	
B9			-	
B10		32.39	-	
B11		31.36	-	
B12			-	
C1		29.48	-	
C2		29.46	-	
C3		29.33	-	
C4		29.56	-	
C5		25.64	-	
C6		25.76	-	
C7		25.45	-	
C8		25.54	-	
C9			-	
C10		25.85	-	
C11		25.56	-	
C12			-	
D1		27.62	-	
D2		27.34	-	
D3		28.9	-	
D4		28.16	-	
D5		26.92	-	
D6		26.91	-	
D7		26.99	-	
D8		26.73	-	
D9			-	
D10		25.45	-	

D11	25.03	-
D12		-
E1	27.42	-
E2	27.45	-
E3	26.87	-
E4	27.64	-
E5	21.83	-
E6	21.83	-
E7	21.89	-
E8	22.2	-
E9		-
E10	22.53	-
E11	22.5	-
E12		-
F1	18.41	-
F2	18.36	-
F3	18.35	-
F4	18.41	-
F5	16.01	-
F6	15.89	-
F7	15.75	-
F8	15.76	-
F9		-
F10	15.75	-
F11	16.23	-
F12		-
G1	11.04	-
G2	34.11	-
G3	25.89	-
G4	25.3	-
G5	22.62	-
G6	16.2	-
G7		-
G8		-
G9		-
G10		-
G11		-
G12		-
H1	10.78	-
H2	33.5	-
H3	25.78	-
H4	25.26	-
H5	22.72	-
H6	16.35	-
H7		-
H8		-
H9		-

H10	-
H11	-
H12	-

Threshold detection parameters:

Thresh(87 (Noiseband)

Baselirautomatic

Drift (OFF

Pos	Name	Ct SYBR	Amount SYTarget SYBR
A1		14.49	-
A2		14.58	-
A3		14.57	-
A4		14.74	-
A5		10.83	-
A6		10.84	-
A7		10.84	-
A8		8.71	-
A9		10.47	-
A10		11.96	-
A11		9.7	-
A12		10.21	-
B1		19.5	-
B2		20.14	-
B3		19.22	-
B4		19.9	-
B5		17.17	-
B6		17.47	-
B7		17.5	-
B8		17.49	-
B9		16.92	-
B10		16.85	-
B11		16.83	-
B12		16.81	-
C1			-
C2		40.4	-
C3		42.65	-
C4			-
C5		2.37	-
C6		42.39	-
C7		43.58	-
C8			-
C9		37.41	-
C10		37.26	-
C11			-
C12		35.69	-
D1			-
D2			-
D3			-
D4			-
D5			-
D6			-
D7			-
D8			-
D9			-
D10			-

D11		–
D12		–
E1		–
E2		–
E3		–
E4		–
E5		–
E6		–
E7		–
E8		–
E9		–
E10		–
E11		–
E12		–
F1		–
F2		–
F3		–
F4		–
F5		–
F6		–
F7		–
F8		–
F9	36.77	–
F10		–
F11		–
F12		–
G1		–
G2		–
G3		–
G4		–
G5		–
G6		–
G7		–
G8		–
G9		–
G10		–
G11		–
G12		–
H1		–
H2		–
H3		–
H4		–
H5		–
H6		–
H7		–
H8		–
H9		–

H10	-
H11	-
H12	-

Threshold detection parameters:

Thresh‹77 (Noiseband)

Baselirautomatic

Drift (OFF

Pos	Name	Ct SYBR	Amount S\	Target SYBR
A1		14.11	-	
A2		14.12	-	
A3		14.28	-	
A4		14.34	-	
A5		14.98	-	
A6		14.96	-	
A7		14.81	-	
A8		14.95	-	
A9		14.81	-	
A10		15.64	-	
A11		14.41	-	
A12		14.6	-	
B1		34.42	-	
B2		31.71	-	
B3		31.63	-	
B4		32.87	-	
B5		30.86	-	
B6		32.51	-	
B7		31.91	-	
B8		32.88	-	
B9		31.9	-	
B10		32.8	-	
B11		31.8	-	
B12		30.47	-	
C1		28.63	-	
C2		28.46	-	
C3		28.64	-	
C4		28.77	-	
C5		31.26	-	
C6		30.31	-	
C7		31.3	-	
C8		30.45	-	
C9		27.81	-	
C10		27.94	-	
C11		27.84	-	
C12		27.74	-	
D1		34.84	-	
D2		33.67	-	
D3		35.36	-	
D4		36.36	-	
D5		35.4	-	
D6		34.02	-	
D7		34.79	-	
D8		34.93	-	
D9		34.89	-	
D10		34.2	-	

D11	34.84	-
D12	35.45	-
E1	14.1	-
E2	13.94	-
E3	14.59	-
E4	14.08	-
E5	14.22	-
E6	14.55	-
E7	14.29	-
E8	14.16	-
E9	14.11	-
E10	13.91	-
E11	14.17	-
E12	14.31	-
F1	27.47	-
F2	27.7	-
F3	27.33	-
F4	27.29	-
F5	29.51	-
F6	30.15	-
F7	29.4	-
F8	28.99	-
F9	30.38	-
F10	29.35	-
F11	29.61	-
F12	29.17	-
G1	19.99	-
G2	20.98	-
G3	19.96	-
G4	20.16	-
G5	20.97	-
G6	20.96	-
G7	20.73	-
G8	20.75	-
G9	20.71	-
G10	20.4	-
G11	20.41	-
G12	20.6	-
H1	38.92	-
H2	34.21	-
H3	35.62	-
H4	35.93	-
H5	34	-
H6	38.66	-
H7	34.7	-
H8	34.82	-
H9	36.98	-

H10	35.97	-
H11	34.77	-
H12	39.19	-

Threshold detection parameters:

Thresh(95 (Noiseband)

Baselirautomatic

Drift (OFF

Pos	Name	Ct SYBR	Amount S\	Target SYBR
A1		14.11	-	
A2		14.12	-	
A3		14.28	-	
A4		14.34	-	
A5		14.98	-	
A6		14.96	-	
A7		14.81	-	
A8		14.95	-	
A9		14.81	-	
A10		15.64	-	
A11		14.41	-	
A12		14.6	-	
B1		34.42	-	
B2		31.71	-	
B3		31.63	-	
B4		32.87	-	
B5		30.86	-	
B6		32.51	-	
B7		31.91	-	
B8		32.88	-	
B9		31.9	-	
B10		32.8	-	
B11		31.8	-	
B12		30.47	-	
C1		28.63	-	
C2		28.46	-	
C3		28.64	-	
C4		28.77	-	
C5		31.26	-	
C6		30.31	-	
C7		31.3	-	
C8		30.45	-	
C9		27.81	-	
C10		27.94	-	
C11		27.84	-	
C12		27.74	-	
D1		34.84	-	
D2		33.67	-	
D3		35.36	-	
D4		36.36	-	
D5		35.4	-	
D6		34.02	-	
D7		34.79	-	
D8		34.93	-	
D9		34.89	-	
D10		34.2	-	

D11	34.84	-
D12	35.45	-
E1	14.1	-
E2	13.94	-
E3	14.59	-
E4	14.08	-
E5	14.22	-
E6	14.55	-
E7	14.29	-
E8	14.16	-
E9	14.11	-
E10	13.91	-
E11	14.17	-
E12	14.31	-
F1	27.47	-
F2	27.7	-
F3	27.33	-
F4	27.29	-
F5	29.51	-
F6	30.15	-
F7	29.4	-
F8	28.99	-
F9	30.38	-
F10	29.35	-
F11	29.61	-
F12	29.17	-
G1	19.99	-
G2	20.98	-
G3	19.96	-
G4	20.16	-
G5	20.97	-
G6	20.96	-
G7	20.73	-
G8	20.75	-
G9	20.71	-
G10	20.4	-
G11	20.41	-
G12	20.6	-
H1	38.92	-
H2	34.21	-
H3	35.62	-
H4	35.93	-
H5	34	-
H6	38.66	-
H7	34.7	-
H8	34.82	-
H9	36.98	-

H10	35.97 -
H11	34.77 -
H12	39.19 -

Threshold detection parameters:
  Thresh(95 (Noiseband)
  Baseliı automatic
  Drift (OFF

Pos	Name	Ct SYBR	Amount S	Target SYBR
A1		15.63	-	
A2		15.26	-	
A3		15.61	-	
A4		15.85	-	
A5		16.18	-	
A6		16.01	-	
A7		15.97	-	
A8		15.33	-	
A9			-	
A10			-	
A11			-	
A12			-	
B1		36.91	-	
B2			-	
B3		10.57	-	
B4			-	
B5			-	
B6			-	
B7		34.69	-	
B8			-	
B9			-	
B10			-	
B11			-	
B12			-	
C1			-	
C2			-	
C3		34.95	-	
C4		35.57	-	
C5			-	
C6			-	
C7			-	
C8			-	
C9			-	
C10			-	
C11			-	
C12			-	
D1		23.64	-	
D2		23.48	-	
D3		23.67	-	
D4		23.49	-	
D5		23.14	-	
D6		22.99	-	
D7		22.75	-	
D8		22.71	-	
D9			-	
D10			-	

D11		-
D12		-
E1		-
E2		-
E3		-
E4		-
E5		-
E6	37.29	-
E7	38.57	-
E8		-
E9		-
E10		-
E11		-
E12		-
F1	15.11	-
F2	15.01	-
F3	15.25	-
F4	15.62	-
F5	27.61	-
F6	27.89	-
F7	28.09	-
F8	27.8	-
F9		-
F10		-
F11		-
F12		-
G1	11.14	-
G2	11.56	-
G3	11.58	-
G4	11.77	-
G5	23.82	-
G6	24.14	-
G7	23.88	-
G8	23.92	-
G9		-
G10		-
G11		-
G12		-
H1	11.47	-
H2	11.71	-
H3	11.63	-
H4	11.99	-
H5	24.15	-
H6	23.88	-
H7	23.56	-
H8	23.79	-
H9		-

H10	-
H11	-
H12	-

Threshold detection parameters:

Thresh‹70 (Noiseband)

Baselirautomatic

Drift (OFF

Pos	Name	Ct SYBR	Amount S'Target SYBR
A1		15.41	-
A2		15.78	-
A3		15.95	-
A4		16.03	-
A5		15.4	-
A6		15.62	-
A7		15.41	-
A8		16.46	-
A9		15.61	-
A10		16.68	-
A11		14.95	-
A12		14.72	-
B1			-
B2			-
B3			-
B4		40.32	-
B5			-
B6			-
B7			-
B8		42.61	-
B9			-
B10			-
B11			-
B12		42.39	-
C1		19.19	-
C2		19.59	-
C3		19.24	-
C4		19.33	-
C5		18.75	-
C6		19.16	-
C7		18.66	-
C8		18.7	-
C9		18.69	-
C10		18.52	-
C11		18.31	-
C12		18.39	-
D1		9.18	-
D2		23.56	-
D3		23.44	-
D4		23.15	-
D5		23.19	-
D6		23.32	-
D7		23.13	-
D8		22.83	-
D9		22.37	-
D10		22.68	-

D11	22.2	-
D12	22.53	-
E1	27.22	-
E2	27.12	-
E3	27.24	-
E4	27.23	-
E5	26.86	-
E6	26.92	-
E7	26.69	-
E8	26.82	-
E9	28.34	-
E10	26.98	-
E11	26.98	-
E12	26.91	-
F1		-
F2		-
F3		-
F4		-
F5		-
F6		-
F7		-
F8		-
F9		-
F10		-
F11		-
F12		-
G1		-
G2		-
G3		-
G4		-
G5		-
G6		-
G7		-
G8		-
G9		-
G10		-
G11		-
G12		-
H1		-
H2		-
H3		-
H4		-
H5		-
H6		-
H7		-
H8		-
H9		-

H10	-
H11	-
H12	-

Threshold detection parameters:

Thresh(74 (Noiseband)

Baselirautomatic

Drift (OFF

Pos	Name	Ct SYBR	Amount SY	Target SYBR
A1		15.98	-	
A2		15.61	-	
A3		15.7	-	
A4		15.92	-	
A5		15.71	-	
A6		15.92	-	
A7		15.71	-	
A8		15.77	-	
A9			-	
A10			-	
A11			-	
A12			-	
B1		29.26	-	
B2		29.51	-	
B3		28.66	-	
B4		29.71	-	
B5		32.21	-	
B6		31.88	-	
B7		32.22	-	
B8		32.9	-	
B9			-	
B10			-	
B11			-	
B12			-	
C1		24.49	-	
C2		24.11	-	
C3		24.13	-	
C4		24.22	-	
C5		24.35	-	
C6		23.85	-	
C7		23.88	-	
C8		23.75	-	
C9			-	
C10			-	
C11			-	
C12			-	
D1		32.82	-	
D2		33.38	-	
D3		33.55	-	
D4		34.51	-	
D5		35.09	-	
D6		33.09	-	
D7		33.23	-	
D8		33.41	-	
D9			-	
D10			-	

D11		-
D12		-
E1	21.64	-
E2	21.94	-
E3	21.79	-
E4	22.12	-
E5	22.41	-
E6	22.49	-
E7	22.19	-
E8	22.44	-
E9		-
E10		-
E11		-
E12		-
F1	28.27	-
F2	28.58	-
F3	28.97	-
F4	28.93	-
F5	30.68	-
F6	30.33	-
F7	29.22	-
F8	30.53	-
F9		-
F10		-
F11		-
F12		-
G1	20.62	-
G2	20.72	-
G3	20.86	-
G4	20.62	-
G5	20.89	-
G6	21.15	-
G7	20.73	-
G8	20.98	-
G9		-
G10		-
G11		-
G12		-
H1	27.09	-
H2	26.62	-
H3	26.91	-
H4	27.98	-
H5	29.6	-
H6	29.67	-
H7	28.85	-
H8	29.54	-
H9		-

H10	-
H11	-
H12	-

Threshold detection parameters:

Thresh‹77 (Noiseband)
Baselirautomatic
Drift (OFF

Pos	Name	Ct SYBR	Amour	Target SYBR
A1		15.13	-	
A2		15.26	-	
A3		15.65	-	
A4		15.48	-	
A5		15.34	-	
A6		15.5	-	
A7		15.24	-	
A8		16.04	-	
A9			-	
A10			-	
A11			-	
A12			-	
B1		21.57	-	
B2		21.87	-	
B3		21.82	-	
B4		21.59	-	
B5		21.95	-	
B6		21.87	-	
B7		22.08	-	
B8		21.85	-	
B9			-	
B10			-	
B11			-	
B12			-	
C1		35.05	-	
C2		34	-	
C3			-	
C4		41.39	-	
C5		1.91	-	
C6		36.59	-	
C7		42.08	-	
C8		35.16	-	
C9			-	
C10			-	
C11			-	
C12			-	
D1		15.52	-	
D2		15.51	-	
D3		15.82	-	
D4		15.86	-	
D5		15.49	-	
D6		15.86	-	
D7		15.39	-	
D8		15.3	-	
D9			-	
D10			-	

D11		-
D12		-
E1	23.01	-
E2	23.32	-
E3	22.84	-
E4	22.98	-
E5	22.41	-
E6	22.39	-
E7	22.5	-
E8	22.93	-
E9		-
E10		-
E11		-
E12		-
F1	38.73	-
F2	34.17	-
F3	36.47	-
F4		-
F5	34.77	-
F6	37.15	-
F7	36.43	-
F8	34.08	-
F9		-
F10		-
F11		-
F12		-
G1		-
G2		-
G3		-
G4		-
G5		-
G6		-
G7		-
G8		-
G9		-
G10		-
G11		-
G12		-
H1		-
H2		-
H3		-
H4		-
H5		-
H6		-
H7		-
H8		-
H9		-

H10	-
H11	-
H12	-

Threshold detection parameters:

Thresh‹68 (Noiseband)

Baselirautomatic

Drift (OFF

Pos	Name	Ct SYBR	Amount SY	Target SY
A1		17.54	-	
A2		17.53	-	
A3		17.78	-	
A4		17.71	-	
A5		25.06	-	
A6		25.13	-	
A7		25.1	-	
A8		23.44	-	
A9		17.06	-	
A10		18.09	-	
A11		16.38	-	
A12		16.03	-	
B1		21.63	-	
B2		21.66	-	
B3		21.6	-	
B4		21.85	-	
B5		26.42	-	
B6		28.13	-	
B7		28.23	-	
B8		28.18	-	
B9		21.61	-	
B10		21.9	-	
B11		21.51	-	
B12		21.32	-	
C1		25.07	-	
C2		24.87	-	
C3		24.92	-	
C4		25.05	-	
C5		32.74	-	
C6		31.85	-	
C7		32.26	-	

C8	31.72	-
C9		-
C10		-
C11		-
C12		-
D1	32.11	-
D2	34.56	-
D3	34.78	-
D4	36.08	-
D5	35.92	-
D6	36.83	-
D7	34.71	-
D8	32.6	-
D9		-
D10		-
D11		-
D12		-
E1	16.65	-
E2	16.59	-
E3	16.69	-
E4	16.51	-
E5	17.09	-
E6	16.68	-
E7	17	-
E8	16.77	-
E9	17.1	-
E10	17.01	-
E11	16.72	-
E12	15.96	-
F1	28	-
F2	27.71	-
F3	27.69	-
F4	27.69	-
F5	22.68	-
F6	22.53	-
F7	22.49	-
F8	22.43	-
F9	29.03	-
F10	28.06	-
F11	28.08	-
F12	28.09	-
G1	23.45	-
G2	23.52	-
G3	24.28	-
G4	23.69	-
G5	23.31	-
G6	23.59	-

G7	23.26	-
G8	23.46	-
G9		-
G10		-
G11		-
G12		-
H1	32.87	-
H2	33.44	-
H3	33.76	-
H4	33.8	-
H5	33.74	-
H6	35.76	-
H7	34.16	-
H8	33.18	-
H9		-
H10		-
H11		-
H12		-

Threshold detection parameters:

Thresh(95 (Noiseband)

Baselir automatic

Drift (OFF

Pos	Name	Ct SYBR	Amount SY	Target SYBR
A1	nc 24h	12.08	-	
A2		11.83	-	
A3		12.08	-	
A4		12.11	-	
A5		33.86	-	
A6		33.83	-	
A7		34.15	-	
A8		33.98	-	
A9			-	
A10			-	
A11			-	
A12			-	
B1	nog24h	12.49	-	
B2		12.19	-	
B3		11.94	-	
B4		11.9	-	
B5		33.85	-	
B6		33.52	-	
B7		33.45	-	
B8		33.3	-	
B9			-	
B10			-	
B11			-	
B12			-	
C1	nc48h	11.82	-	
C2		11.76	-	
C3		12.03	-	
C4		11.89	-	
C5		32.21	-	
C6		32.09	-	
C7		32.49	-	
C8		32.5	-	
C9			-	
C10			-	
C11			-	
C12			-	
D1	nog48h	13.49	-	

Pos	Name	Ct SYBR	Amount S	Target SYBR
A1		15.64	-	
A2		15.54	-	
A3		15.84	-	
A4		15.76	-	
A5		35.18	-	
A6		35.72	-	
A7		40.25	-	
A8		34.71	-	
A9		29.41	-	
A10		30.7	-	
A11		29.2	-	
A12		29.51	-	
B1		13.32	-	
B2		13.08	-	
B3		13.24	-	
B4		13.35	-	
B5		35.18	-	
B6		35.38	-	
B7		33.82	-	
B8		32.44	-	
B9		29.85	-	
B10		29.46	-	
B11		29.81	-	
B12		29.44	-	
C1		13.84	-	
C2		13.72	-	
C3		13.68	-	
C4		14.27	-	
C5		33.76	-	
C6		36.75	-	
C7		33.97	-	
C8		33.74	-	
C9		28.8	-	
C10		28.73	-	
C11		28.66	-	
C12		28.38	-	
D1		22.03	-	
D2		21.63	-	
D3		21.92	-	
D4		22.03	-	
D5		34.7	-	
D6		34.04	-	
D7		33.9	-	
D8		34.83	-	
D9		30.1	-	
D10		29.9	-	

D11	29.19	-
D12	29.67	-
E1	18.06	-
E2	17.55	-
E3	17.77	-
E4	18.26	-
E5	34.42	-
E6	38.15	-
E7	34.94	-
E8	35.9	-
E9	29.54	-
E10	29.28	-
E11	29.48	-
E12	29.08	-
F1	17.54	-
F2	17.08	-
F3	16.95	-
F4	17.28	-
F5	35.12	-
F6	40.43	-
F7	41.88	-
F8	32.5	-
F9	29.07	-
F10	7.16	-
F11	29.25	-
F12	28.74	-
G1	12.96	-
G2	13.08	-
G3	12.96	-
G4	13.05	-
G5	37.04	-
G6	35.39	-
G7	39.83	-
G8	35.22	-
G9		-
G10		-
G11		-
G12		-
H1	13.59	-
H2	13.44	-
H3	13.84	-
H4	13.67	-
H5	30.9	-
H6	31.18	-
H7	31.38	-
H8	30.4	-
H9	31.17	-

H10	30.7	-
H11	31.22	-
H12	30.03	-

Threshold detection parameters:

Thresh(97 (Noiseband)

Baselirautomatic

Drift (OFF

Pos	Name	Ct SYBR	Amount SYTarget SYBR
A1		15.68	-
A2		16.33	-
A3		16.15	-
A4		17.42	-
A5		17.04	-
A6			-
A7		27.86	-
A8		31.15	-
A9			-
A10			-
A11			-
A12			-
B1		16.02	-
B2		16.57	-
B3		16.06	-
B4		16.73	-
B5		16.64	-
B6		14.98	-
B7		27.6	-
B8		32.06	-
B9			-
B10			-
B11			-
B12			-
C1		15.47	-
C2		17.23	-
C3		16.02	-
C4		17.1	-
C5		16.99	-
C6		14.96	-
C7		27.89	-
C8		31.51	-
C9			-
C10			-
C11			-
C12			-
D1		15.72	-
D2		17.05	-
D3		16.75	-
D4		16.76	-
D5		17.24	-
D6		15.14	-
D7		28.67	-
D8		33.23	-
D9			-
D10			-

D11		-
D12		-
E1	28.67	-
E2	27.62	-
E3	27.4	-
E4	23.01	-
E5	27.6	-
E6	14.94	-
E7	23.54	-
E8	25.61	-
E9		-
E10		-
E11		-
E12		-
F1	28.13	-
F2	27.67	-
F3	28.17	-
F4	28.48	-
F5	27.47	-
F6	15.03	-
F7	23.84	-
F8	25.47	-
F9		-
F10		-
F11		-
F12		-
G1	28.67	-
G2	27.26	-
G3	28.2	-
G4	27.72	-
G5	27.45	-
G6	15.04	-
G7	23.84	-
G8	26	-
G9		-
G10		-
G11		-
G12		-
H1	28.82	-
H2		-
H3	27.73	-
H4	27.09	-
H5	27.9	-
H6	29.05	-
H7		-
H8	25.61	-
H9		-

H10	-
H11	-
H12	-

Threshold detection parameters:

Thresh‹70 (Noiseband)

Baselirautomatic

Drift (OFF

Pos	Name	Ct SYBR	Amount S'	Target SYBR
A1		16.8	-	
A2		17.75	-	
A3		17.36	-	
A4		17.81	-	
A5		17.38	-	
A6		15.52	-	
A7		28.66	-	
A8		32.99	-	
A9		31.75	-	
A10		33.52	-	
A11			-	
A12			-	
B1		16.69	-	
B2		17.75	-	
B3		16.98	-	
B4		18.01	-	
B5		17.71	-	
B6		15.66	-	
B7		28.62	-	
B8		34.95	-	
B9		32.16	-	
B10		31.62	-	
B11			-	
B12			-	
C1		16.67	-	
C2		17.57	-	
C3		16.98	-	
C4		13.72	-	
C5		17.86	-	
C6		15.57	-	
C7		28.21	-	
C8			-	
C9		33.4	-	
C10		31.88	-	
C11			-	
C12			-	
D1		16.94	-	
D2		17.9	-	
D3		17.62	-	
D4		18.07	-	
D5		17.83	-	
D6		15.61	-	
D7		28.98	-	
D8		32.58	-	
D9		35.28	-	
D10		32.03	-	

D11		-
D12		-
E1	28.92	-
E2	28.43	-
E3	28.77	-
E4	29.75	-
E5	28.01	-
E6	15.69	-
E7	23.47	-
E8	26.31	-
E9	31.11	-
E10	28.47	-
E11		-
E12		-
F1	28.71	-
F2	29.07	-
F3	29	-
F4	28.96	-
F5	27.81	-
F6	15.63	-
F7	24.06	-
F8	26.45	-
F9	30.85	-
F10	28.76	-
F11		-
F12		-
G1	29.24	-
G2	28.55	-
G3	28.76	-
G4	28.88	-
G5	28.22	-
G6	15.8	-
G7	23.69	-
G8	26.44	-
G9	30.77	-
G10	28.61	-
G11		-
G12		-
H1	29.33	-
H2	28.79	-
H3	28.65	-
H4	28.44	-
H5	28.49	-
H6	15.62	-
H7	23.62	-
H8	26.07	-
H9	31.11	-

H10	28.72	-
H11		-
H12		-

Threshold detection parameters:
  Thresh(107 (Noiseband)
  Baselirautomatic
  Drift (OFF

Pos	Name	Ct SYBR	Amount SY	Target SYBR
A1		26.11	-	
A2		25.88	-	
A3		26.86	-	
A4		25.68	-	
A5			-	
A6			-	
A7			-	
A8		2.95	-	
A9		27.42	-	
A10		28.16	-	
A11		27.59	-	
A12		27.74	-	
B1		31.48	-	
B2		29.66	-	
B3		32.23	-	
B4		31.99	-	
B5			-	
B6			-	
B7			-	
B8			-	
B9		27.43	-	
B10		27.81	-	
B11		28.27	-	
B12		27.92	-	
C1		25.22	-	
C2		25.52	-	
C3		25.39	-	
C4		26.11	-	
C5		34.94	-	
C6		41.45	-	
C7			-	
C8		41.61	-	
C9			-	
C10			-	
C11			-	
C12			-	
D1		26.28	-	
D2		24.42	-	
D3		24.64	-	
D4		26.11	-	
D5		26.88	-	
D6		26.94	-	
D7		27.08	-	
D8		26.58	-	
D9			-	
D10			-	

D11	–
D12	–
E1	–
E2	–
E3	–
E4	–
E5	–
E6	–
E7	–
E8	–
E9	–
E10	–
E11	–
E12	–
F1	–
F2	–
F3	–
F4	–
F5	–
F6	–
F7	–
F8	–
F9	–
F10	–
F11	–
F12	–
G1	–
G2	–
G3	–
G4	–
G5	–
G6	–
G7	–
G8	–
G9	–
G10	–
G11	–
G12	–
H1	–
H2	–
H3	–
H4	–
H5	–
H6	–
H7	–
H8	–
H9	–

H10	-
H11	-
H12	-

Threshold detection parameters:

Threshc 56 (Noiseband)

Baselir automatic

Drift (OFF

Pos	Name	Ct SYBR	Amount S	Target SYBR
A1		17.54	-	
A2		17.53	-	
A3		17.78	-	
A4		17.71	-	
A5		25.06	-	
A6		25.13	-	
A7		25.1	-	
A8		23.44	-	
A9		17.06	-	
A10		18.09	-	
A11		16.38	-	
A12		16.03	-	
B1		21.63	-	
B2		21.66	-	
B3		21.6	-	
B4		21.85	-	
B5		26.42	-	
B6		28.13	-	
B7		28.23	-	
B8		28.18	-	
B9		21.61	-	
B10		21.9	-	
B11		21.51	-	
B12		21.32	-	
C1		25.07	-	
C2		24.87	-	
C3		24.92	-	
C4		25.05	-	
C5		32.74	-	
C6		31.85	-	
C7		32.26	-	
C8		31.72	-	
C9			-	
C10			-	
C11			-	
C12			-	
D1		32.11	-	
D2		34.56	-	
D3		34.78	-	

Pos	Name	+/- Resu	Ct SYBR
A1		Positive	15.58
A2		Positive	18.48
A3		Positive	34.26
A4		Positive	17.95
A5		Positive	14.9
A6		Positive	14.77
A7		Positive	42.16
A8		Positive	11.23
A9		Positive	41.4
A10		Positive	15.73
A11		Positive	40.01
A12		Negative	
B1		Positive	15.48
B2		Positive	15.39
B3		Positive	28.2
B4		Positive	15.93
B5		Positive	14.9
B6		Positive	14.51
B7		Positive	35.59
B8		Positive	13.08
B9		Positive	36.11
B10		Positive	14.1
B11		Positive	34.94
B12		Negative	
C1		Positive	15.56
C2		Positive	15.32
C3		Positive	14.92
C4		Positive	15
C5		Positive	14.87
C6		Positive	14.4
C7		Negative	
C8		Positive	13.45
C9		Positive	35.94
C10		Positive	13.98
C11		Negative	
C12		Negative	
D1		Positive	15.79
D2		Positive	14.83
D3		Positive	15
D4		Positive	14.86
D5		Positive	16.13
D6		Positive	14.88
D7		Negative	
D8		Positive	13.49
D9		Positive	38.85
D10		Positive	13.89

D11	Positive	37.25
D12	Negative	
E1	Positive	25.46
E2	Positive	29.67
E3	Positive	28.12
E4	Positive	27.96
E5	Positive	28
E6	Negative	
E7	Negative	
E8	Negative	
E9	Negative	
E10	Negative	
E11	Negative	
E12	Negative	
F1	Positive	25.81
F2	Positive	29.37
F3	Positive	28.92
F4	Positive	28.09
F5	Positive	27.47
F6	Negative	
F7	Negative	
F8	Negative	
F9	Negative	
F10	Negative	
F11	Negative	
F12	Negative	
G1	Positive	25.67
G2	Positive	29.3
G3	Negative	
G4	Positive	27.83
G5	Positive	27.93
G6	Negative	
G7	Negative	
G8	Negative	
G9	Negative	
G10	Negative	
G11	Negative	
G12	Negative	
H1	Positive	25.88
H2	Positive	30.17
H3	Positive	29
H4	Positive	27.84
H5	Positive	27.71
H6	Negative	
H7	Negative	
H8	Negative	
H9	Negative	

H10	Negative
H11	Negative
H12	Negative

Threshold detection parameters:

Thresh(	78 (Noiseband)
Baselir	automatic
Drift (	OFF

Pos	Name	Ct SYBR	Amount SY	Target SYBR
A1		21.79	-	
A2		26.15	-	
A3		21.42	-	
A4		21.82	-	
A5		25.96	-	
A6		20.87	-	
A7		20.42	-	
A8		22.82	-	
A9		24.24	-	
A10			-	
A11			-	
A12			-	
B1		21.45	-	
B2		25.82	-	
B3		20.93	-	
B4		21.76	-	
B5		26.03	-	
B6		20.91	-	
B7		20.84	-	
B8		19.98	-	
B9		24.08	-	
B10			-	
B11			-	
B12			-	
C1		21.82	-	
C2		25.6	-	
C3		21.19	-	
C4		21.71	-	
C5		26.25	-	
C6		20.79	-	
C7		20.17	-	
C8		19.66	-	
C9		24.2	-	
C10			-	
C11			-	
C12			-	
D1		21.86	-	
D2		25.74	-	
D3		20.86	-	
D4		21.7	-	
D5		26.3	-	
D6		21.33	-	
D7		19.92	-	
D8		19.87	-	
D9		24.16	-	
D10			-	

D11		-
D12		-
E1	21.56	-
E2	25.67	-
E3	22.05	-
E4	20.97	-
E5	25.82	-
E6	21.99	-
E7	20.42	-
E8	26.35	-
E9	19.61	-
E10		-
E11		-
E12		-
F1	21.9	-
F2	25.86	-
F3	22.06	-
F4	21.41	-
F5	35.68	-
F6	21.98	-
F7	19.98	-
F8	26.06	-
F9	19.61	-
F10		-
F11		-
F12		-
G1	21.51	-
G2	25.61	-
G3	21.74	-
G4	21.26	-
G5	25.32	-
G6	21.99	-
G7	20.08	-
G8	25.98	-
G9	19.95	-
G10		-
G11		-
G12		-
H1	21.36	-
H2	25.74	-
H3	21.82	-
H4	21.24	-
H5	25.7	-
H6	21.12	-
H7	19.92	-
H8	26.3	-
H9	19.58	-

H10	-
H11	-
H12	-

Threshold detection parameters:

Thresh‹107 (Noiseband)

Baselii automatic

Drift (OFF

Pos	Name	Ct SYBR	Amount SY	Target SYBR
A1		16.9	-	
A2		17.61	-	
A3		17.23	-	
A4		17.47	-	
A5		17.58	-	
A6		18.6	-	
A7		17.86	-	
A8		19.32	-	
A9		18.53	-	
A11		18.18	-	
A12		18.45	-	
B1		16.84	-	
B2		17.95	-	
B3		16.95	-	
B4		17.13	-	
B5		17.44	-	
B6		18.49	-	
B7		17.8	-	
B8		18.48	-	
B9		18.62	-	
B10		19.57	-	
B11		18.21	-	
B12		18.18	-	
C1		16.35	-	
C2		17.98	-	
C3		17.18	-	
C4		17.61	-	
C5		17.36	-	
C6		18.64	-	
C7		18.2	-	
C8		18.6	-	
C9		18.52	-	
C10		19.3	-	
C11		18.25	-	
C12		18.55	-	
D1		17.15	-	
D2		18.44	-	
D3		17.17	-	
D4		18.45	-	
D5		17.99	-	
D6		18.78	-	
D7		17.89	-	
D8		18.92	-	
D9		18.72	-	
D10		19.5	-	
D11		18.62	-	

D12	18.68	-
E1	26.69	-
E2	30	-
E3	29.81	-
E4		-
E5	29.73	-
E6	28.25	-
E7	28.33	-
E8	29.3	-
E9	28.18	-
E10	27.59	-
E11	29.02	-
E12	27.39	-
F1	32.66	-
F2	30.71	-
F3	28.65	-
F4	30.82	-
F5	30.91	-
F6	28.15	-
F7	28.48	-
F8	29.4	-
F9	28.05	-
F10	27.83	-
F11	27.76	-
F12	29.36	-
G1	26.61	-
G2	30.37	-
G3	29.19	-
G4	29.12	-
G5	29.79	-
G6	28.07	-
G7	28.66	-
G8	29.5	-
G9	28.3	-
G10	27.23	-
G11	28.89	-
G12	29.4	-
H1	26.3	-
H2	30.33	-
H3	29.45	-
H4	29.91	-
H5	28.72	-
H6	27.33	-
H7	27.83	-
H8	29.26	-
H9	27.89	-
H10	27.34	-

H11	29.72	-
H12	29.02	-

Threshold detection parameters:
  Thresh 162 (Noiseband)
  Baselir automatic
  Drift (OFF

Pos	Name	Ct SYBR	Amount SYTarget SYBR
A1		19.69	-
A2		19.85	-
A3		19.61	-
A4		19.6	-
A5		23.25	-
A6		23.49	-
A7		23.39	-
A8		23.82	-
A9			-
A10			-
A11			-
A12			-
B1		17.76	-
B2		17.75	-
B3		18.08	-
B4		18.06	-
B5			-
B6			-
B7			-
B8			-
B9			-
B10			-
B11			-
B12			-
C1		20.1	-
C2		20.47	-
C3		20.47	-
C4		20.49	-
C5		22.4	-
C6		22.28	-
C7		22.62	-
C8			-
C9			-
C10			-
C11			-
C12		16.55	-
D1		16.64	-
D2		16.74	-
D3		17.46	-
D4			-
D5			-
D6			-
D7			-
D8			-
D9			-
D10			-

D11		-
D12		-
E1	18.51	-
E2	19.1	-
E3	18.58	-
E4	18.58	-
E5	19.16	-
E6	19.14	-
E7	19.36	-
E8	19.39	-
E9		-
E10		-
E11		-
E12		-
F1	23.16	-
F2	23.5	-
F3	23.61	-
F4	24.11	-
F5		-
F6		-
F7		-
F8		-
F9		-
F10		-
F11		-
F12		-
G1	19.12	-
G2	19.35	-
G3	19.66	-
G4	19.74	-
G5	19.51	-
G6	19.49	-
G7	19.34	-
G8	20.49	-
G9		-
G10		-
G11		-
G12		-
H1	26	-
H2	26.4	-
H3	26.32	-
H4	26.4	-
H5		-
H6		-
H7		-
H8		-
H9		-

H10	-
H11	-
H12	-

Threshold detection parameters:

Thresh(84 (Noiseband)

Baselir automatic

Drift (OFF

Pos	Name	Ct SYBR	Amount SY	Target SYBR
A1		17.54	-	
A2		17.53	-	
A3		17.78	-	
A4		17.71	-	
A5		25.06	-	
A6		25.13	-	
A7		25.1	-	
A8		23.44	-	
A9		17.06	-	
A10		18.09	-	
A11		16.38	-	
A12		16.03	-	
B1		21.63	-	
B2		21.66	-	
B3		21.6	-	
B4		21.85	-	
B5		26.42	-	
B6		28.13	-	
B7		28.23	-	
B8		28.18	-	
B9		21.61	-	
B10		21.9	-	
B11		21.51	-	
B12		21.32	-	
C1		25.07	-	
C2		24.87	-	
C3		24.92	-	
C4		25.05	-	
C5		32.74	-	
C6		31.85	-	
C7		32.26	-	

C8	31.72	-
C9		-
C10		-
C11		-
C12		-
D1	32.11	-
D2	34.56	-
D3	34.78	-
D4	36.08	-
D5	35.92	-
D6	36.83	-
D7	34.71	-
D8	32.6	-
D9		-
D10		-
D11		-
D12		-
E1	16.65	-
E2	16.59	-
E3	16.69	-
E4	16.51	-
E5	17.09	-
E6	16.68	-
E7	17	-
E8	16.77	-
E9	17.1	-
E10	17.01	-
E11	16.72	-
E12	15.96	-
F1	28	-
F2	27.71	-
F3	27.69	-
F4	27.69	-
F5	22.68	-
F6	22.53	-
F7	22.49	-
F8	22.43	-
F9	29.03	-
F10	28.06	-
F11	28.08	-
F12	28.09	-
G1	23.45	-
G2	23.52	-
G3	24.28	-
G4	23.69	-
G5	23.31	-
G6	23.59	-

G7	23.26	-
G8	23.46	-
G9		-
G10		-
G11		-
G12		-
H1	32.87	-
H2	33.44	-
H3	33.76	-
H4	33.8	-
H5	33.74	-
H6	35.76	-
H7	34.16	-
H8	33.18	-
H9		-
H10		-
H11		-
H12		-

Threshold detection parameters:

Threshc	95 (Noiseband)
Baselir	automatic
Drift (	OFF

Pos	Name	Ct SYBR	Amount SY	Target SYBR
A1		19.96	-	
A2		15.61	-	
A3		15.69	-	
A4		16.55	-	
A5		16.63	-	
A6		16.8	-	
A7		14.41	-	
A8		15.15	-	
A9		15.88	-	
A10			-	
A11		20.54	-	
A12			-	
B1			-	
B2		20.58	-	
B3		20.85	-	
B4		18.71	-	
B5		15.77	-	
B6		16	-	
B7		14.86	-	
B8		14.8	-	
B9		14.66	-	
B10		16.2	-	
B11		16.66	-	
B12		15.21	-	
C1			-	
C2		30.21	-	
C3		30.1	-	
C4		32.23	-	
C5		30.96	-	
C6		31.6	-	
C7		29.14	-	
C8		29.5	-	
C9		30.56	-	
C10		16.2	-	
C11		16.92	-	
C12		15.49	-	
D1			-	
D2		29.61	-	
D3		29.76	-	
D4		30.5	-	
D5		30.93	-	
D6		31	-	
D7		28.58	-	
D8		27.95	-	
D9		28.85	-	
D10		16.25	-	
D11		16.94	-	
D12		15.32	-	
E1		17.04	-	
E2		15.38	-	
E3		15.68	-	
E4		16.6	-	
E5		16.11	-	
E6		16.87	-	
E7		14.97	-	
E8		14.99	-	
E9		14.87	-	

E10	40.96	-
E11	35.64	-
E12	36.57	-
F1	17.99	-
F2	15.9	-
F3	16.3	-
F4	15.27	-
F5	16.07	-
F6	14.93	-
F7	15.59	-
F8	15.5	-
F9	15.71	-
F10	37.86	-
F11		-
F12	37.41	-
G1	24.66	-
G2	20.41	-
G3	20.09	-
G4	20.77	-
G5	20.41	-
G6	21.21	-
G7	18.8	-
G8	18.73	-
G9	18.61	-
G10	35.6	-
G11		-
G12	33.2	-
H1		-
H2	20.6	-
H3	20.74	-
H4	20.02	-
H5	20.51	-
H6	20.24	-
H7	19.97	-
H8	20.55	-
H9	19.89	-
H10		-
H11	38.99	-
H12	33.56	-

Threshold detection parameters:

Threshold	159 (Noiseband)
Baseline S	automatic
Drift Corr	OFF

Pos	Name	Ct SYBR	Amount SY	Target SYBR
A1		18.21	-	
A2		15.93	-	
A3		16.06	-	
A4		16.61	-	
A5		16.76	-	
A6		16.91	-	
A7		15.09	-	
A8		15.54	-	
A9		18.92	-	
A10			-	
A11		18.77	-	
A12		18.49	-	
B1			-	
B2		15.28	-	
B3		14.94	-	
B4		16.09	-	
B5		15.97	-	
B6		16.18	-	
B7		15.28	-	
B8		15.14	-	
B9		15.23	-	
B10		14.87	-	
B11		15.86	-	
B12		15.12	-	
C1		35.7	-	
C2		37.28	-	
C3		35.49	-	
C4		35.2	-	
C5		35.65	-	
C6		34.78	-	
C7		33.85	-	
C8		38.33	-	
C9		20.47	-	
C10		14.74	-	
C11		15.76	-	
C12		15.23	-	
D1			-	
D2		34.8	-	
D3		32.93	-	
D4		35.28	-	
D5		36.79	-	
D6		36.49	-	
D7		35.39	-	
D8		35.53	-	
D9		34.49	-	
D10		15.5	-	
D11		16	-	
D12		15.34	-	
E1		30.69	-	
E2		28.32	-	
E3		28.21	-	
E4		30.65	-	
E5		29.82	-	

E6	30.76	-
E7	28.79	-
E8	28.89	-
E9	28.89	-
E10		-
E11	37.08	-
E12		-
F1	28.6	-
F2	27.93	-
F3	27.3	-
F4	30.55	-
F5	30.47	-
F6	30.1	-
F7	27.59	-
F8	27	-
F9	27.58	-
F10	40.41	-
F11		-
F12		-
G1	33.69	-
G2	34.91	-
G3	35.72	-
G4	33.83	-
G5	34.46	-
G6	32.89	-
G7	37.36	-
G8	34.4	-
G9	33.83	-
G10		-
G11	41.09	-
G12		-
H1		-
H2	38.18	-
H3	33.03	-
H4	32.38	-
H5	32.17	-
H6	31.94	-
H7	32.77	-
H8	32.89	-
H9	33.72	-
H10		-
H11		-
H12		-

Threshold detection parameters:

Threshc 193 (Noiseband)

Baselir automatic

Drift (OFF

Pos	Name	Ct SYBR	Amount SYBR	Target SYBR
A1		14.8	-	
A2		14.93	-	
A3		14.82	-	
A4		15.16	-	
A5		14.78	-	
A6		14.98	-	
A7		15.47	-	
A8		14.62	-	
A9			-	
A10		29.09	-	
A11		28.37	-	
A12			-	
B1		37.55	-	
B2		34.19	-	
B3		34.6	-	
B4		35.63	-	
B5		36.54	-	
B6		37.74	-	
B7		35.27	-	
B8		34	-	
B9			-	
B10		27.7	-	
B11		28.35	-	
B12			-	
C1		25.6	-	
C2		25.3	-	
C3		25.29	-	
C4		25.4	-	
C5		24.97	-	
C6		25.26	-	
C7		25.16	-	
C8		24.99	-	
C9			-	
C10		27.78	-	
C11		28.88	-	
C12			-	
D1		26.84	-	
D2		26.6	-	
D3		26.5	-	
D4		26.35	-	
D5		26.3	-	
D6		26.13	-	
D7		25.91	-	
D8		25.7	-	
D9			-	
D10		27.94	-	

D11	28.66	-
D12		-
E1	19.84	-
E2	19.93	-
E3	19.98	-
E4	20.1	-
E5	16.37	-
E6	16.54	-
E7	16.27	-
E8	16.42	-
E9		-
E10	27.86	-
E11	29.49	-
E12		-
F1	33.28	-
F2	36.05	-
F3	31	-
F4	37.26	-
F5	35.32	-
F6	33.67	-
F7	30.99	-
F8	36.41	-
F9		-
F10	27.82	-
F11	30.34	-
F12		-
G1	28.73	-
G2	28.93	-
G3	27.71	-
G4	29.71	-
G5	27.89	-
G6	28.16	-
G7	28.09	-
G8	28.21	-
G9		-
G10	27.83	-
G11	29.95	-
G12		-
H1	27.5	-
H2	27.08	-
H3	27.33	-
H4	27.45	-
H5	28.79	-
H6	29.3	-
H7	28.42	-
H8	28.97	-
H9		-

H10	27.43	-
H11	29.07	-
H12		-

Threshold detection parameters:
  Thresh(93 (Noiseband)
  Baselirautomatic
  Drift (OFF

Pos	Name	Ct SYBR	Amount SYTarget SYBR
A1		12.06	-
A2		11.94	-
A3		11.91	-
A4		11.95	-
A5		12.74	-
A6		12.6	-
A7		12.4	-
A8		11.54	-
A9		11.37	-
A10		12.55	-
A11		11.39	-
A12		11.04	-
B1		33.97	-
B2		34.63	-
B3		33.57	-
B4		32.98	-
B5		34.81	-
B6		33.77	-
B7		35.1	-
B8		34.61	-
B9		34.54	-
B10		35.24	-
B11		32.62	-
B12		34.55	-
C1		17.39	-
C2		17.23	-
C3		17.03	-
C4		17.05	-
C5		17.9	-
C6		17.6	-
C7		17.6	-
C8		17.53	-
C9		21.65	-
C10		21.34	-
C11		21.64	-
C12		22.25	-
D1		25.45	-
D2		25.56	-
D3		25.54	-
D4		25.4	-
D5		26.32	-
D6		26.27	-
D7		25.42	-
D8		25.77	-
D9		30.4	-
D10		30.28	-

D11	30.36	-
D12	29.26	-
E1	30.87	-
E2	32.62	-
E3	31.9	-
E4	35.93	-
E5	32.12	-
E6	33.44	-
E7	32.13	-
E8	32.6	-
E9	32.44	-
E10	33.2	-
E11	37.22	-
E12	32.66	-
F1		-
F2		-
F3		-
F4		-
F5		-
F6		-
F7		-
F8		-
F9		-
F10		-
F11		-
F12		-
G1		-
G2		-
G3		-
G4		-
G5		-
G6		-
G7		-
G8		-
G9		-
G10		-
G11		-
G12		-
H1		-
H2		-
H3		-
H4		-
H5		-
H6		-
H7		-
H8		-
H9		-

H10	-
H11	-
H12	-

Threshold detection parameters:

Thresh	77 (Noiseband)
Baseli	automatic
Drift (	OFF

Pos	Name	Ct SYBR	Amount S'	Target SYBR
A1		14.23	-	
A2		14.36	-	
A3		14.29	-	
A4		14.15	-	
A5		20.45	-	
A6		20.29	-	
A7		20.4	-	
A8		20.28	-	
A9		15.36	-	
A10		16.63	-	
A11		15.12	-	
A12		15.03	-	
B1		18.81	-	
B2		18.75	-	
B3		17.56	-	
B4		18.64	-	
B5			-	
B6		25.6	-	
B7		25.79	-	
B8		25.4	-	
B9		19.83	-	
B10		19.88	-	
B11		19.62	-	
B12		19.52	-	
C1		23.05	-	
C2		23.39	-	
C3		23.42	-	
C4		23.2	-	
C5		30.56	-	
C6		31.06	-	
C7		30	-	
C8		30.51	-	
C9		25.06	-	
C10		24.78	-	
C11		25.11	-	
C12		25.11	-	
D1		14.35	-	
D2		14.35	-	
D3		14.1	-	
D4		14.06	-	
D5		14.62	-	
D6		14.65	-	
D7		14.89	-	
D8		14.58	-	
D9			-	
D10			-	

D11		-
D12		-
E1	26.98	-
E2	27.03	-
E3	27.05	-
E4	27.11	-
E5	25.11	-
E6	25.57	-
E7	25.18	-
E8	25.08	-
E9		-
E10		-
E11		-
E12		-
F1	42.25	-
F2	30.87	-
F3	31.59	-
F4	30.89	-
F5	27.19	-
F6	27.31	-
F7	27.4	-
F8	27.19	-
F9		-
F10		-
F11		-
F12		-
G1	31.8	-
G2	31.32	-
G3	34.18	-
G4	31.51	-
G5	33.07	-
G6	32.28	-
G7	31.63	-
G8	31.49	-
G9		-
G10		-
G11		-
G12		-
H1	31.5	-
H2	32.12	-
H3	32.74	-
H4	31.48	-
H5	28	-
H6	28.35	-
H7	27.76	-
H8	27.84	-
H9		-

H10	-
H11	-
H12	-

Threshold detection parameters:

Thresh‹85 (Noiseband)

Baseli‹automatic

Drift ‹OFF

Pos	Name	Ct SYBR	Amount SY	Target SYBR
A1		15.24	-	
A2		15.13	-	
A3		15.04	-	
A4		15.58	-	
A5		14.87	-	
A6		18.61	-	
A7		15.03	-	
A8		15.55	-	
A9		13.6	-	
A10		15.07	-	
A11		13.45	-	
A12		13.37	-	
B1		33.13	-	
B2		30.42	-	
B3		32.15	-	
B4		31.62	-	
B5		31.69	-	
B6		31.98	-	
B7		30.94	-	
B8		31.15	-	
B9		34.04	-	
B10		31.05	-	
B11		33.88	-	
B12		32.01	-	
C1		26.3	-	
C2		26.81	-	
C3		26.59	-	
C4		26.68	-	
C5		26.06	-	
C6		25.52	-	
C7		25.25	-	
C8		25.43	-	
C9		31.48	-	
C10		31.52	-	
C11		31.25	-	
C12		31.66	-	
D1			-	
D2		33.7	-	
D3			-	
D4		4.95	-	
D5			-	
D6		33.57	-	
D7		35.38	-	
D8			-	
D9			-	
D10			-	

D11		-
D12	34.77	-
E1	40.19	-
E2	42.54	-
E3	41.57	-
E4		-
E5	40.9	-
E6		-
E7	44.71	-
E8		-
E9	40.9	-
E10		-
E11		-
E12		-
F1	28.11	-
F2	28.08	-
F3	27.41	-
F4	27.84	-
F5	27.74	-
F6	28.05	-
F7	27.8	-
F8	27.91	-
F9	33.25	-
F10	33.8	-
F11	33	-
F12	31.51	-
G1	31.29	-
G2	31.6	-
G3	33.04	-
G4	32.54	-
G5	29.64	-
G6	29.05	-
G7	30.05	-
G8	29.51	-
G9	42.45	-
G10	35.95	-
G11		-
G12	40.72	-
H1		-
H2		-
H3		-
H4		-
H5		-
H6		-
H7		-
H8		-
H9		-

H10	-
H11	-
H12	-

Threshold detection parameters:

Thresh‹89 (Noiseband)

Baselir automatic

Drift (OFF

Pos	Name	Ct SYBR	Amount S'	Target SYBR
A1		17.93	-	
A2		17.72	-	
A3		17.83	-	
A4		18.22	-	
A5		17.02	-	
A6		17.2	-	
A7		17.34	-	
A8		16.96	-	
A9		20.3	-	
A10		21.54	-	
A11		19.84	-	
A12		19.82	-	
B1		22.49	-	
B2		22.59	-	
B3		22.51	-	
B4		22.34	-	
B5		23.18	-	
B6		23.02	-	
B7		22.73	-	
B8		23.16	-	
B9		24.78	-	
B10		22.6	-	
B11		24.83	-	
B12		24.67	-	
C1		36.11	-	
C2			-	
C3			-	
C4			-	
C5		37.41	-	
C6		39.05	-	
C7		35.85	-	
C8			-	
C9		33.77	-	
C10		35.89	-	
C11			-	
C12		35.07	-	
D1		28.23	-	
D2		28.1	-	
D3		28.16	-	
D4		27.96	-	
D5		27.63	-	
D6		27.68	-	
D7		27.35	-	
D8		27.78	-	
D9		28.99	-	
D10		28.96	-	

D11	29.45	-
D12	29.74	-
E1	22.27	-
E2	22.21	-
E3	22.2	-
E4	22.31	-
E5	22.94	-
E6	22.86	-
E7	23.33	-
E8	22.83	-
E9	24.61	-
E10	24.6	-
E11		-
E12		-
F1	24.63	-
F2	24.31	-
F3	24.39	-
F4	24.68	-
F5	24.87	-
F6		-
F7	24.67	-
F8	24.91	-
F9	24.92	-
F10	24.85	-
F11	24.81	-
F12	24.78	-
G1		-
G2		-
G3		-
G4		-
G5	4.35	-
G6		-
G7		-
G8		-
G9		-
G10		-
G11		-
G12		-
H1	28.49	-
H2	26.57	-
H3	26.27	-
H4	26.66	-
H5	25.53	-
H6	25.59	-
H7	25.72	-
H8	25.99	-
H9	27.99	-

H10	28.09	-
H11	28.46	-
H12	27.8	-

Threshold detection parameters:
  Thresh(123 (Noiseband)
  Baseliiautomatic
  Drift (OFF

Pos	Name	Ct SYBR	Amount SYTarget SYBR
A1		18.35	-
A2		17.91	-
A3		18.23	-
A4		18.27	-
A5		16.84	-
A6		17.1	-
A7		16.8	-
A8		16.56	-
A9		20.07	-
A10		21.23	-
A11		19.79	-
A12		19.82	-
B1		39.57	-
B2		38.9	-
B3			-
B4		33	-
B5			-
B6			-
B7			-
B8			-
B9			-
B10			-
B11			-
B12			-
C1			-
C2		38.09	-
C3			-
C4			-
C5		34.44	-
C6			-
C7			-
C8			-
C9			-
C10			-
C11			-
C12		43.05	-
D1		24.47	-

D2	24.45	-
D3	24.6	-
D4	24.54	-
D5	23.4	-
D6	23.69	-
D7	23.29	-
D8	23.67	-
D9	24.83	-
D10	24.91	-
D11	24.62	-
D12	24.79	-
E1		-
E2	3.27	-
E3		-
E4		-
E5		-
E6	3.83	-
E7		-
E8		-
E9		-
E10		-
E11		-
E12	3.82	-
F1	29.45	-
F2	38.65	-
F3	33.59	-
F4	34.92	-
F5	34.5	-
F6	36.18	-
F7	36.52	-
F8	34.58	-
F9	35.8	-
F10	33.03	-
F11	34.88	-
F12	35.85	-
G1	23.91	-
G2	24.01	-
G3	24.15	-
G4	23.97	-
G5	22.6	-
G6	22.56	-
G7	22.75	-
G8	22.62	-
G9	25.65	-
G10	25.78	-
G11	25.27	-
G12	25.44	-

H1	21.78	-
H2	21.81	-
H3	21.89	-
H4	22.07	-
H5	21.37	-
H6	21.37	-
H7	21.01	-
H8	21.16	-
H9	23.25	-
H10	23.07	-
H11	22.81	-
H12	22.92	-

Threshold detection parameters:
  Thresh(112 (Noiseband)
  Baselir automatic
  Drift (OFF

Pos	Name	Ct SYBR	Amount S\	Target SYBR
A1		16.24	-	
A2		16.12	-	
A3		15.89	-	
A4		16.31	-	
A5		16.54	-	
A6		17.27	-	
A7		16.7	-	
A8		16.71	-	
A9		19.35	-	
A10		21.05	-	
A11		19.02	-	
A12		19.18	-	
B1			-	
B2		0.76	-	
B3			-	
B4			-	
B5			-	
B6			-	
B7		0.24	-	
B8			-	
B9		0.08	-	
B10			-	
B11			-	
B12			-	
C1		33.42	-	
C2		34.83	-	
C3		32.41	-	
C4		32.49	-	

C5	34.51	-
C6	38.4	-
C7	34	-
C8	33.98	-
C9	34.27	-
C10	37.59	-
C11	35.69	-
C12	33.95	-
D1		-
D2		-
D3		-
D4		-
D5		-
D6	1.56	-
D7		-
D8		-
D9		-
D10		-
D11		-
D12		-
E1	28.86	-
E2	29.42	-
E3	29.14	-
E4	29.54	-
E5	35.17	-
E6	30.96	-
E7	32.46	-
E8	28.49	-
E9	31.46	-
E10	32.06	-
E11	32.93	-
E12	31.21	-
F1		-
F2		-
F3		-
F4		-
F5		-
F6		-
F7		-
F8		-
F9		-
F10		-
F11		-
F12		-
G1		-
G2		-
G3		-

G4	-
G5	-
G6	-
G7	-
G8	-
G9	-
G10	-
G11	-
G12	-
H1	-
H2	-
H3	-
H4	-
H5	-
H6	-
H7	-
H8	-
H9	-
H10	-
H11	-
H12	-

Threshold detection parameters:

Thresh(	86 (Noiseband)
Baselii	automatic
Drift (	OFF

Pos	Name	Ct SYBR	Amount S'	Target SYBR
A1		17.44	-	
A2		16.83	-	
A3		17.41	-	
A4		17.57	-	
A5		17.17	-	
A6		17.95	-	
A7		17.18	-	
A8		17.26	-	
A9		20.14	-	
A10		22.22	-	
A11		20.34	-	
A12		20.15	-	
B1		20.88	-	
B2		20.72	-	
B3		20.34	-	
B4		17.27	-	
B5		21.68	-	
B6		20.98	-	
B7		24.26	-	

B8	19.9	-
B9	21.42	-
B10	23.23	-
B11	22.46	-
B12	23.31	-
C1	31.53	-
C2	30.88	-
C3	32.59	-
C4		-
C5	44.99	-
C6	34.15	-
C7	34.01	-
C8	33.02	-
C9		-
C10		-
C11		-
C12		-
D1	26.12	-
D2	25.98	-
D3	25.89	-
D4	26.06	-
D5	29.19	-
D6	29.24	-
D7	29.56	-
D8	29.02	-
D9	31.18	-
D10	31.62	-
D11	31.17	-
D12	31.18	-
E1	23.31	-
E2	23.28	-
E3	23.39	-
E4	23.75	-
E5	23.98	-
E6	23.78	-
E7	23.76	-
E8	23.77	-
E9	25.09	-
E10	25.03	-
E11	24.94	-
E12		-
F1	26.8	-
F2	26.93	-
F3	26.82	-
F4	26.74	-
F5	28.73	-
F6	28.5	-

F7	28.67	-
F8	28.24	-
F9	30.02	-
F10	29.61	-
F11	29.98	-
F12	30.55	-
G1		-
G2		-
G3		-
G4		-
G5		-
G6		-
G7		-
G8		-
G9	41.17	-
G10		-
G11	33.86	-
G12	38.37	-
H1	22.62	-
H2	22.49	-
H3	22.75	-
H4	23.26	-
H5	22.76	-
H6	22.99	-
H7	22.65	-
H8	22.84	-
H9	26.07	-
H10	26.03	-
H11	25.74	-
H12	25.94	-

Threshold detection parameters:
Thresh(154 (Noiseband)
Baselii automatic
Drift (OFF

Pos	Name	Ct SYBR	Amount S\	Target SYBR
A1		15.46	-	
A2		15.1	-	
A3		15.53	-	
A4		15.58	-	
A5		15.5	-	
A6		16.16	-	
A7		15.86	-	
A8		16.04	-	
A9			-	
A10			-	

A11		-
A12		-
B1	19.85	-
B2	19.73	-
B3	19.6	-
B4	19.39	-
B5	22.26	-
B6	22.06	-
B7	22.17	-
B8	22.66	-
B9		-
B10		-
B11		-
B12		-
C1	25.57	-
C2	25.68	-
C3	25.76	-
C4	25.75	-
C5	26.74	-
C6	27.5	-
C7	26.93	-
C8	27.32	-
C9		-
C10		-
C11		-
C12		-
D1	18.64	-
D2	18.62	-
D3	18.45	-
D4	18.89	-
D5	35.17	-
D6	19.9	-
D7	19.63	-
D8	19.73	-
D9		-
D10		-
D11		-
D12		-
E1	21.4	-
E2	21.6	-
E3	21.58	-
E4	21.45	-
E5	22.29	-
E6	22.14	-
E7	21.98	-
E8	21.81	-
E9		-

E10		-
E11		-
E12		-
F1	22.98	-
F2	22.56	-
F3	22.76	-
F4	22.9	-
F5	24.23	-
F6	24.33	-
F7	24.25	-
F8	23.89	-
F9		-
F10		-
F11		-
F12		-
G1		-
G2		-
G3		-
G4		-
G5		-
G6		-
G7		-
G8		-
G9		-
G10		-
G11		-
G12		-
H1		-
H2		-
H3		-
H4		-
H5		-
H6		-
H7		-
H8		-
H9		-
H10		-
H11		-
H12		-

Threshold detection parameters:
Thresh(83 (Noiseband)
Baselir automatic
Drift (OFF

Pos	Name	Ct SYBR	Amount S\	Target SYBR
A1		16.93	-	

A2	16.71	-
A3	16.64	-
A4	16.89	-
A5	23.91	-
A6	24.06	-
A7	23.77	-
A8	23.66	-
A9	26.01	-
A10	27.06	-
A11	25.82	-
A12	25.5	-
B1	17.16	-
B2	17.28	-
B3	16.71	-
B4	17.08	-
B5	24.86	-
B6	29.83	-
B7	24.81	-
B8	24.46	-
B9	21.45	-
B10	21.65	-
B11	21.27	-
B12	20.85	-
C1	16.79	-
C2	16.68	-
C3	16.84	-
C4	17.1	-
C5	38.21	-
C6		-
C7		-
C8	34.04	-
C9	33.55	-
C10	43.9	-
C11	39.54	-
C12		-
D1	16.01	-
D2	16.13	-
D3	16.15	-
D4	16.23	-
D5	24.57	-
D6	27.92	-
D7	24.58	-
D8	24.17	-
D9	23.28	-
D10	22.86	-
D11	22.69	-
D12	23.04	-

E1	16.91	-
E2	16.61	-
E3	16.99	-
E4	17.39	-
E5		-
E6		-
E7		-
E8		-
E9		-
E10		-
E11		-
E12		-
F1		-
F2		-
F3		-
F4		-
F5		-
F6		-
F7		-
F8		-
F9		-
F10		-
F11		-
F12		-
G1		-
G2		-
G3		-
G4		-
G5		-
G6		-
G7		-
G8		-
G9		-
G10		-
G11		-
G12		-
H1		-
H2		-
H3		-
H4		-
H5		-
H6		-
H7		-
H8		-
H9		-
H10		-
H11		-

H12 -

Threshold detection parameters:
  Thresh 68 (Noiseband)
  Baselir automatic
  Drift (OFF

Pos	Name	Ct SYBR	Amount S'	Target SYBR
A1		16.94	-	
A2		16.77	-	
A3		16.77	-	
A4		16.97	-	
A5		22.46	-	
A6		22.73	-	
A7		22.71	-	
A8		22.66	-	
A9		23.79	-	
A10		24.83	-	
A11		23.57	-	
A12		23.67	-	
B1		17	-	
B2		17.47	-	
B3		17.16	-	
B4		17.8	-	
B5		23.08	-	
B6		23.21	-	
B7		23.17	-	
B8		23.39	-	
B9		24.95	-	
B10		24.88	-	
B11		24.51	-	
B12		24.64	-	
C1		16.8	-	
C2		16.78	-	
C3		17.01	-	
C4		18.5	-	
C5		22.82	-	
C6		22.92	-	
C7		31.8	-	
C8		23.49	-	
C9		31.9	-	
C10		31.56	-	
C11		33.35	-	
C12		32.51	-	
D1		16.35	-	
D2		16.47	-	
D3		16.38	-	

D4	16.41	-
D5	23.25	-
D6	22.63	-
D7	23.26	-
D8	23.09	-
D9	25.05	-
D10	24.81	-
D11	24.66	-
D12	24.89	-
E1	17.2	-
E2	16.99	-
E3	17.23	-
E4	17.41	-
E5	22.85	-
E6	23.12	-
E7	22.78	-
E8	23.2	-
E9	25.37	-
E10	25.27	-
E11	25.91	-
E12	25.38	-
F1	21.74	-
F2	21.56	-
F3	21.62	-
F4	21.79	-
F5	32	-
F6	34.23	-
F7	33.62	-
F8	32.55	-
F9	23.33	-
F10	23.33	-
F11	23.28	-
F12	23.34	-
G1		-
G2		-
G3		-
G4		-
G5		-
G6		-
G7		-
G8		-
G9		-
G10		-
G11		-
G12		-
H1		-
H2		-

H3			-	
H4			-	
H5			-	
H6			-	
H7			-	
H8			-	
H9			-	
H10			-	
H11			-	
H12			-	

Threshold detection parameters:
  Thresh(98 (Noiseband)
  Baselii automatic
  Drift (OFF

Pos	Name	Ct SYBR	Amount S\	Target SYBR
A1		16.99	-	
A2		20.51	-	
A3		17.59	-	
A4		32.1	-	
A5		17	-	
A6		21.95	-	
A7		17.15	-	
A8		33.63	-	
A9		22.54	-	
A10			-	
A11		15.42	-	
A12		19.38	-	
B1		16.7	-	
B2		20.81	-	
B3		16.92	-	
B4		36.15	-	
B5		16.98	-	
B6		22.1	-	
B7		17.45	-	
B8		34.83	-	
B9		21.93	-	
B10			-	
B11		15.39	-	
B12		19.32	-	
C1		20.89	-	
C2		20.91	-	
C3		17.57	-	
C4		34.35	-	
C5		16.99	-	
C6		21.88	-	

C7	17.03	-
C8	39.84	-
C9	21.58	-
C10		-
C11	15.53	-
C12	19.51	-
D1	16.44	-
D2	20.08	-
D3	17.03	-
D4	35.33	-
D5	17.03	-
D6	21.66	-
D7	17.09	-
D8	34.14	-
D9	21.85	-
D10		-
D11	8.4	-
D12	4.48	-
E1	17.57	-
E2	24.02	-
E3	16.63	-
E4		-
E5	16.33	-
E6	25.53	-
E7	16.58	-
E8	32.84	-
E9	24.42	-
E10		-
E11	15.72	-
E12	21.77	-
F1	17.78	-
F2	25.73	-
F3	17.13	-
F4	33.9	-
F5	16.15	-
F6	25.13	-
F7	16.97	-
F8	31.39	-
F9	24.7	-
F10		-
F11	15.78	-
F12	22.54	-
G1	16.94	-
G2	23.85	-
G3	17.11	-
G4	38.12	-
G5	16.25	-

G6	25.46	-
G7	17.43	-
G8	34.96	-
G9	24.35	-
G10		-
G11	15.44	-
G12	21.57	-
H1	16.7	-
H2	23.88	-
H3	17.28	-
H4	38.08	-
H5	17.53	-
H6	25.29	-
H7	17.12	-
H8	8.07	-
H9		-
H10		-
H11	4.7	-
H12	21.7	-

Threshold detection parameters:
Thresh(101 (Noiseband)
Baselii automatic
Drift (OFF

Pos	Name	Ct SYBR	Amount S'	Target SYBR
A1		15.55	-	
A2		35.03	-	
A3		15.2	-	
A4		36.18	-	
A5		15.4	-	
A6		21.49	-	
A7		15.52	-	
A8		34.31	-	
A9		20.96	-	
A10			-	
A11		16.59	-	
A12		33.14	-	
B1		15.05	-	
B2		35.5	-	
B3		15.41	-	
B4		37.41	-	
B5		15.49	-	
B6		21.43	-	
B7		15.59	-	
B8		33.87	-	
B9		20.85	-	

B10		-
B11	16.13	-
B12	34.95	-
C1	15.64	-
C2		-
C3	15.55	-
C4	34.71	-
C5	15.57	-
C6	21.23	-
C7		-
C8	38.17	-
C9	21.04	-
C10		-
C11	16.6	-
C12		-
D1	15.2	-
D2	34.43	-
D3	15.72	-
D4	34.81	-
D5	15.76	-
D6	21.43	-
D7	15.76	-
D8	32.95	-
D9	20.93	-
D10		-
D11	16.45	-
D12		-
E1	15.75	-
E2	38.58	-
E3	15.84	-
E4		-
E5	16.44	-
E6	24	-
E7	16.14	-
E8	33.23	-
E9	23.54	-
E10		-
E11	16.57	-
E12	36.91	-
F1	16.15	-
F2		-
F3	16.09	-
F4	34.89	-
F5	16.75	-
F6	23.68	-
F7	16.26	-
F8	34.49	-

F9	23.5	-
F10		-
F11	16.33	-
F12		-
G1	16.23	-
G2		-
G3	16.31	-
G4	37.34	-
G5	16.79	-
G6	24.03	-
G7	15.98	-
G8	34.95	-
G9	23.72	-
G10		-
G11	16.29	-
G12		-
H1	15.58	-
H2		-
H3	16.12	-
H4	41.16	-
H5	16.13	-
H6	23.87	-
H7	16.12	-
H8	36.53	-
H9	23.18	-
H10		-
H11	15.97	-
H12	36.7	-

Threshold detection parameters:
Thresh(110 (Noiseband)
Baselir automatic
Drift (OFF

Pos	Name	Ct SYBR	Amount S'	Target SYBR
A1		15.51	-	
A2		15.37	-	
A3		15.47	-	
A4		15.58	-	
A5		25.42	-	
A6		25.59	-	
A7		24.28	-	
A8		25.44	-	
A9			-	
A10			-	
A11		35.27	-	
A12			-	

B1	15.33	-
B2	15.6	-
B3	15.4	-
B4	15.63	-
B5	25.44	-
B6	25.24	-
B7	25.2	-
B8	25.13	-
B9		-
B10		-
B11		-
B12		-
C1	15.61	-
C2	15.41	-
C3	15.53	-
C4	15.85	-
C5	25.54	-
C6	25.94	-
C7	25.64	-
C8	25.94	-
C9	42.72	-
C10		-
C11	35.38	-
C12	37.36	-
D1	16.09	-
D2	15.94	-
D3	16.01	-
D4	16.15	-
D5	26.84	-
D6	26.67	-
D7	26.66	-
D8	26.44	-
D9	42.04	-
D10		-
D11	35.64	-
D12	44.64	-
E1	15.9	-
E2	15.68	-
E3	15.89	-
E4	15.78	-
E5	26.24	-
E6	26.09	-
E7	26.39	-
E8	25.85	-
E9		-
E10	42.76	-
E11		-

E12		-
F1	17.1	-
F2	16.47	-
F3	16.87	-
F4	16.94	-
F5	21.35	-
F6	21.34	-
F7	21.41	-
F8	21.16	-
F9	36.01	-
F10		-
F11	34.31	-
F12	35.63	-
G1	17.63	-
G2	16.7	-
G3	16.86	-
G4	16.84	-
G5	21.02	-
G6	20.92	-
G7	21.03	-
G8	20.81	-
G9		-
G10		-
G11		-
G12	44.7	-
H1	17.15	-
H2	16.86	-
H3	17.2	-
H4	17.17	-
H5	15.68	-
H6	20.27	-
H7	20.16	-
H8	20.11	-
H9	35.51	-
H10		-
H11		-
H12		-

Threshold detection parameters:

Thresh(113 (Noiseband)

Baselii automatic

Drift (OFF
