# Supplementary material for: CpG-binding protein CFP1 promotes ovarian cancer cell proliferation by regulating BST2 transcription
Source: Cancer Gene Ther. 2022 Jul 21;29(12):1895–907. doi: 10.1038/s41417-022-00503-z (PMC9750859; doi:10.1038/s41417-022-00503-z)

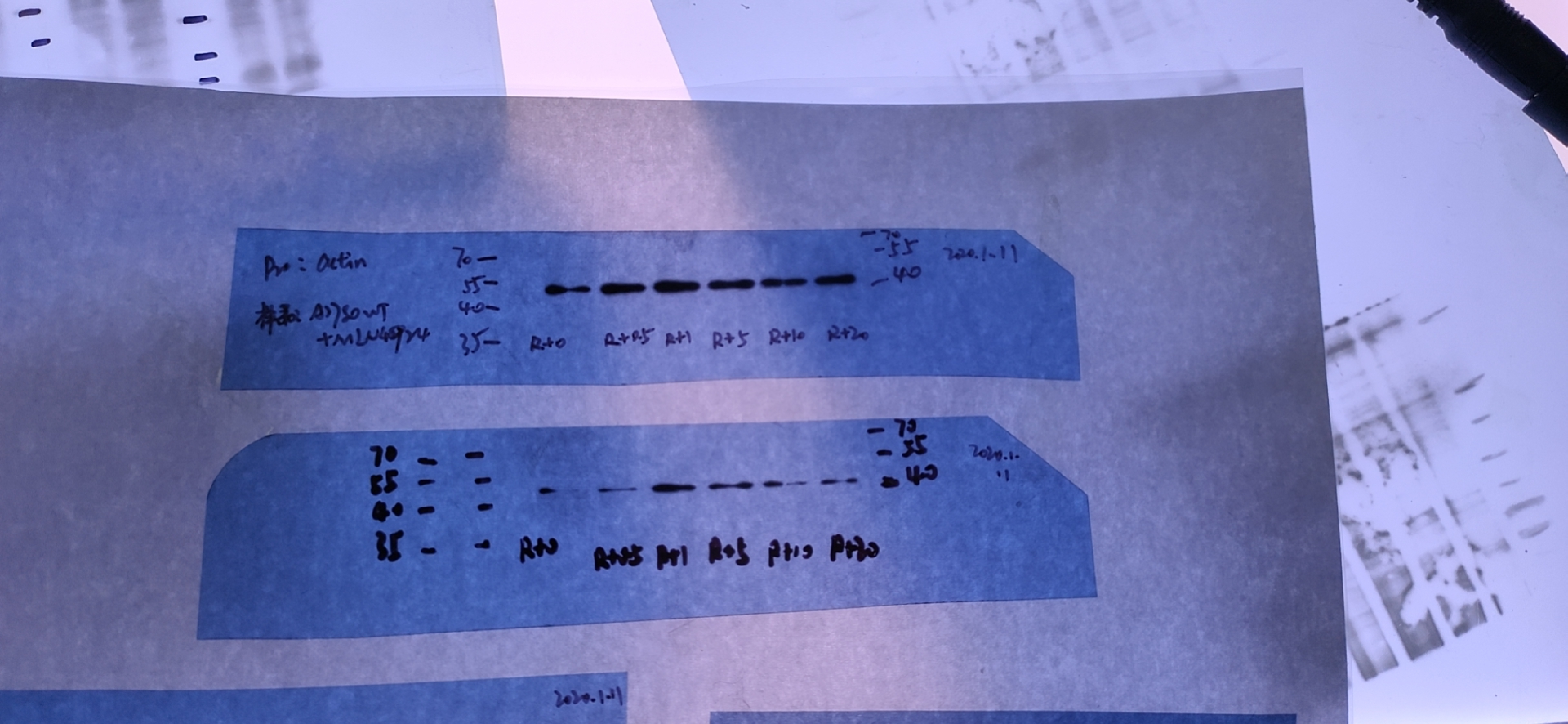

Pro: Actin
70—
55—
40—
35—
R+0
R+1
R+5
R+10
R+20
2020.1.11

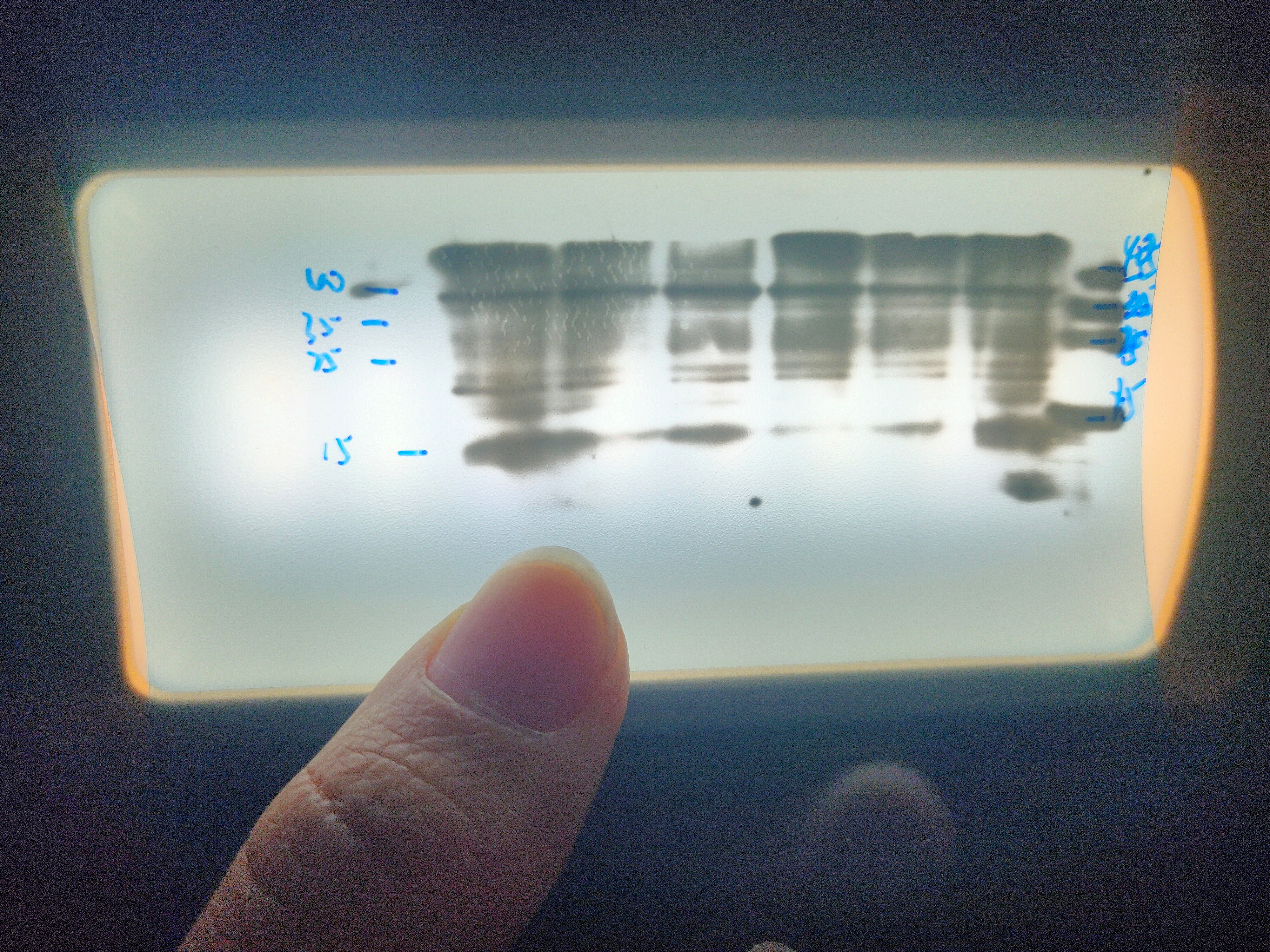
35
25
15

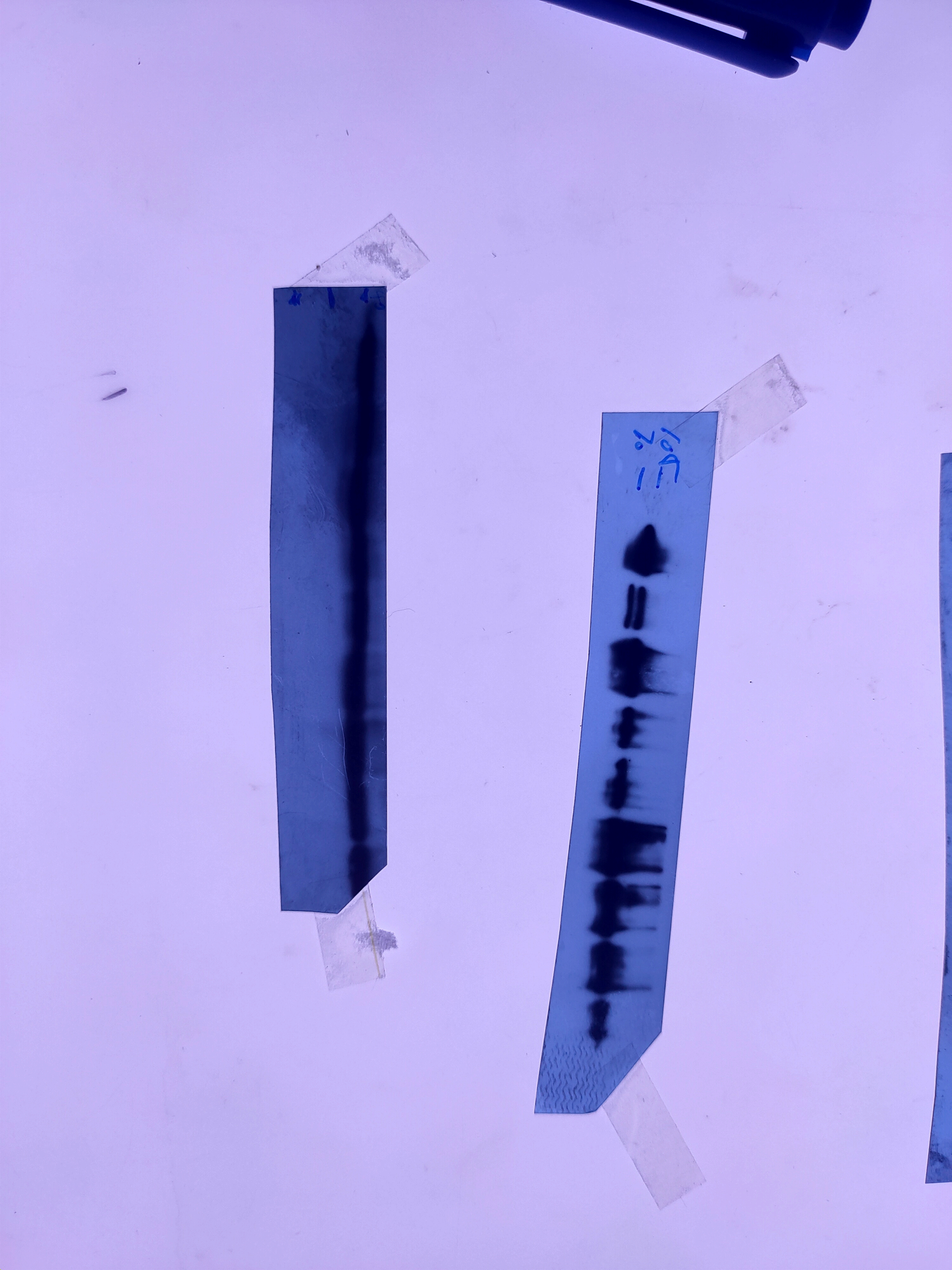

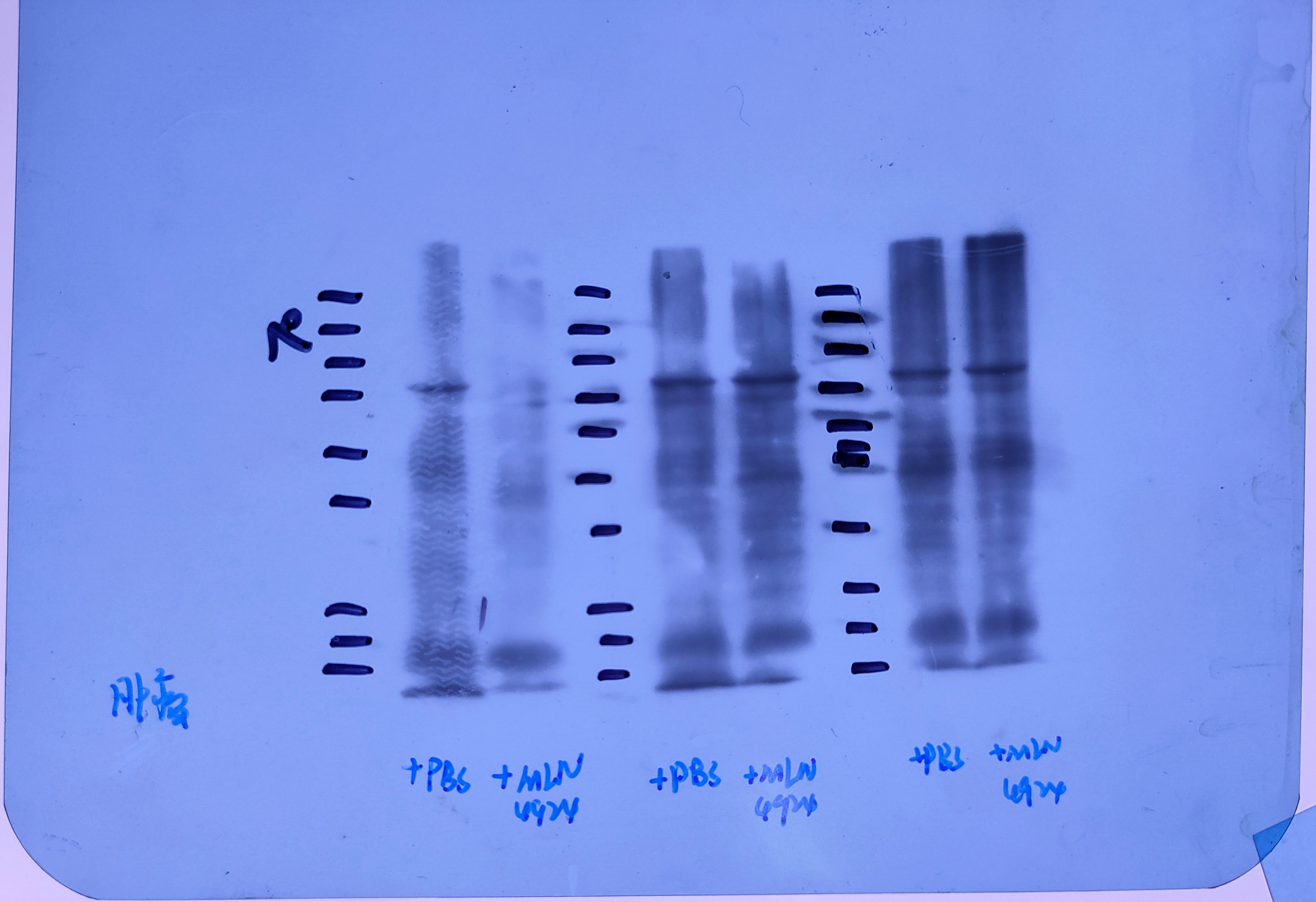
+PBS
+MLN 4924
+PBS
+MLN 4924
+PBS
+MLN 4924

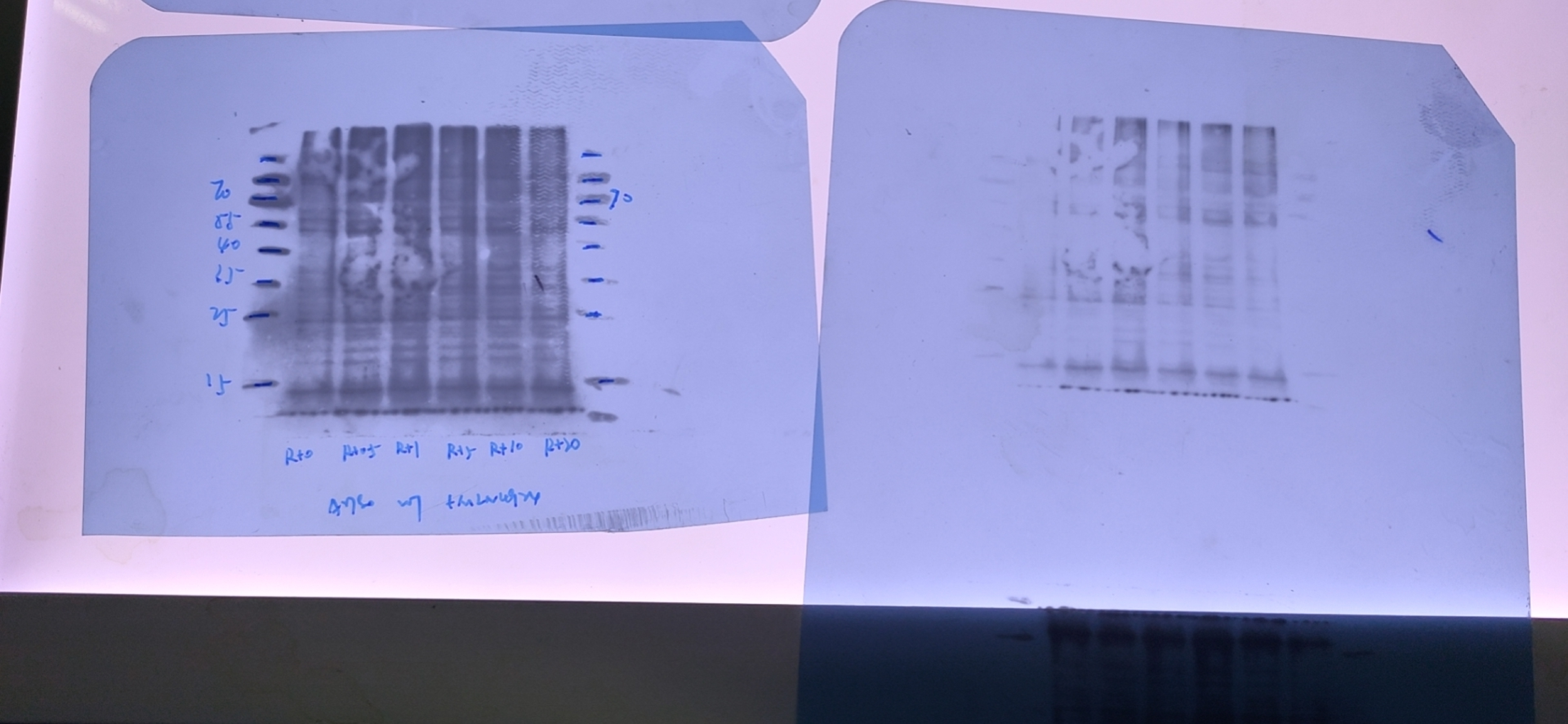
70
55
40
35
25
15
70
R+0
R+1
R+5
R+10
R+20

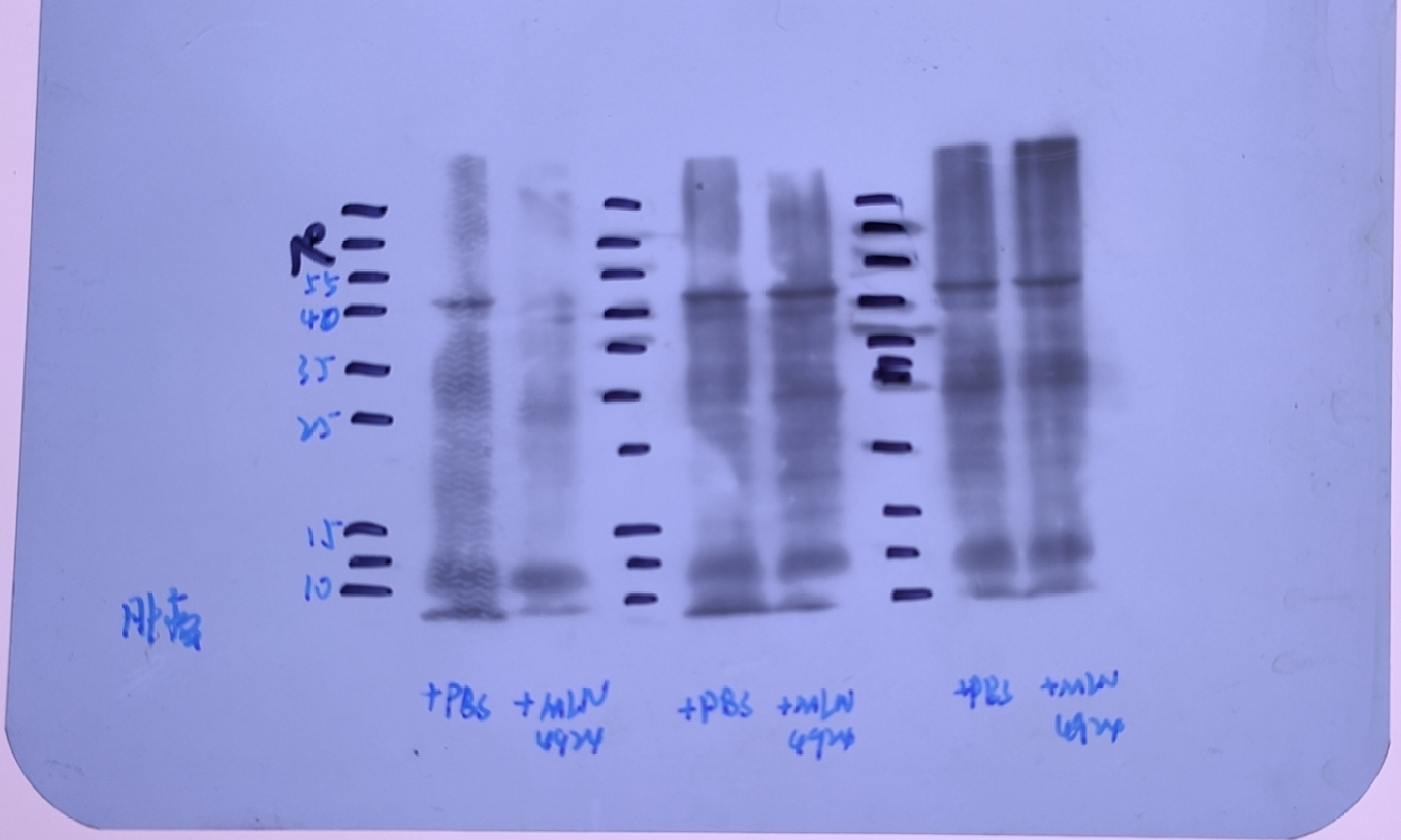
55
40
35
25
15
10
+PBS
+MLN 4924
+PBS
+MLN 4924
+PBS
+MLN 4924

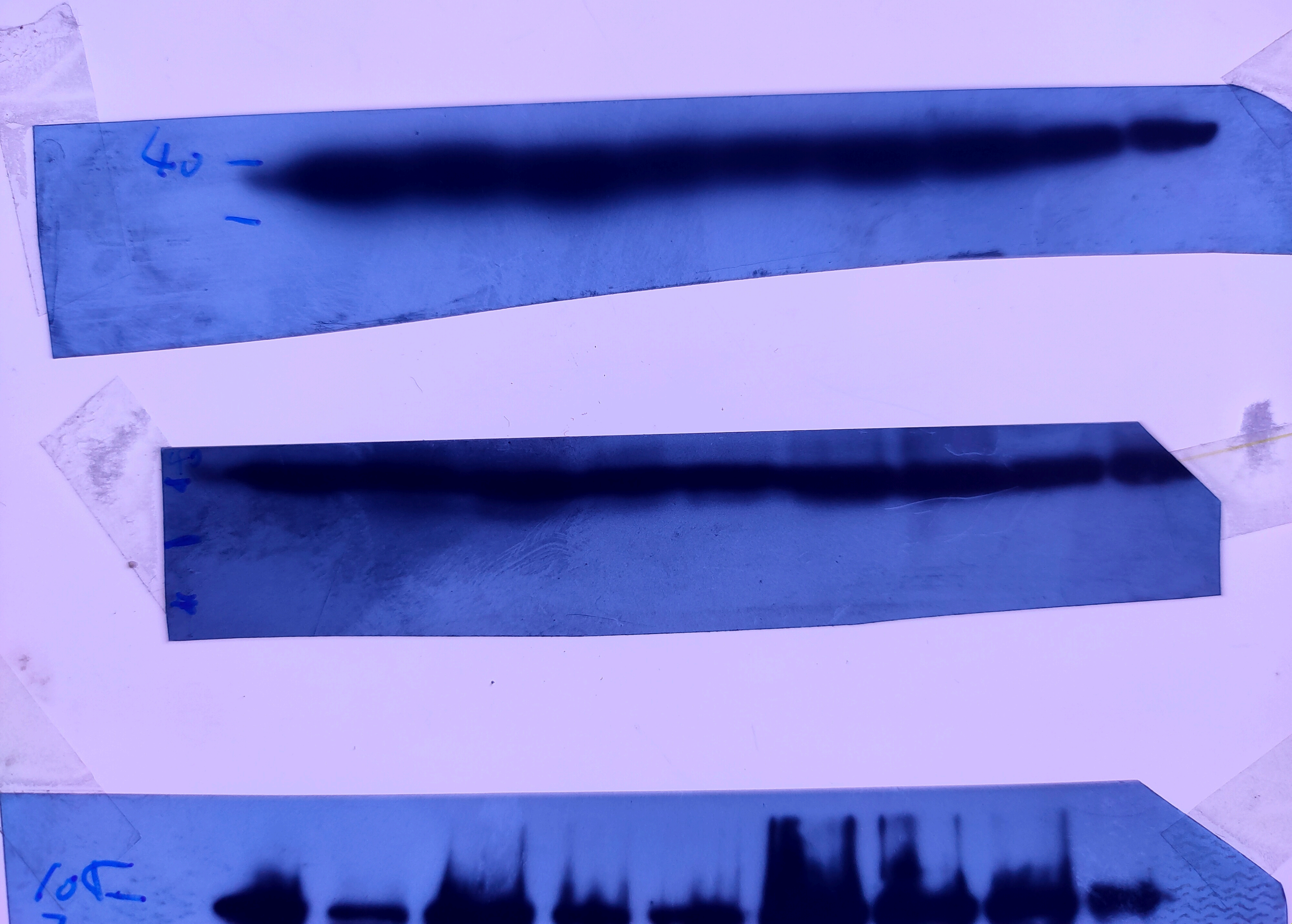
40

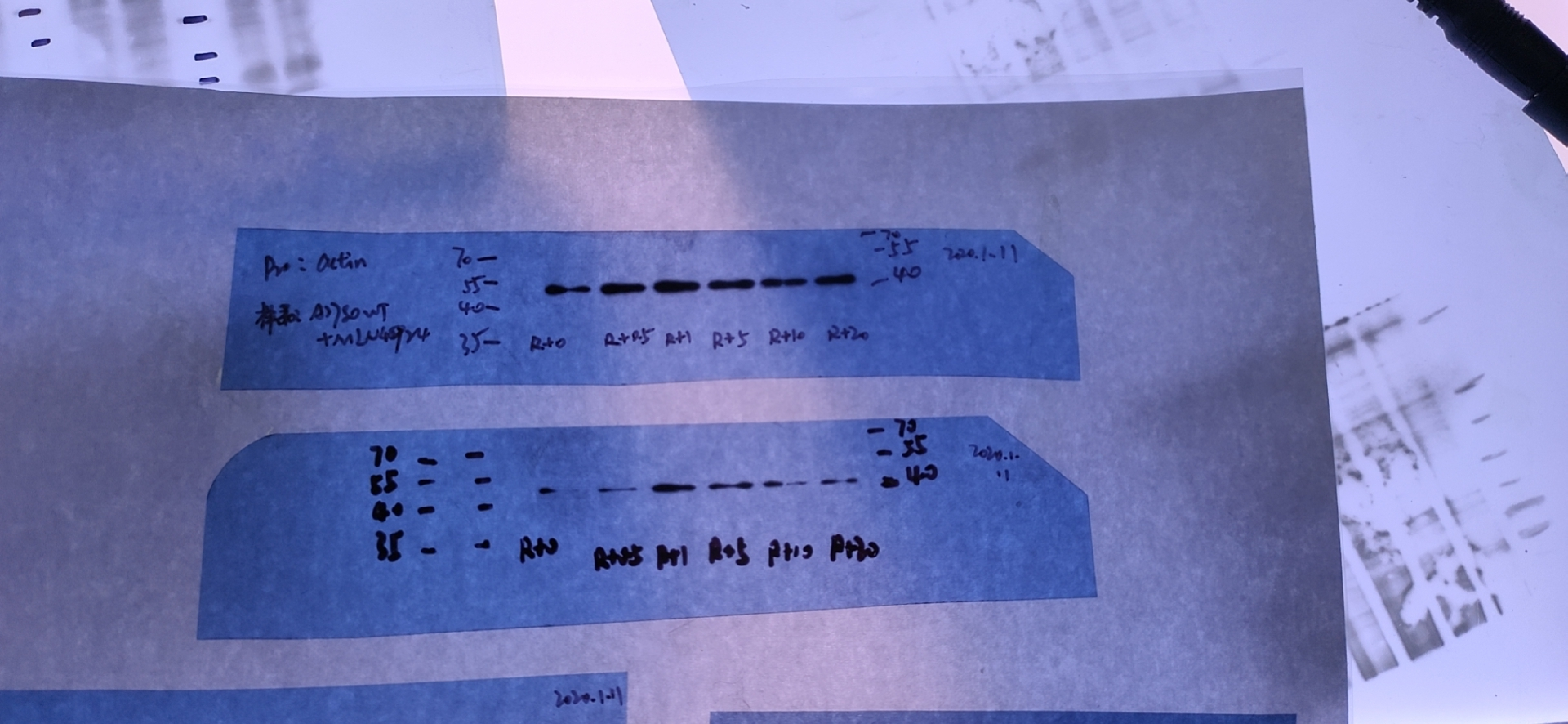

Pro: Actin
70—
55—
40—
35—
R+0
R+1
R+5
R+10
R+20
2020.1.11

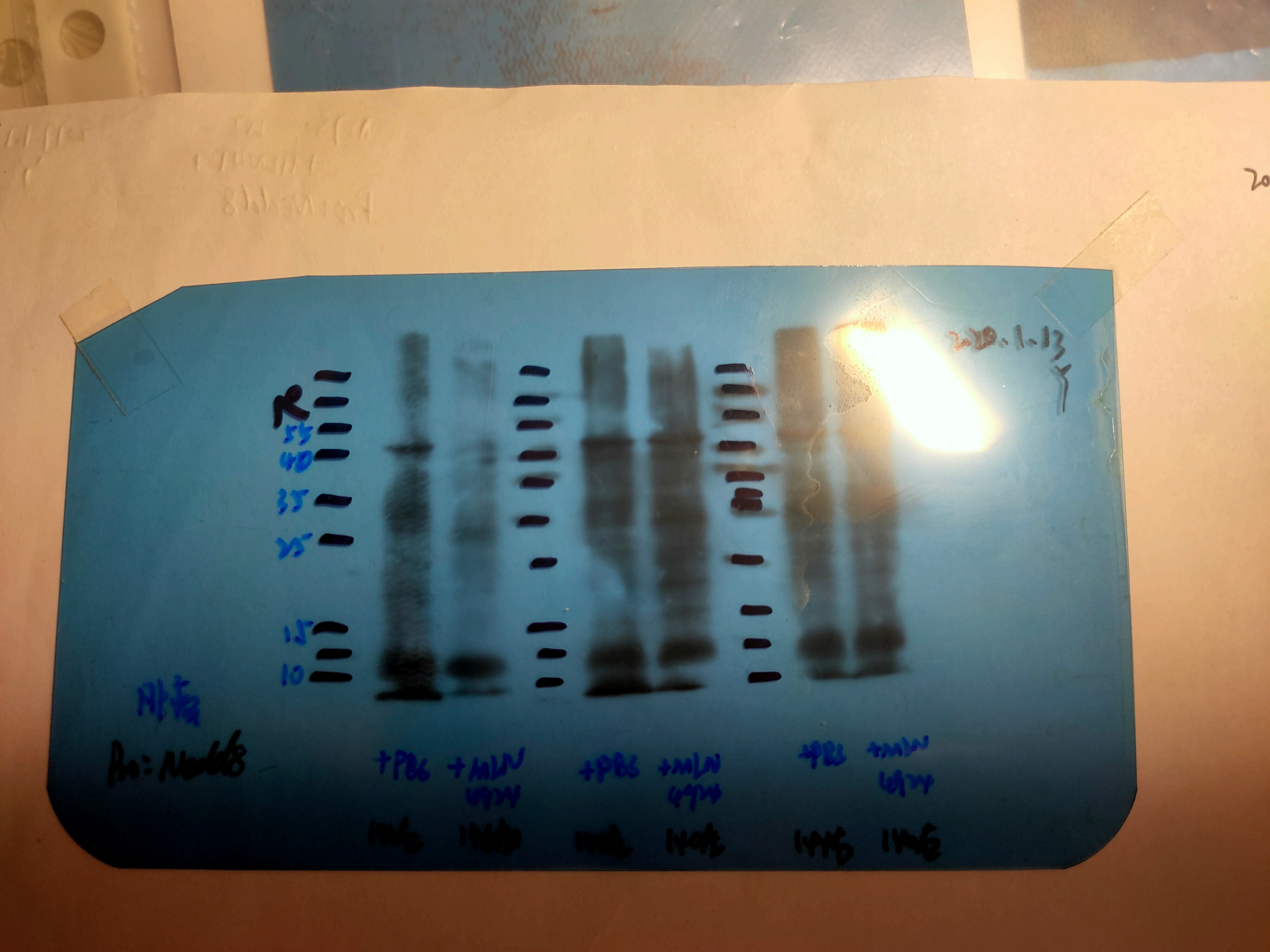

2020.1.13
55
40
35
25
15
10
+PBS
+MLN 4924
+PBS
+MLN 4924
+PBS
+MLN 4924

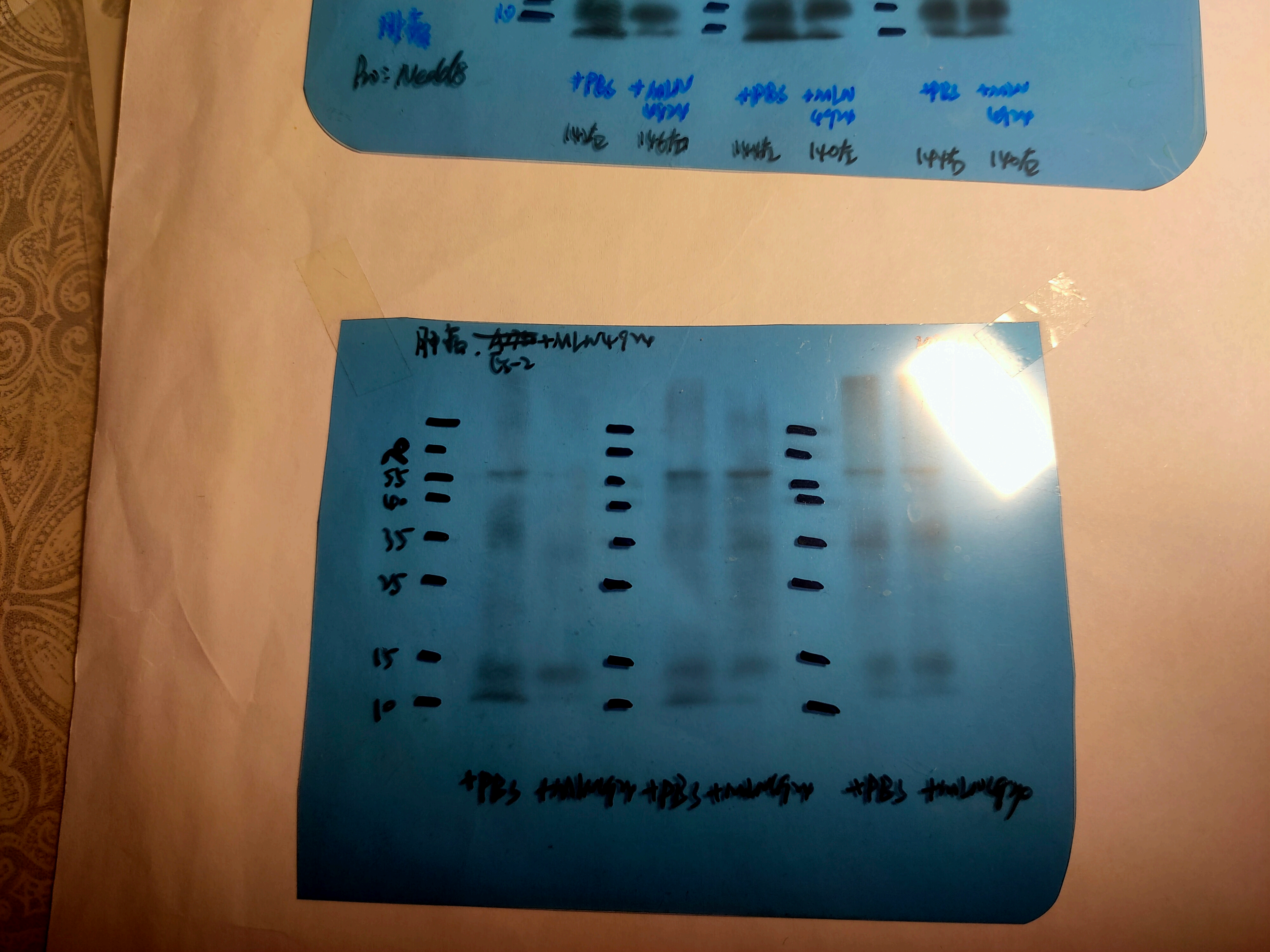

Pro: Nedd8
+PBS
+MLN 4924
+PBS
+MLN 4924
+PBS
+MLN 4924
70
55
40
35
25
15
10
+PBS
+MLN4924
+PBS
+MLN4924
+PBS
+MLN4924

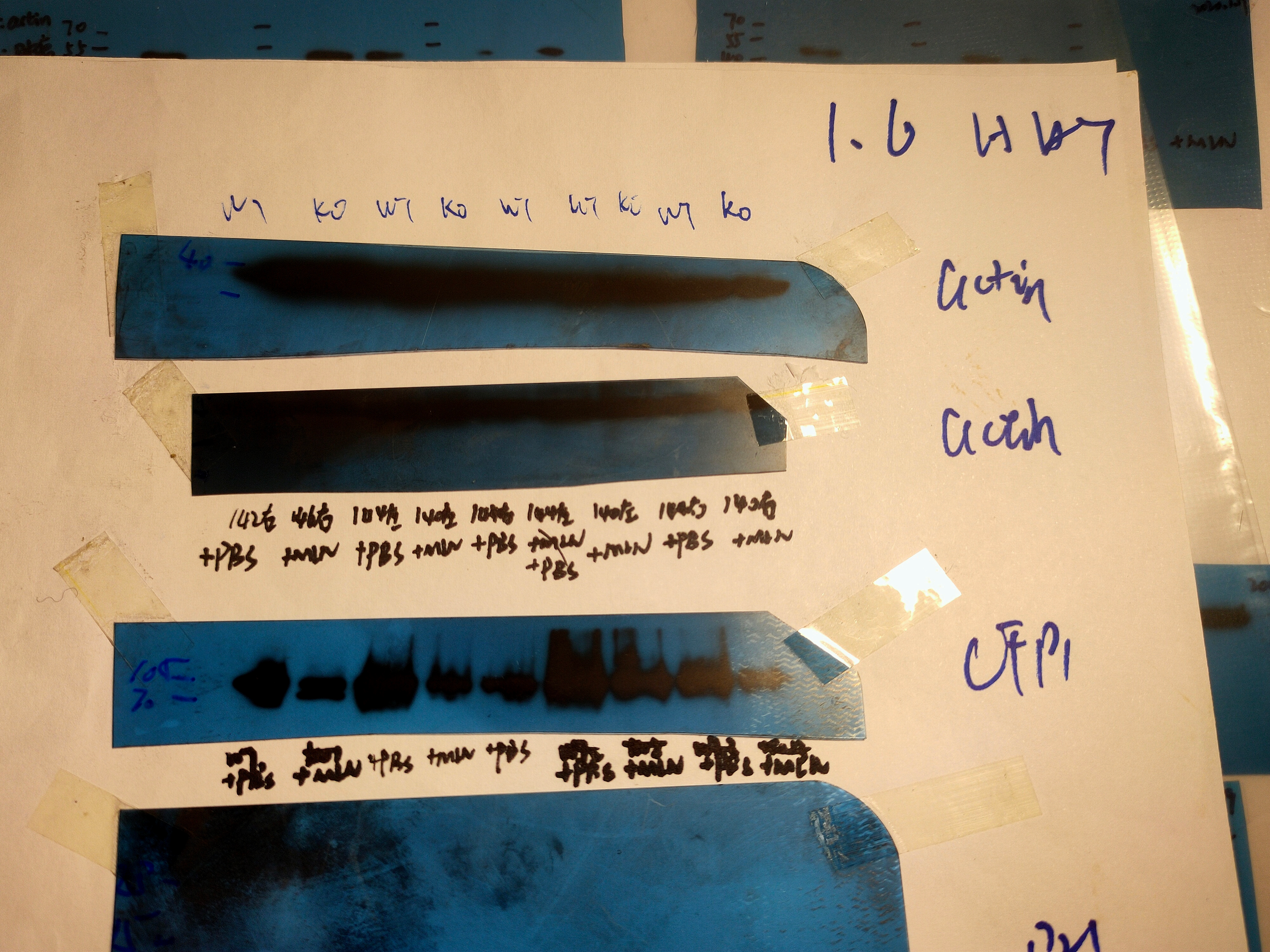
WT KO WT KO WT WT KO WT KO
40
Actin
Actin
+PBS +MLN +PBS +MLN +PBS +PBS +MLN +PBS +MLN
105
70
CFP1

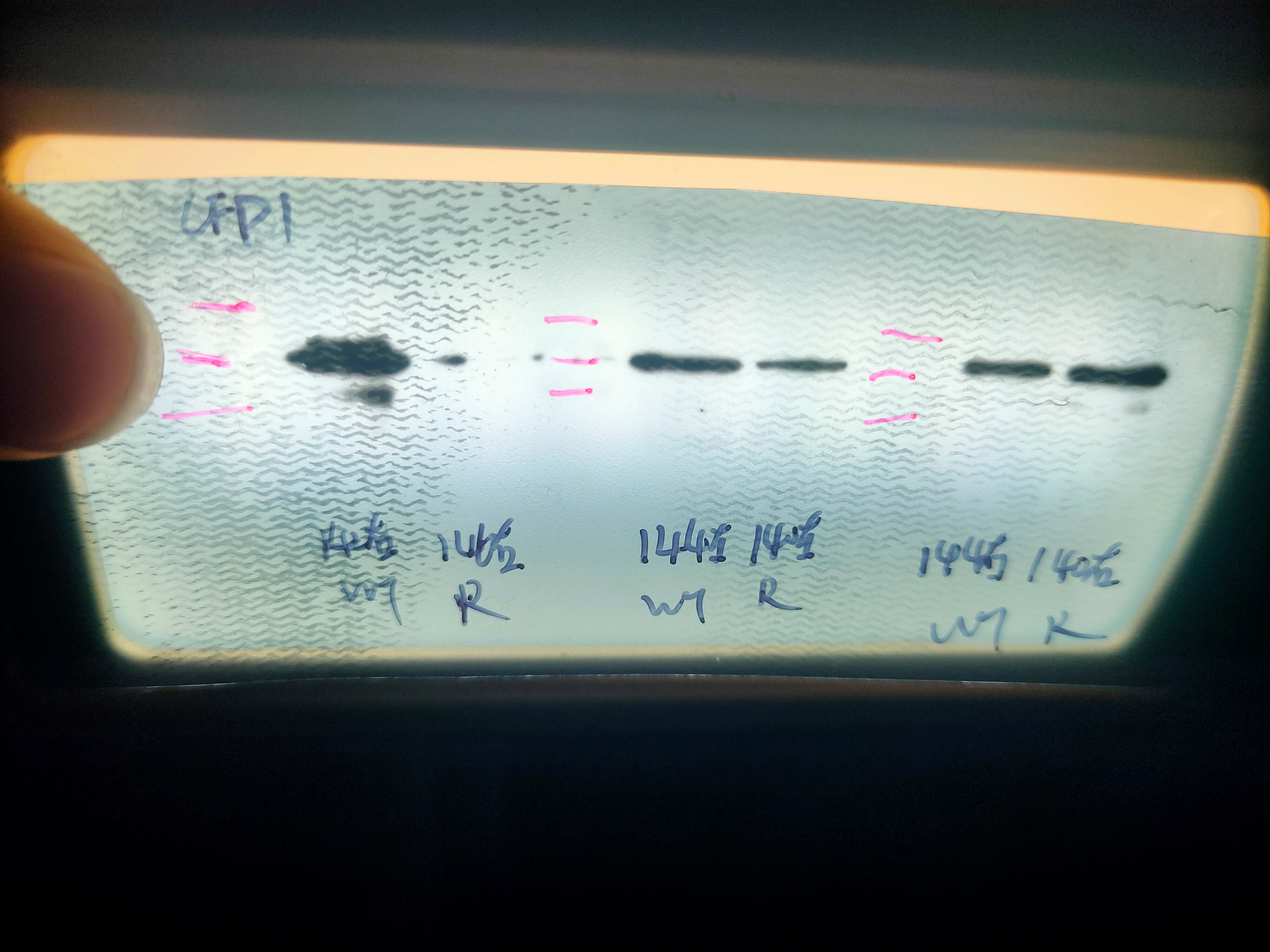
UFD1
WT
R
WT
R
WT
K

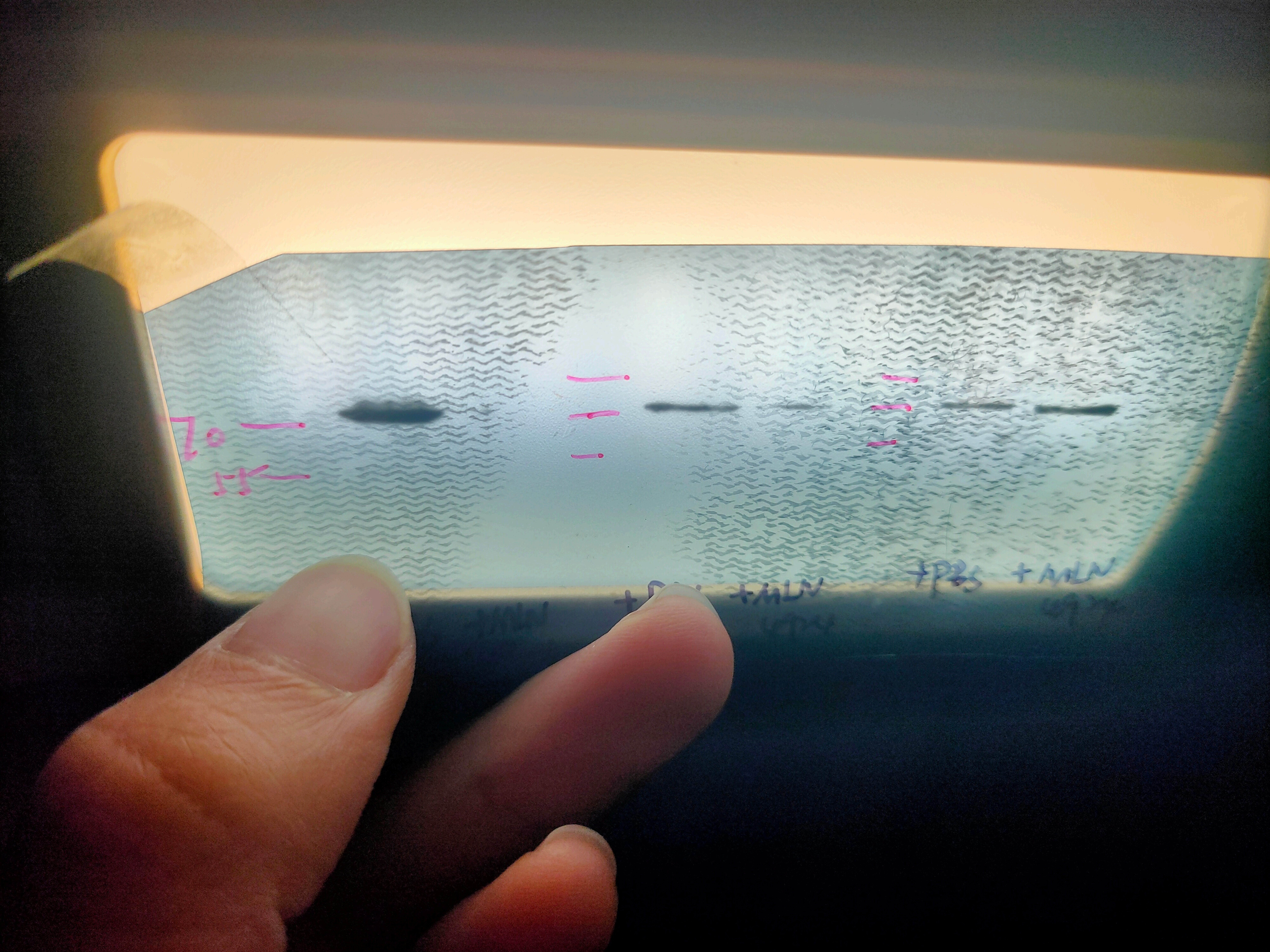

70
55
+PBS
+MLN

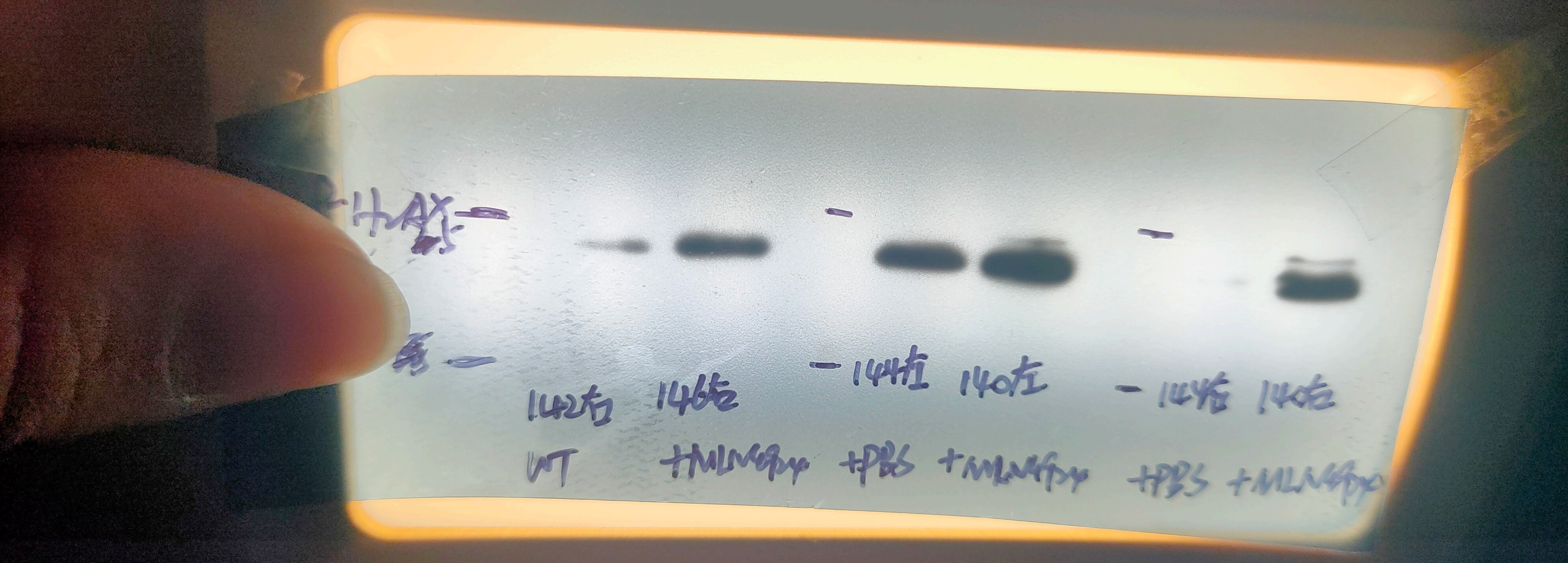

140左
WT
+PBS
+PBS

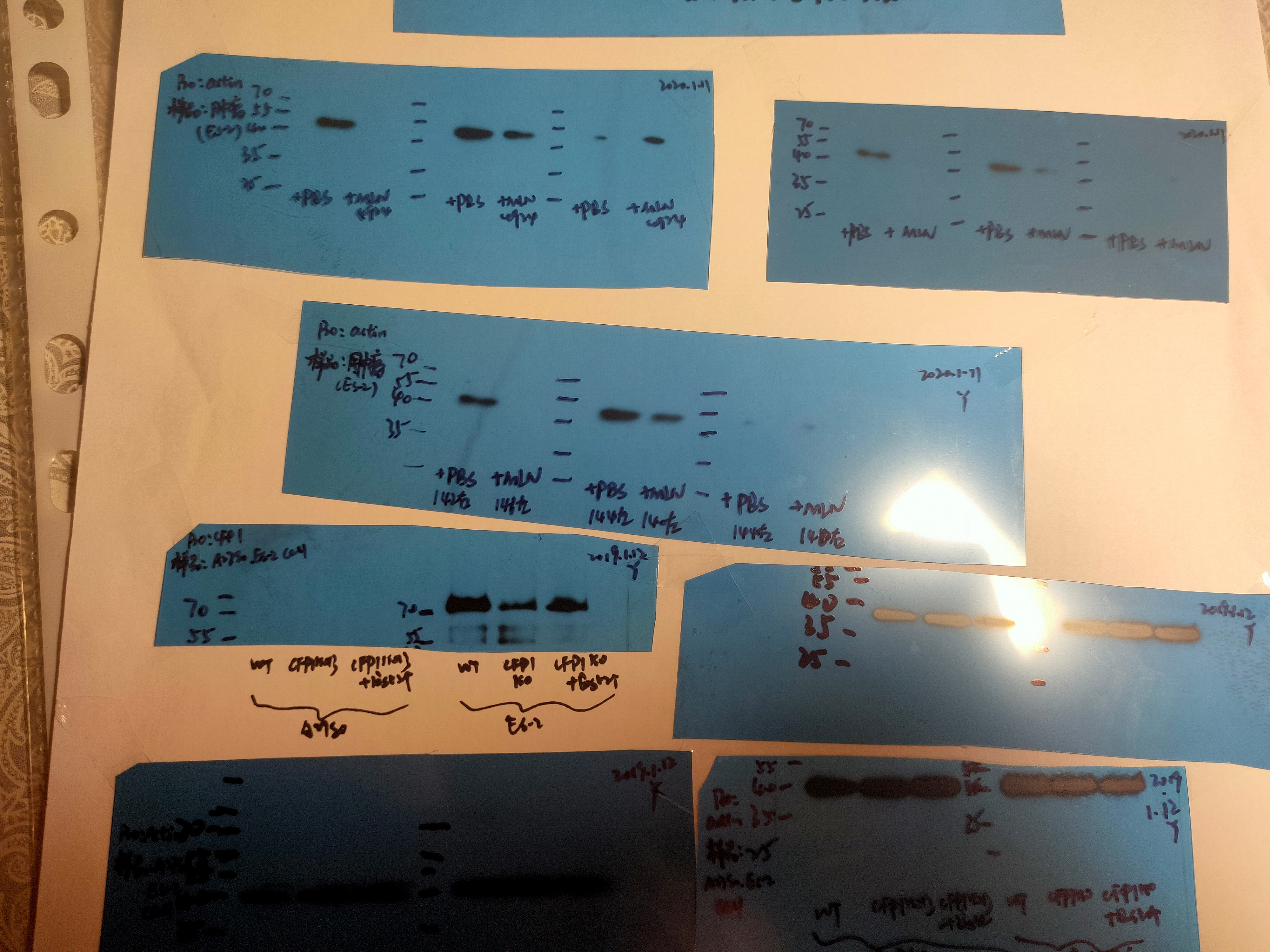

Pro: actin
70
35
+PBS
+MLN
2020.1.11
WT
Es-2

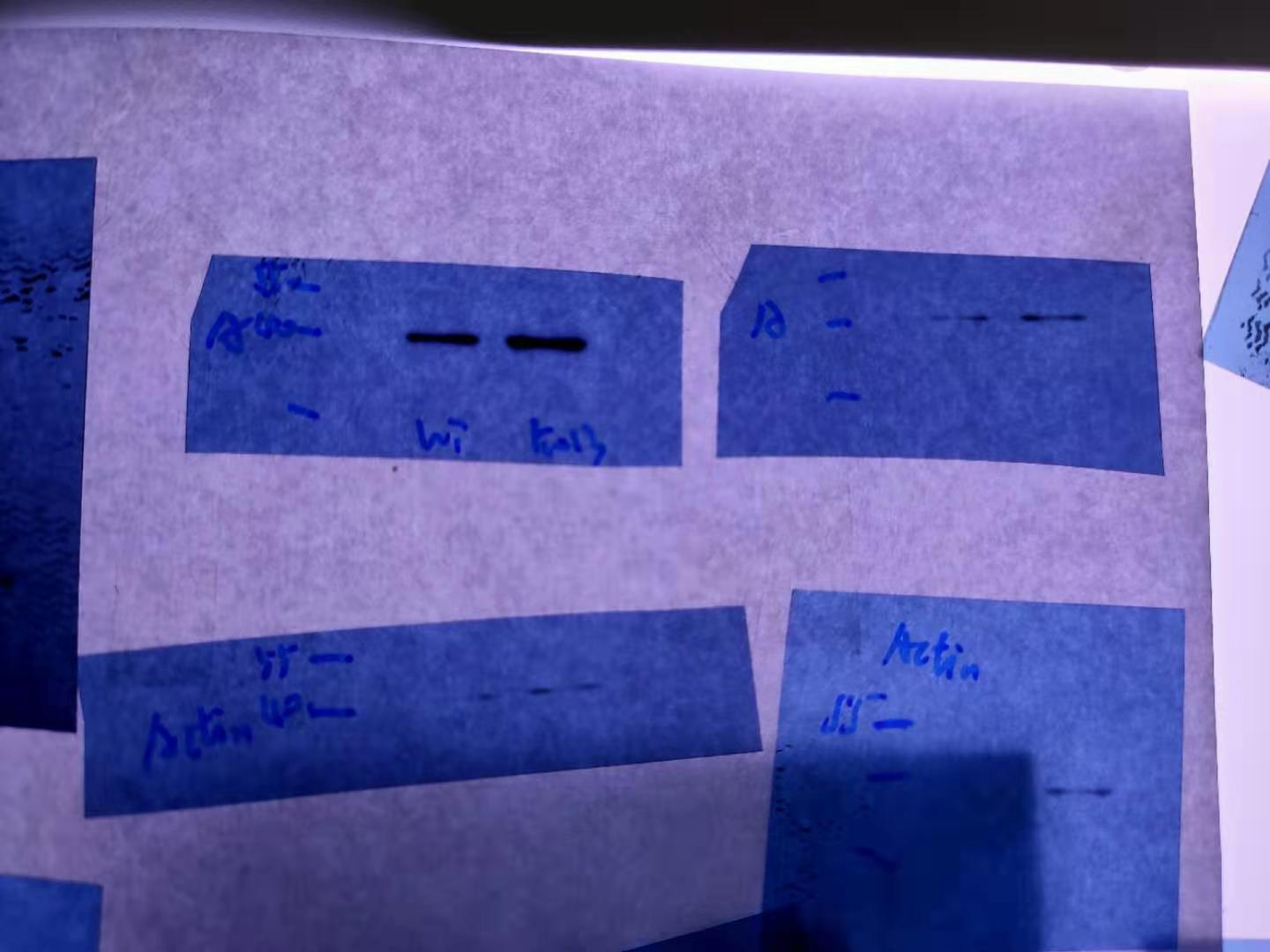
Actin

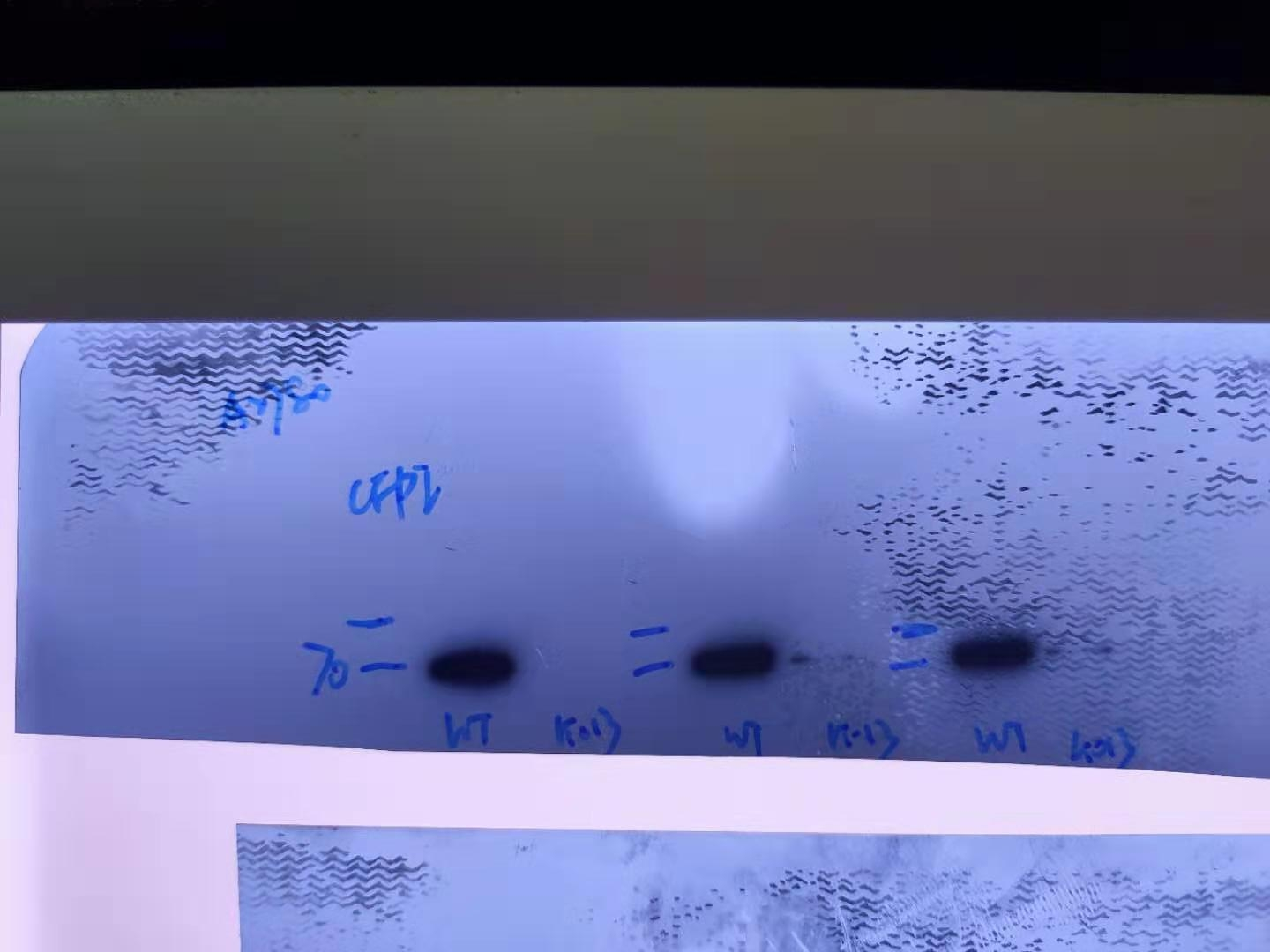
70
WT

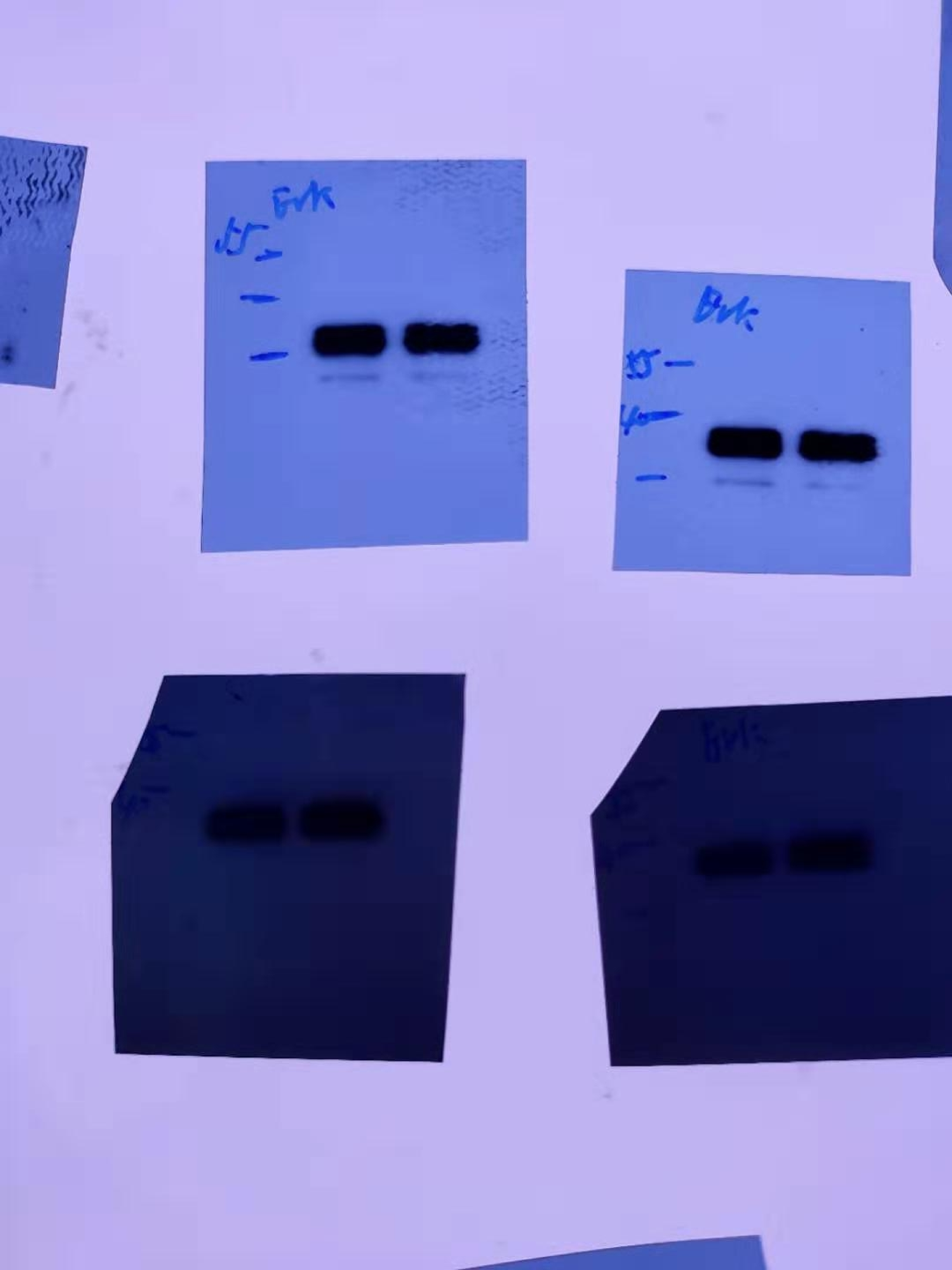

Erk
55
Erk
55
40

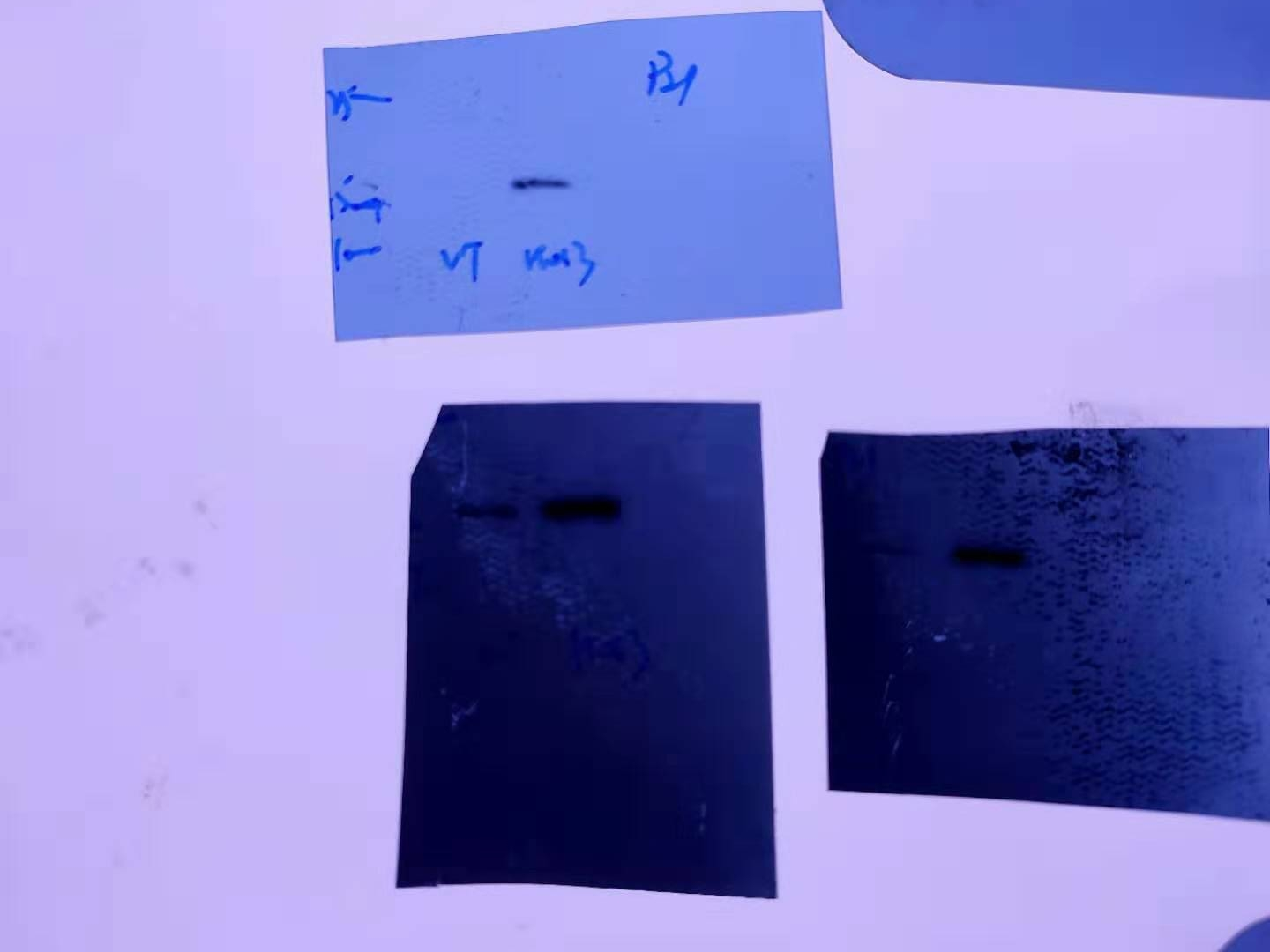

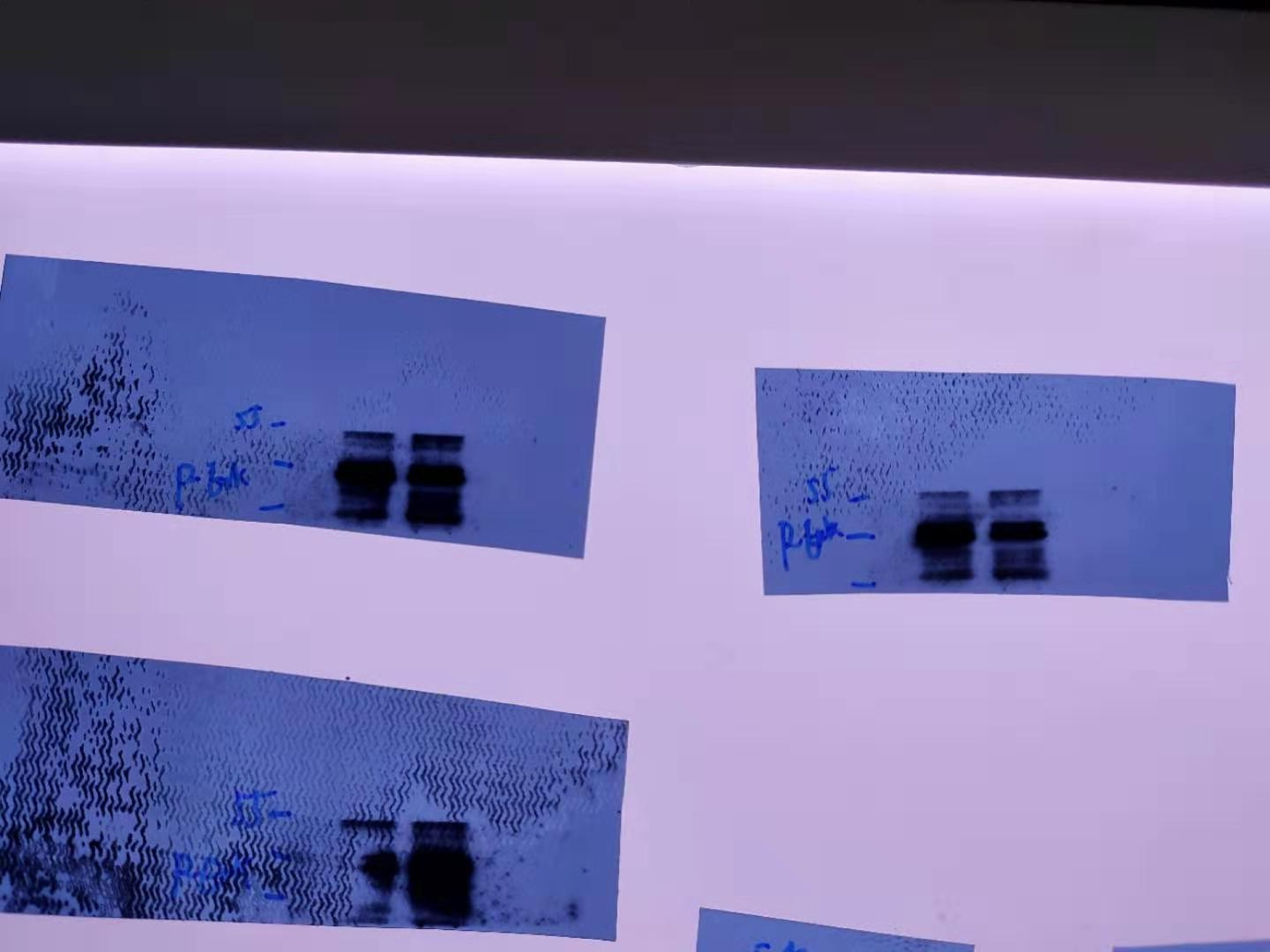
55
55
55

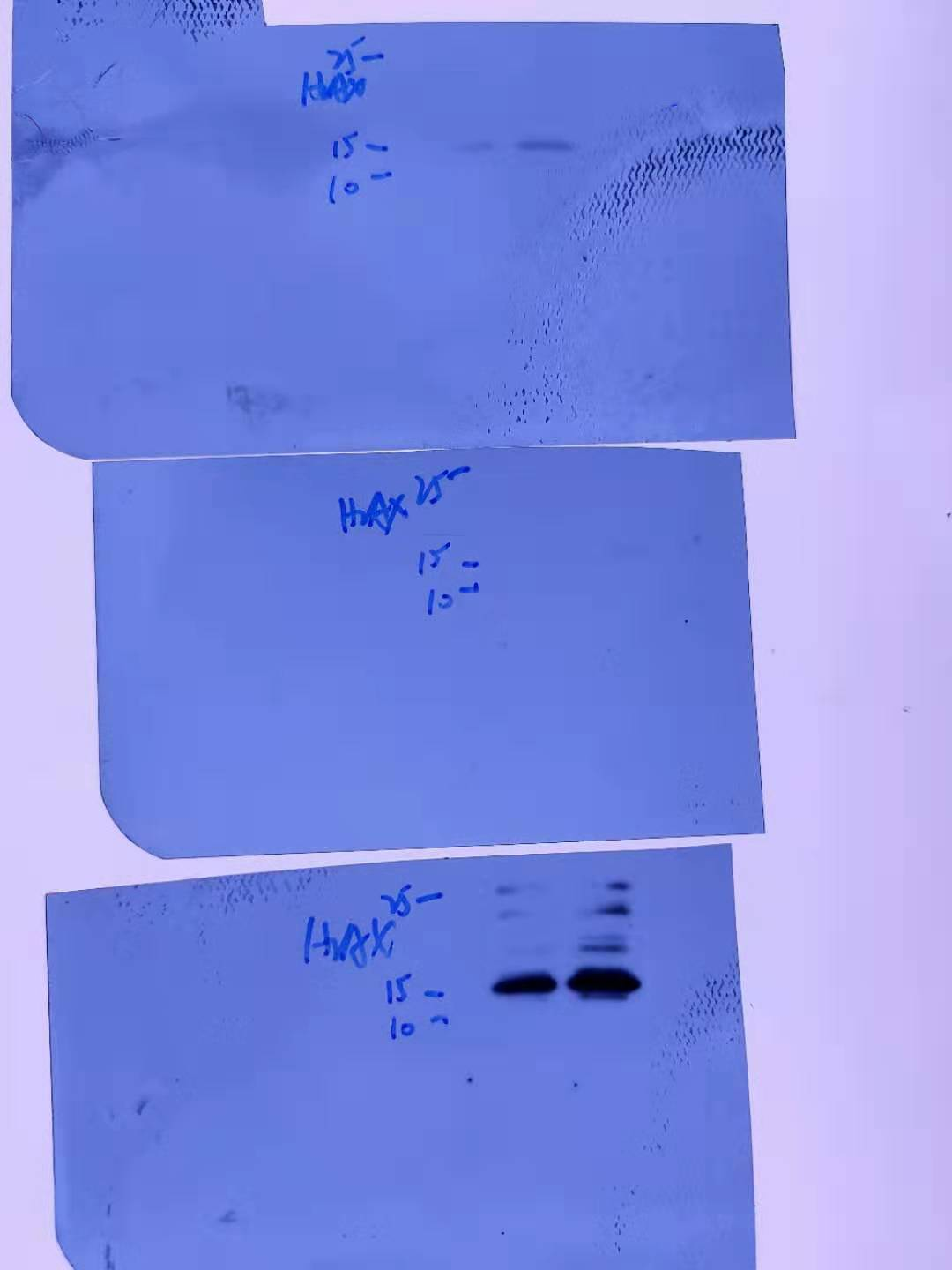
25
H2AX
15
10
H2AX 25
15
10
25
H2AX
15
10

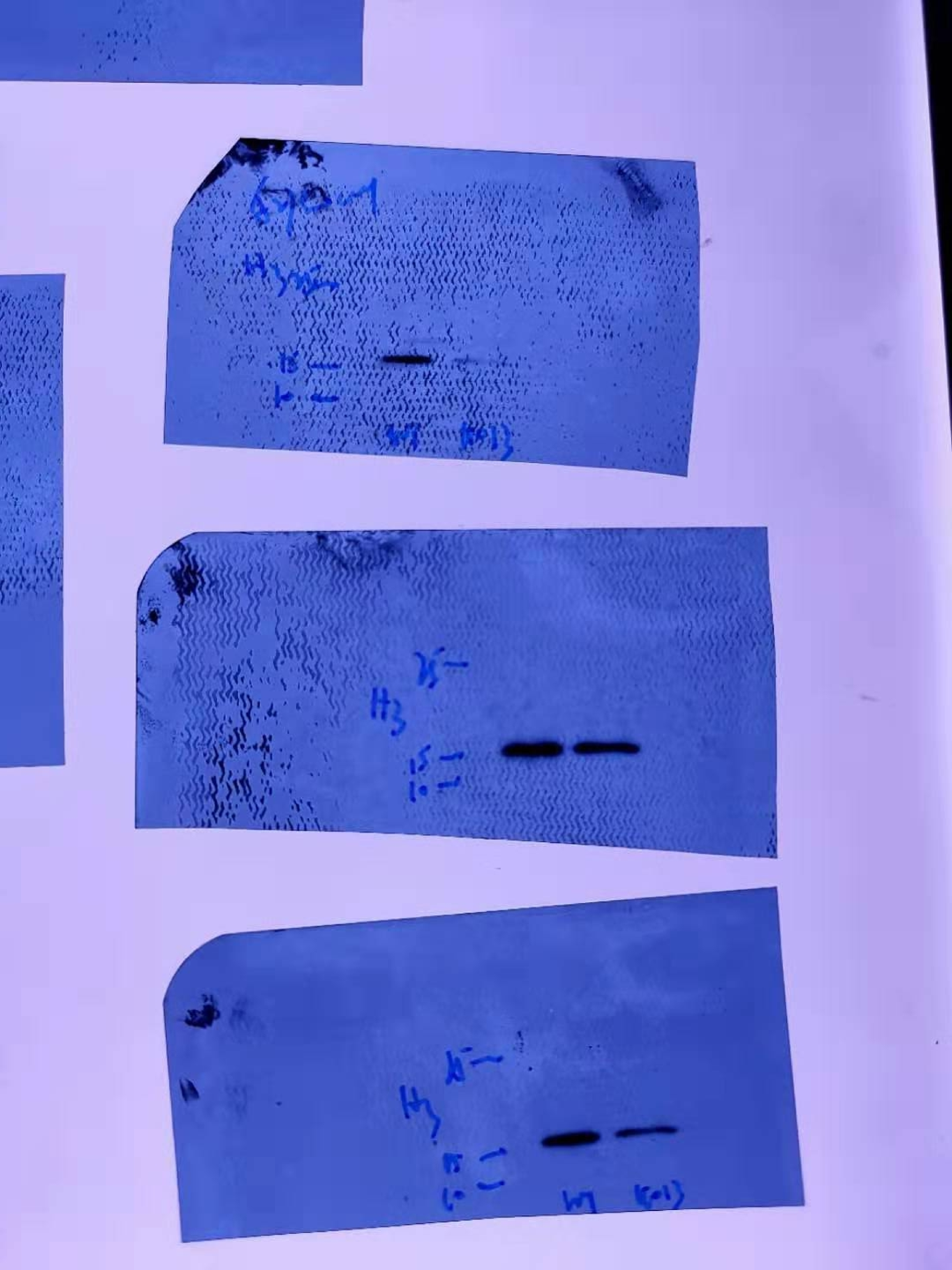
H3

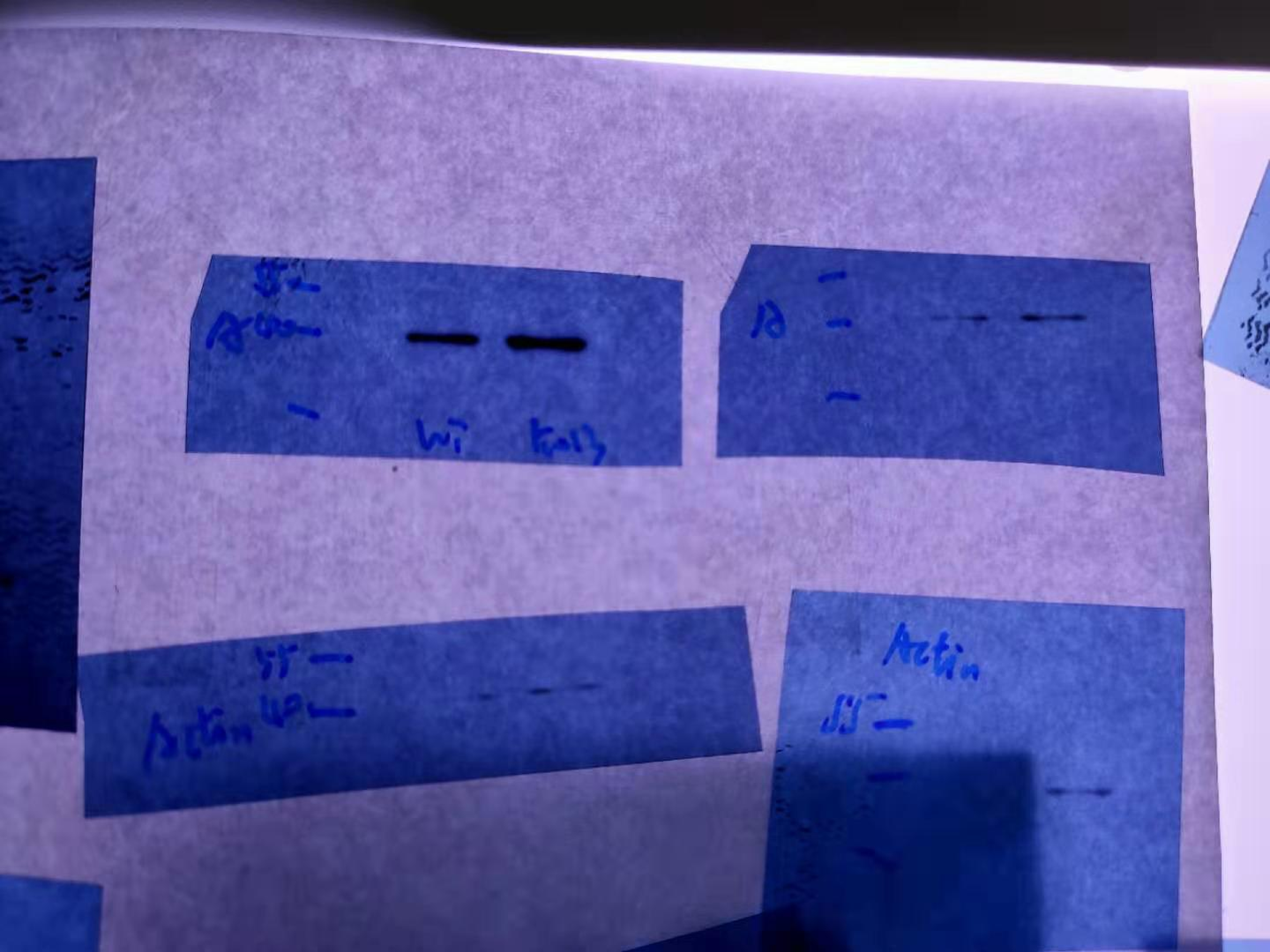
Actin

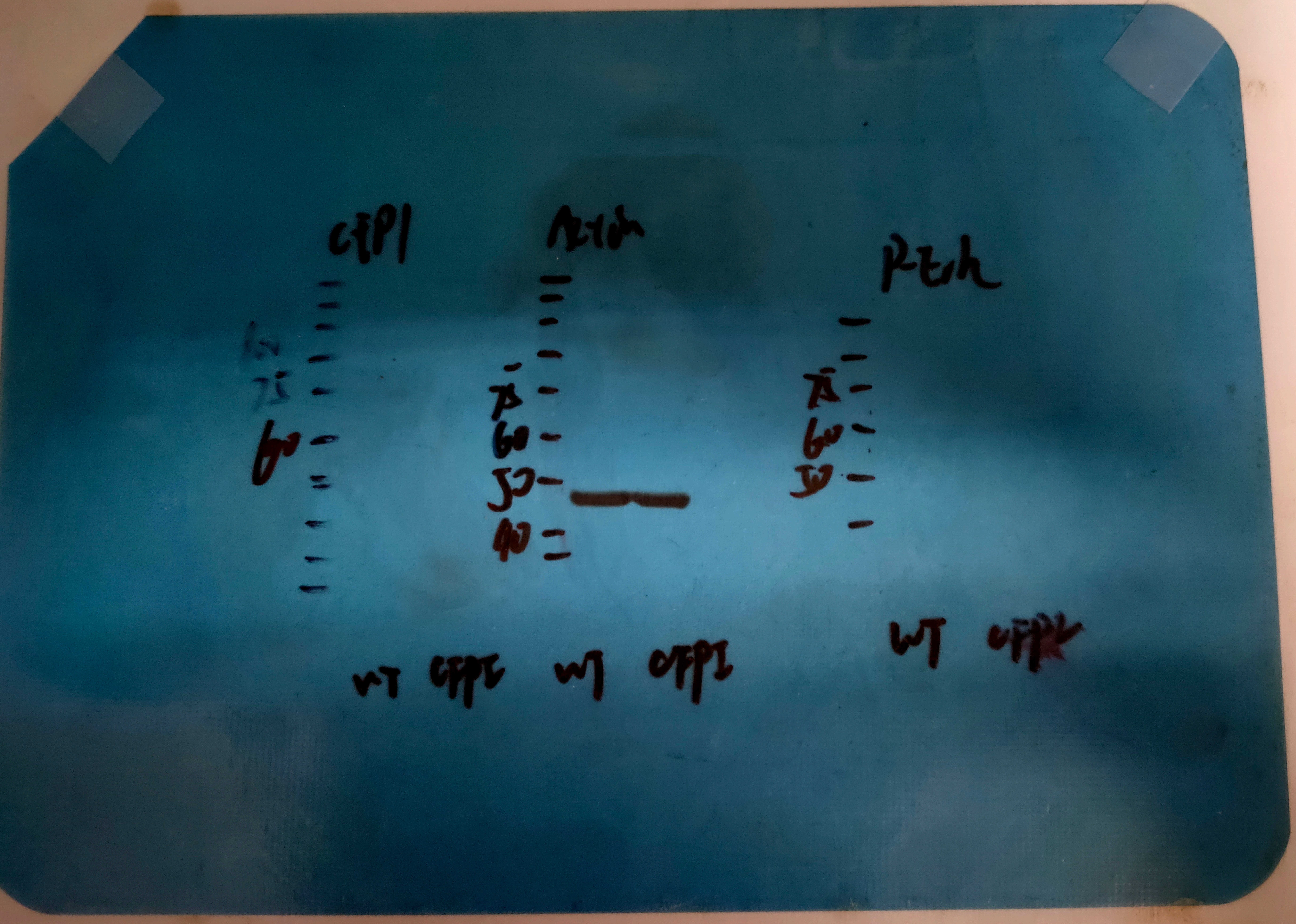
GFP1
Actin
Ptch
75
60
50
40
WT
WT
WT

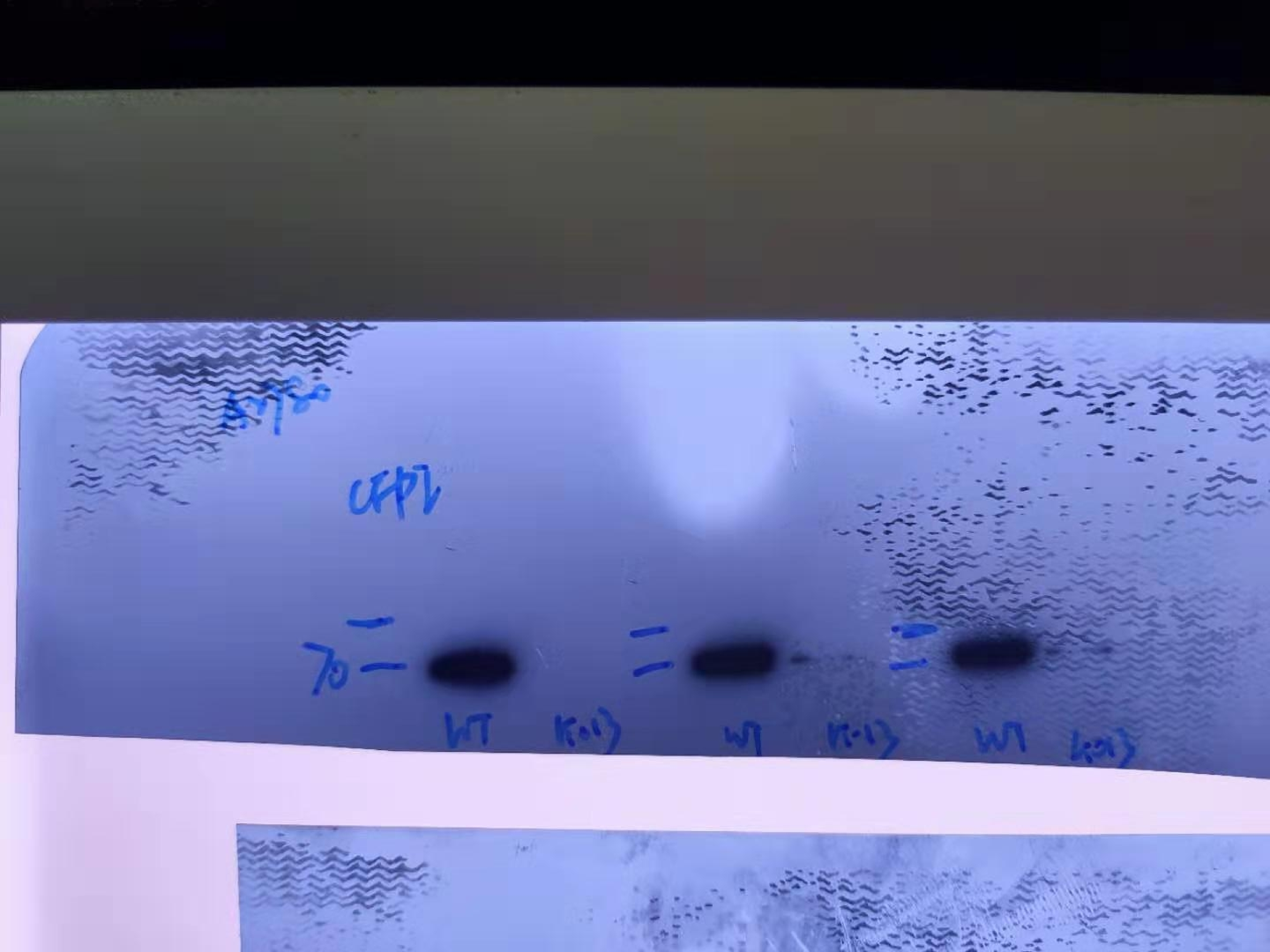
70
WT
WT
WT

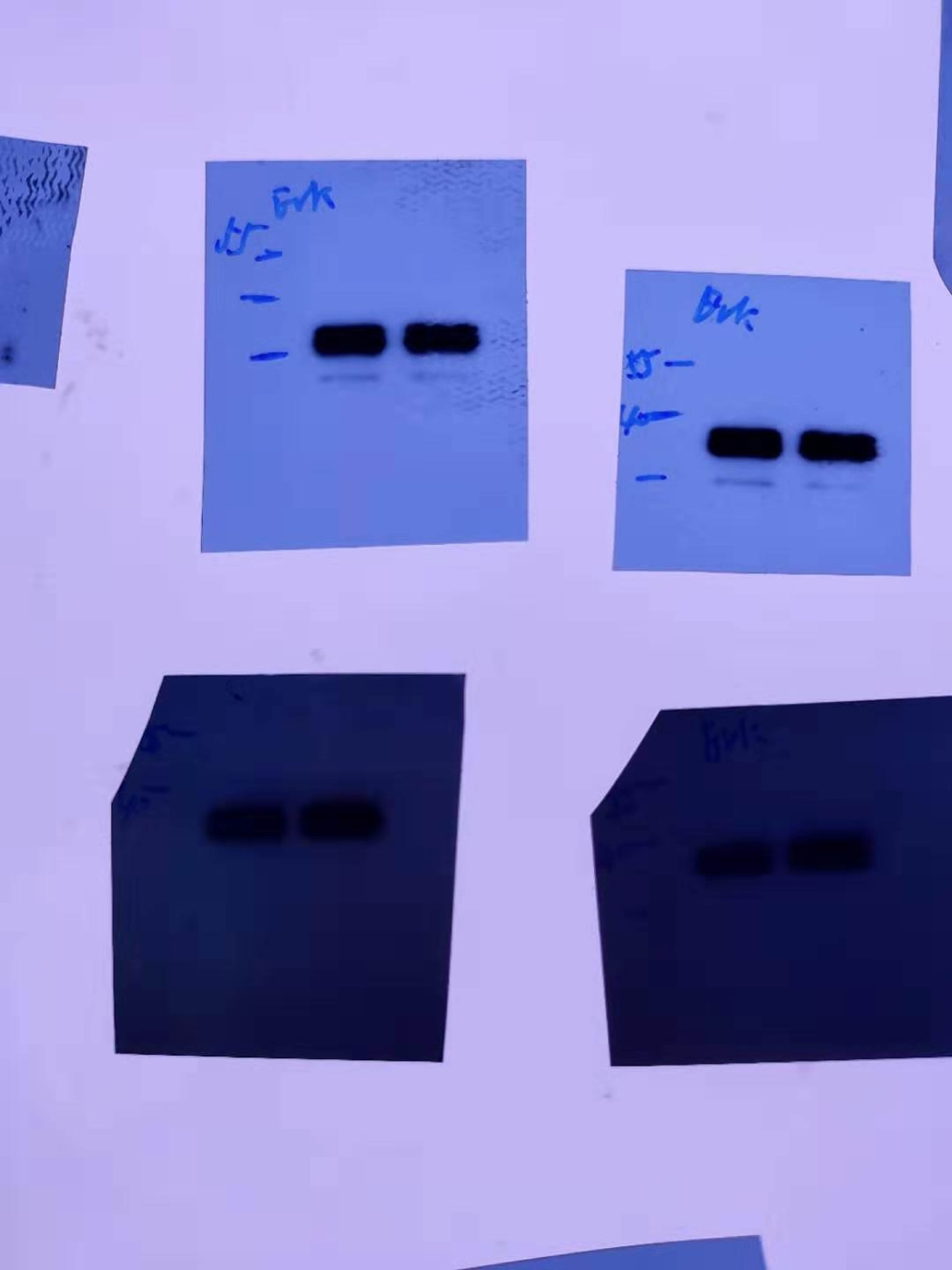
Erk
55
Erk
55
40

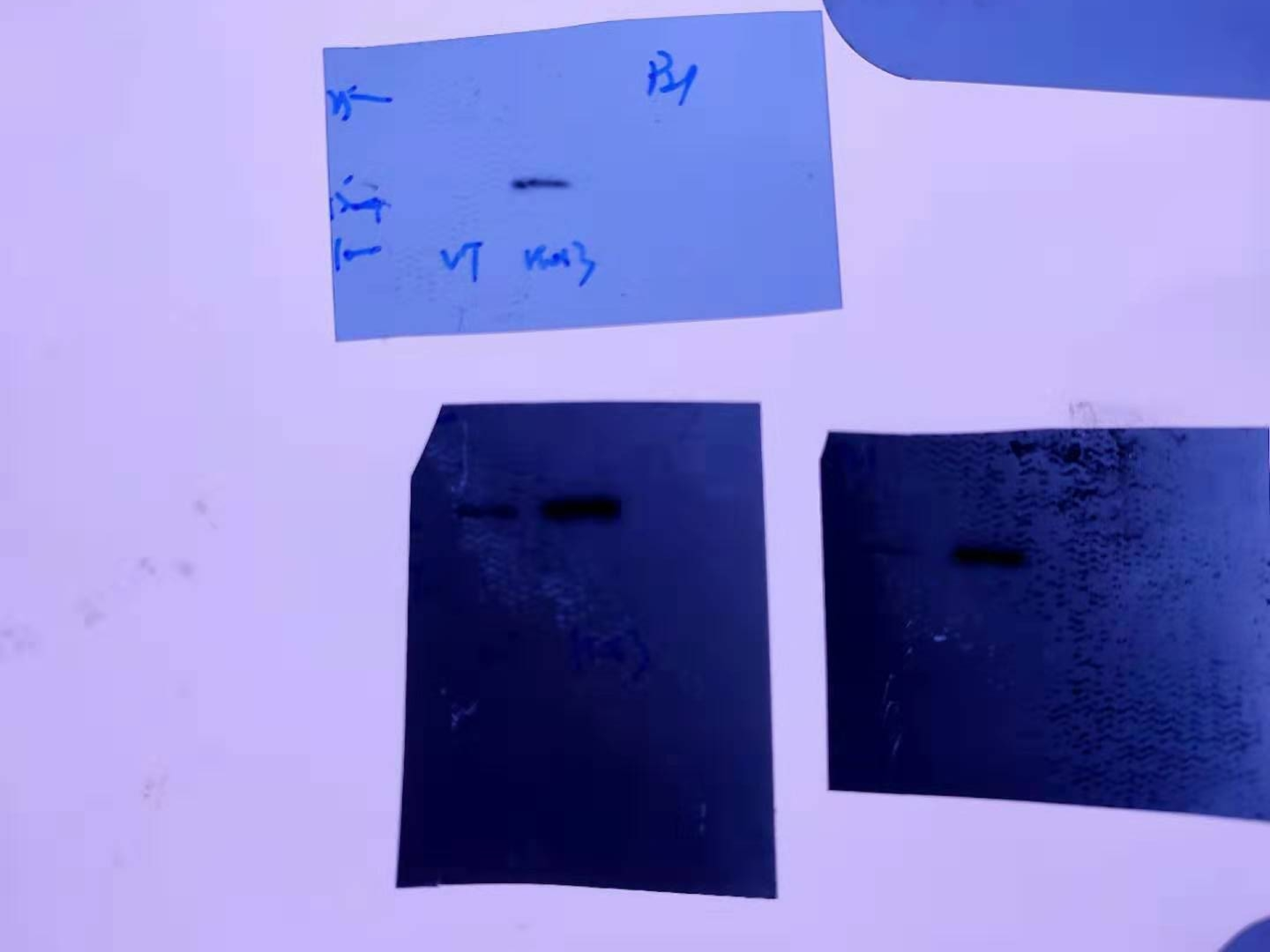

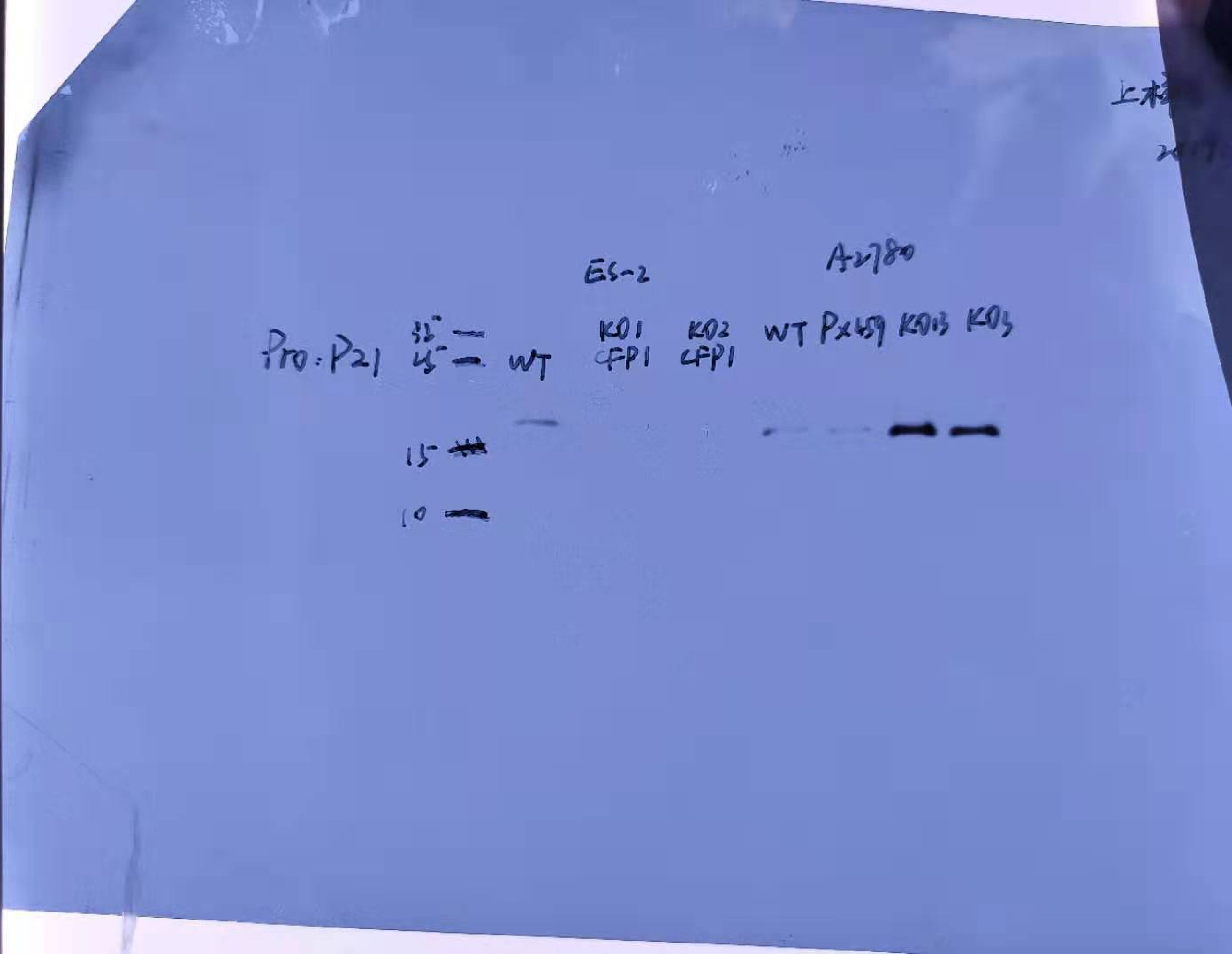
Pro: P21
35
45
15
10
ES-2
WT
KO1 CFP1
KO2 CFP1
A2780
WT
Px459
KO13
KO3

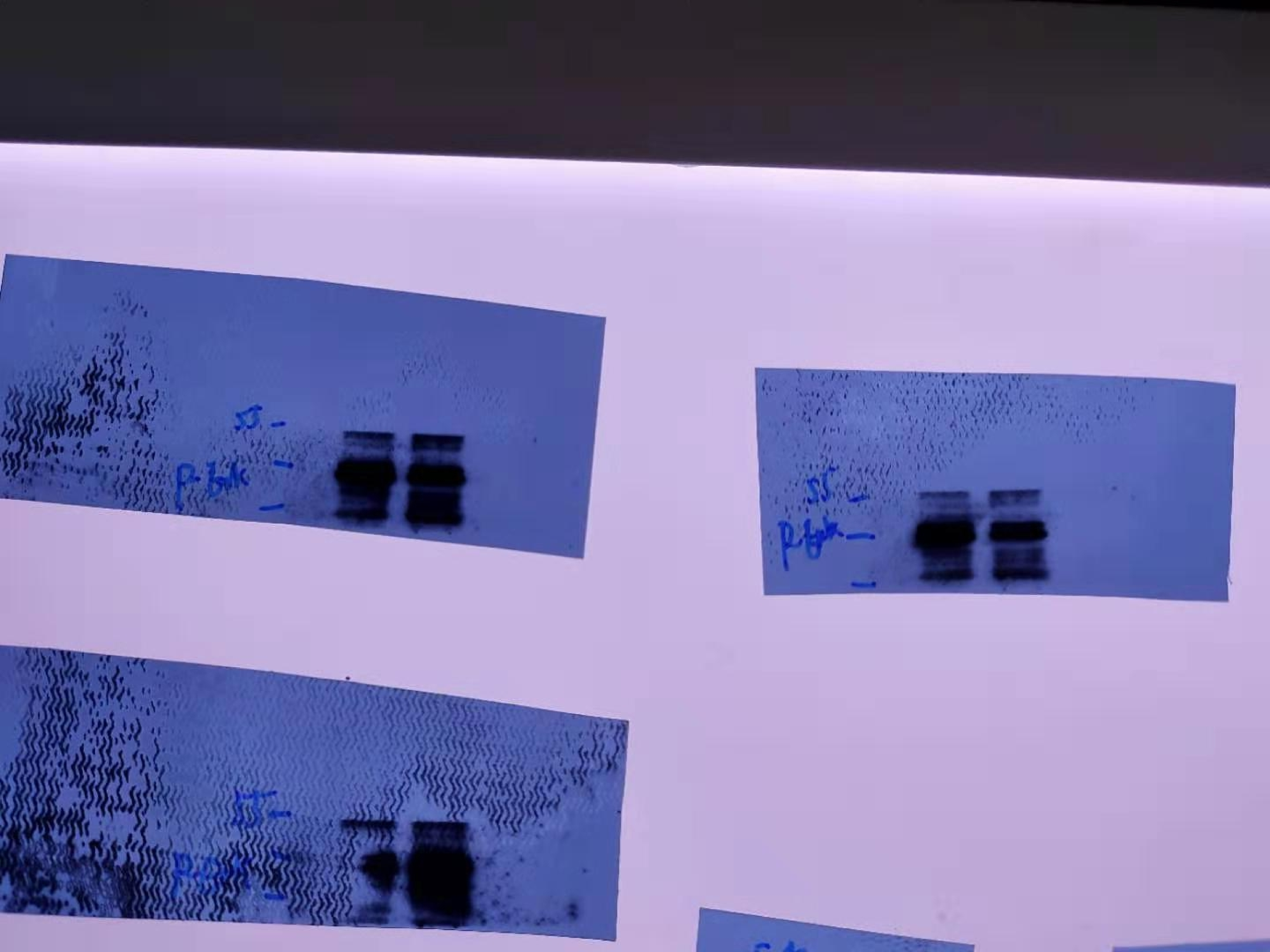

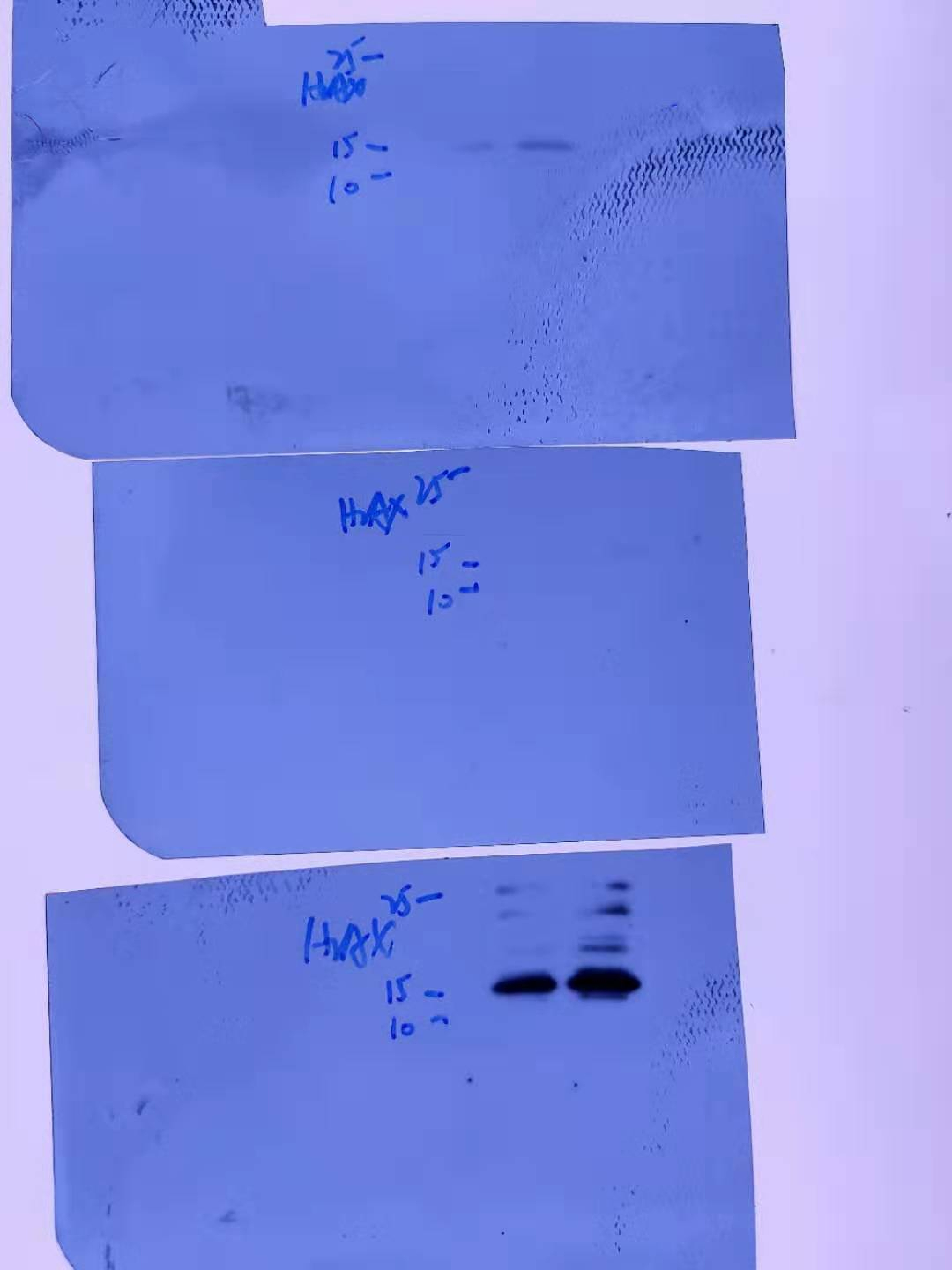
25
H2AX
15
10
H2AX 25
15
10
25
H2AX
15
10

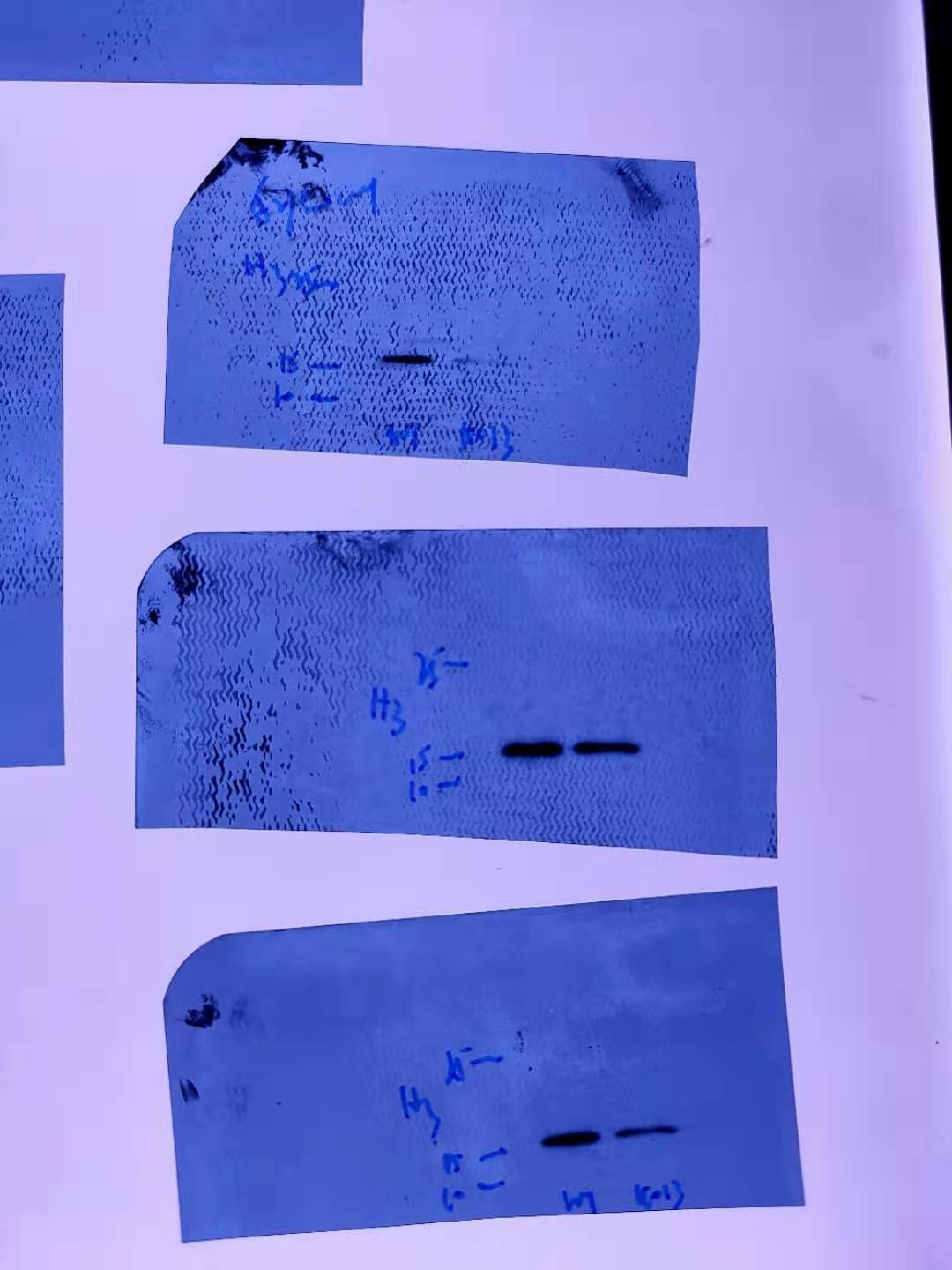
H3
15 —
10 —
WT

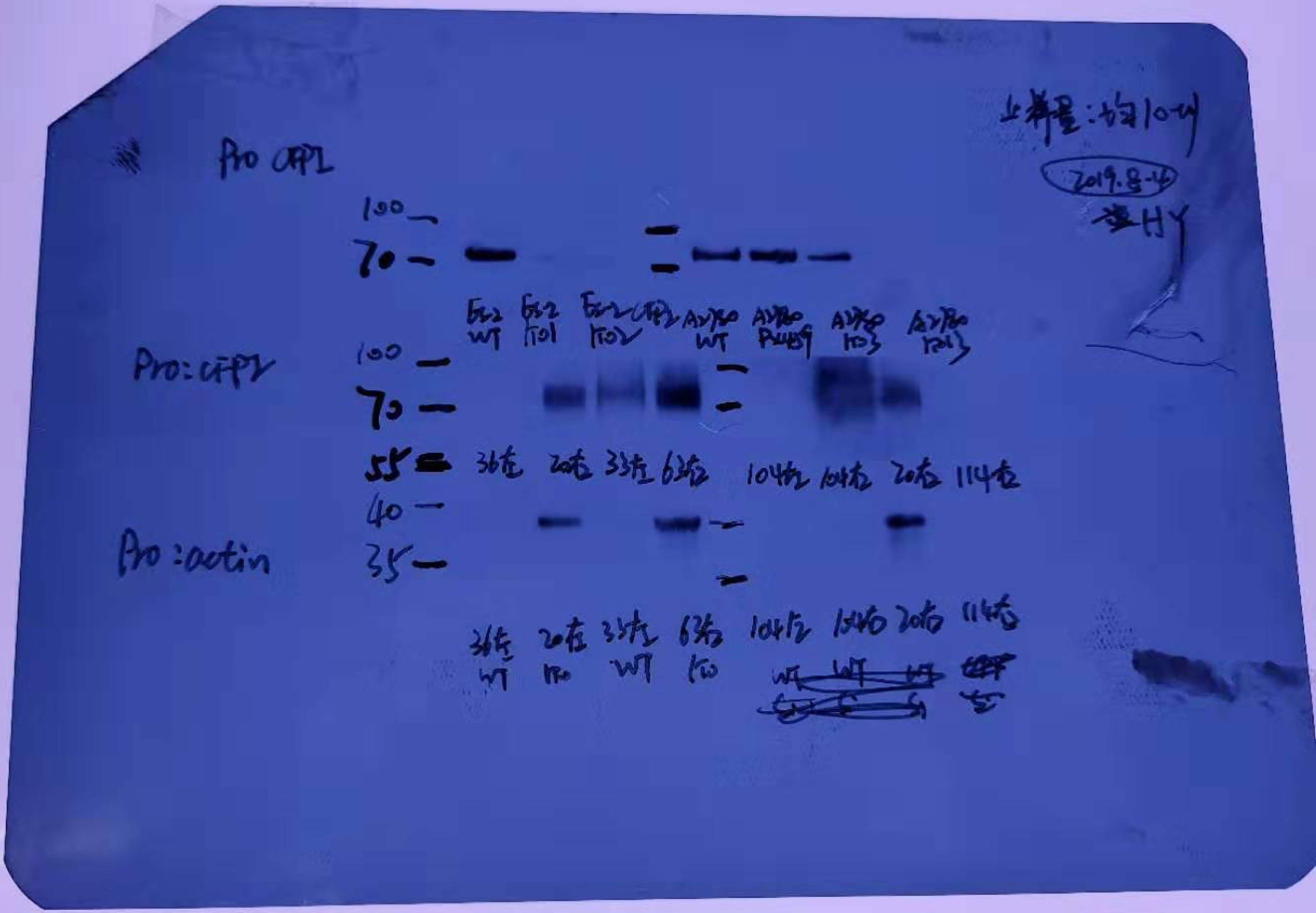

Pro:CFP2
Pro:actin
100
70
55
40
35
WT

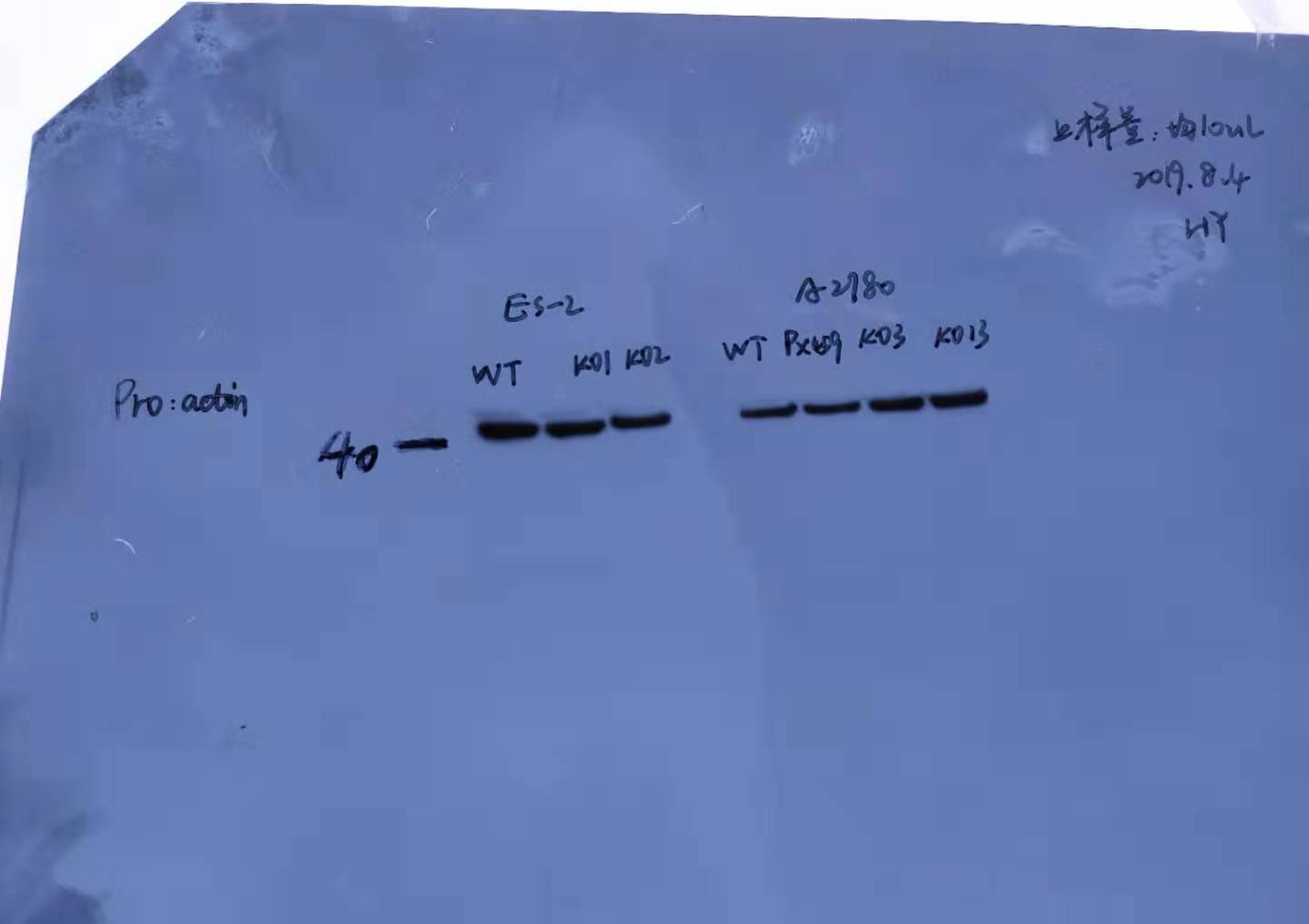
上样量：均10uL
2019.8.4
HY
ES-2
A2780
WT
KO1
KO2
WT
Px459
KO3
KO13
Pro: actin
40

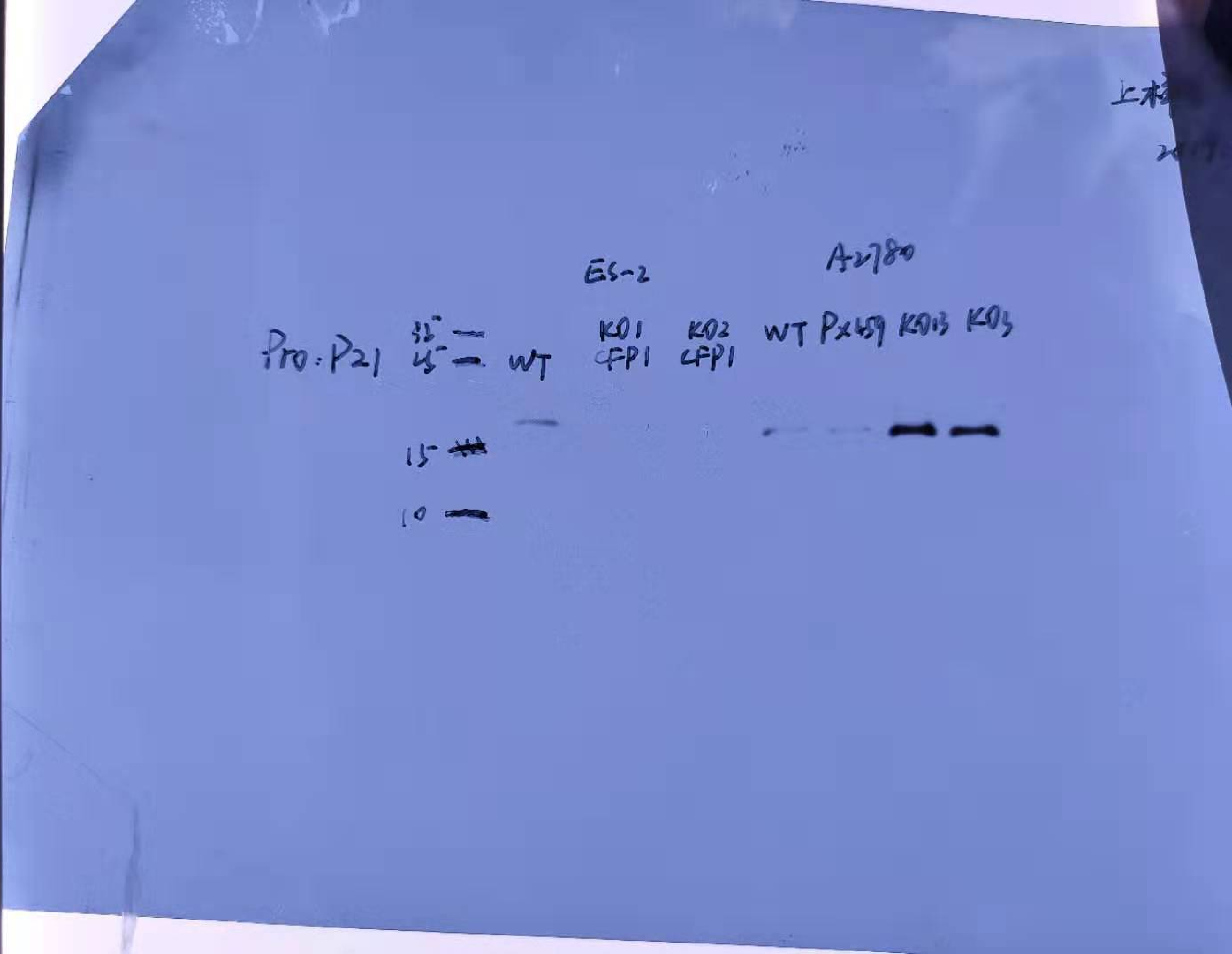
ES-2
A2780
Pro: P21
35
45
15
10
WT
KO1 CFP1
KO2 CFP1
WT
Px459
KO13
KO3

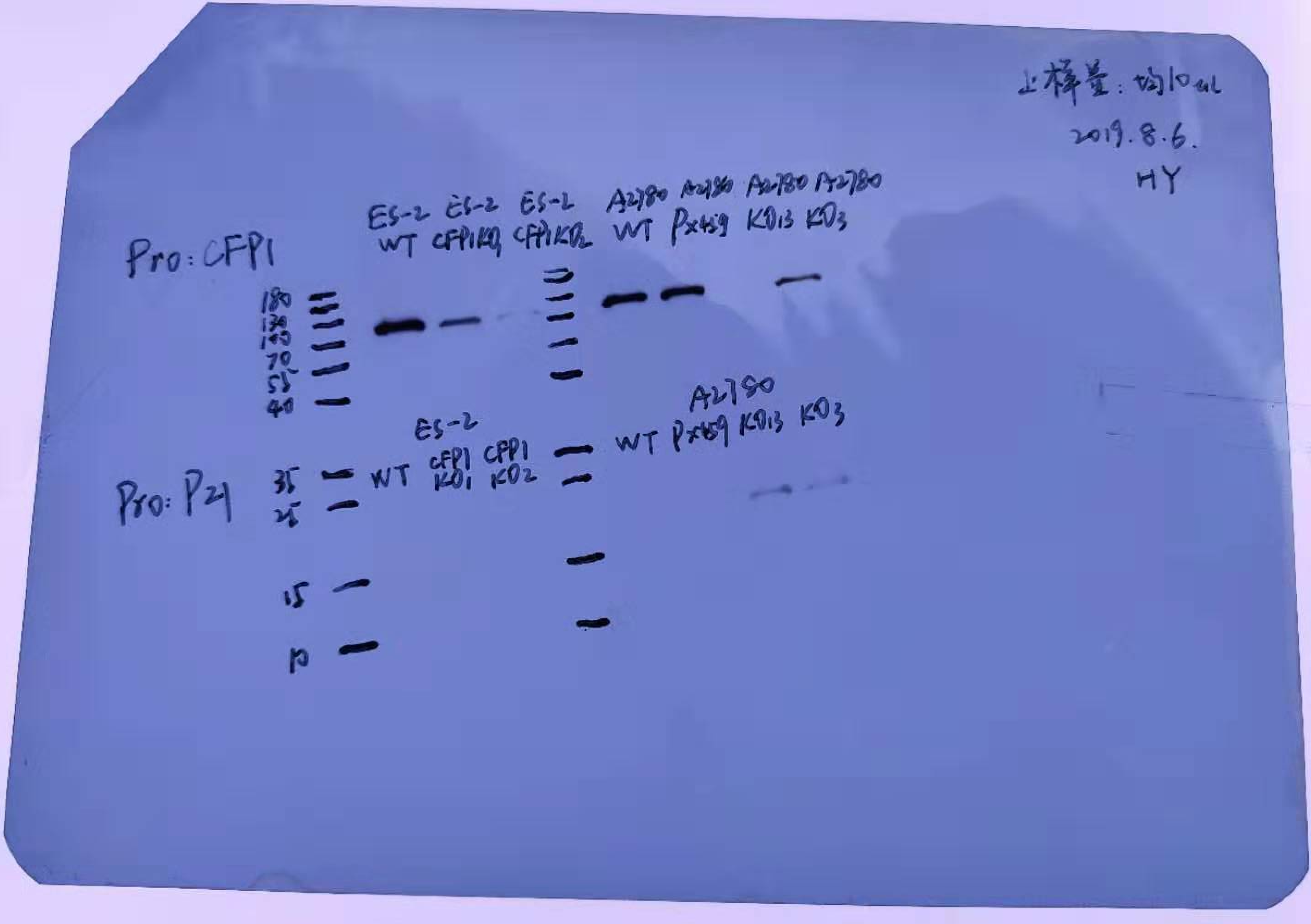
上样量：均10uL
2019.8.6.
HY
Pro: CFP1
ES-2 WT
ES-2 CFP1 KO1
ES-2 CFP1 KO2
A2780 WT
A2780 Px459
A2780 KO13
A2780 KO3
180
130
100
70
55
40
Pro: P21
ES-2
WT
CFP1 KO1
CFP1 KO2
A2780
WT
Px459
KO13
KO3
35
25
15
10

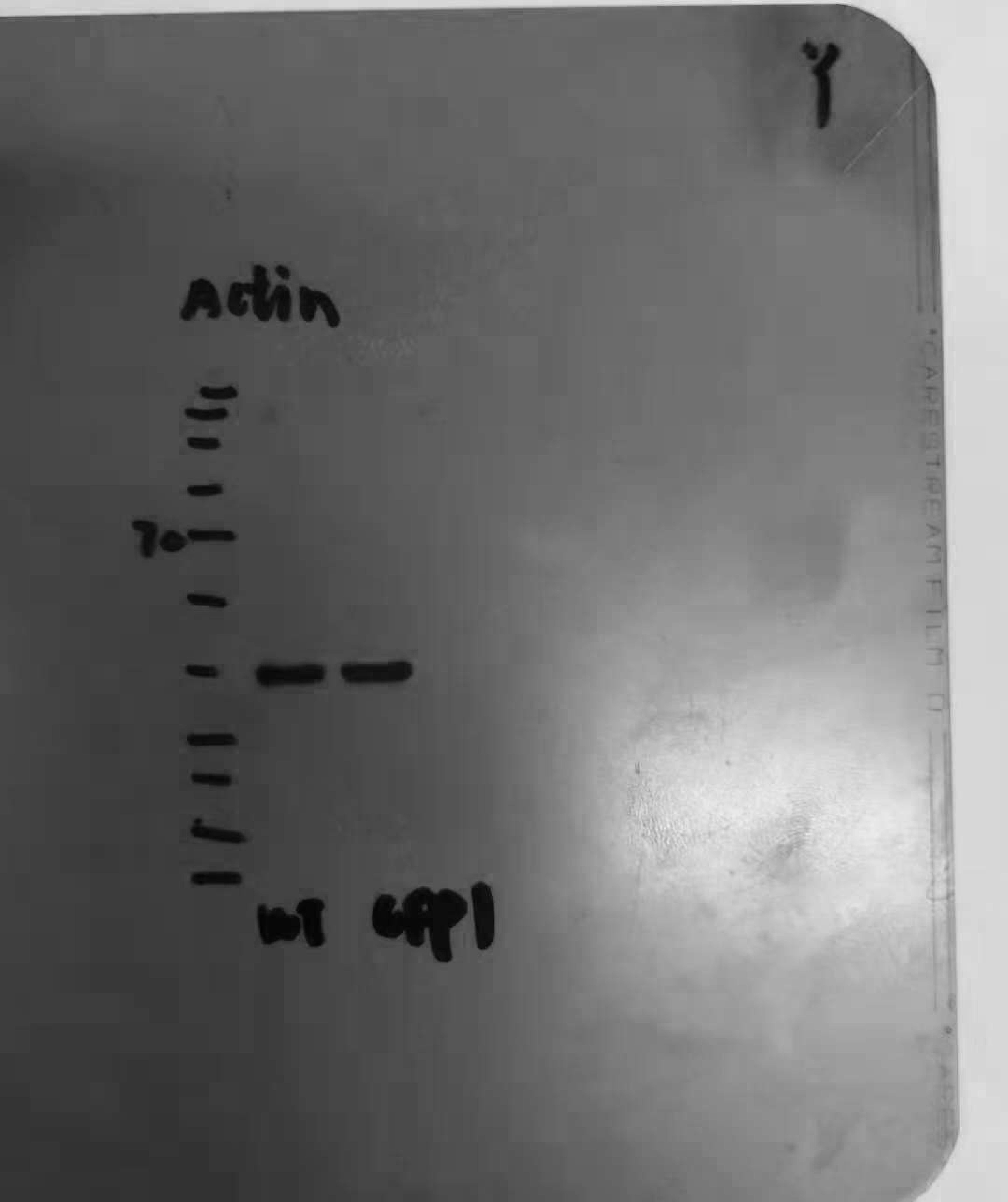
Actin
70
wt

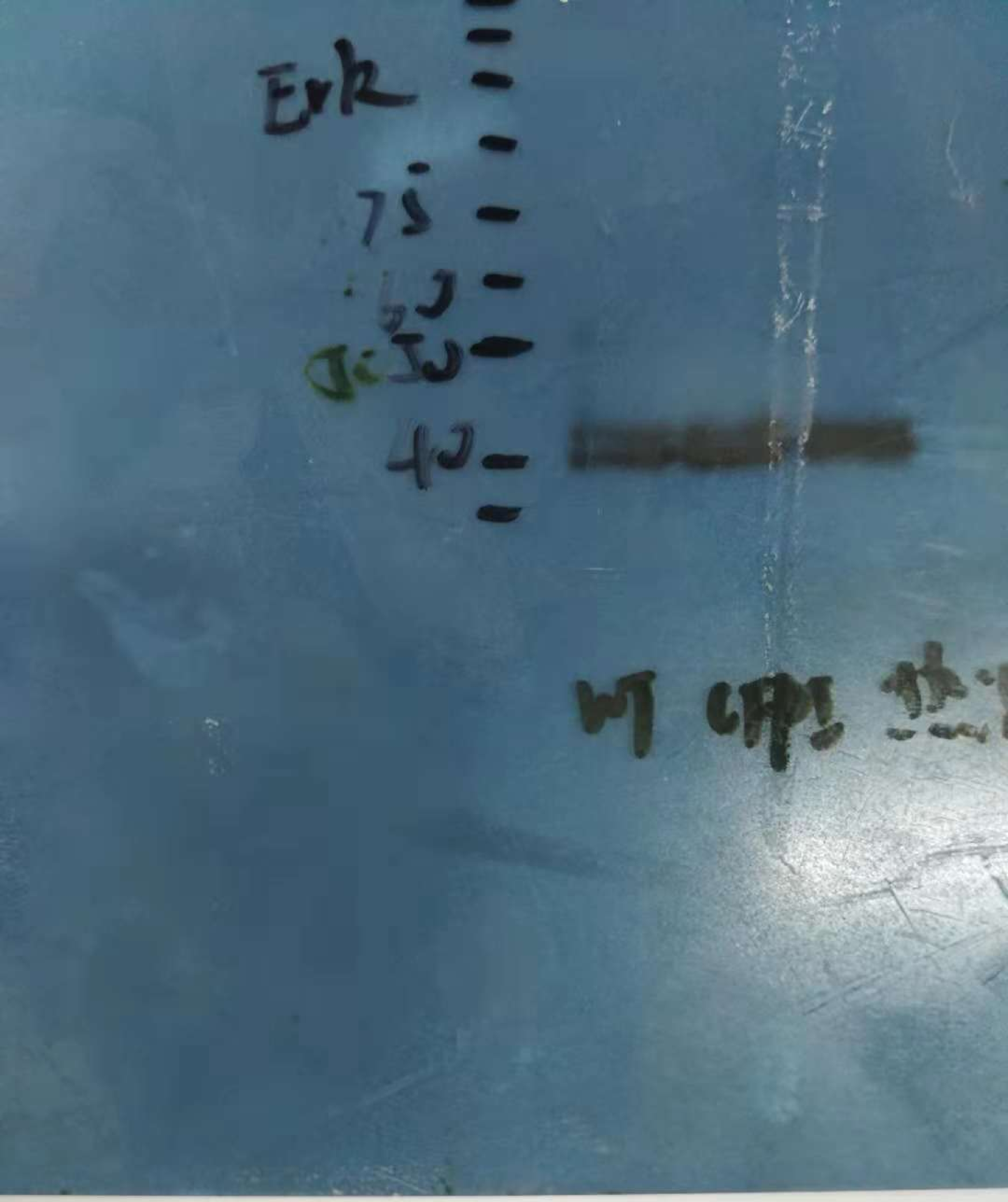
Erk

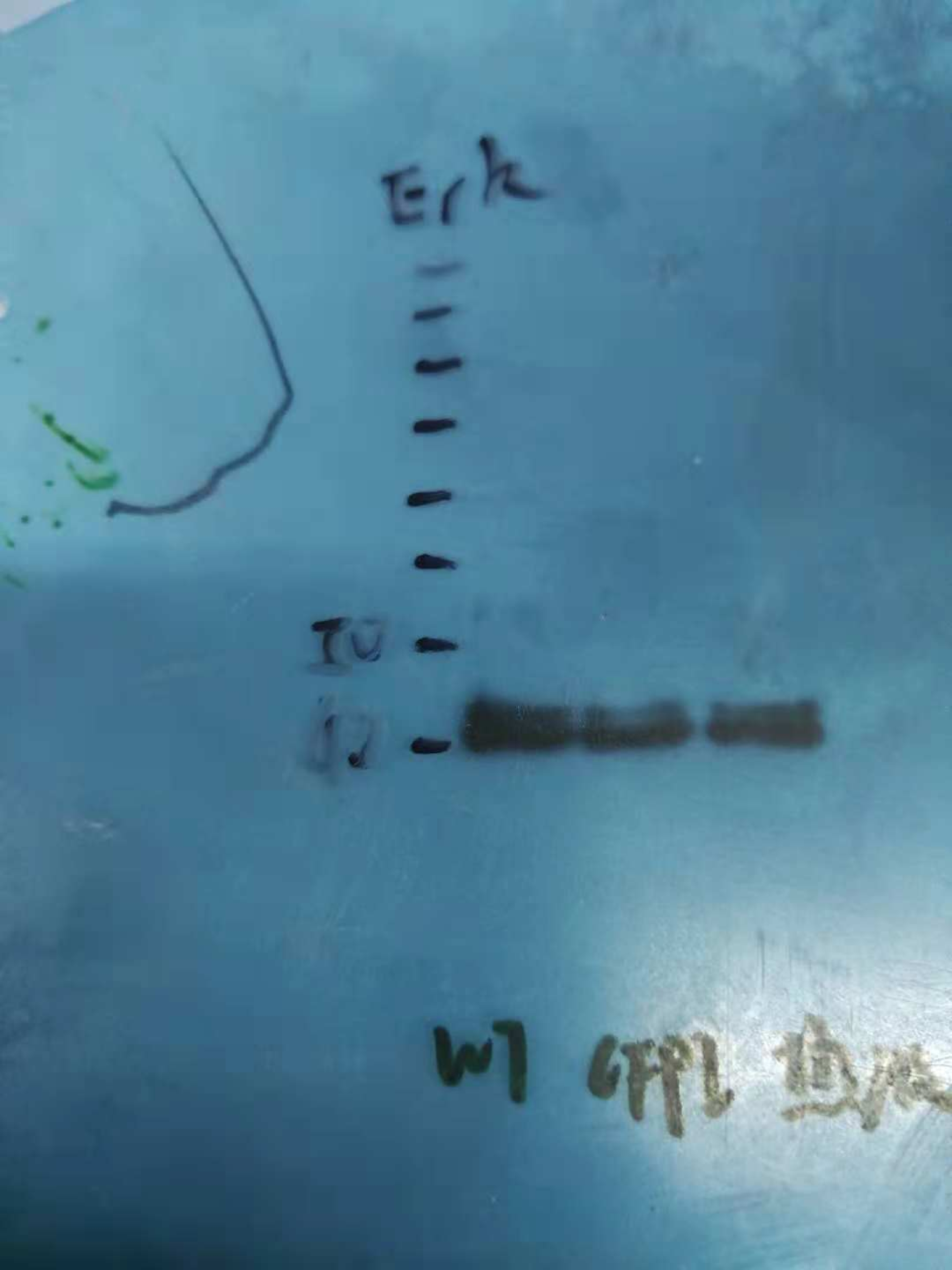
Erk
WT

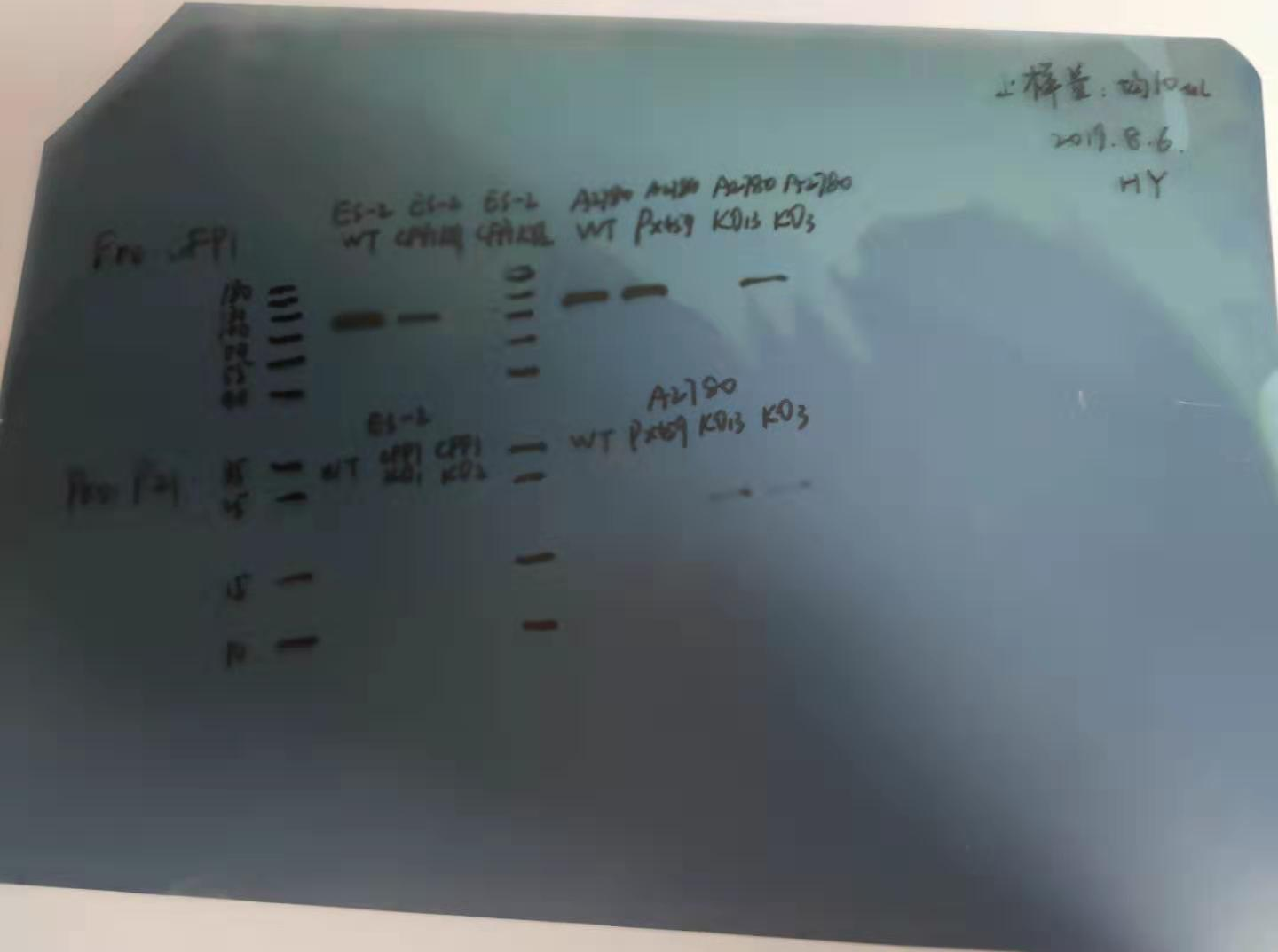

上样量：均10μL
2019.8.6.
HY
ES-2
WT
A2780
WT
Px459
KO13
KO3
ES-2
WT
A2780
WT
Px459
KO13
KO3

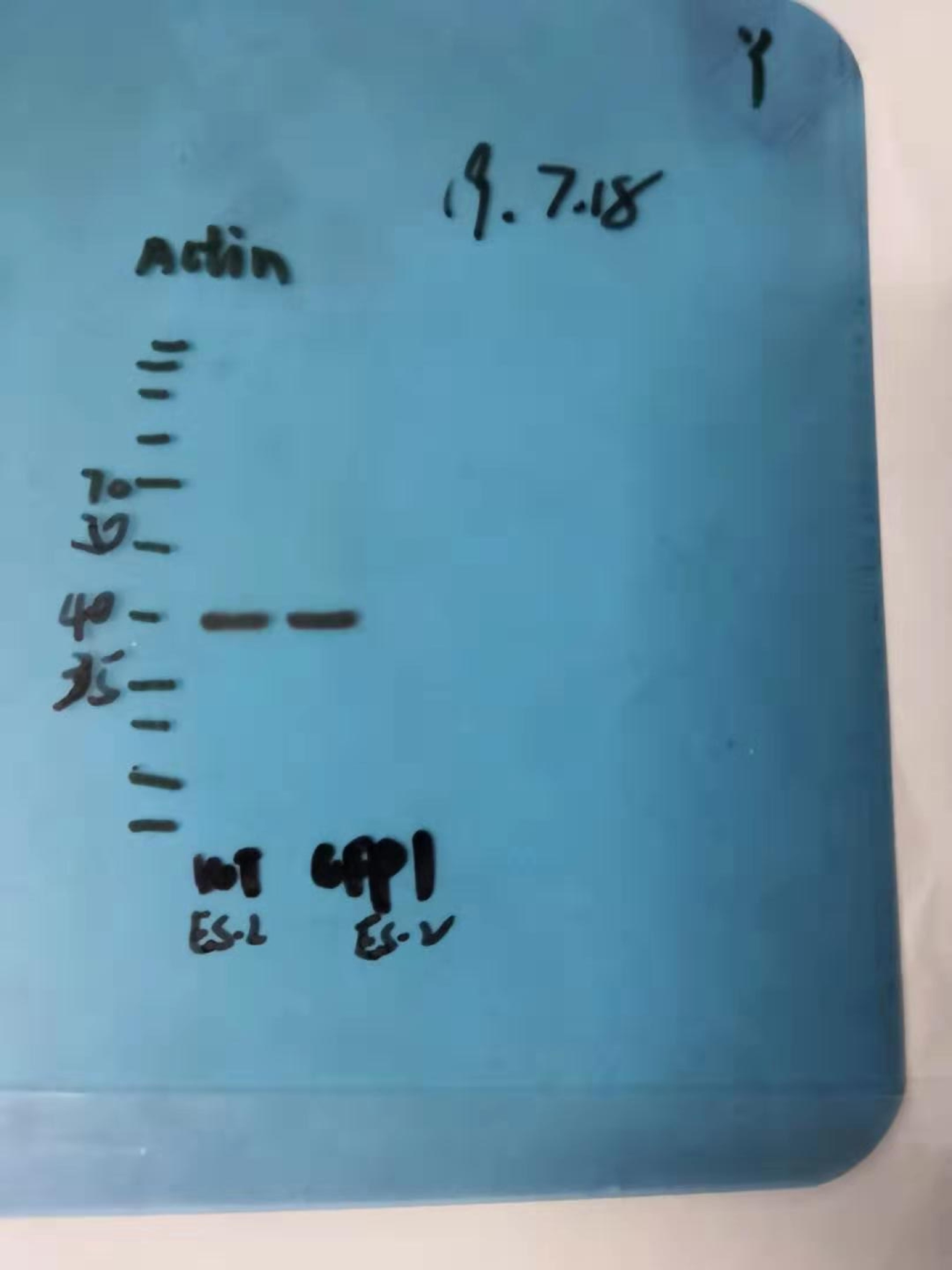
19.7.18
Actin
70
55
40
35
WT

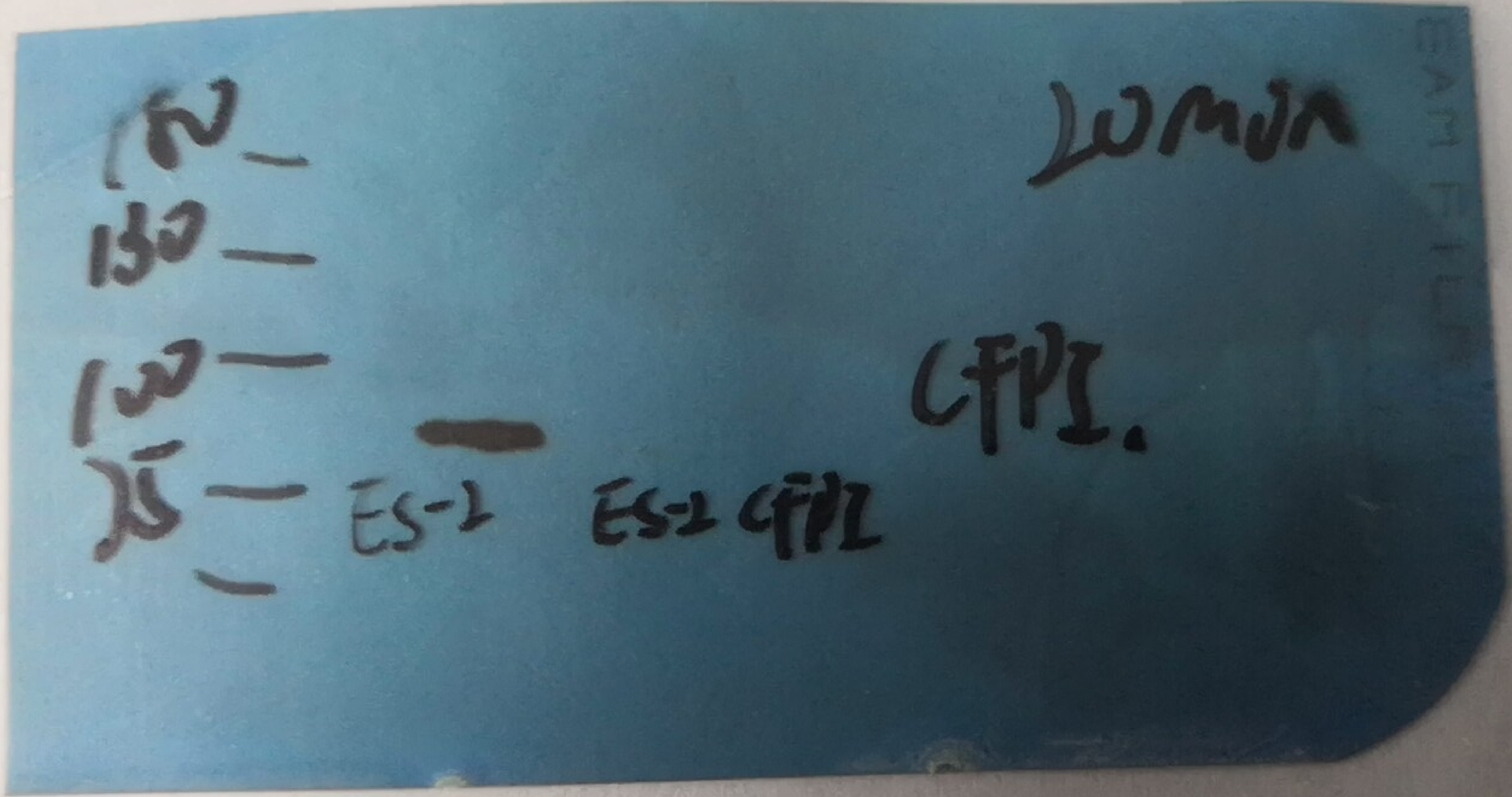

130
100
ES-2
ES-2 CFPI
CFPI.

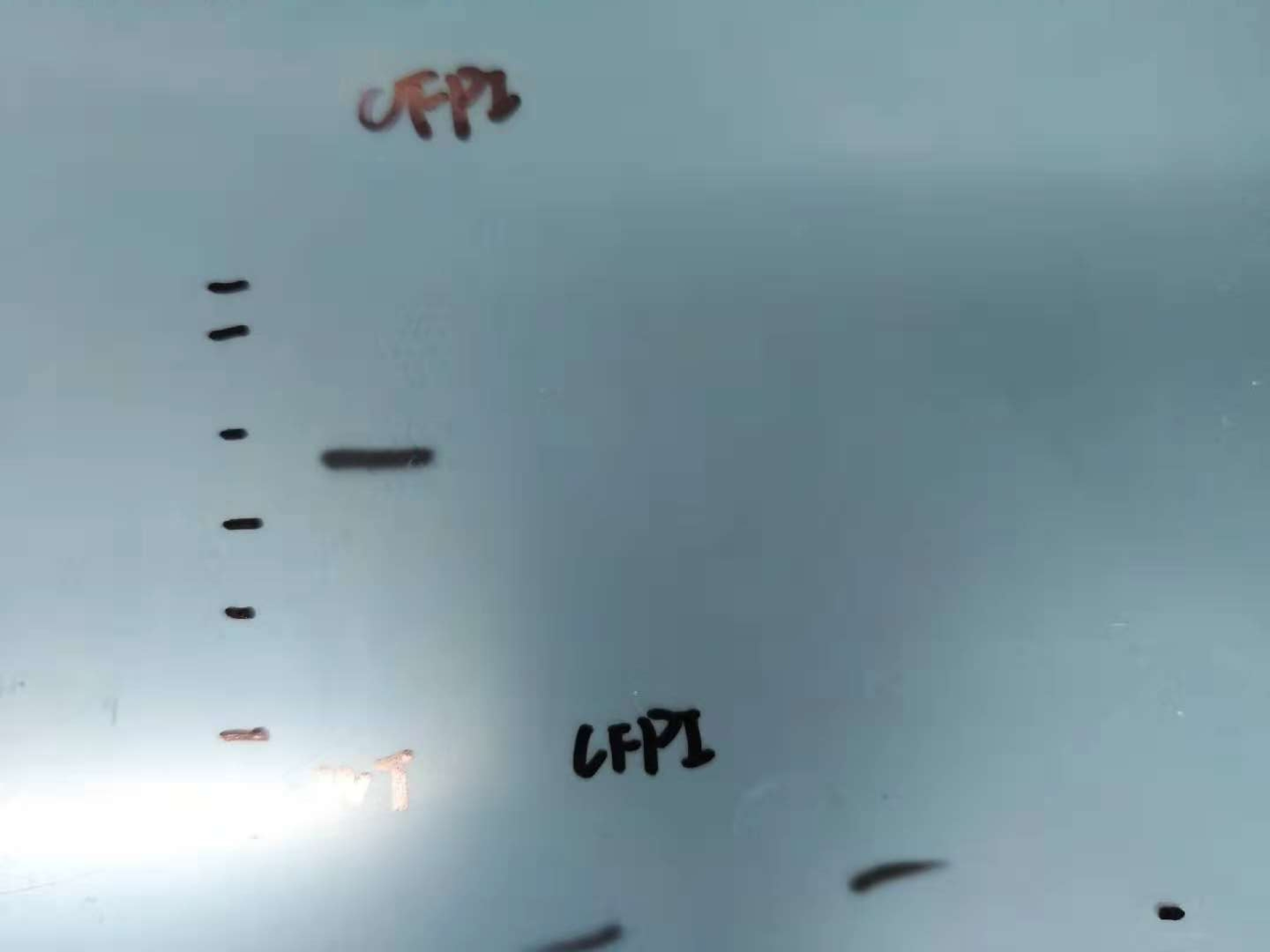
CFP1
WT
CFP1

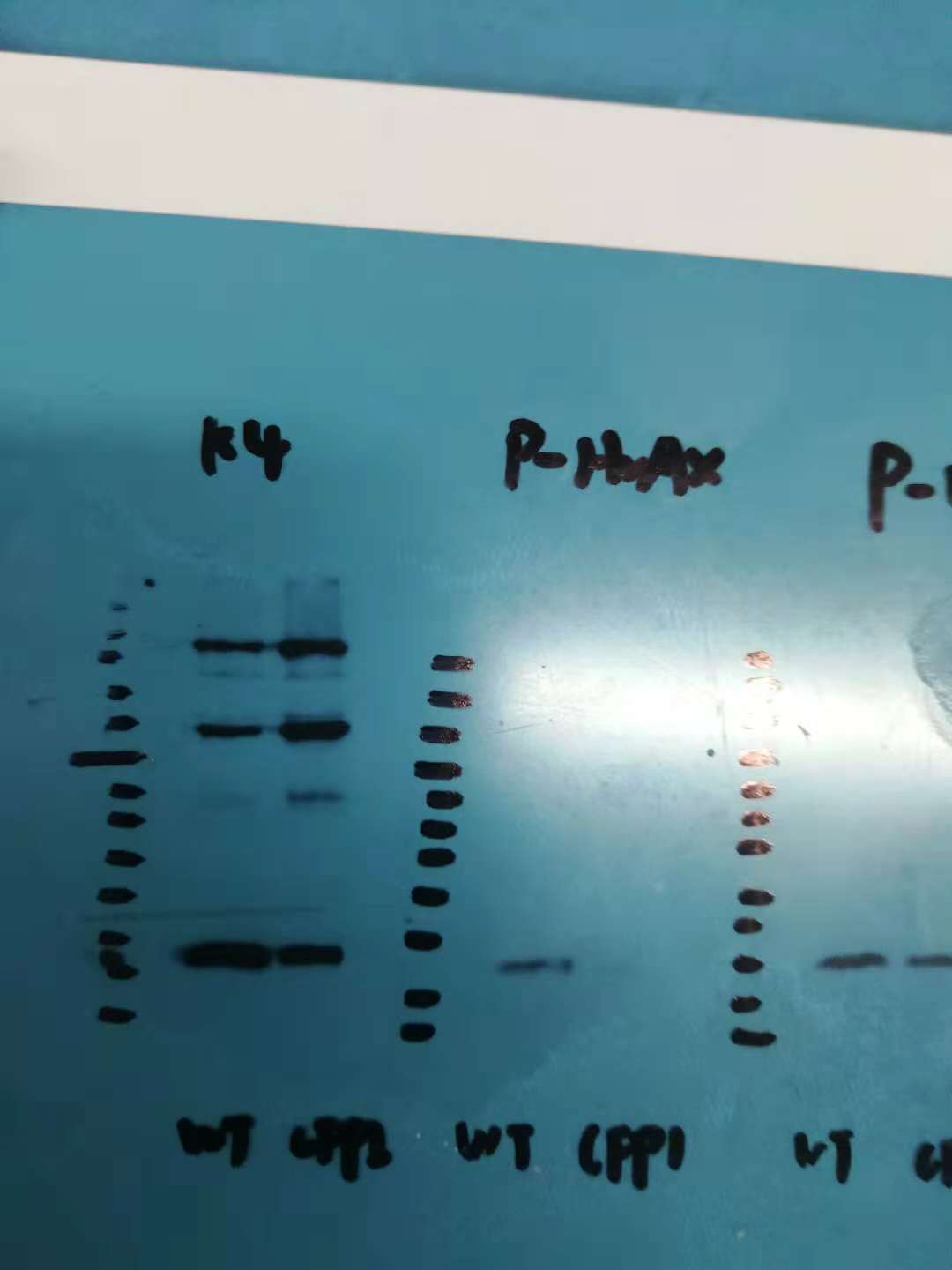
K4
P-H2AX
WT
WT
CPP1
WT

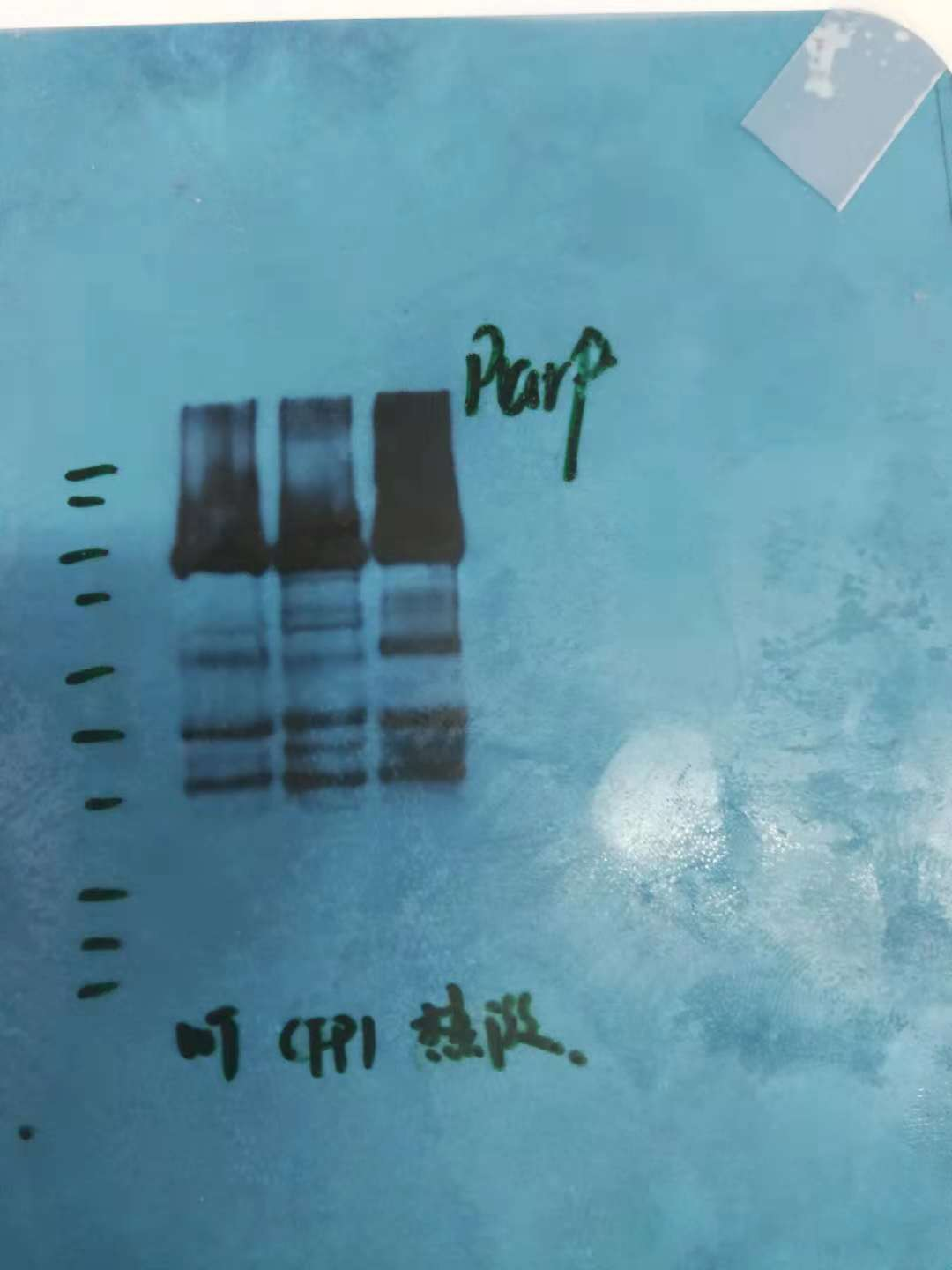
Parp
CFP1 热激.

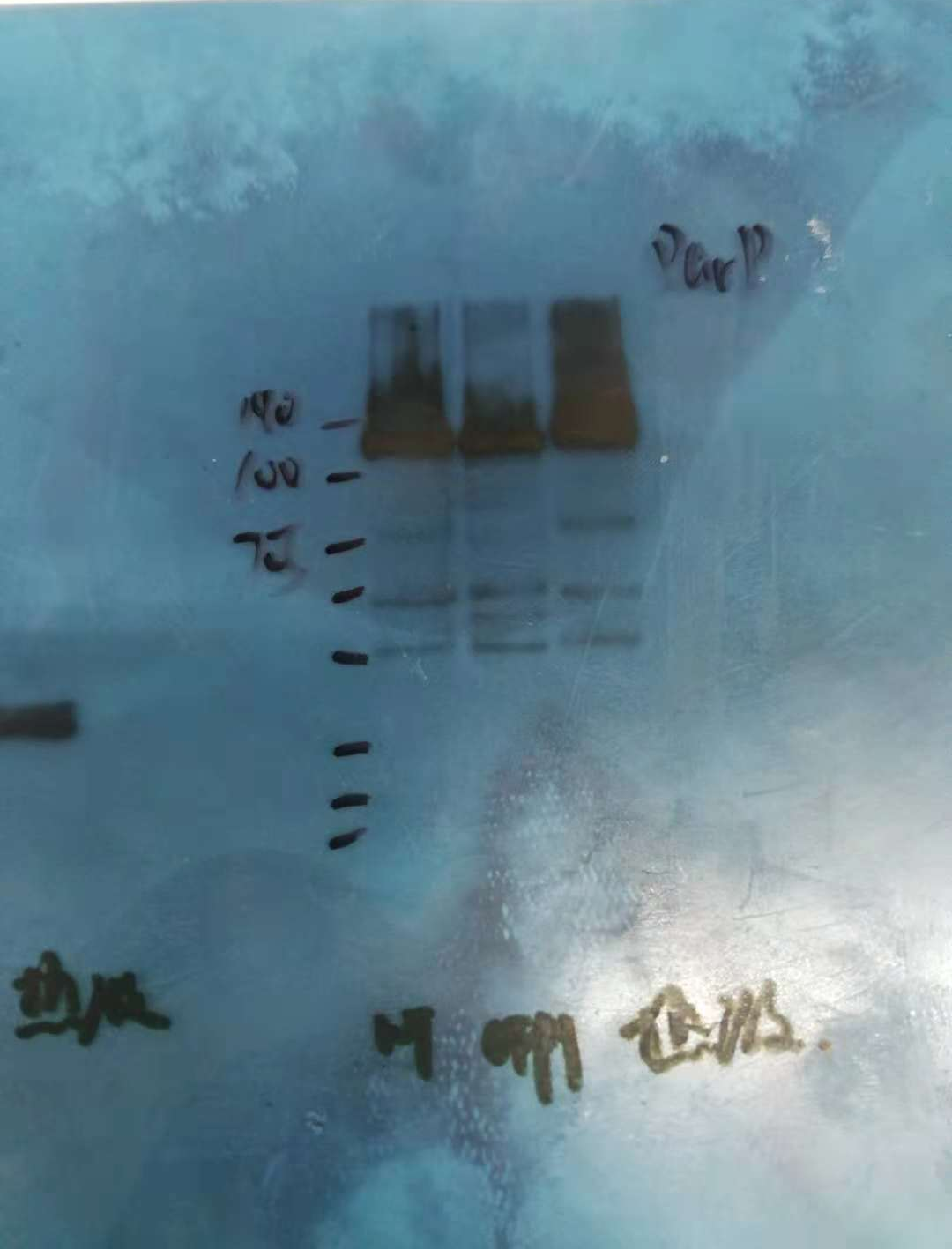

ParP
100
75

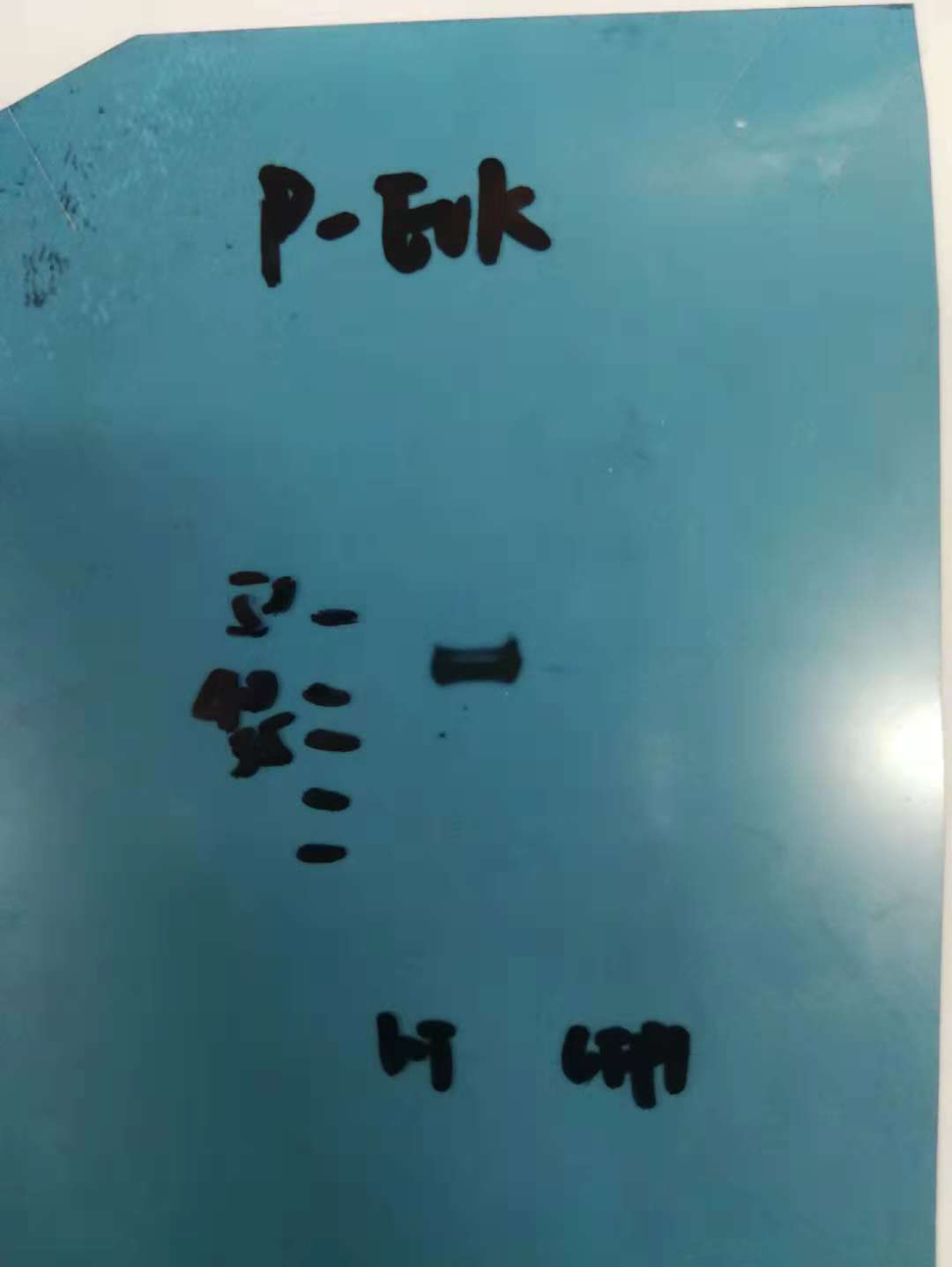
P-Erk

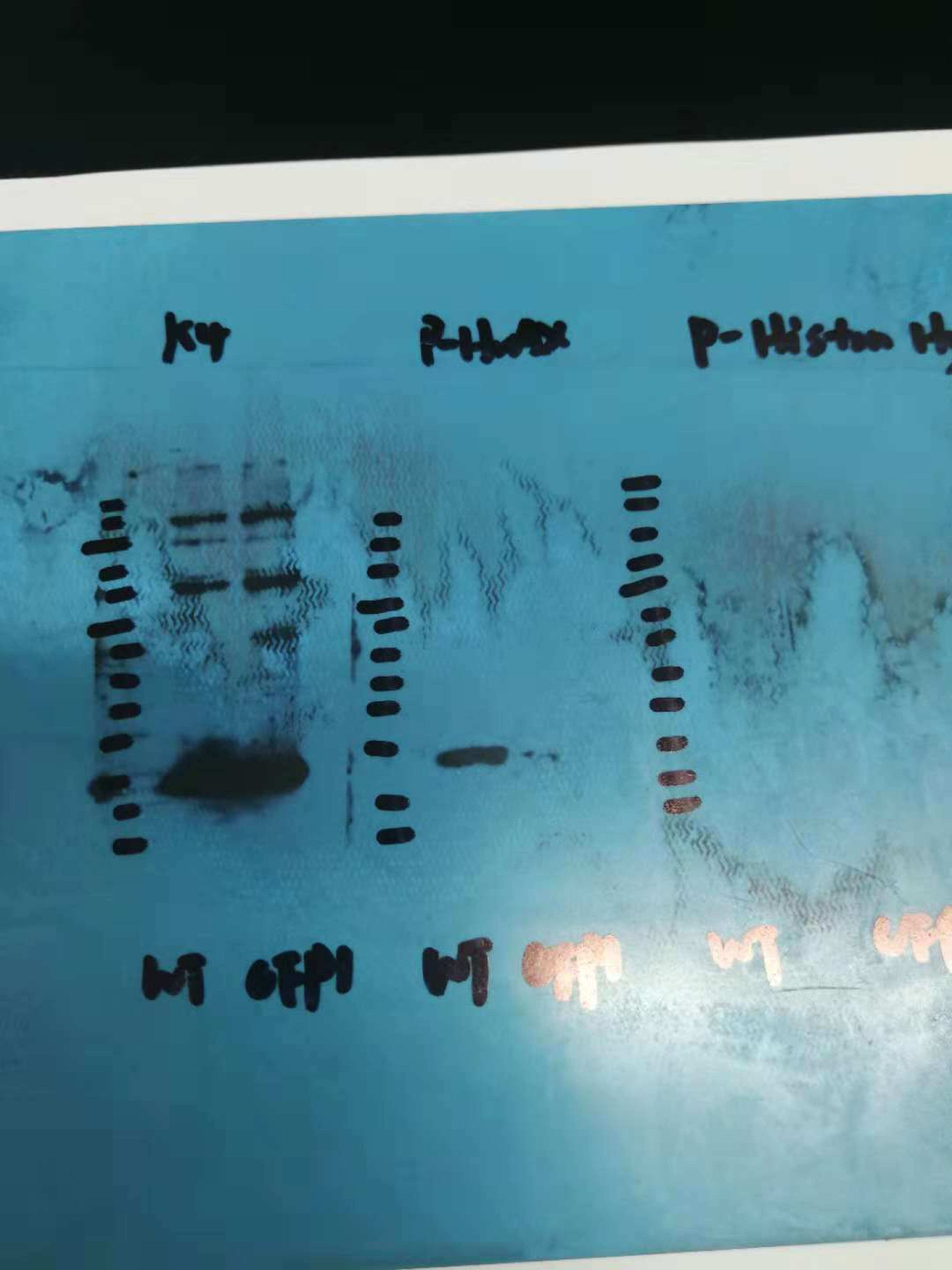
WT
WT
WT

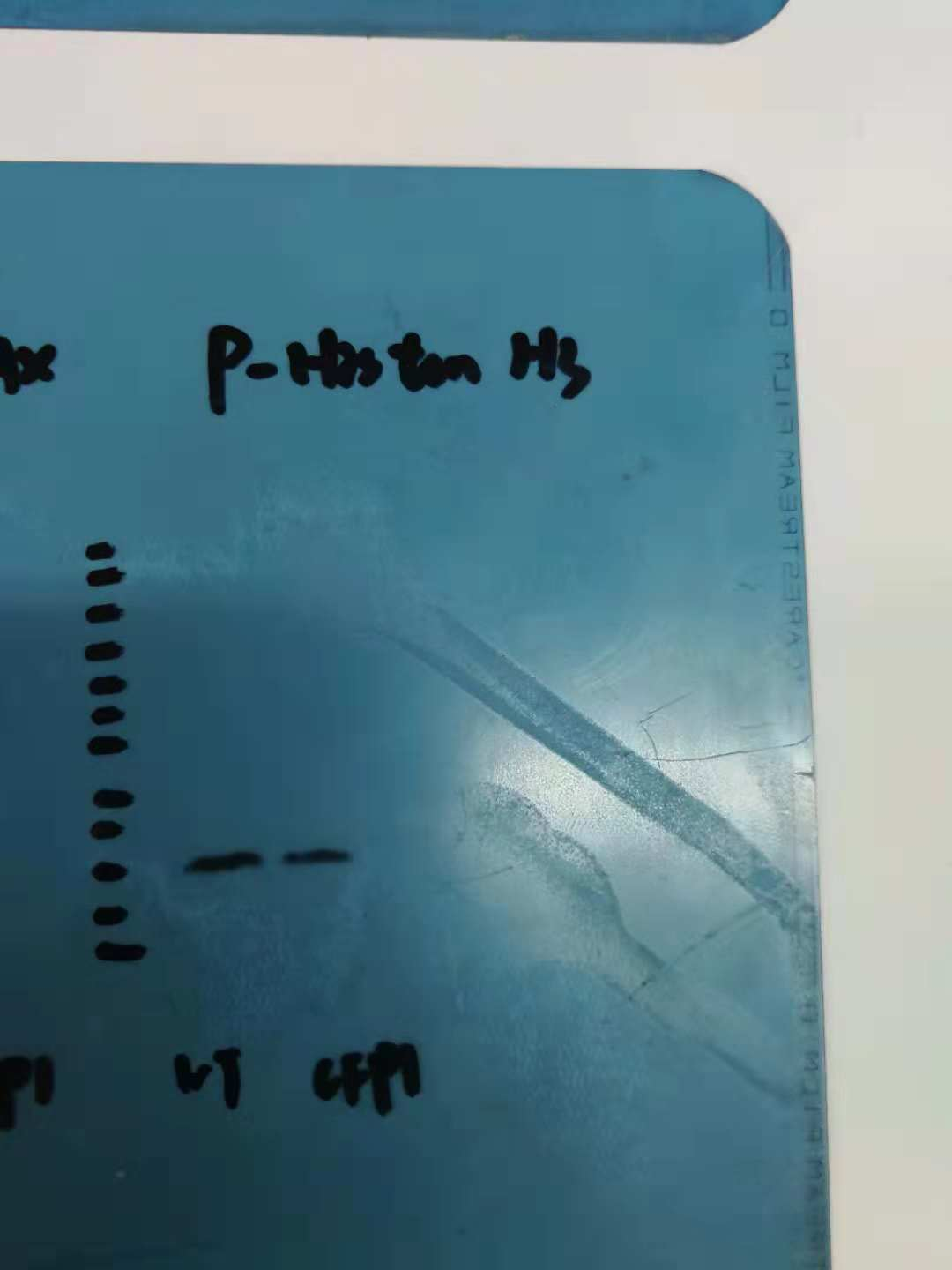
P-Histon H3
WT
CFP1

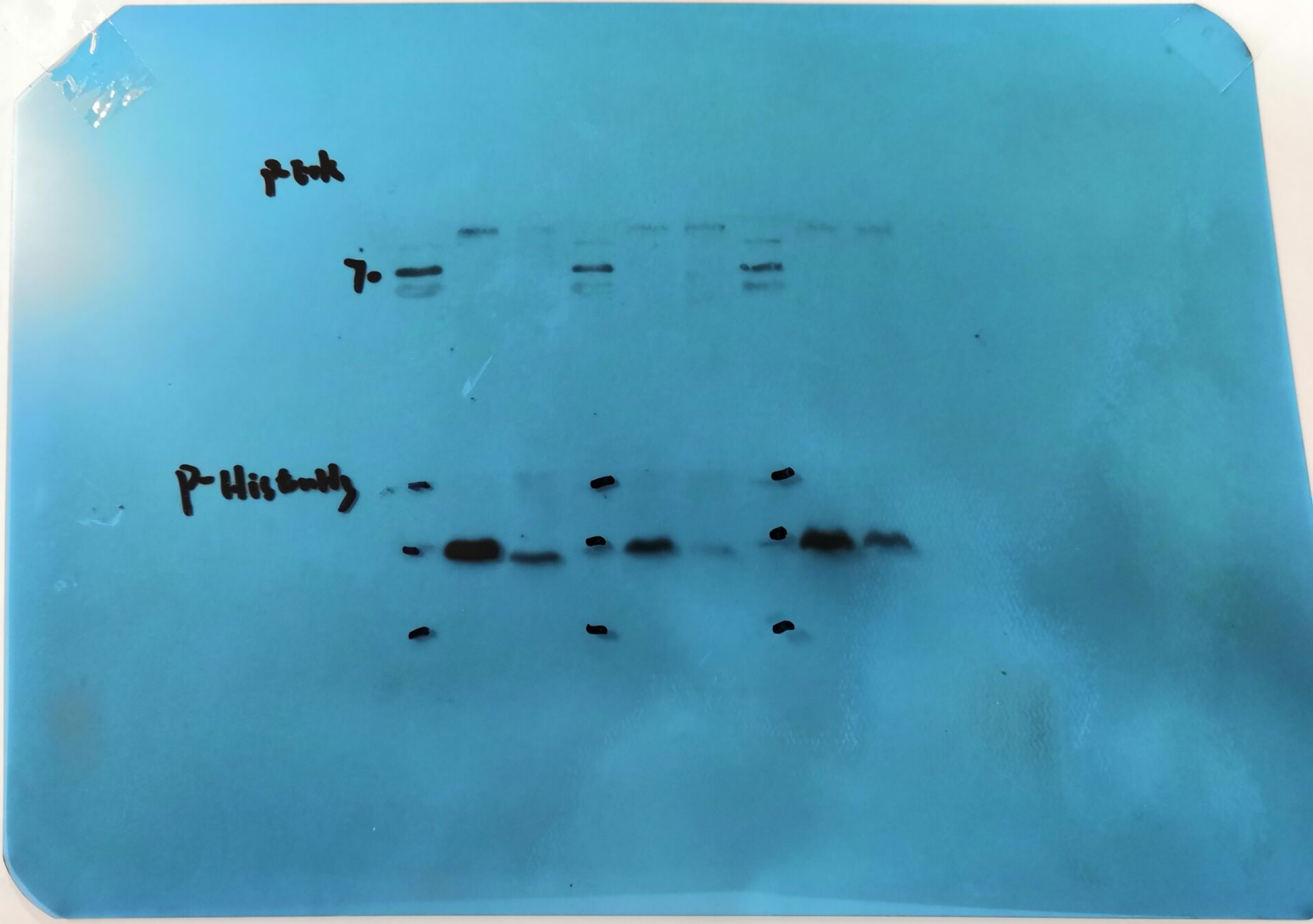

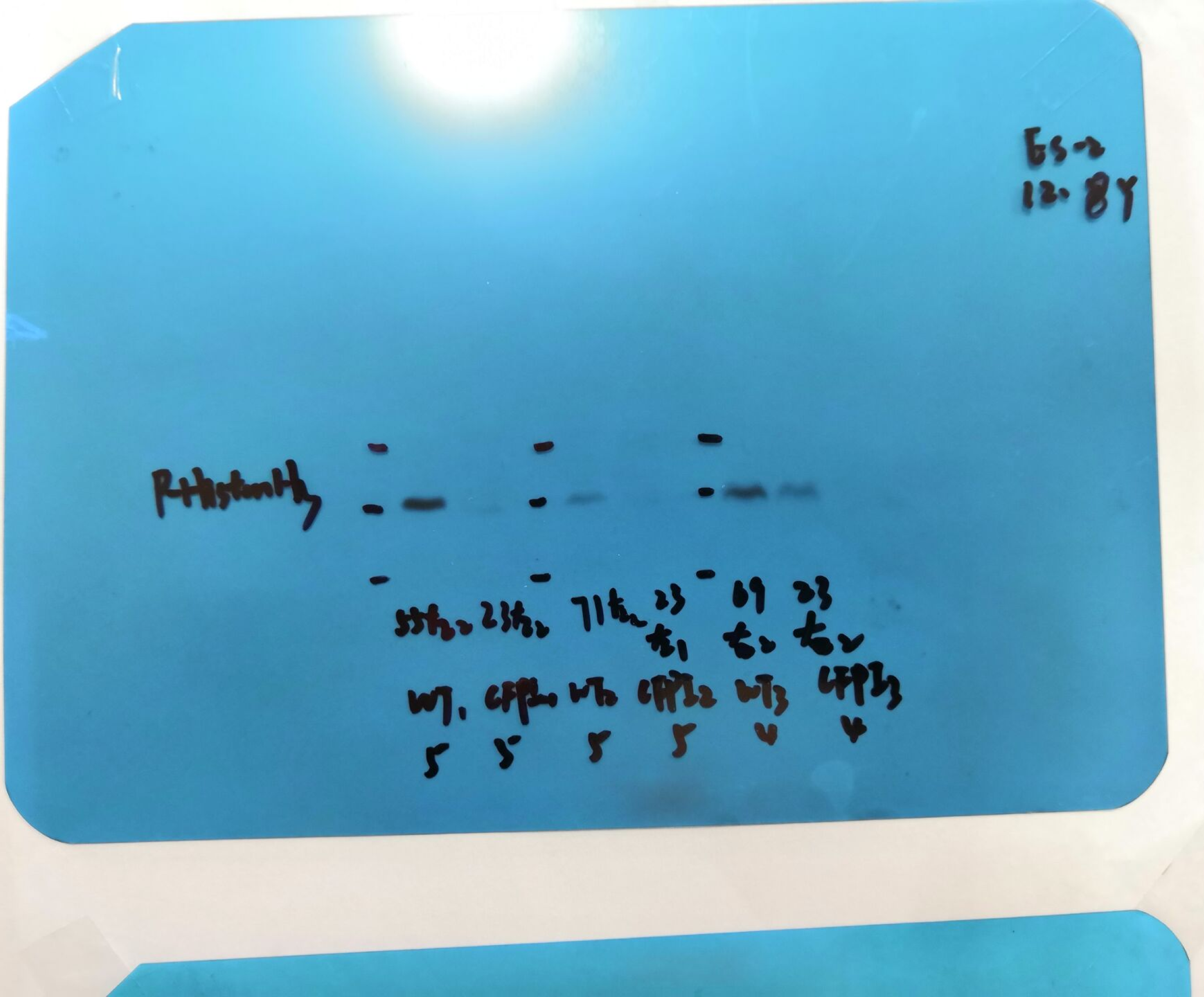

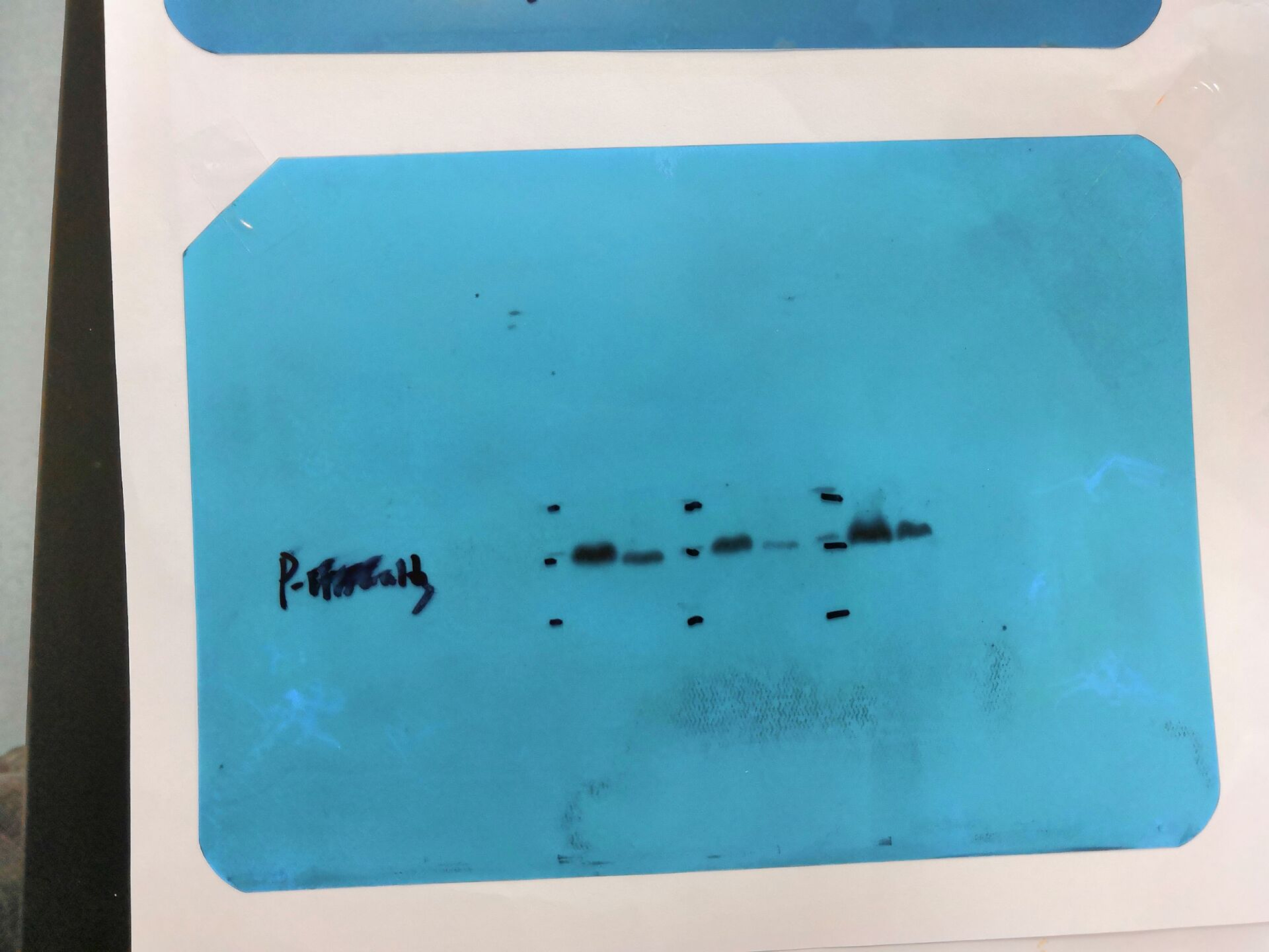

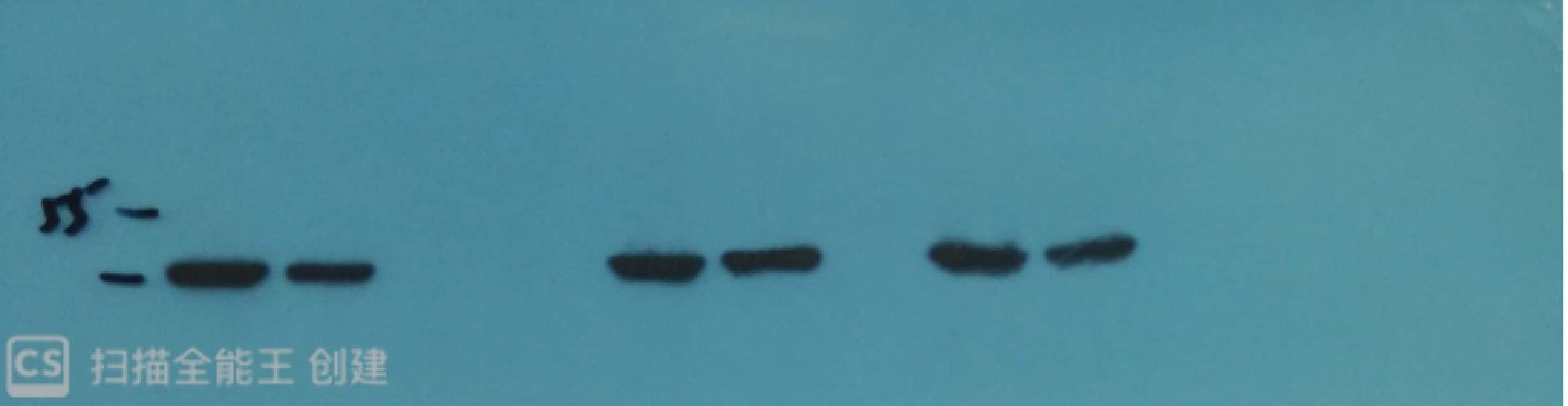

CS 扫描全能王 创建

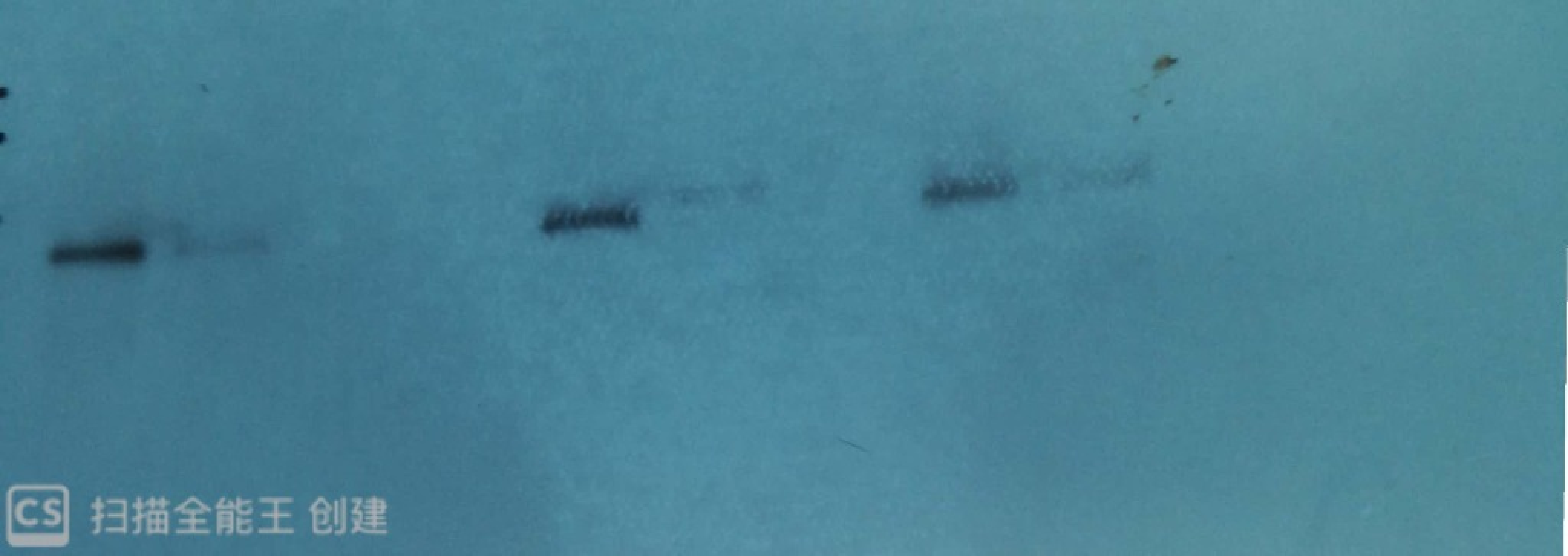

CS 扫描全能王 创建

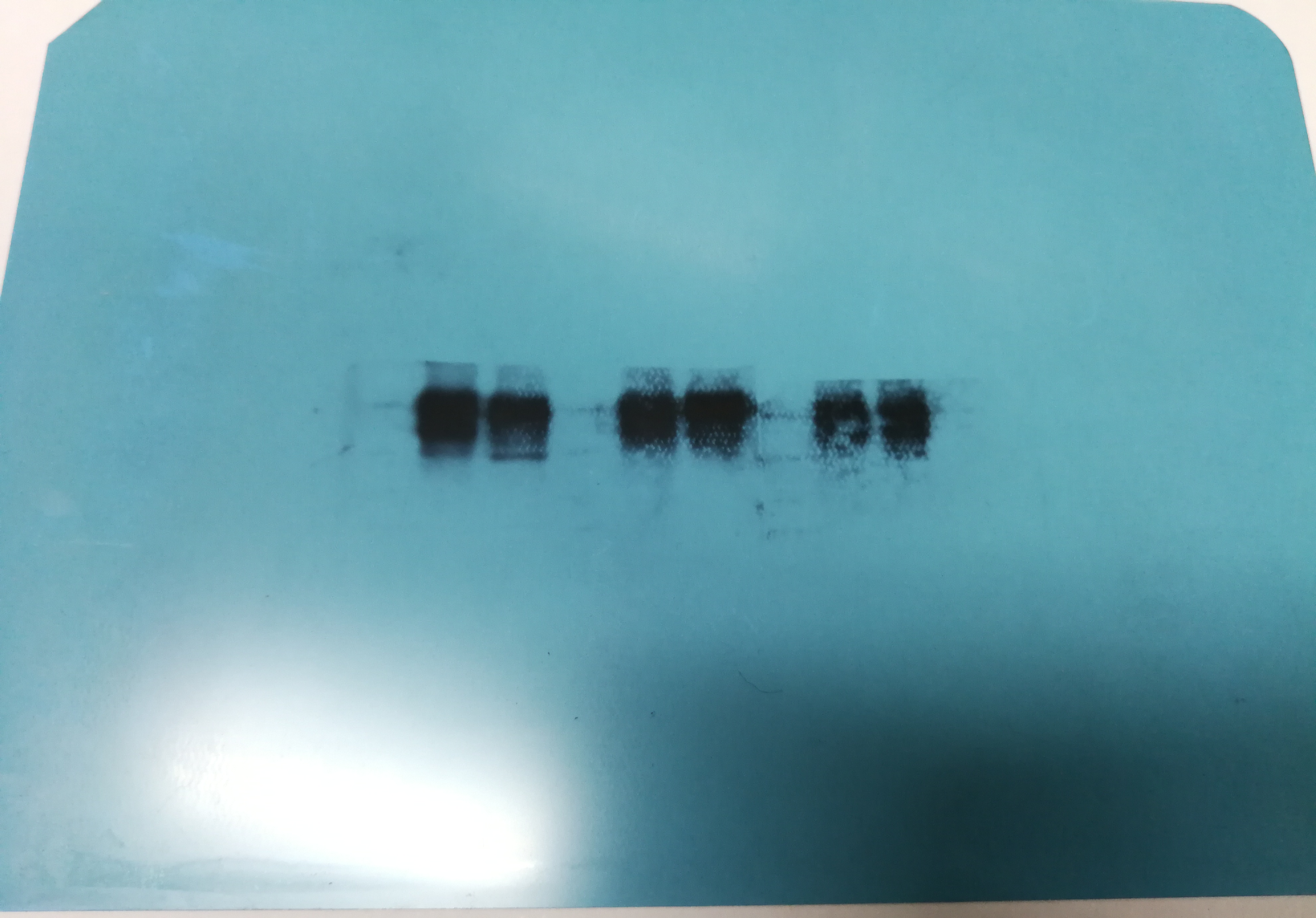

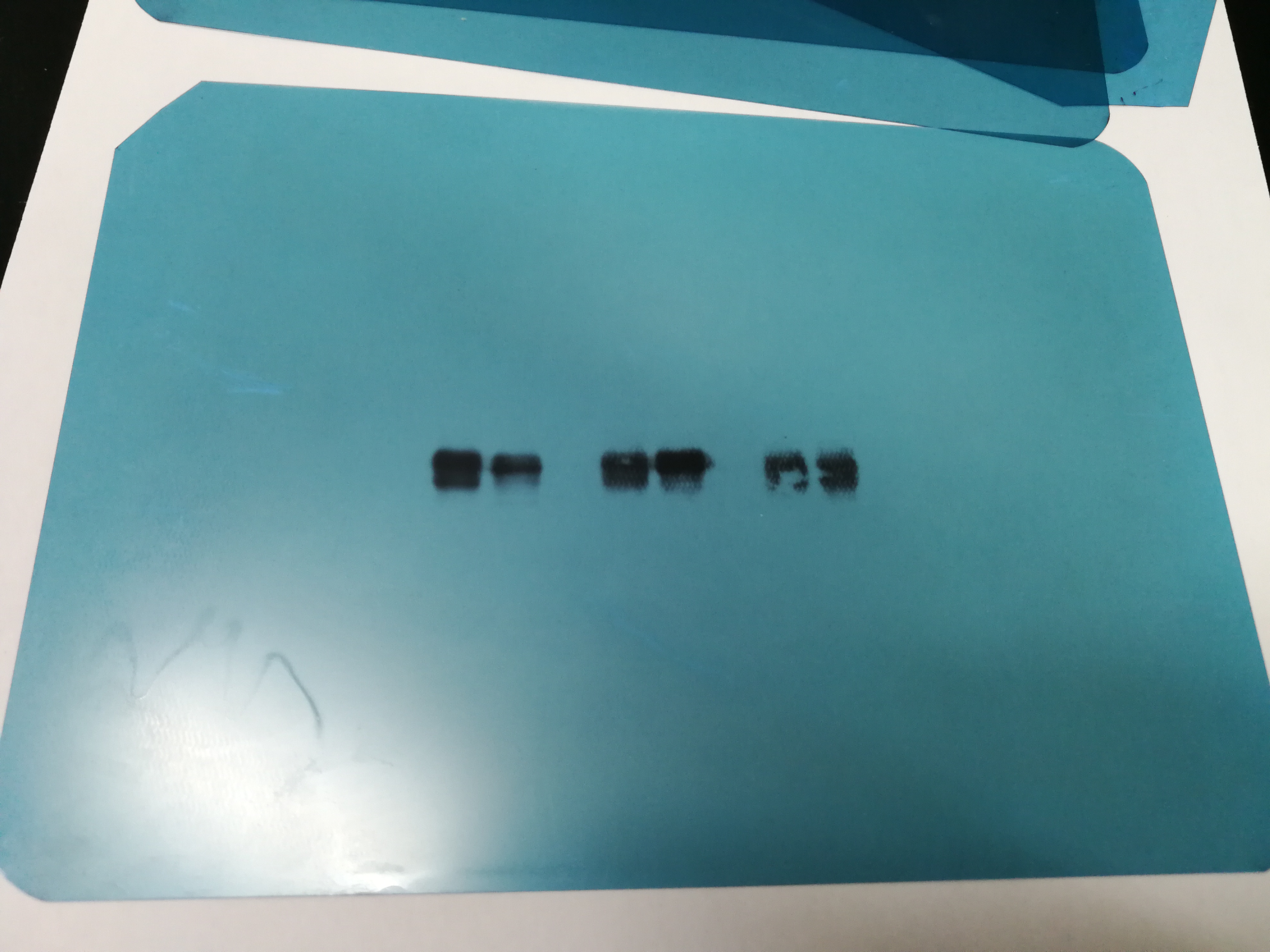

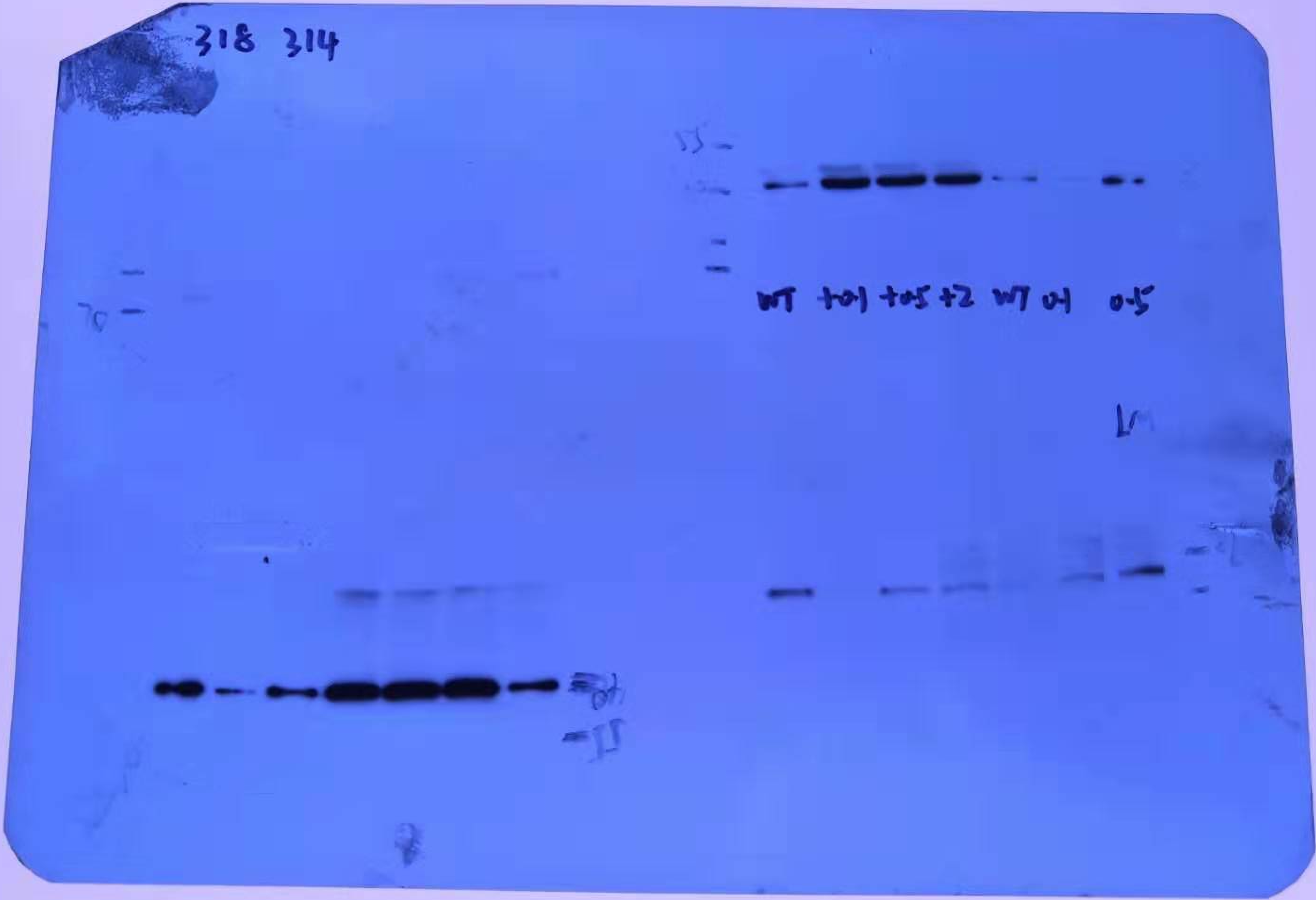
318 314
WT +0.1 +0.5 +2 WT 0.1 0.5

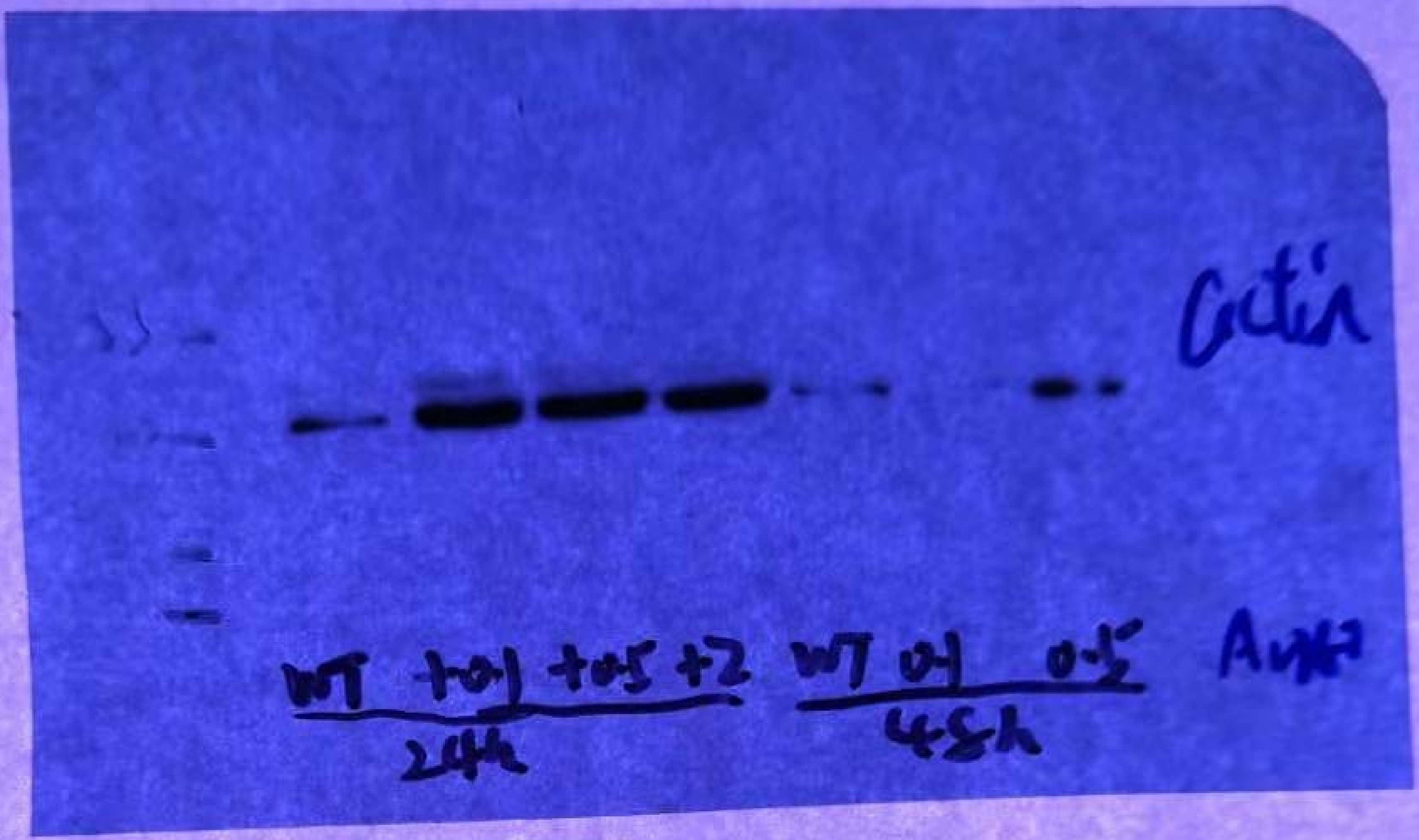
actin
WT +0.1 +0.5 +2 WT 0.1 0.5
24h
48h

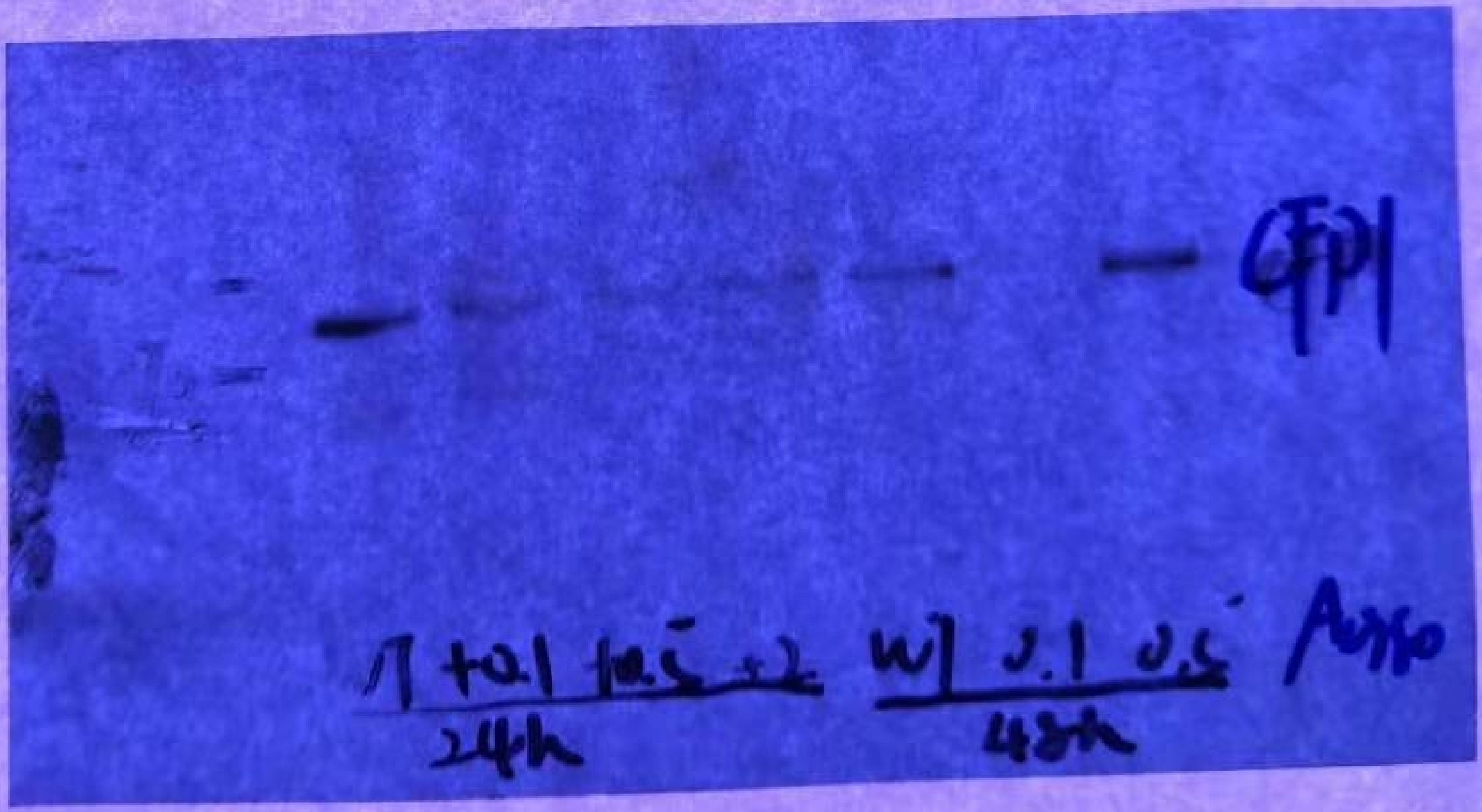
+0.1 +0.5 +2 WT 0.1 0.5
24h
48h

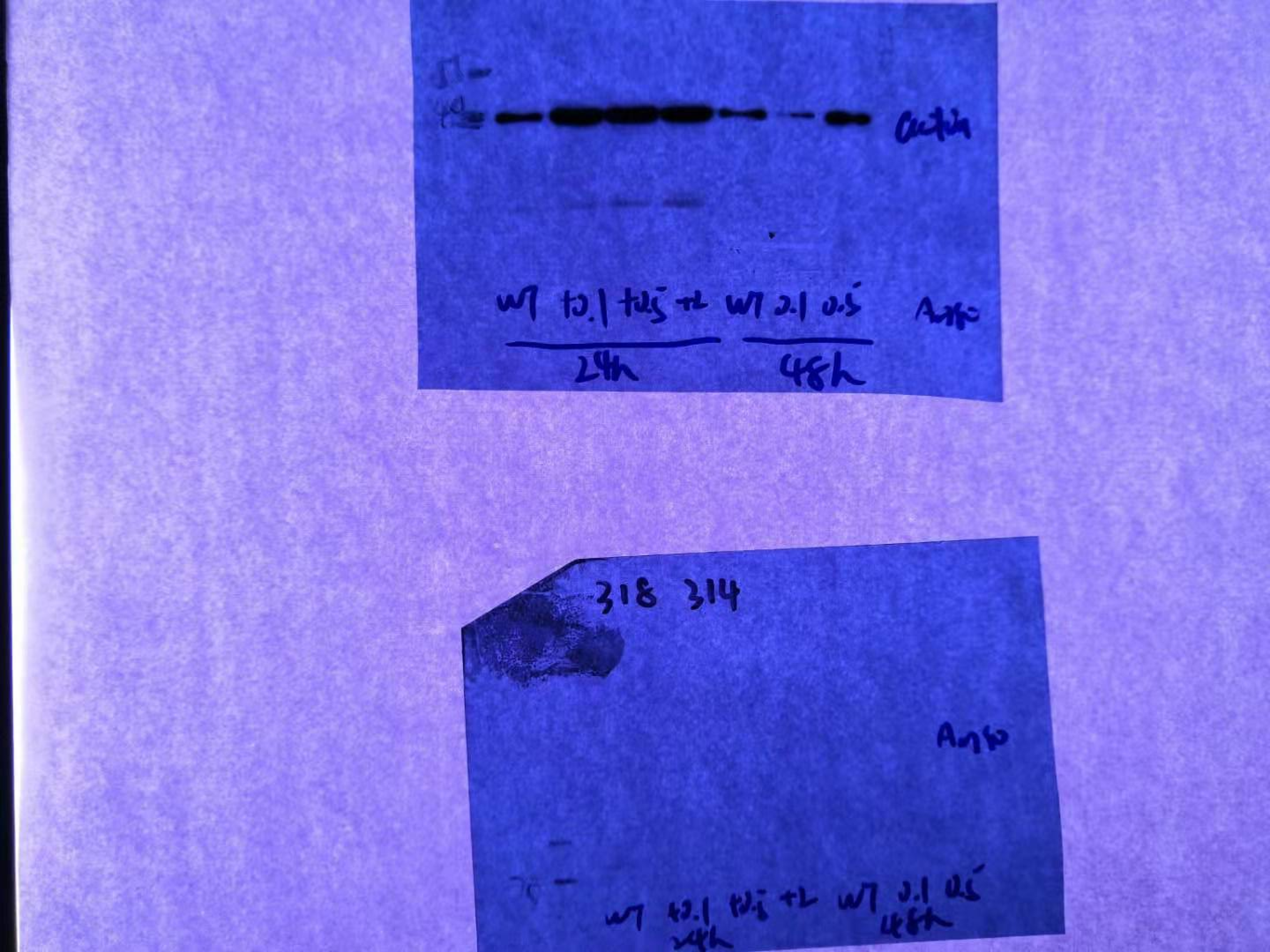
actin
24h
48h
318 314

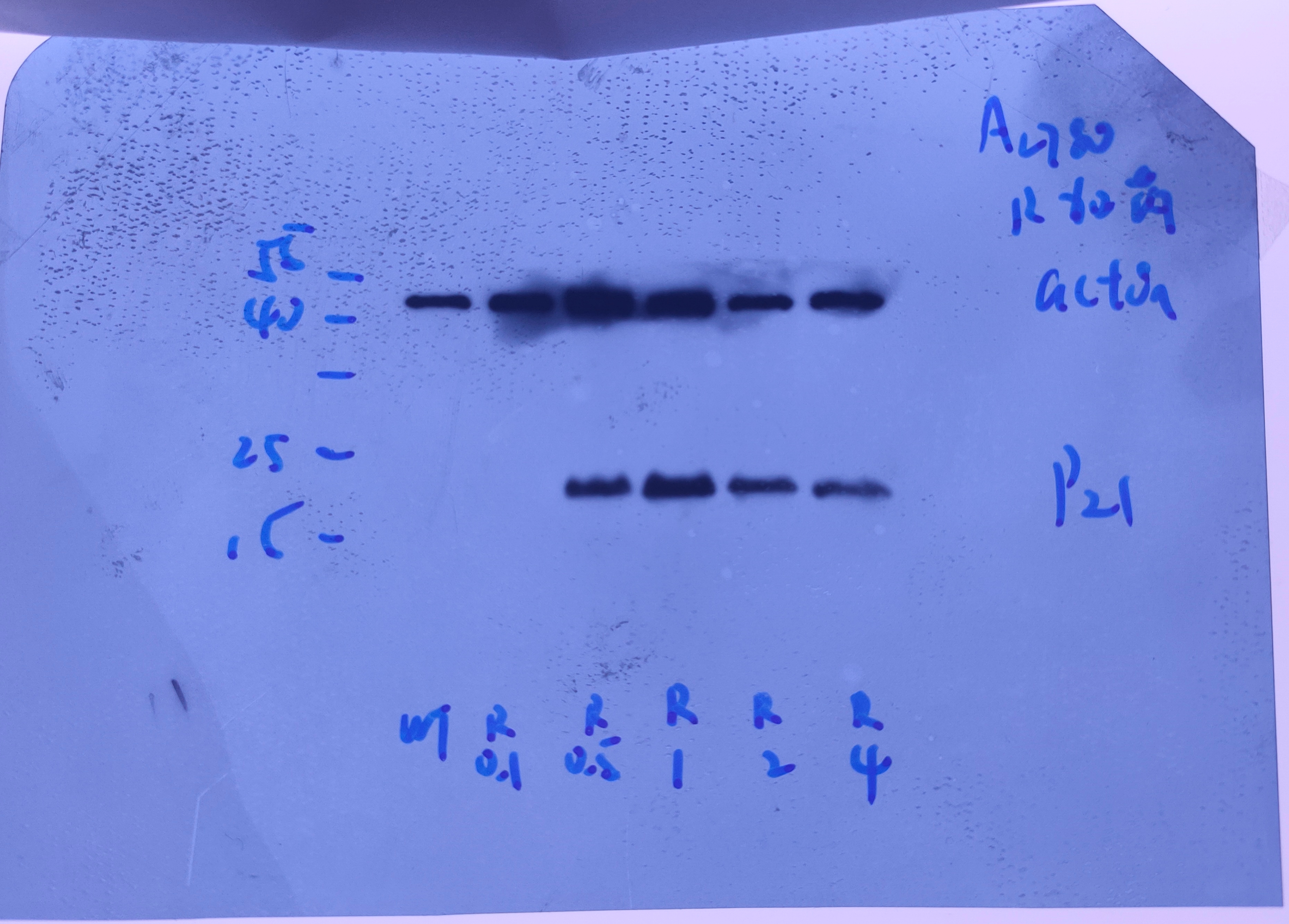
A2780
actin
P21
55
40
25
15
WT
R 0.1
R 0.5
R 1
R 2
R 4

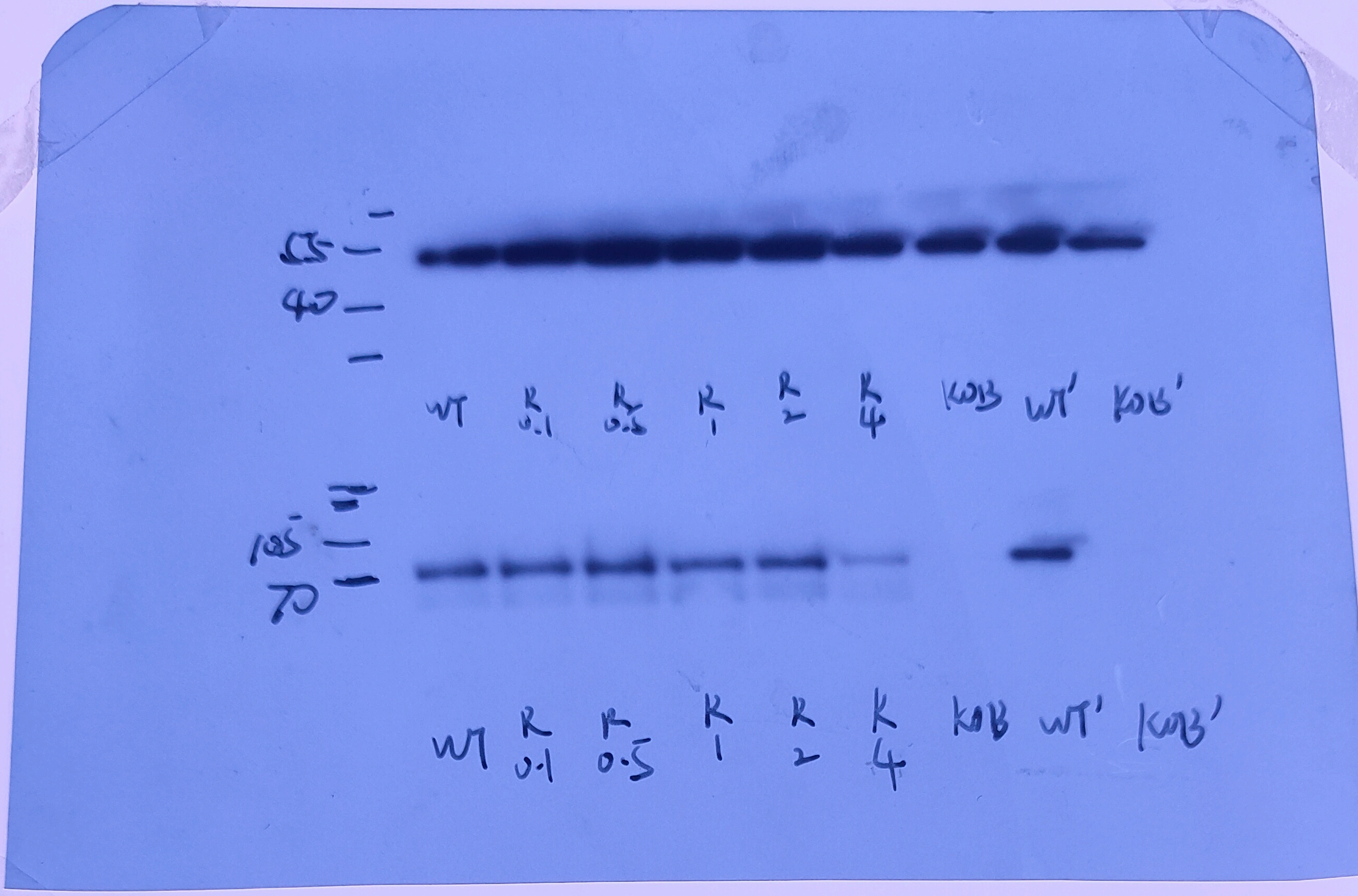
55
40
WT
R 0.1
R 0.5
R 1
R 2
R 4
KOB
WT'
KOB'
105
70
WT
R 0.1
R 0.5
R 1
R 2
R 4
KOB
WT'
KOB'

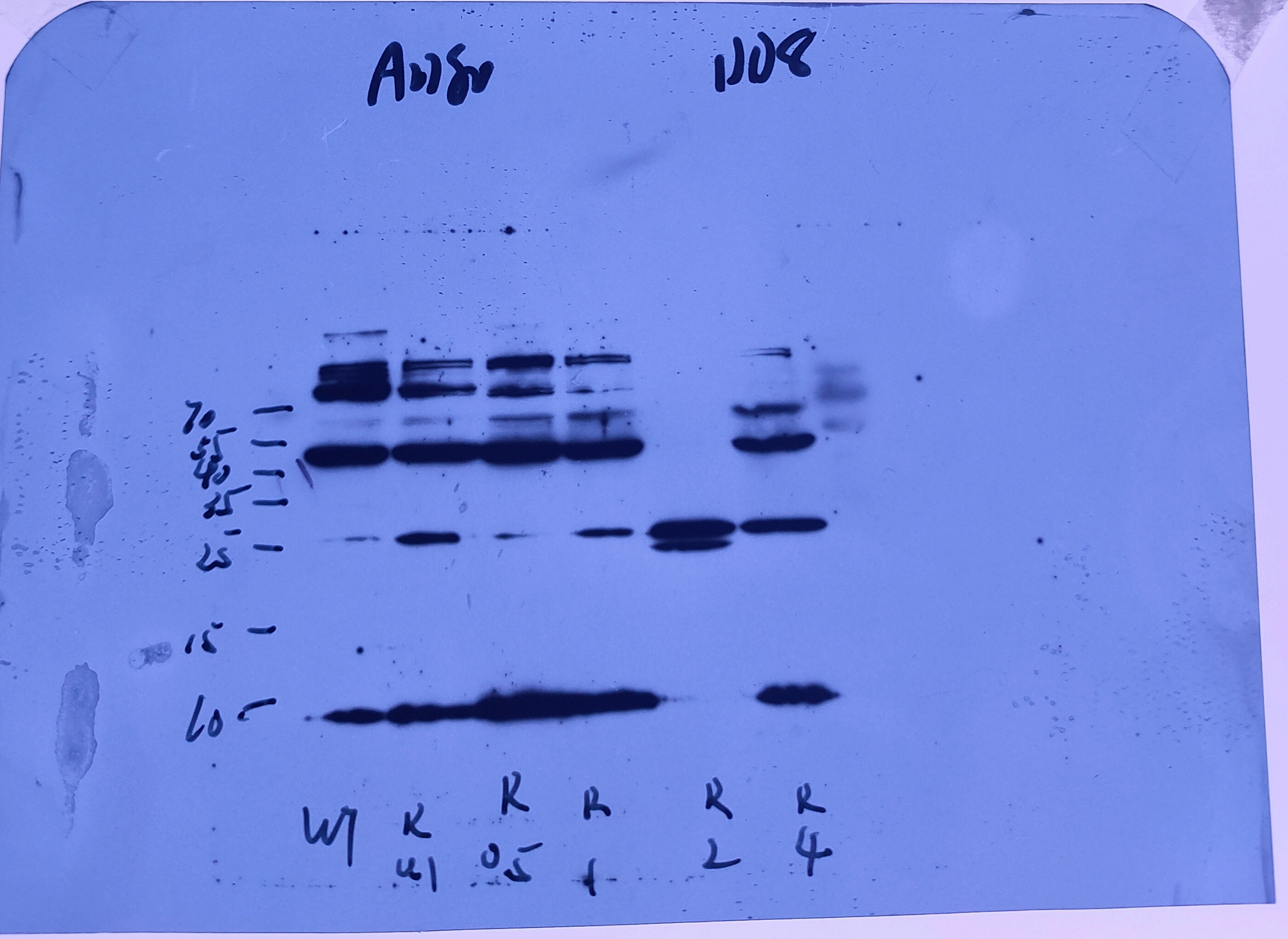
70
55
40
35
25
15
10
WT
R 0.1
R 0.5
R 1
R 2
R 4

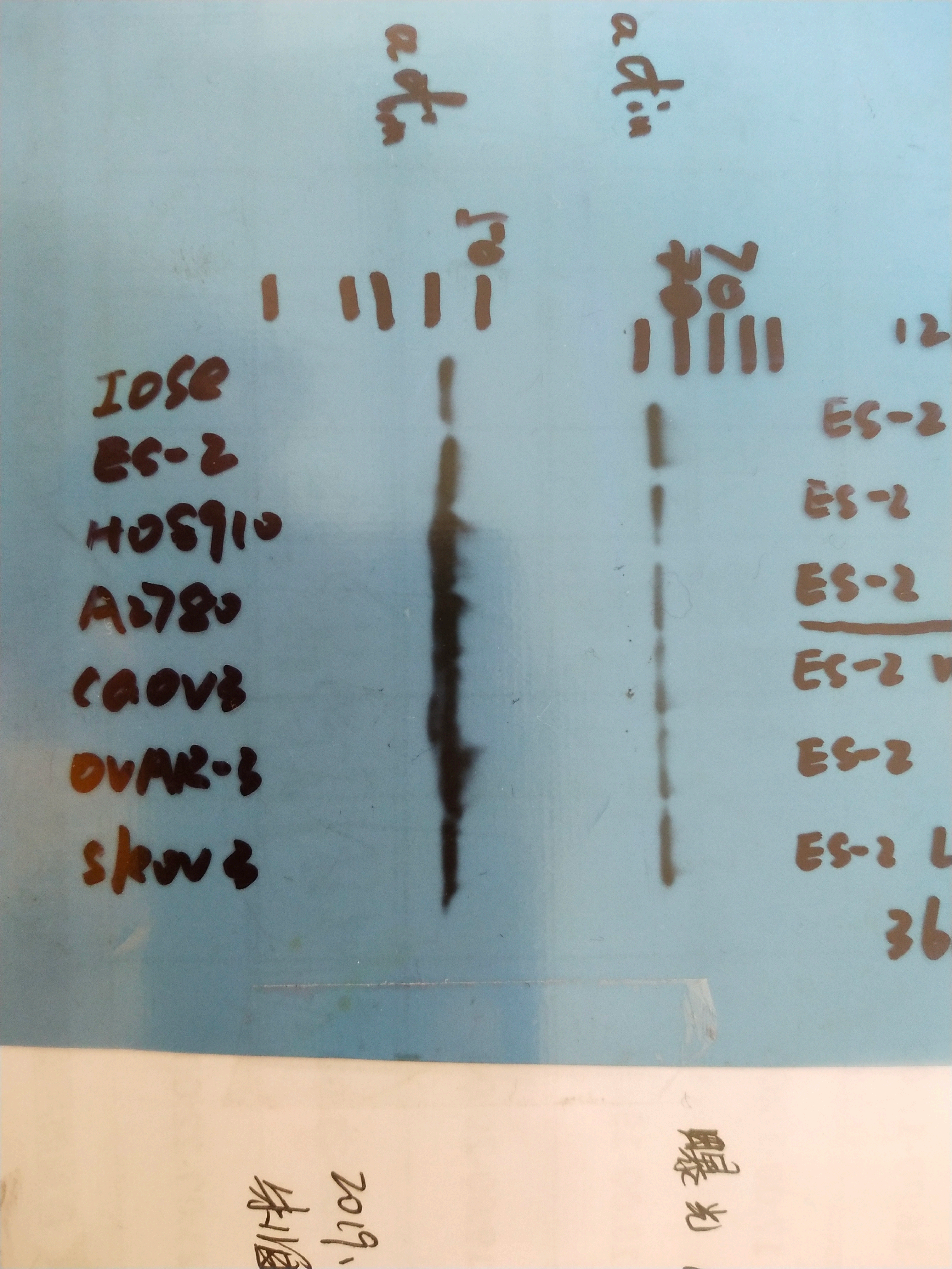
IOSE
ES-2
HO8910
A2780
CAOV3
OVAR-3
SKOV3
ES-2
ES-2
ES-2
ES-2
ES-2
ES-2
36

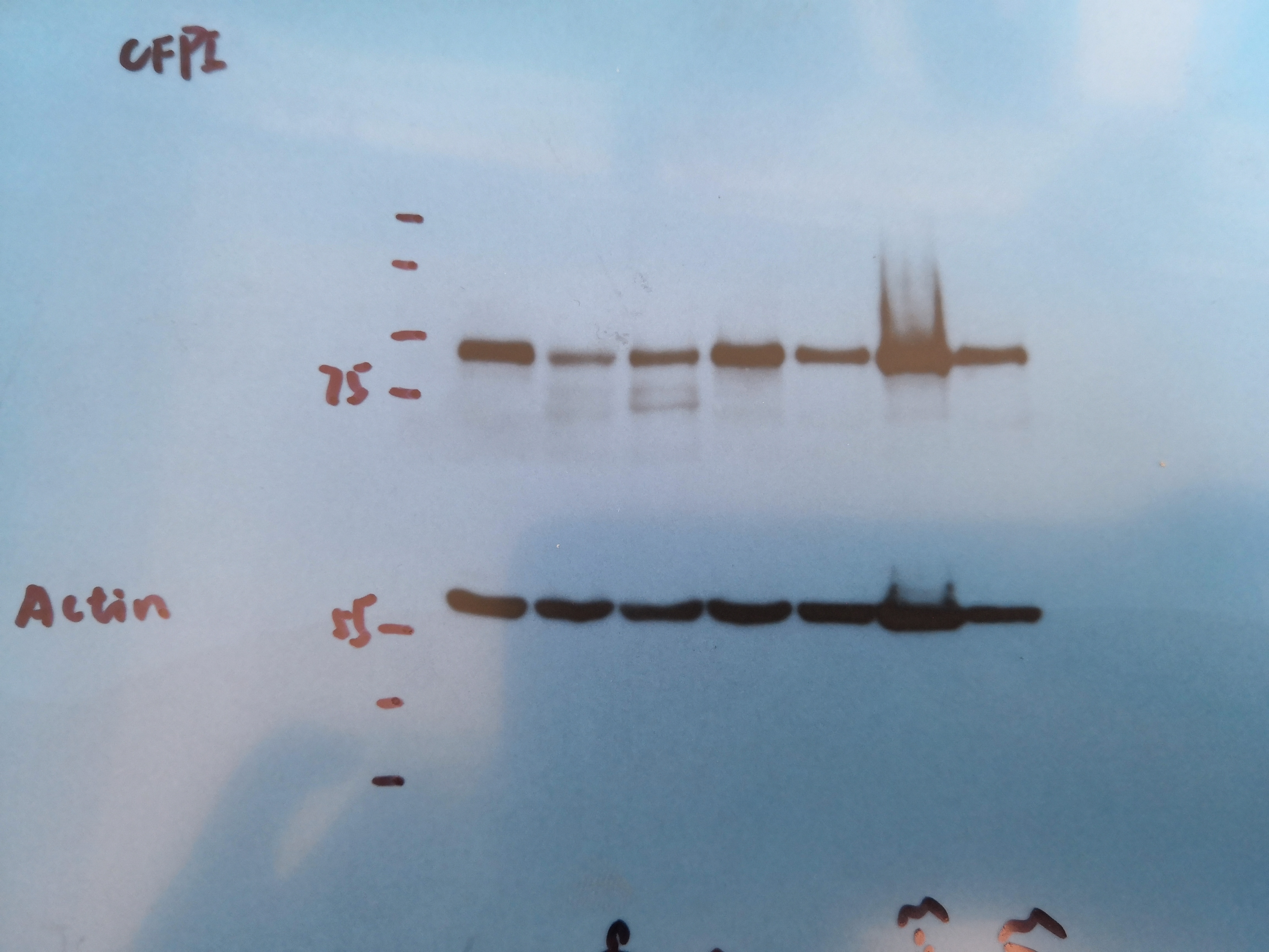

75
Actin
55

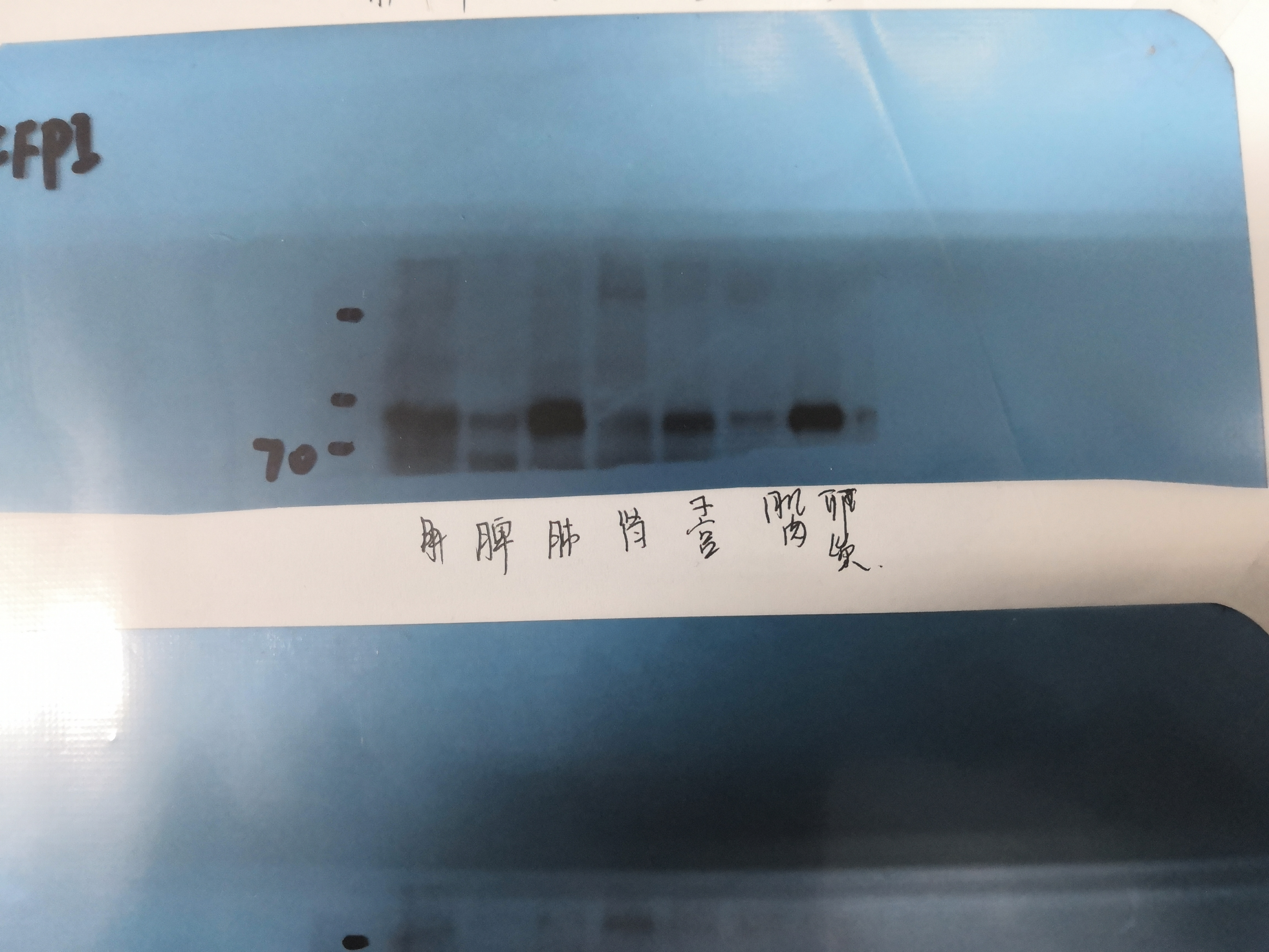
FP1
70

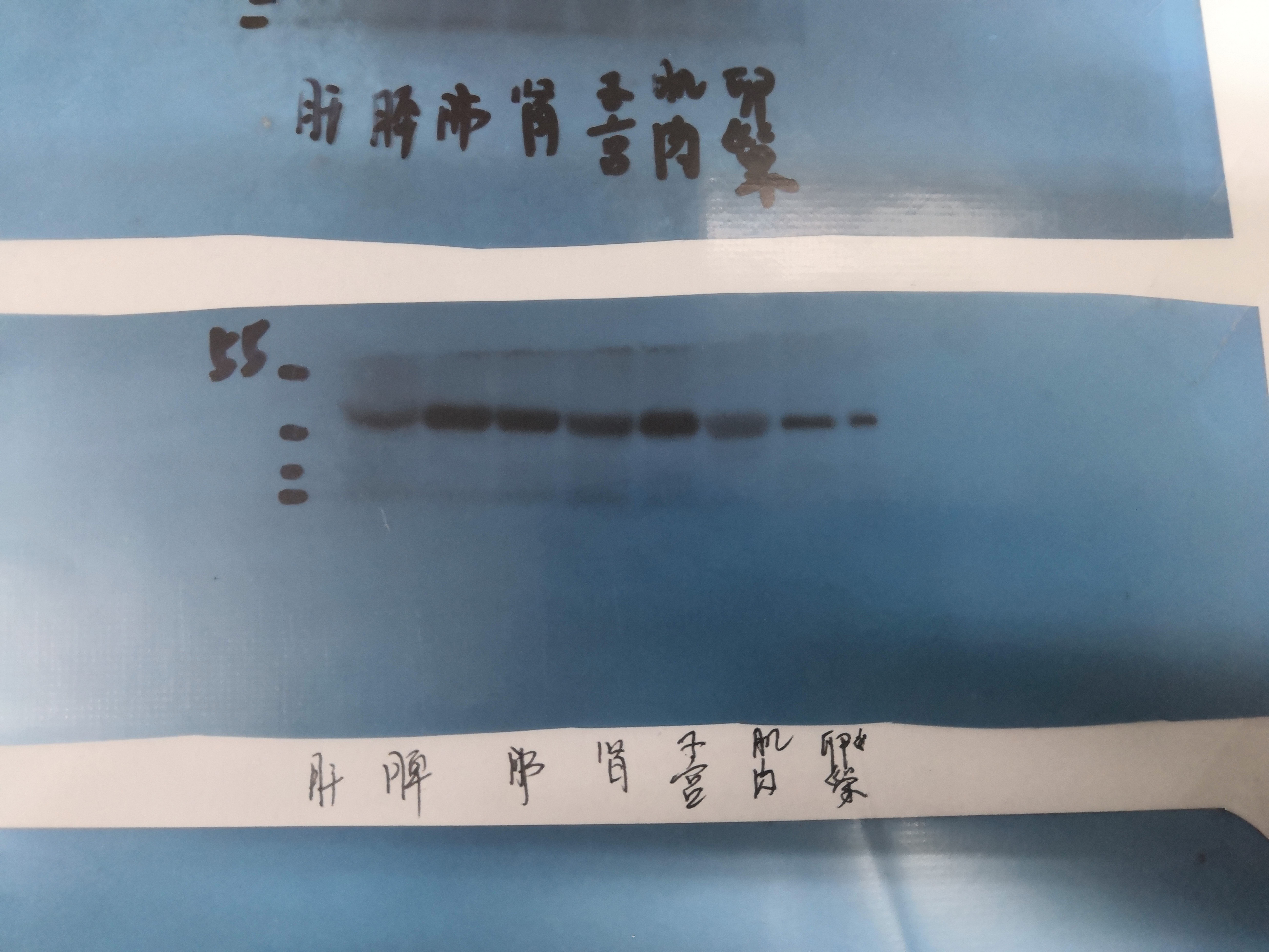
肝 脾 肺 肾 子宫 肌肉 卵巢
55
肝 脾 肺 肾 子宫 肌肉 卵巢

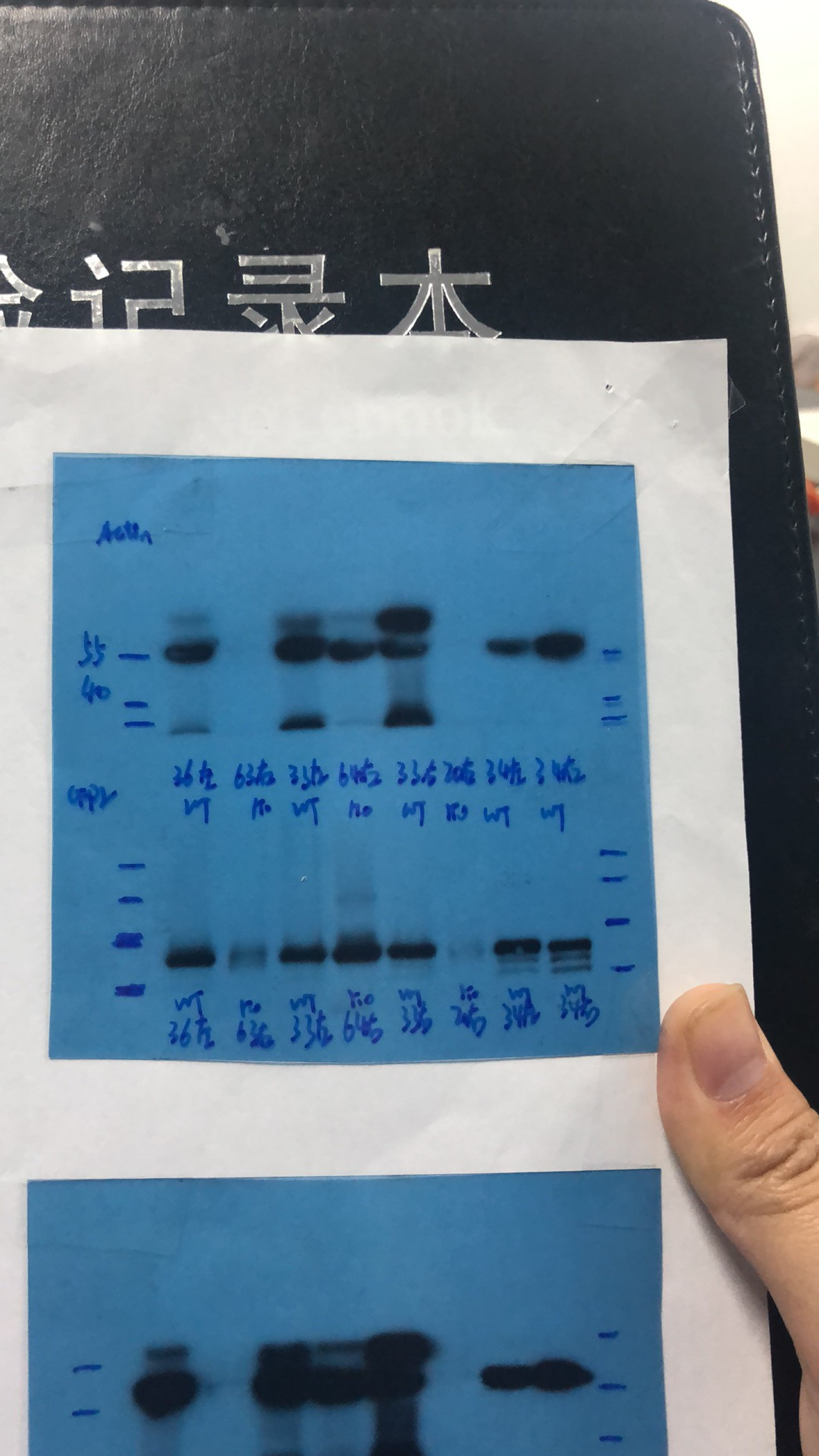
记录本
Actin
55
40
WT
KO

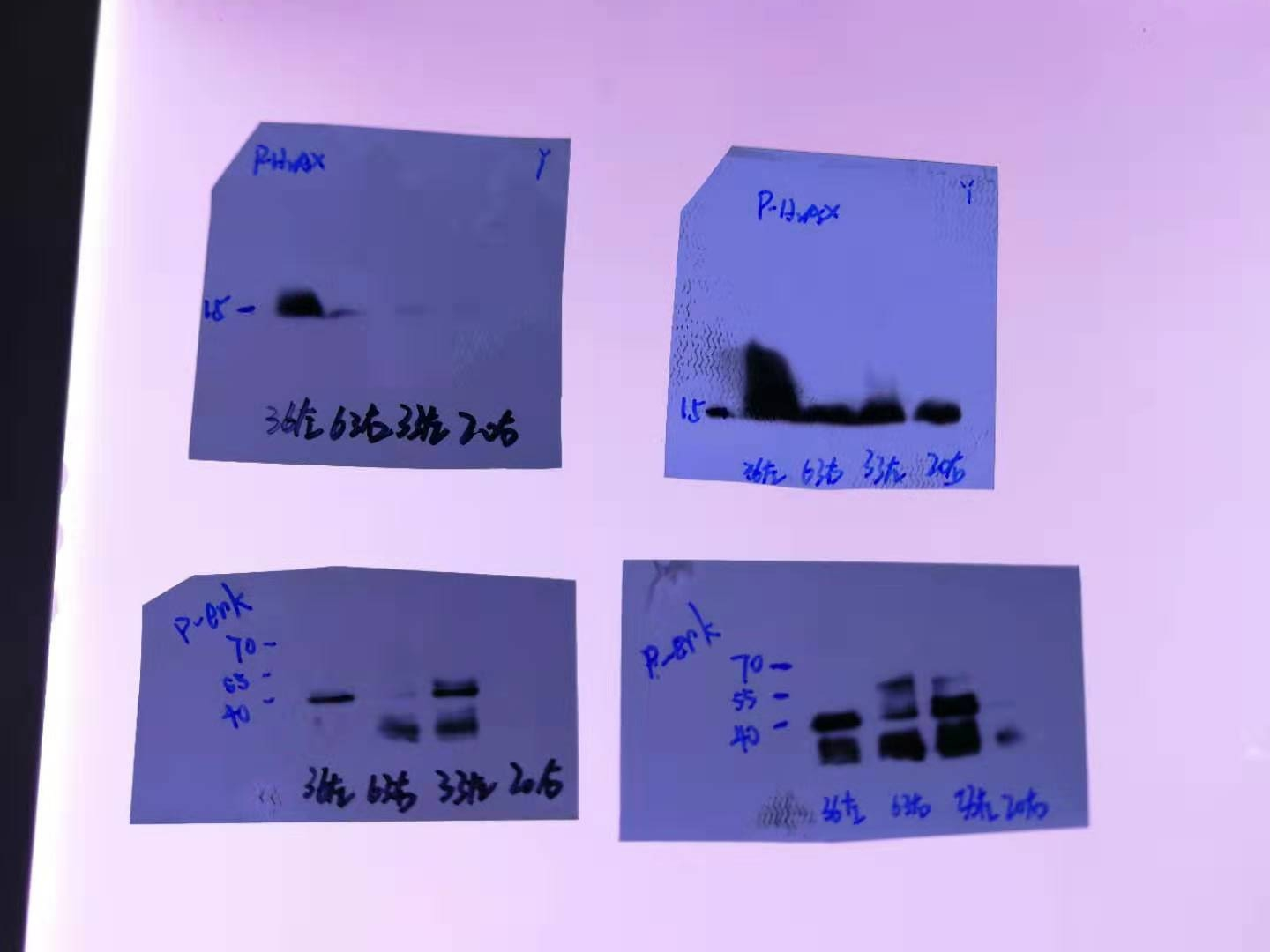
P-erk
70
40
15

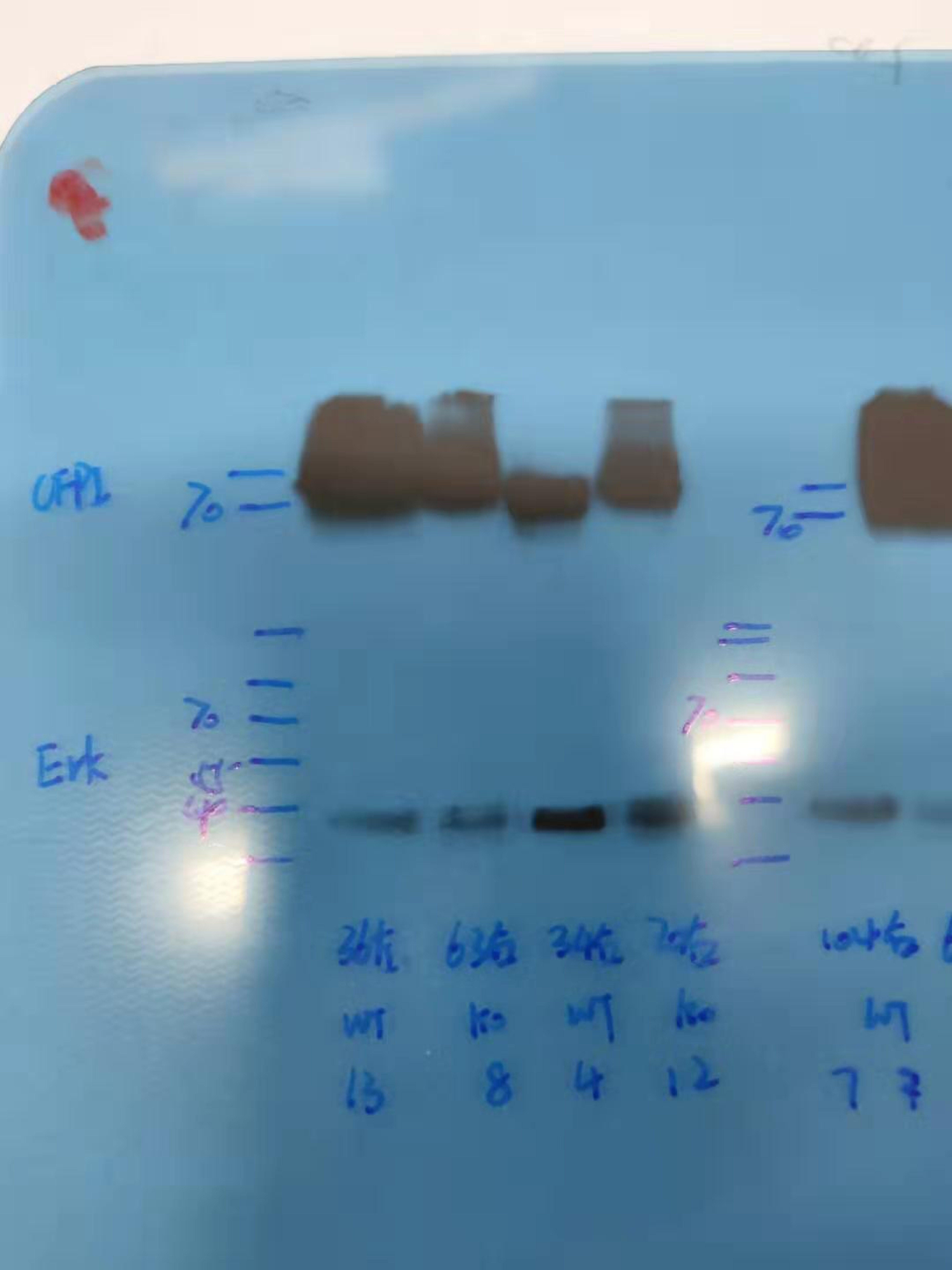

70
70
Erk
70
70
WT
KO
WT
KO
WT
13
8
4
12
7
7

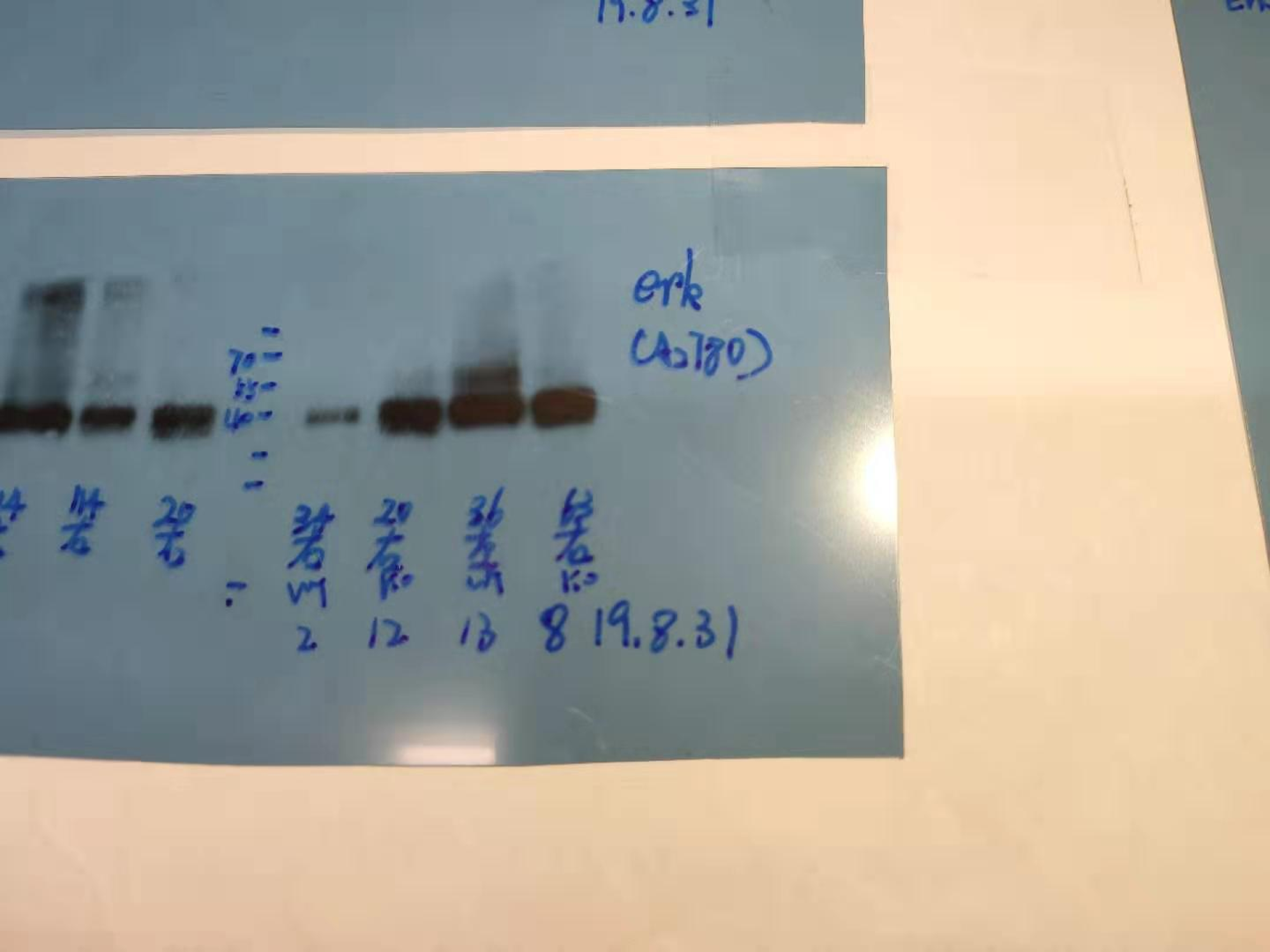
erk
(A2780)
70
40
2 12 13 8 19.8.31

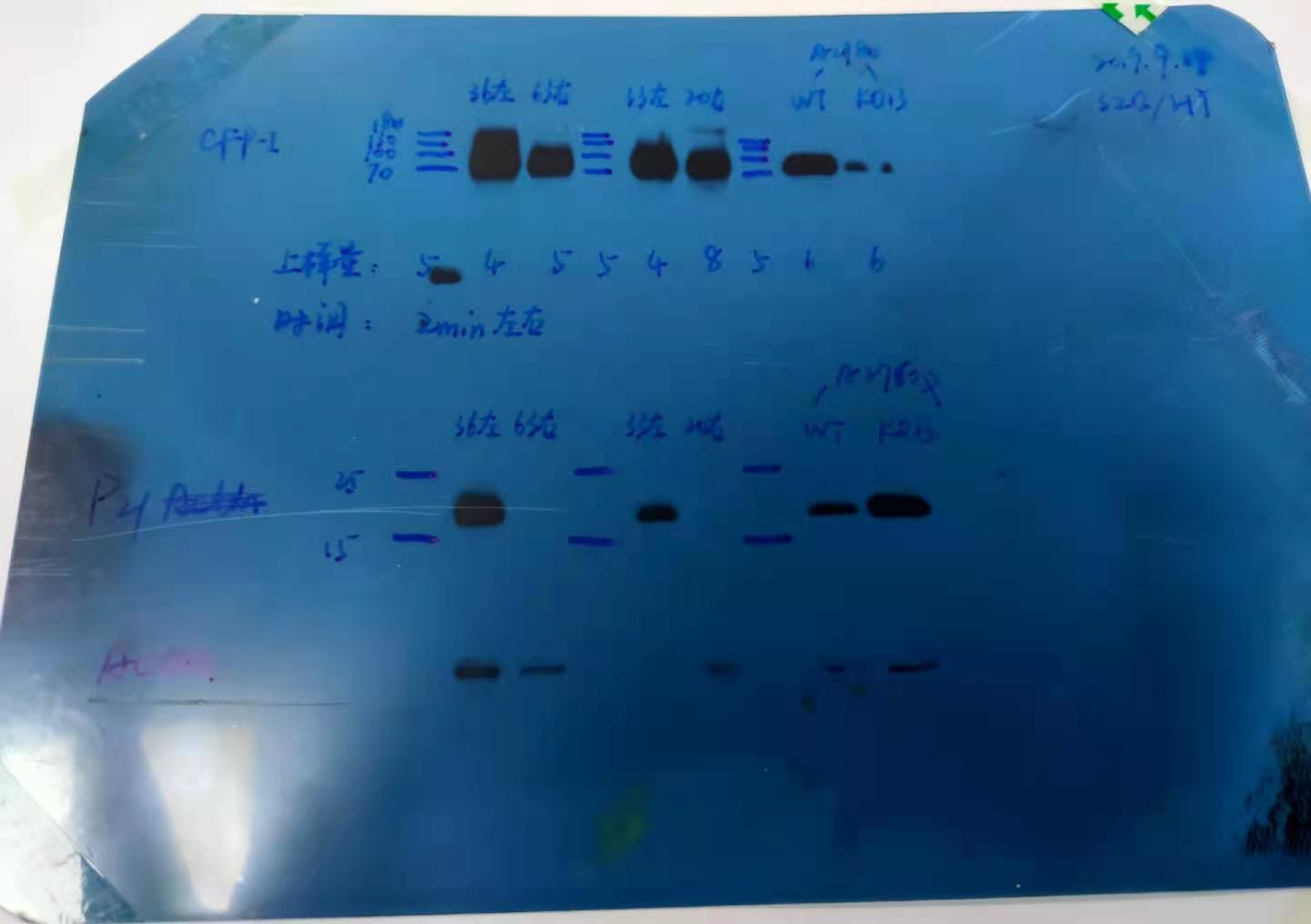

WT KO13
上样量： 5 4 5 5 4 8 5 6 6
时间： 2min左右
WT KO13
25
15

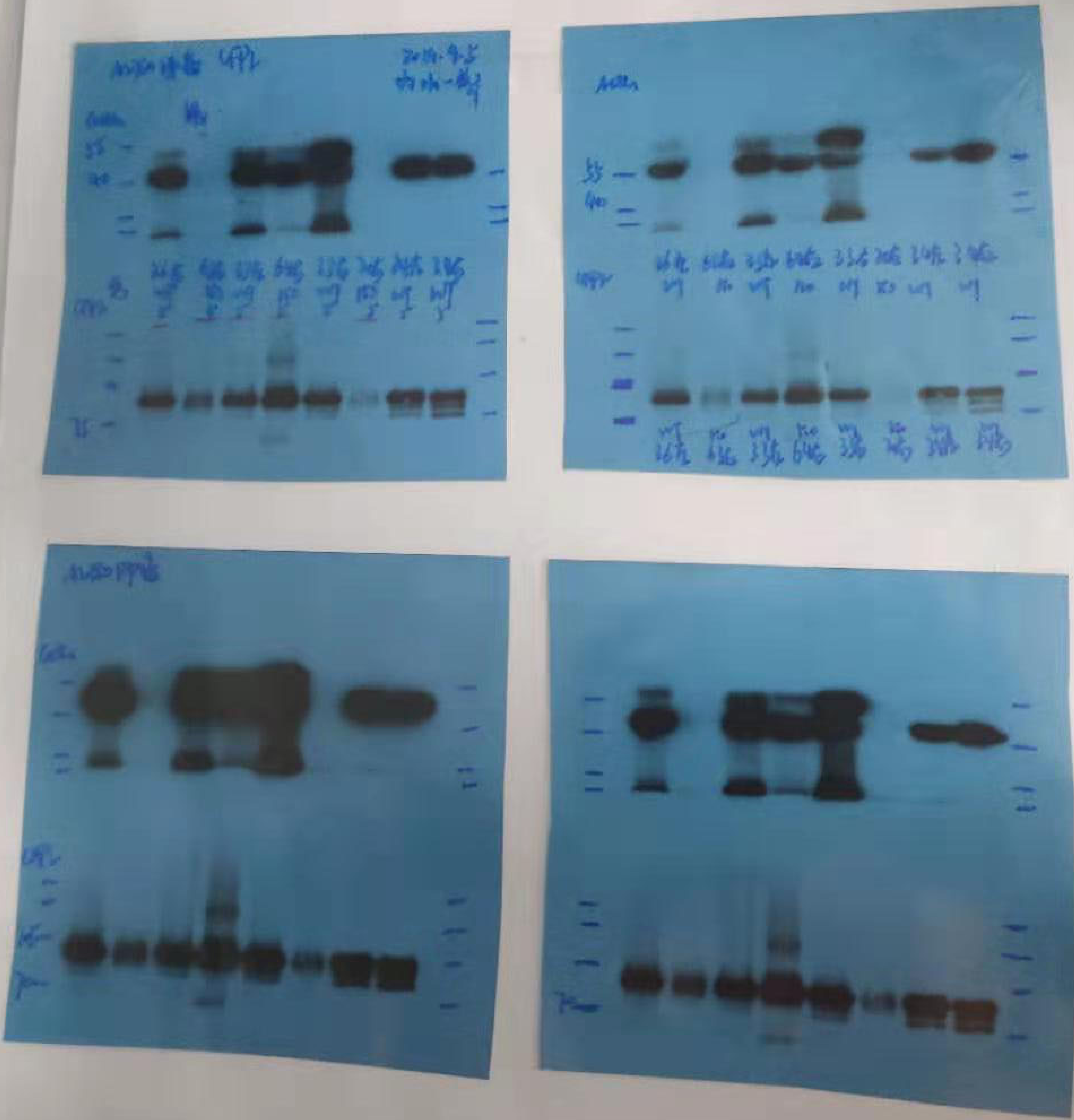

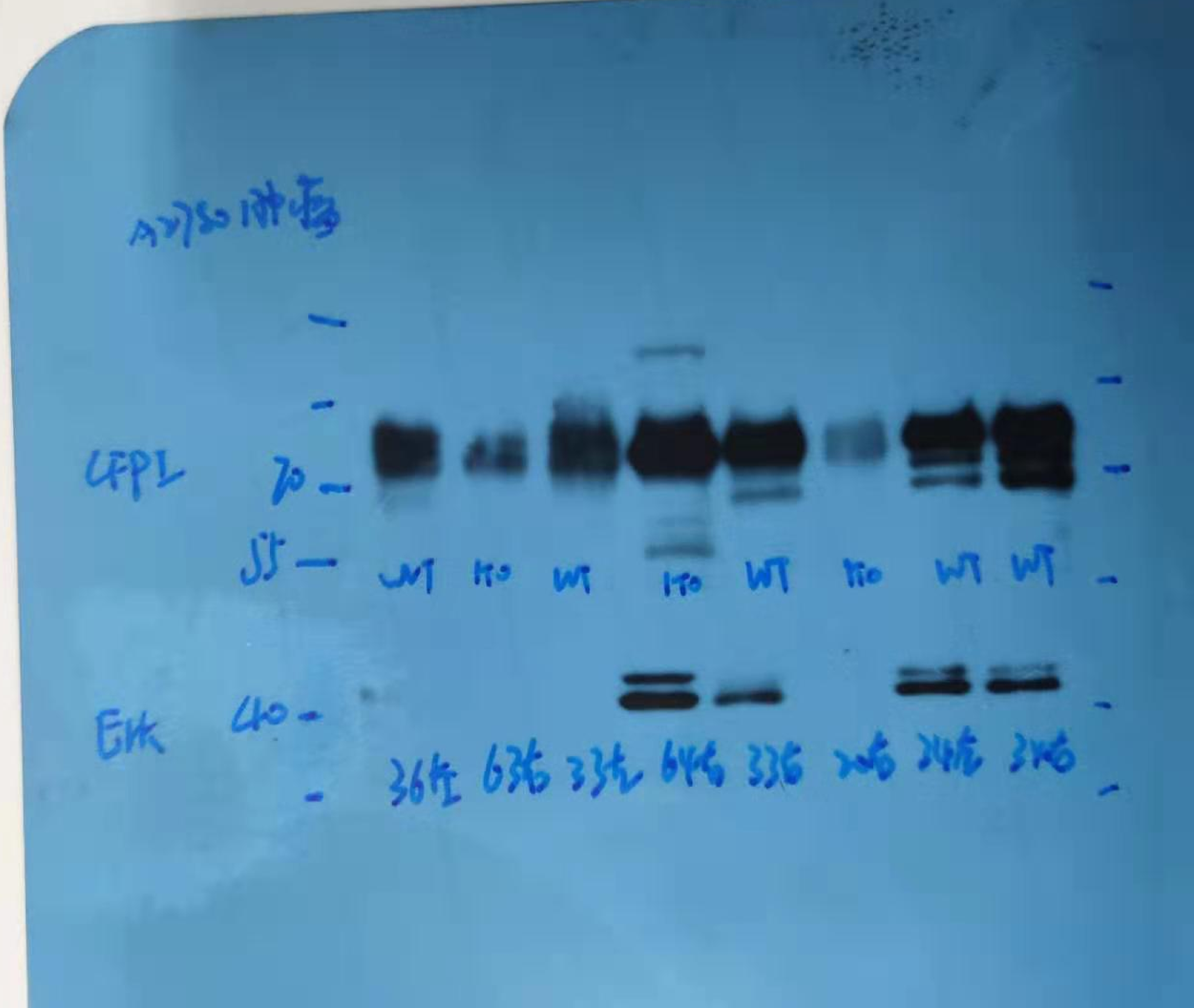
70
55
40
WT
KO
WT
KO
WT
KO
WT
WT
Erk

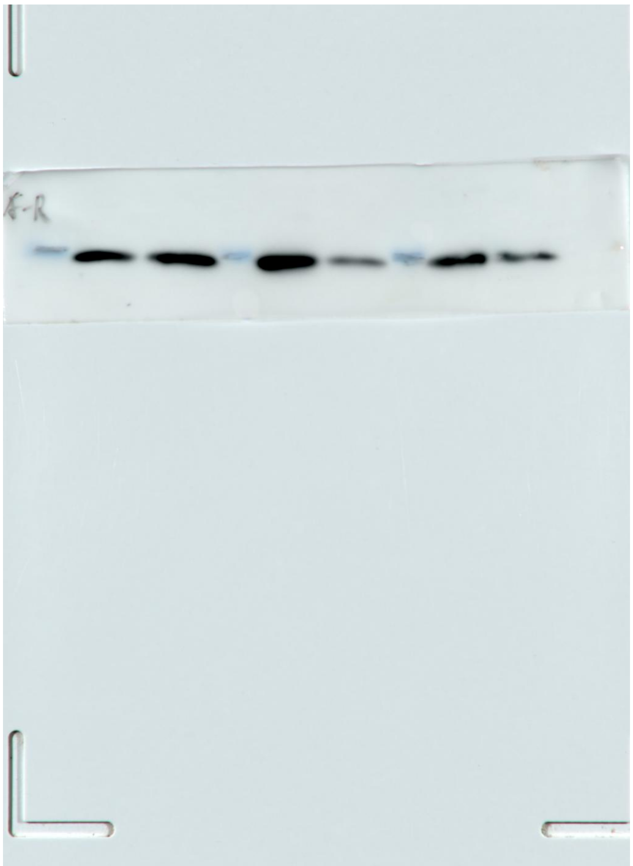

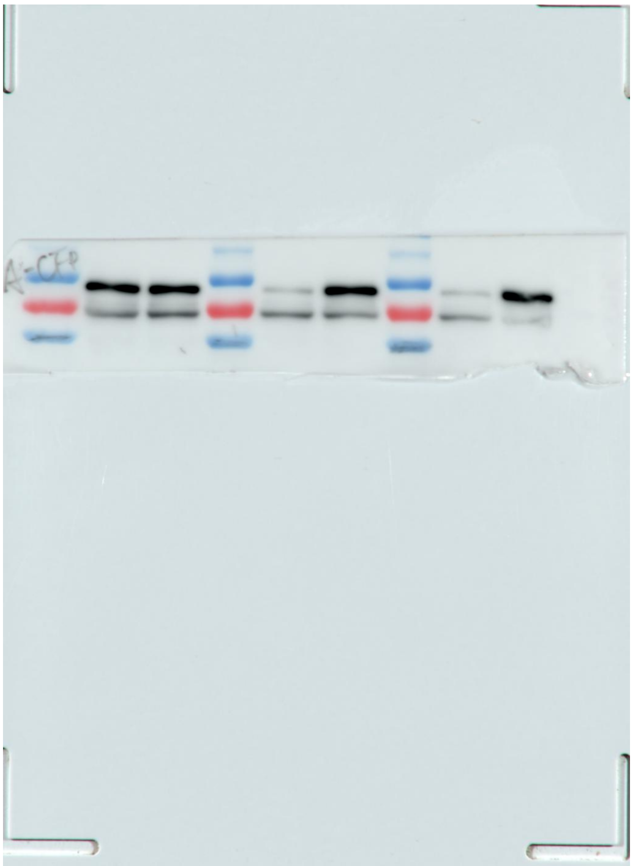

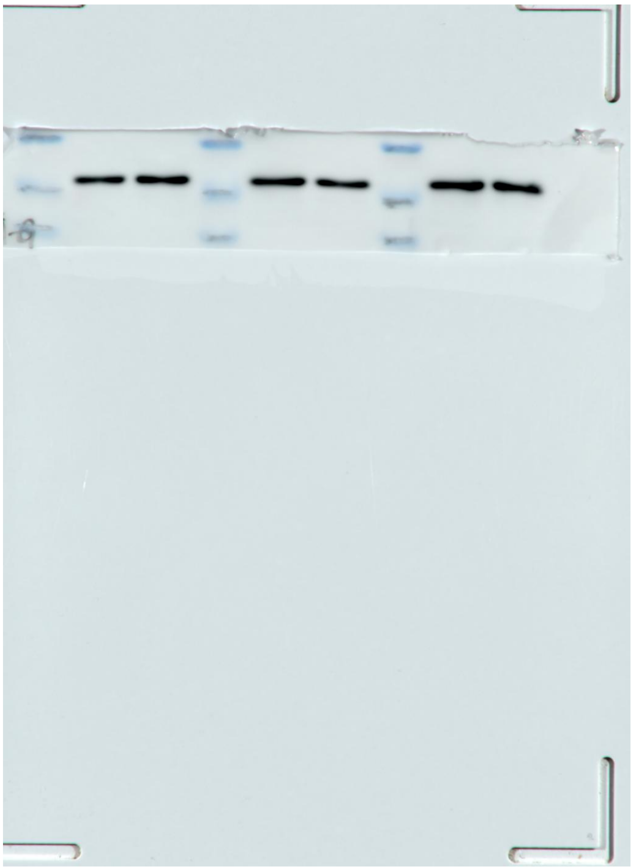

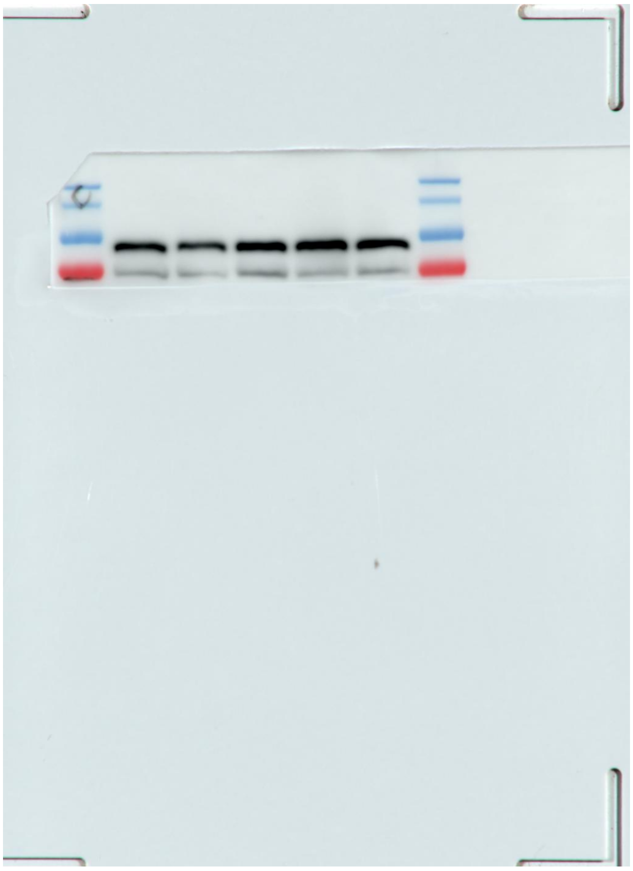

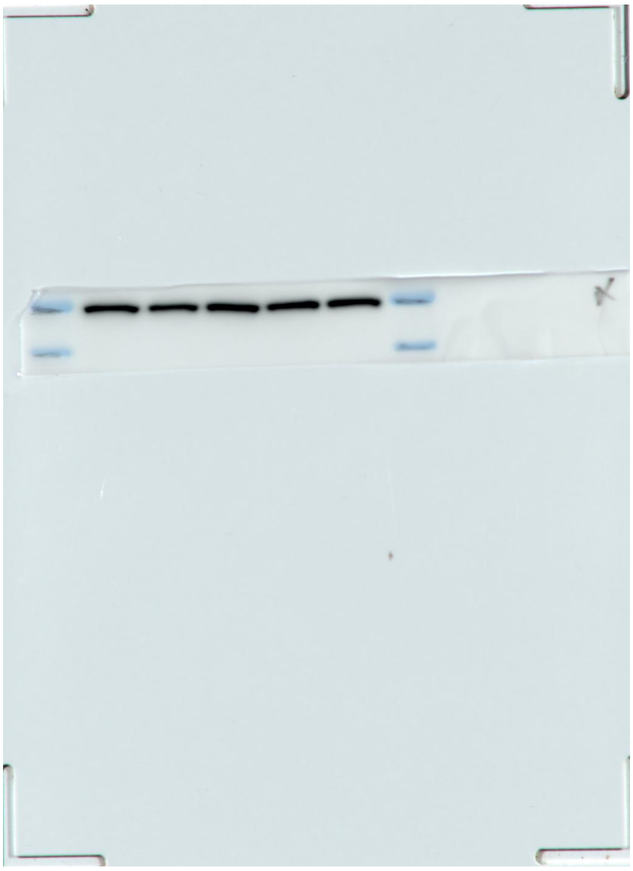

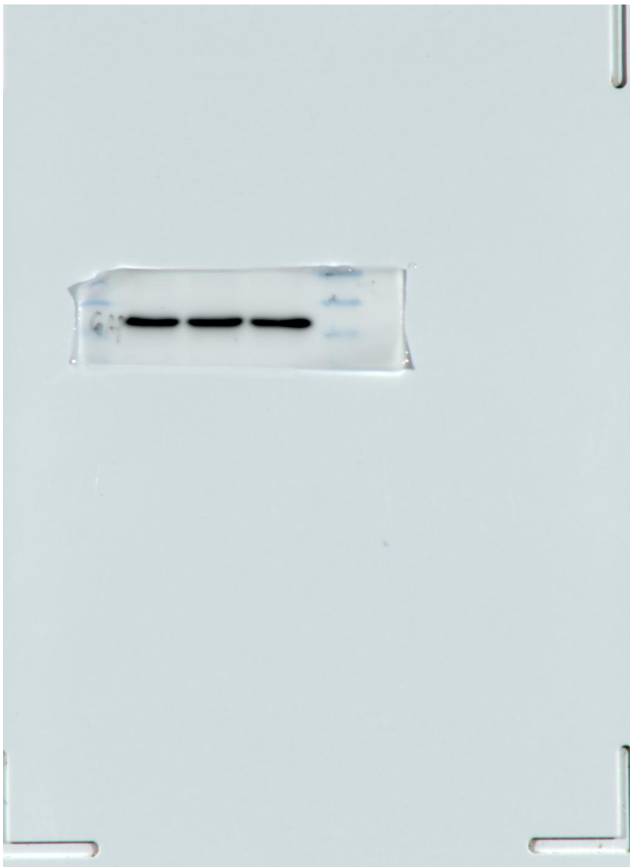

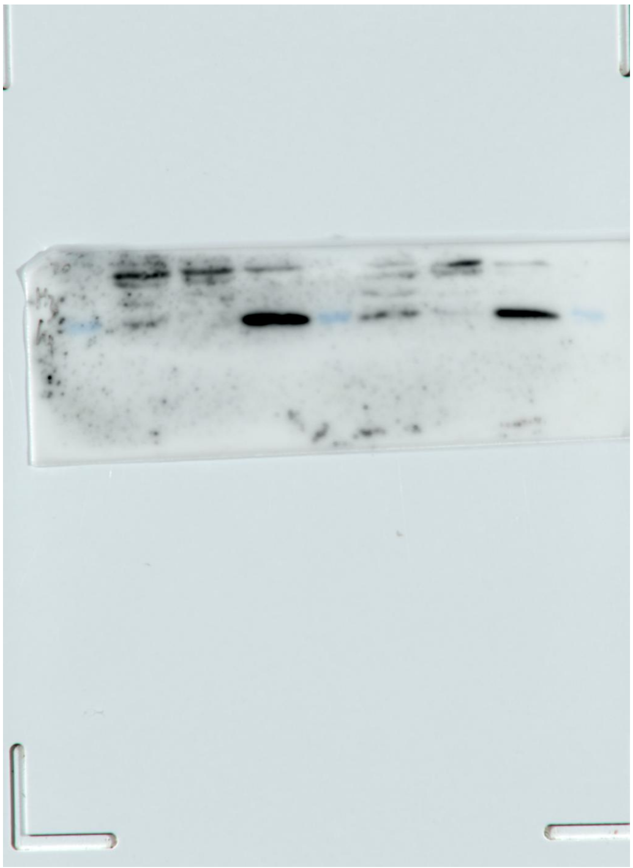

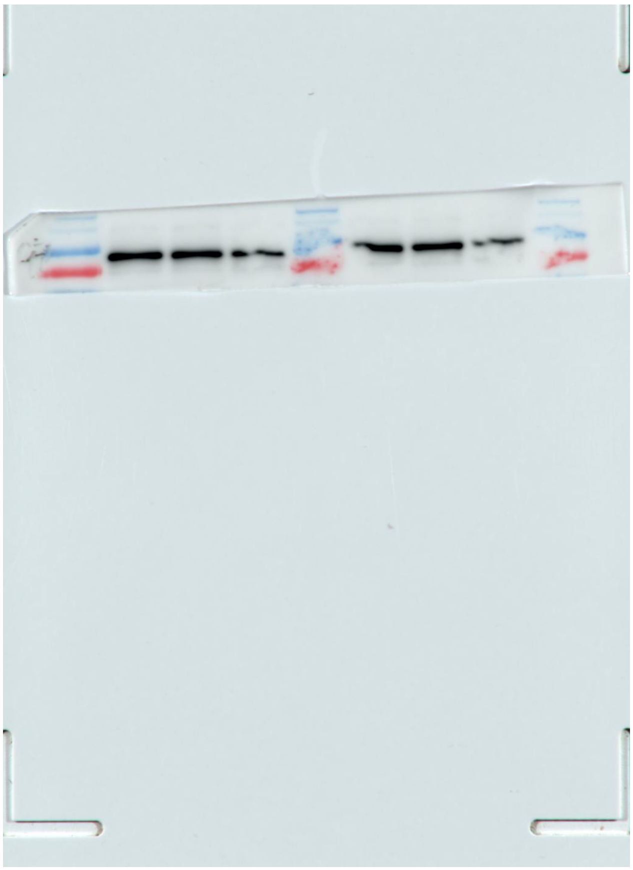

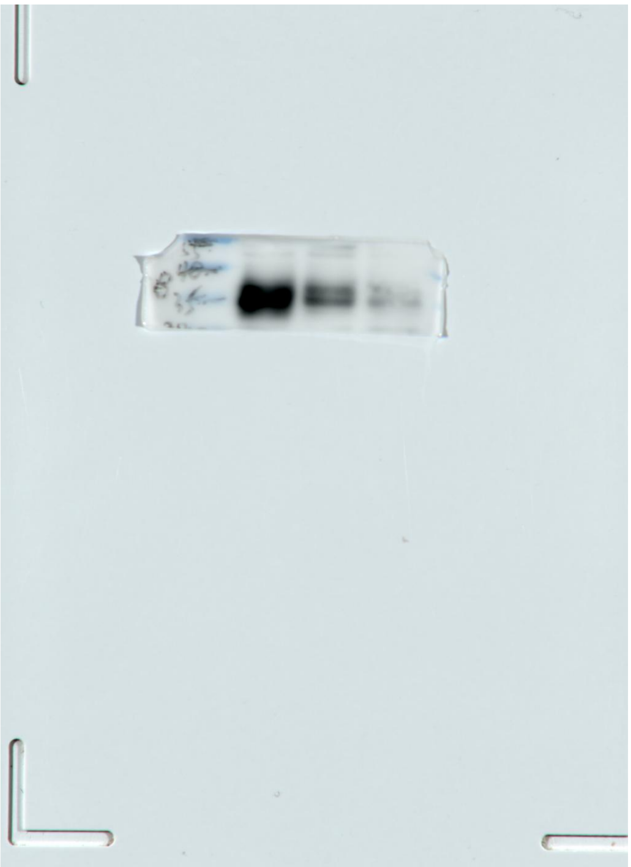

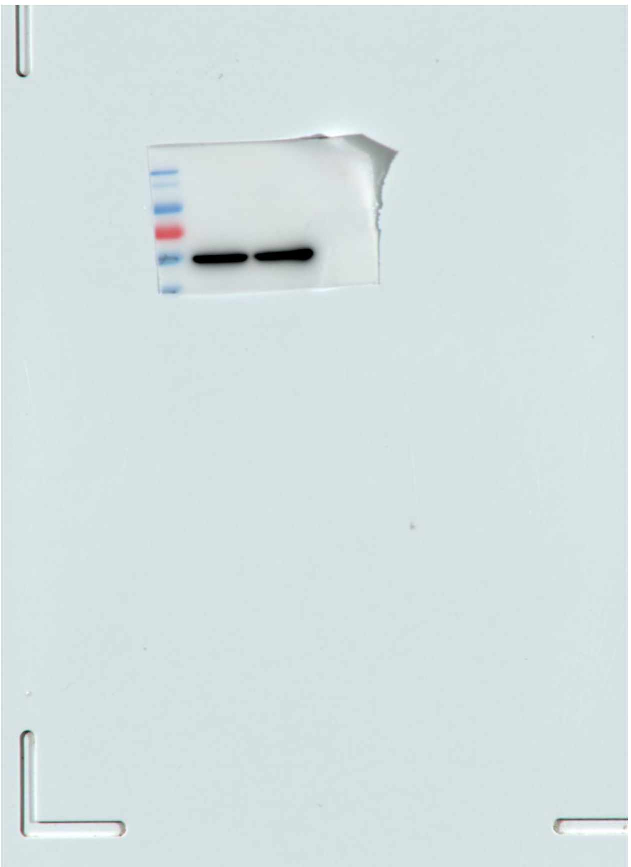

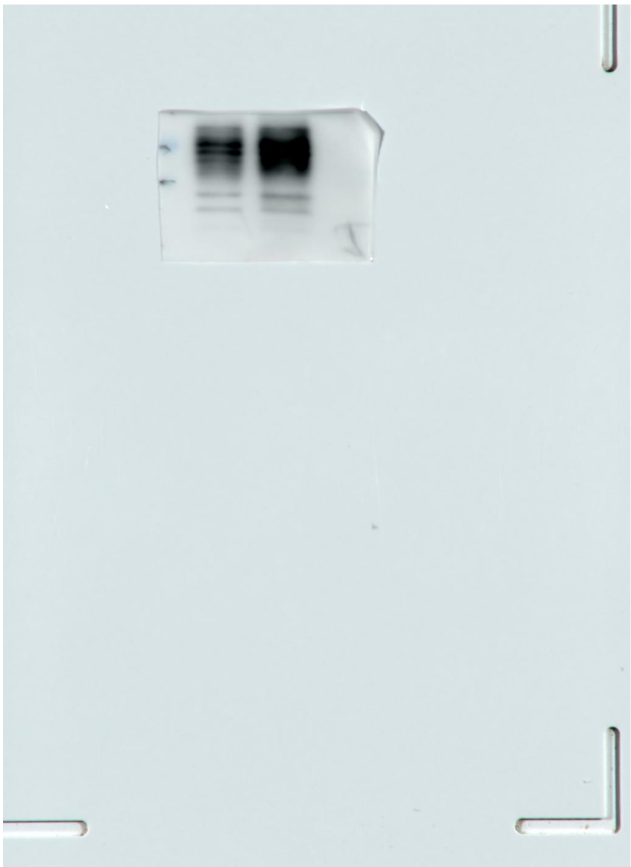

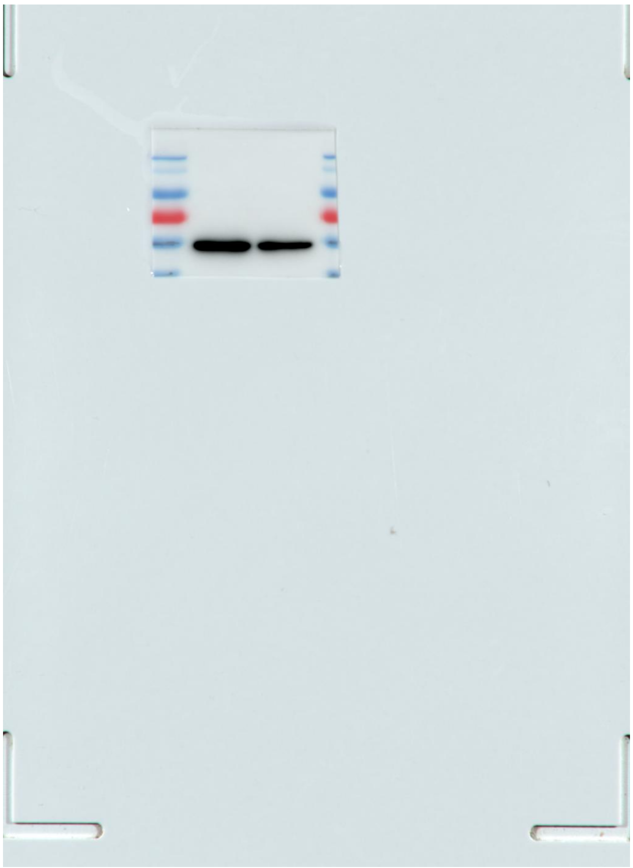

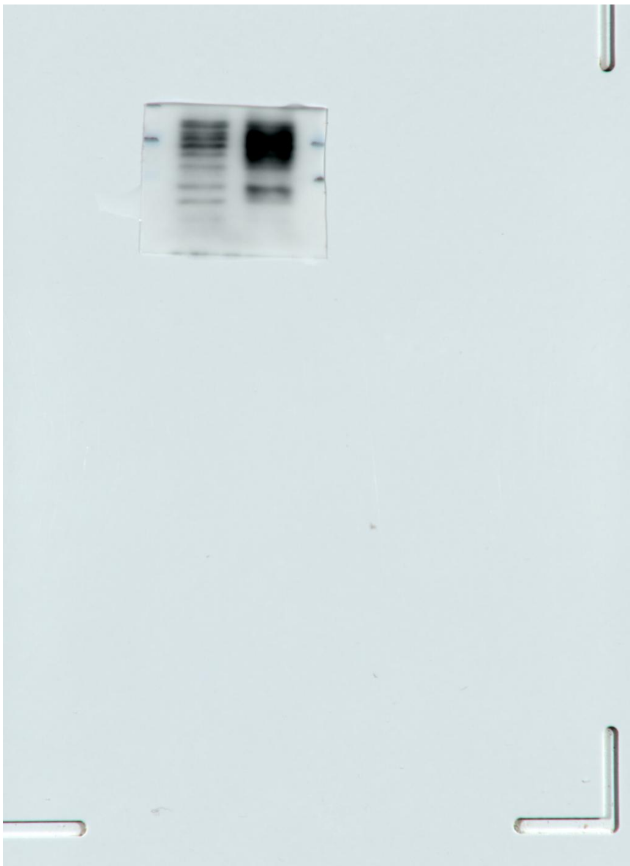

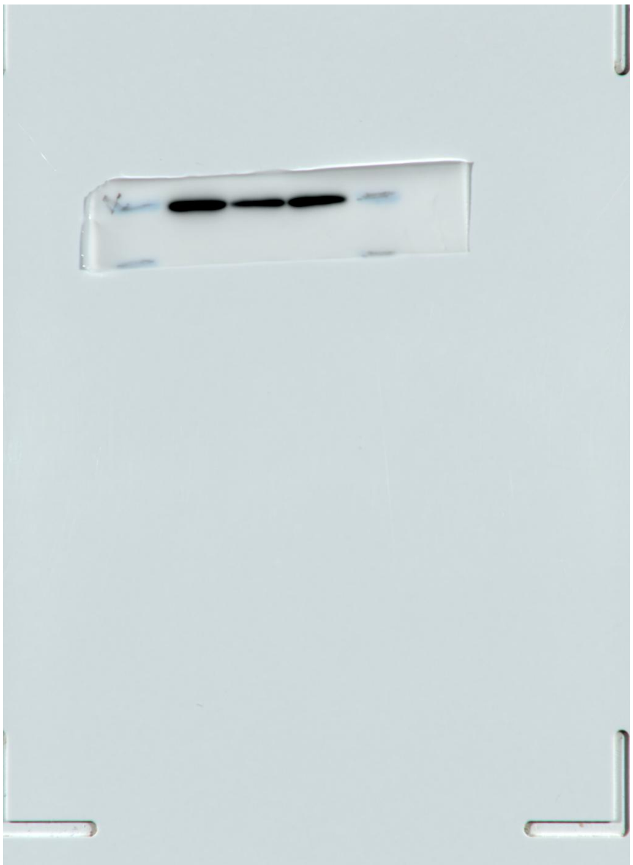

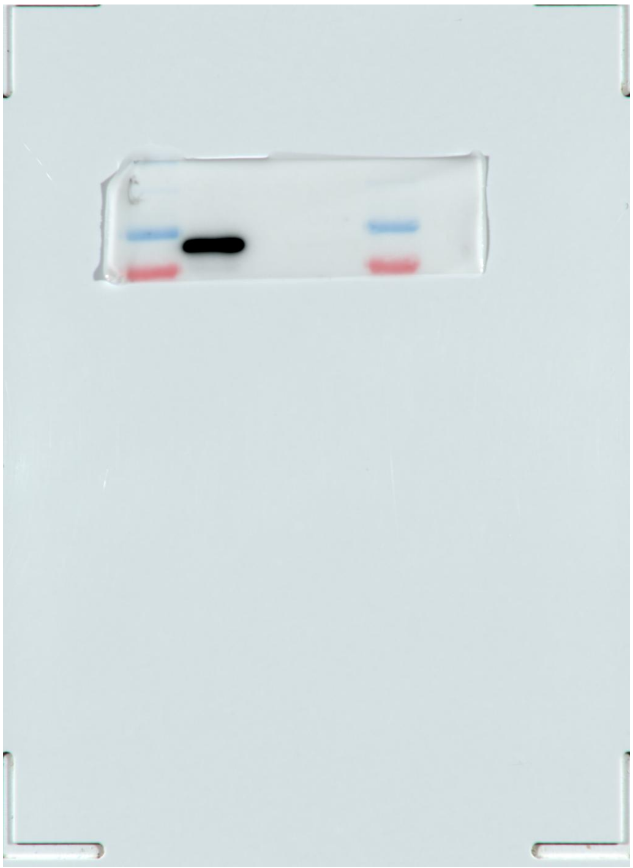

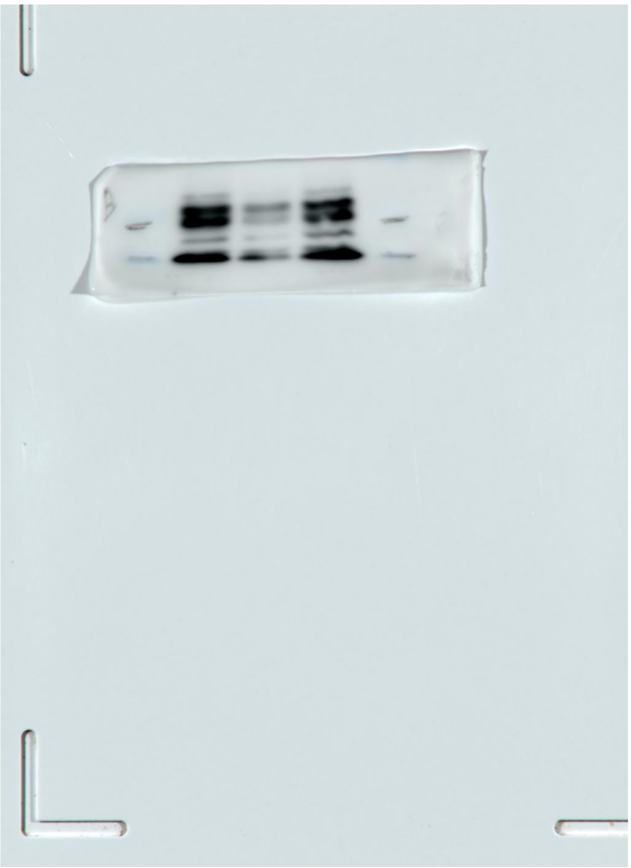

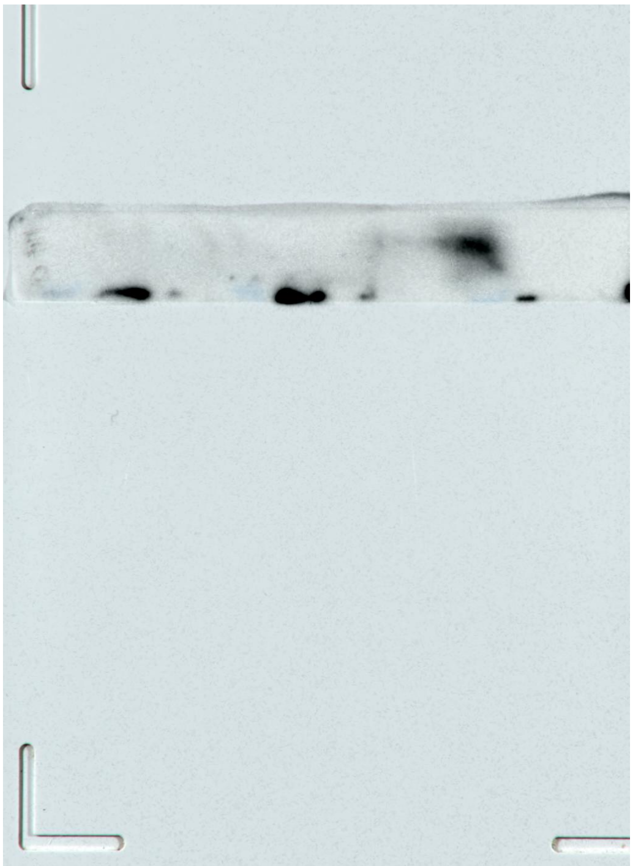
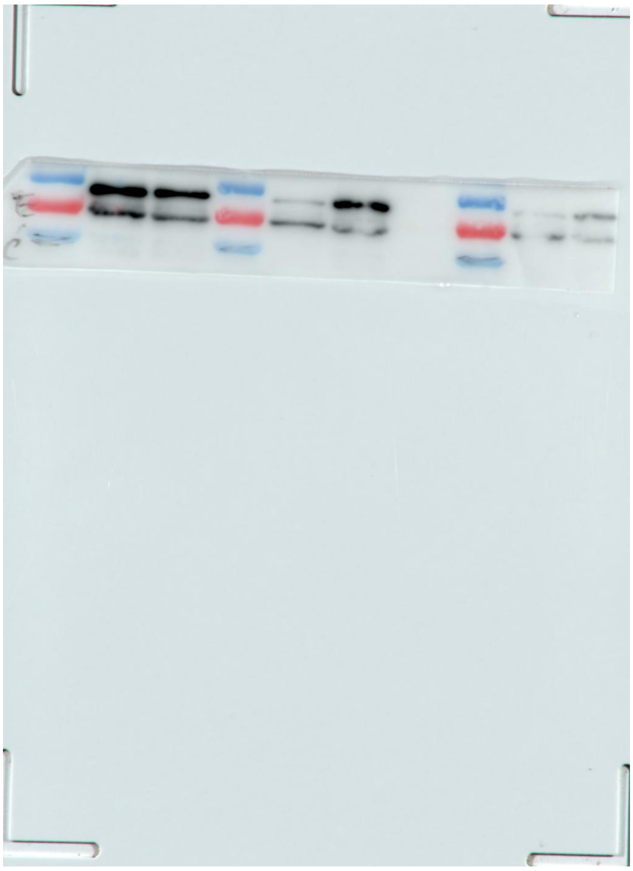

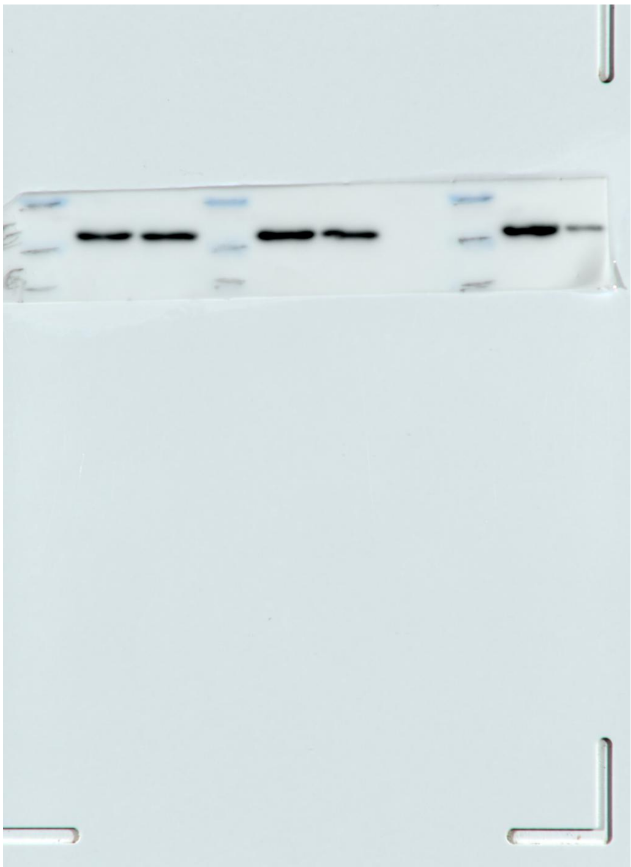

Supplement: Supplementary file 8 — Dataset 2 [file 41417_2022_503_MOESM8_ESM.pdf]
